# Mapping the Human Platelet Lipidome Reveals Cytosolic Phospholipase A_2_ as a Regulator of Mitochondrial Bioenergetics during Activation

**DOI:** 10.1016/j.cmet.2016.04.001

**Published:** 2016-05-10

**Authors:** David A. Slatter, Maceler Aldrovandi, Anne O’Connor, Stuart M. Allen, Christopher J. Brasher, Robert C. Murphy, Sven Mecklemann, Saranya Ravi, Victor Darley-Usmar, Valerie B. O’Donnell

**Affiliations:** 1 Institute of Infection and Immunity and Systems Immunity Research Institute, School of Medicine, Cardiff University, Cardiff CF14 4XN, UK; 2 School of Computer Science and Informatics, Cardiff University, Cardiff CF14 4XN, UK; 3 Department of Pharmacology, University of Colorado Denver, Aurora, CO 80045, USA; 4 Department of Pathology, University of Alabama at Birmingham, Birmingham, AL 35294, USA

## Abstract

Human platelets acutely increase mitochondrial energy generation following stimulation. Herein, a lipidomic circuit was uncovered whereby the substrates for this are exclusively provided by cPLA_2_, including multiple fatty acids and oxidized species that support energy generation via β-oxidation. This indicates that acute lipid membrane remodeling is required to support energetic demands during platelet activation. Phospholipase activity is linked to energy metabolism, revealing cPLA_2_ as a central regulator of both lipidomics and energy flux. Using a lipidomic approach (LipidArrays), we also estimated the total number of lipids in resting, thrombin-activated, and aspirinized platelets. Significant diversity between genetically unrelated individuals and a wealth of species was revealed. Resting platelets demonstrated ∼5,600 unique species, with only ∼50% being putatively identified. Thrombin elevated ∼900 lipids >2-fold with 86% newly appearing and 45% inhibited by aspirin supplementation, indicating COX-1 is required for major activation-dependent lipidomic fluxes. Many lipids were structurally identified. With ∼50% of the lipids being absent from databases, a major opportunity for mining lipids relevant to human health and disease is presented.

## Highlights

• The complete lipidome of resting and agonist-activated human platelets is presented
• Low-dose aspirin supplementation profoundly affects the platelet lipidome
• Significant diversity in the lipidomes of three unrelated individuals is seen
• Cytosolic phospholipase A2 provides lipids for mitochondrial oxygen consumption

## Graphical Abstract

**Figure:**
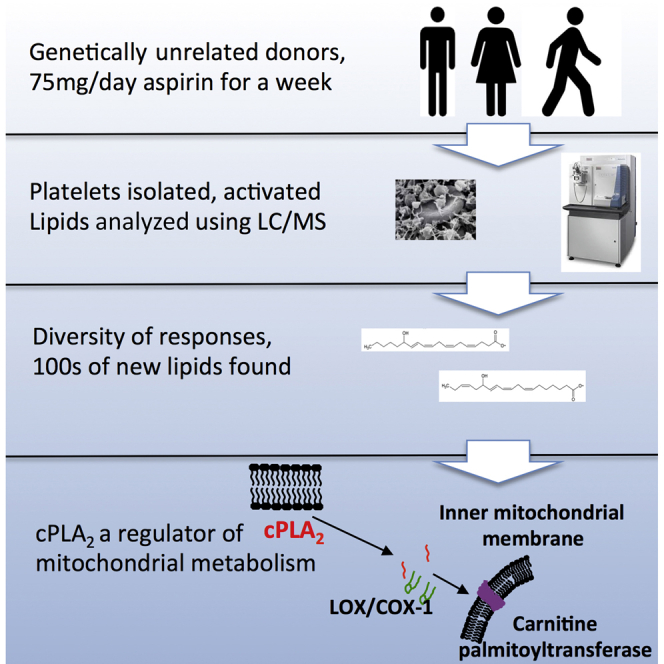

## Teaser

Slatter et al. characterize the lipidomic network of human platelets. Besides the characterization of nearly 200 oxidized species, the resource shows that remodeling of the membrane via phospholipase activity provides energy substrates for respiration. The findings demonstrate a direct link between innate immunity and mitochondrial bioenergetics in human platelets.

## Introduction

Lipids play essential structural roles, act as nutrients, and control a broad range of physiological and pathophysiological events in cells. While several lipid families are well characterized at the molecular level, the total diversity and number of unique lipids in cells and how they change during cellular activation and differ in individuals are unknown. This hampers integration of lipidomics into systems biology, and addressing it will aid (1) fundamental understanding of lipid biology, (2) identification of drug targets for therapy, and (3) discovery of lipid biomarkers from disease cohorts. This is particularly relevant for identification of low-abundance bioactive lipids that are not detected by widely used high-throughput lipidomics.

Lipidomic methods usually target known families of lipids using mass spectrometry and do not “mine” the vast unknowns contained in mass spectrometry datasets. Previous estimates suggested mammalian cells contain 2,000–100,000, with a theoretical number of possible lipids at 100,000–500,000, but experimental attempts to robustly define this have not been undertaken (van Meer, 2005, Yetukuri et al., 2008). We developed a lipidomic workflow that maximizes coverage, ensuring that many low-abundance lipids were detected and verified (LipidArrays), a step essential for discovery of signaling lipids. This required multiple chromatographic separations, high-resolution mass spectrometry, and in-house generation of bioinformatic tools with both automatic and painstaking manual verification of data.

Platelets are central players in hemostasis, have emerging roles in cancer and immune regulation, and robustly activate lipid metabolism during thrombin-dependent aggregation. They are the therapeutic target of low-dose aspirin, via inhibition of cyclooxygenase-1 (COX-1)-dependent formation of thromboxane (TX). Aspirin at platelet-selective doses may prevent cancer metastasis in humans (Langley and Rothwell, 2013, Rothwell, 2013a, Rothwell, 2013b), through mechanisms that could involve lipid signaling. Last, COX-1-derived eicosanoids are mediators of pain, cancer metastasis, fever, vasoconstriction, and platelet activation. Thus, we sought to characterize the platelet COX-sensitive lipidome as a model system of relevance for human health and disease and to use this to uncover lipidomic control networks.

Lipids were mapped under basal and thrombin-activated conditions, with or without in vivo aspirin supplementation (75 mg/day for 7 days). The results indicate that the human platelet lipidome is complex and heterogeneous, with profound changes on activation and in vivo aspirin inhibition. In particular, 192 fatty acids (FAs) and oxidized phospholipids (oxPLs) that support coagulation were structurally identified, including many lipids. FA formation required cytosolic phospholipaseA_2_ (cPLA_2_), which then fed into oxidative phosphorylation via β-oxidation. The activation of cPLA_2_ by thrombin was also revealed to be an energy-requiring process itself. Last, energy from FA oxidation was required for maintaining phospholipid asymmetry. These data link acute lipidomic flux with metabolic processes during innate immunity. Finally, the large numbers of unknowns represent a significant opportunity for lipid discovery.

## Results

### Developing a Comprehensive Method to Analyze the Complete Platelet Lipidome

To determine the complete lipidome, high-resolution mass spectrometry was used to maximize detection of lipids with close *m/z* values, and extensive chromatography was used to separate isobaric species and enable detection of low-abundance lipids. New software was generated, and datasets were also subject to painstaking manual verification. The approach, including bioinformatic and technical aspects, is described in full in Supplemental Information.

### Characterizing the Size and Diversity of the Platelet Lipidome in Three Genetically Unrelated Donors under Basal Conditions

**Figure 1:**
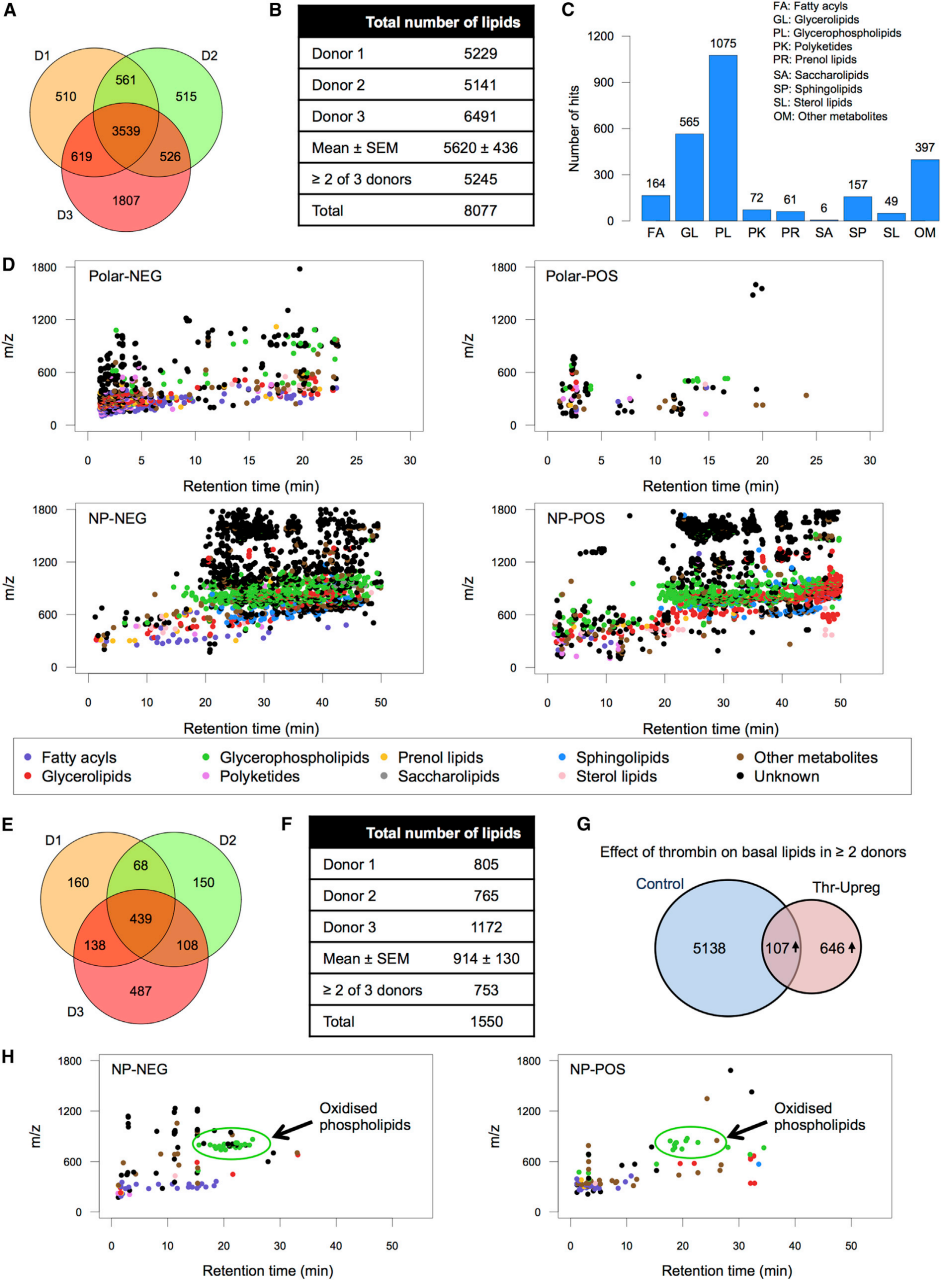
The Resting Human Platelet Lipidome and How It Changes upon Thrombin Activation Platelet lipid extracts from three genetically unrelated donors (D1–D3) were analyzed by LC-FTMS on the Orbitrap Elite, at 60,000 resolution, then processed using SIEVE 2.0 followed by in-house-generated software as described in Supplemental Information. (A) Venn diagram showing the number of ions in each donor lipid isolate. Common and donor specific ions are demonstrated. (B) Table showing the number of ions in each donor and mean, total and lipids common to two or more donors. Calculated from (A). (C) Predominant lipid classes in platelets. Ions were grouped according to the most predominant classification family based on classifications in databases. (D) Scatter diagrams showing elution of lipids from polar or non-polar (NP) columns, in either negative- or positive-ion mode. Lipids are color-coded according to classification from databases. To view in interactive mode, download Data S2, which allows zooming and viewing of individual accurate *m/z* values for all ions. Putative identifications for all are in Data S1. (E–H) The thrombin-stimulated lipidome. Platelet lipid extracts from three genetically unrelated donors (D1–D3), before and after activation for 30 min using 0.2 U/mL thrombin, were analyzed as above, showing ions that are elevated at least 2-fold. (E) Venn diagram thrombin-upregulated lipids in two or more donors. (F) Total number of lipids in each donor, shown individually (G) The effect of thrombin on basal platelet lipids. Lipids that are newly detected or elevate at least 2-fold in two or more donors are shown. Out of 5,245 lipids, only 107 were upregulated, with an additional 646 appearing only in the thrombin-activated samples. (H) Scatter diagrams showing elution of lipids from polar or NP columns, in either negative- or positive-ion mode. Putative identifications for all are in Data S1.

Three genetically unrelated volunteers provided a baseline blood sample, and a second was provided following aspirin ingestion (75 mg/day, 7 days). First, we identified donor-specific total lipids at baseline. Platelets from all donors contained a total of 8,077 lipids, of which 5,245 (65%) were common in two or more donors. A single individual’s platelets showed 5,620 ± 436 species (mean ± SEM, n = 3; Figures 1A and 1B). Full details of all ions detected in two or more donors are in Data S1. A degree of variation between individuals is noted, with each donor being ∼84% the same as two or more donors, or 63.7% the same as all three (Figures 1A and 1B).

Results were queried against Human Metabolome Database (HMDB), LipidMaps, LipidHome, and METLIN, with tolerance of 5 ppm (Figures S1A and S1B) (Foster et al., 2013, Smith et al., 2005, Sud et al., 2007, Wishart et al., 2013). Many identified with multiple names, in a few cases with HMDB, as many as 200–300 (Figure S1B). Thus, to putatively assign an ion to a lipid family, we used the most common class for each. Most lipid classes were detected in platelets, with glycerolipids (GLs) and glycerophospholipids (PLs) predominating (Figure 1C; Data S1). METLIN does not assign molecules to a class, thus “other metabolites (OM)” refers to METLIN-identified ions of unknown class.

Scatter diagrams show the range of lipids contained in platelets (Figure 1D). Several lipids of unknown structure group together, typically at higher mass ranges and hydrophobicity. These and many other unknowns (total 51%) leave a wealth of lipids to be discovered and characterized. To allow detailed examination of the data, we created interactive scatter diagrams using GoogleVis that allow users to mine the unknowns, with the ability to zoom and identify based on *m/z* and retention time (Data S2). Crosschecking with Data S1 provides putative identifications for *m/z* values of interest. For example, note the large group of glycerophospholipids (PLs) in green in both non-polar negative and non-polar positive, and glycerides (GLs; including triglycerides) in red in the non-polar-positive dataset.

### Characterizing the Platelet Lipidome on Thrombin Activation

Next, we examined global lipid changes on thrombin activation, analyzing lipids that upregulate ≥2-fold. Thrombin caused a selected subset of lipids to be generated, 1,550 in all three donors, with 753 lipids common to two or more donors, and mean of 914 per isolate (Figures 1E and 1F; Data S1). Of these, 86% were absent basally and only detected on thrombin activation (Figure 1 G). Within all upregulated lipids, 420 were increased by >10-fold. The most predominant upregulated lipids were putatively identified as FAs (including eicosanoids) and PLs (Figures S1C and S1D). Principal component analysis (PCA) and a heatmap show the diversity in thrombin responses between donors, with samples separated in both PC1 and PC2, before and after activation (Figures S1E and S1F). Furthermore, the heat map (showing lipids upregulated in two or more donors) demonstrates that each donor elevates groups of individual lipids to different extents (Figure S1E). Scatter diagrams show the pattern of thrombin upregulated lipids found in two or more donors, with individual signatures shown for reference (Figure S2). FAs (purple), including eicosanoids, are strongly represented in negative mode. As for basal lipids, there are similarities and differences between donors, in particular a prominent group of PLs (green) in both positive and negative mode at ∼18–25 min in the non-polar datasets (*m/z* ∼700–900), most of which are oxidized PLs, as shown later (Figure 1H, showing lipids present in two or more donors). The scatter patterns appear unique to each donor, analogous to a fingerprint (Figure S2). How they differ in larger groups of people, and with variables including age, gender, health, disease, and diet, will be the subject of future study. Interactive GoogleVis scatter diagrams for these are in Data S3.

### Full Structural Identification of a Large Group of OxPLs Generated Acutely by Thrombin-Activated Platelets

**Figure 2:**
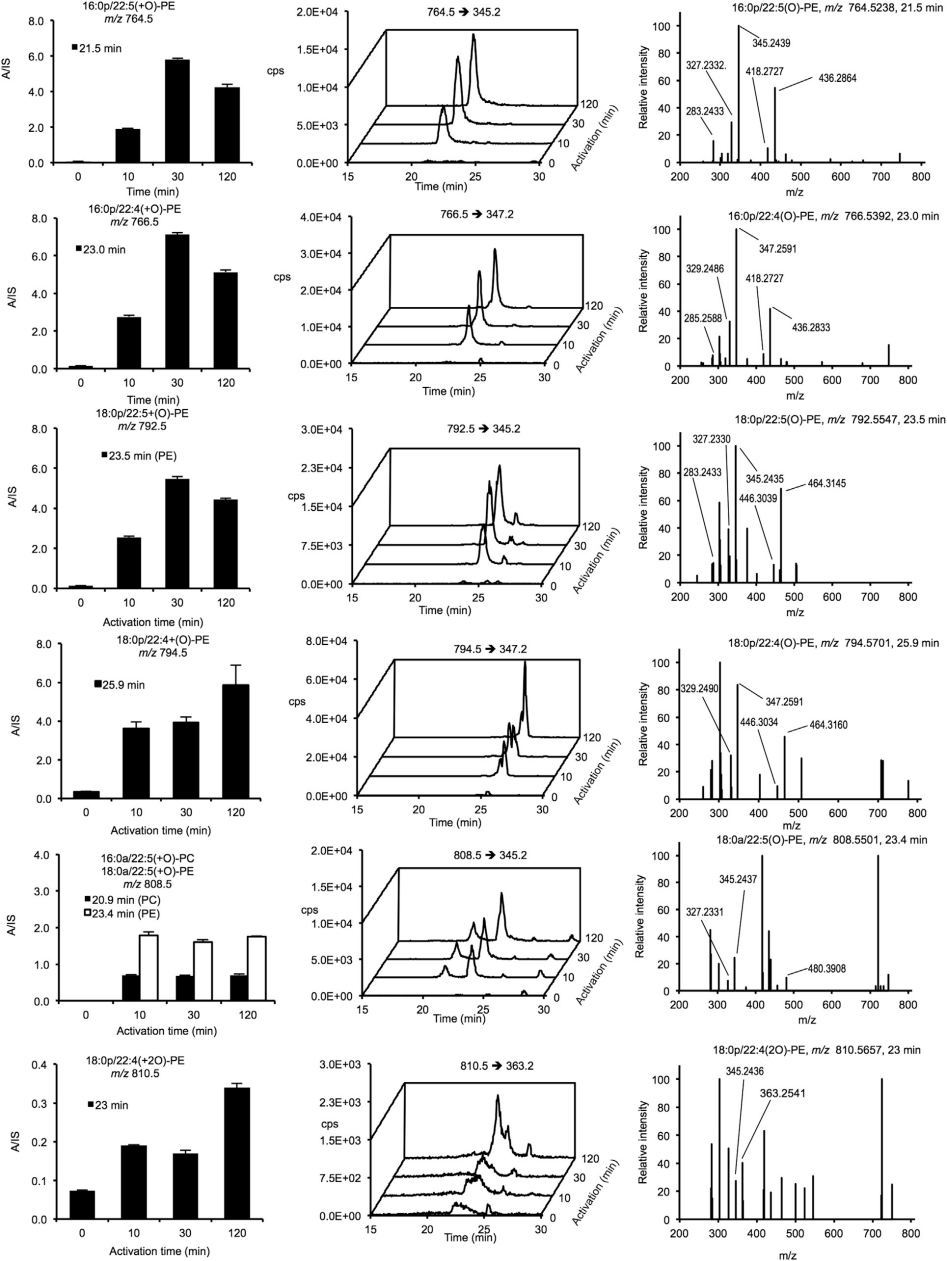
Time Course of Selected OxPL Generation by Human Platelets in Response to Thrombin Washed platelets were activated using 0.2 U/min thrombin for varying times, then lipids were extracted and analyzed using a 4000 Q-Trap as described in Experimental Procedures, and with parent to daughter transitions as listed on the chromatograms. Left: time courses of integrated areas compared to internal standard (n = 3, mean ± SEM). Middle: sample chromatograms showing almost absence of lipids at 0 min followed by upregulation from 10 to 120 min. Right: MS/MS spectra for all lipids shown. More detailed MS/MS data are available in Data S4.

**Figure 3:**
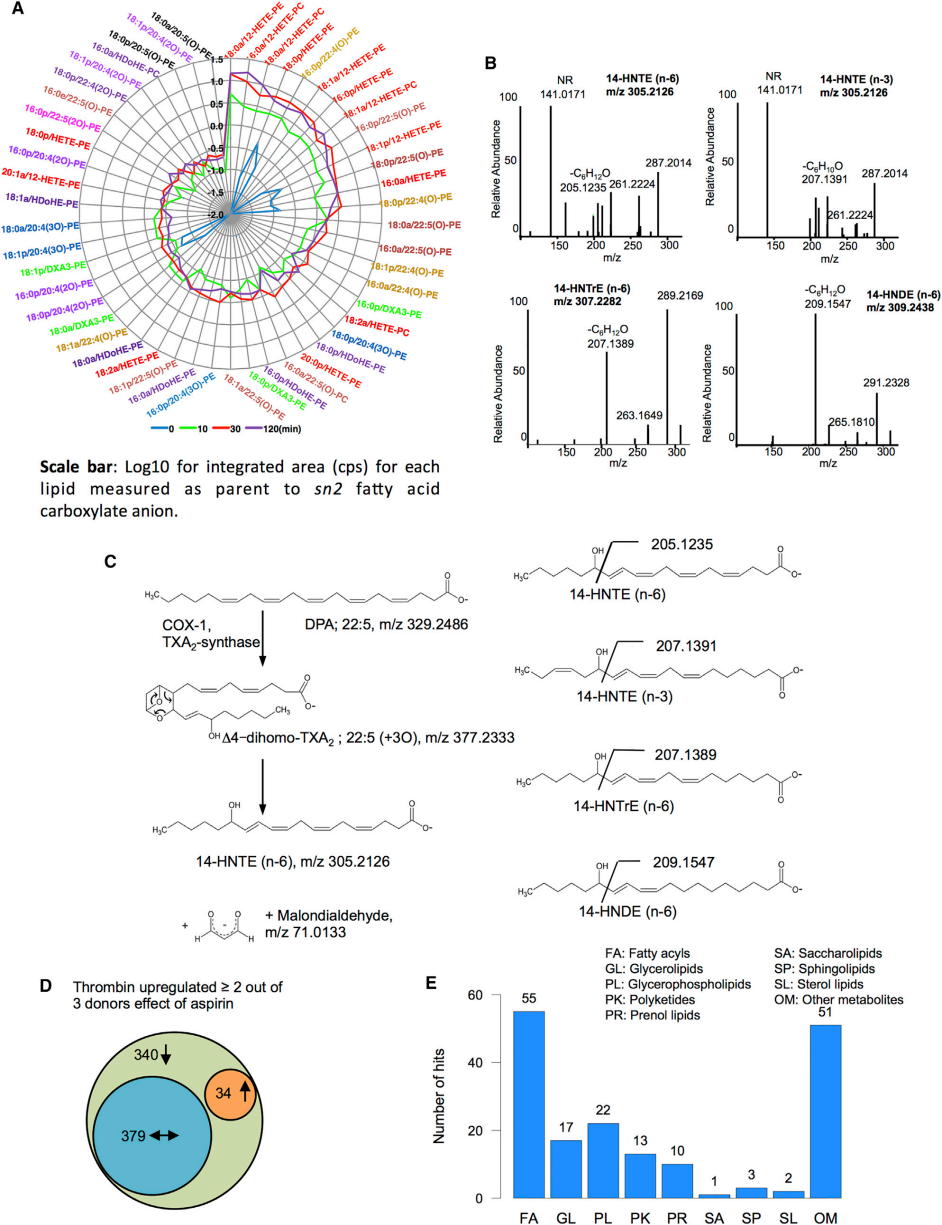
Radar Plot Showing Relative Levels of OxPLs Generated by Platelets, Identification of Four Oxidized FAs Generated via COX-1/TXS, and Effect of Aspirin on the Platelet Lipidome (A) Radar plot showing time course of the 52 most upregulated oxPLs. The lipids are color-coded based on sn2 fatty acid, and show that monohydroxylipids of AA, 22:4, and 22:5 predominate (red, brown, and burgundy labels are on right-hand side of plot), while poly-hydroxylated FAs are less abundant. (B) MS/MS spectra of four lipids identified as 14-HNTE (n6), 14-HNTE (n3), 14-HNTrE (n6), and 14-HNDE (n6). Note a major ion, identified in (C). NR, not related (an ion from an unrelated isobaric lipid). (C) Mechanism of formation of 14-HNTE (n6) (left) and fragmentation of all four isomers (right). (D) Aspirin blocks generation of 45% of platelet lipids upregulated by thrombin. Platelet lipid extracts from three genetically unrelated donors with or without 7 days on 75 mg/day aspirin were analyzed after activation for 30 min using 0.2 U/mL thrombin, as in Figure 1. Ions noted in Figure 1G to be upregulated by at least 2-fold in two or more donors are shown. (E) Predominant lipid classes upregulated by thrombin and aspirin inhibited in platelets. Putative ions were grouped according to the most predominant classification family based on classifications in LipidMaps, LipidHome, or HMDB, as for Figure 1C.

Our global analysis putatively identified several lipid families in platelets. Given the large number of ions, full structural validation of all is not feasible. Thus, we concentrated on mapping FAs and PLs that are elevated on thrombin activation. We previously found that platelets acutely generate oxPL via cyclooxygenase (COX) and lipoxygenase (LOX), giving a total of 18 molecular species (Aldrovandi et al., 2013, Morgan et al., 2010, O’Donnell and Murphy, 2012, O’Donnell et al., 2014, Thomas et al., 2010). Herein, a prominent group of ions was noted at 15–25 min on the non-polar column (green in Figure 1H) and initially assigned as PLs via database searching. Further manual interrogation identified 111 oxPLs, indicating a large number of species (Table S2; Data S4). Tandem mass spectrometry (MS/MS) spectra were acquired and compared against LipidMaps and METLIN spectral databases, in tandem with manual interrogation. Assignments were based on the presence of diacyl fatty acid (*sn1*), oxidized fatty acid (*sn2*), neutral loss of ketene with and without water, and deprotonated precursor mass ion or loss of methyl ([M-CH_3_]^−^) for phosphatidylcholines (PCs) in negative ion mode, and many isobaric species are noted indicating positional and regioisomers. To further validate, we monitored the ions in a new donor platelet isolate, using molecular reaction monitoring (MRM) mode on a Q-Trap platform, monitoring parent *m/z* to *sn2* carboxylate anion as daughter ion (Table S3). As examples, time courses for generation of seven phosphatidylcholines (PC) and –ethanolamines (PE) containing 22:4 and 22:5 hydroxy lipids are shown, along with chromatograms and MS/MS spectra (Figure 2). All are virtually absent in resting platelets with immediate rises on activation and levels sustained for at least 2 hr. The top 52 upregulated oxPLs are displayed on a radar plot, which shows the relative abundance of specific families (Figure 3A). Twenty-three of the top 26 are monohydroxy lipids from arachidonic acid (AA), docosapentanoic acid (DPA), docosahexanoic acid (DHA), eicosapentanoic acid (EPA), and eicosatetraenoic acid (ETrA). Less abundant are multiple oxidized species and unsaturated fatty acids containing more than one oxygen group.

### Identification of Fatty Acids and Oxidized Fatty Acids that Are Generated Acutely by Thrombin-Activated Platelets

A number of lipids putatively identified as FAs in Figure 1H, with additional ions eluting primarily on the polar-negative column, were subject to liquid chromatography (LC) MS/MS for identification. Of these, 81 were validated, by comparison with MS/MS spectra at LipidMaps or HMDB, or through manual curation (Table S4; Data S5). Many have never been detected before in human platelets, and include saturated and unsaturated fatty acids, known COX and LOX products, and mono/poly-oxygenated species of unknown origin. Several had daughter ions consistent with prostaglandins (PGs) or eicosanoids and include lipids with fragmentation similar to PGB_2_ and PGF2α.

We highlight four, at *m/z* 309.1538, 307.2282, and 305.2126 (two lipids; Figures 3B and 3C). Their elemental composition indicated four C19-monohydroxy fatty acids, with two, three, or four double bonds, respectively. MS/MS spectra confirmed they were structurally related, showing +2 for several daughter ions, including *m/z* 205, 261, and 287 (Figure 3B; Data S5). Three showed neutral loss of 100 amu, indicating the position of the −OH at C14. The fourth showed loss of 98 due to the presence of a double bond distal to the −OH at C14. This indicates these to be 14-hydroxynonadecatetraenoic, -trienoic and -dienoic acids, respectively, for *m/z* 305, 307, and 309 (14-HNTE, 14-HNTrE, and 14-HNDE, n-6), with an n-3 form also found for 14-HNTE (Figure 3C). These are analogous to 12-hydroxy-5,8,10-heptadecatrienoic acid (12-HHTrE) generated by COX/thromboxane synthase oxidation of AA but instead form via oxidation of 22:5(n6), 22:4(n6), 22:3(n6), and 22:5(n3). We also highlight dioxolane A3 (DXA_3_) identified via extensive gas chromatography/mass spectrometry (GC/MS), MS^3^, and biological studies as a new lipid derived from COX-1. The complete data on this lipid will be presented elsewhere (unpublished data). MS/MS spectra for all 81 FAs are provided in Data S5.

### Aspirin Blocks Selected Thrombin-Dependent Lipid Mobilization in Platelets, but Responses Are Highly Heterogeneous

To block platelet COX-1 in vivo, the same donors were administered 75 mg/day aspirin for 7 days, then platelets were isolated and activated using thrombin. Aspirin blocked TXB_2_ and 12-HHT generation, demonstrating donor compliance (Data S6). Forty-five percent of thrombin-upregulated lipids were decreased by at least 50% by aspirin consumption. Aspirin also upregulated a small group of lipids (34 ions, 4.5%), while just over 50% were unchanged (Figure 3D).

**Figure 4:**
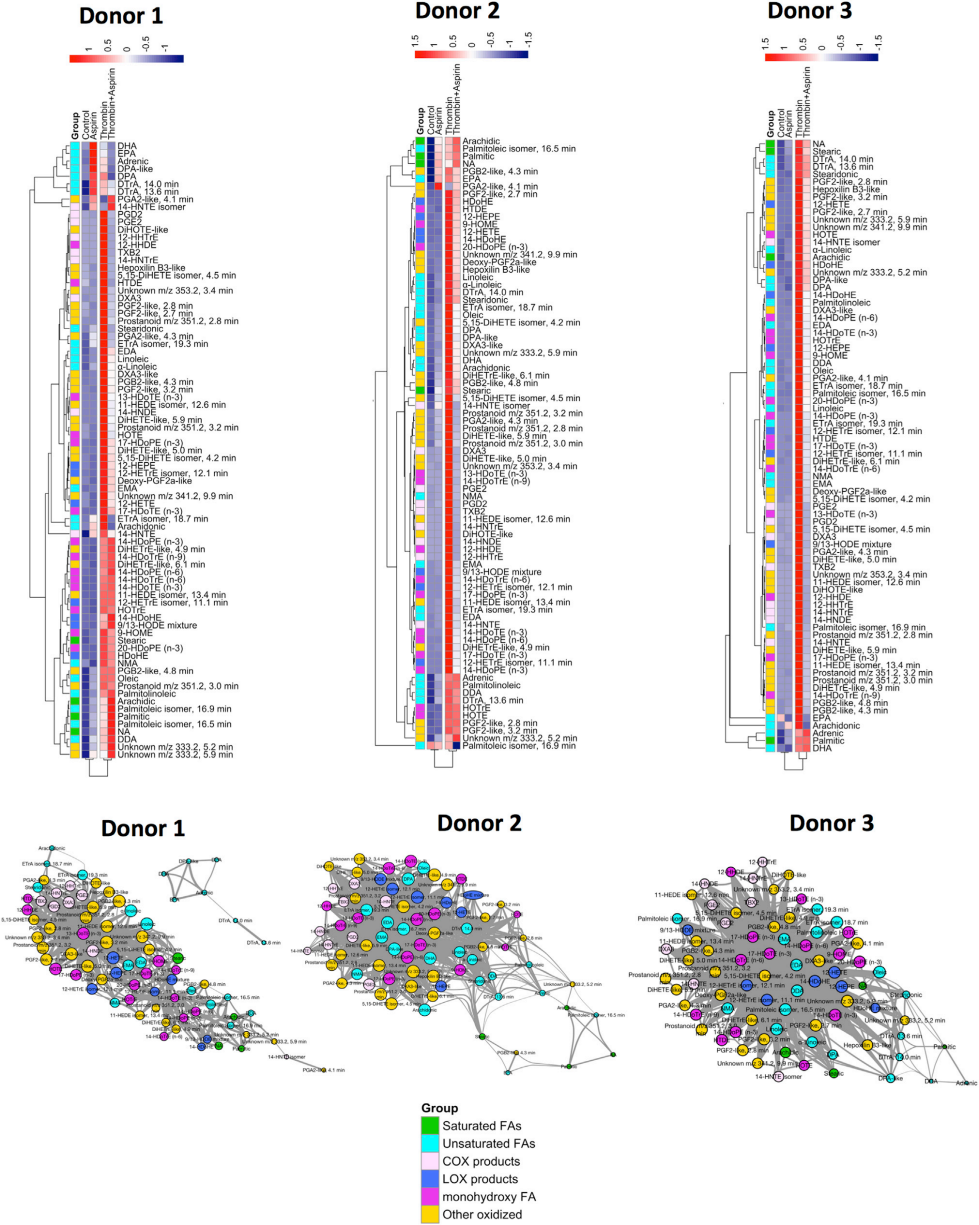
Heatmaps and Network Analysis Showing the Diversity of Thrombin and Aspirin Responses for Free and Oxidized FAs Generated by Three Genetically Unrelated Donors Heatmaps were generated using full-scan FTMS data for each donor using the pheatmap package in R, and lipids are color-coded according to lipid group. Levels of treatment response are represented by a color gradient ranging from blue (decrease in response) to white (no change) to red (increase in response). Lipids are color-coded by group and clustered by similarity in overall response to the treatments. Network analysis was done using Cytoscape 3.2.1. For each donor, pairwise correlations between lipids were calculated in R. Nodes represent individual lipids, and degree (size) is the number of links. Edge thickness represents strength of correlation between nodes. Full data are available in Data S6 for all individual lipids.

Given the therapeutic importance of aspirin, we focused on suppressed ions. Forty-eight percent were absent from databases, while the majority were putatively identified primarily as FAs (Figure 3E). There were both similarities and variations in donor responses to aspirin. To illustrate this, we focused on the large group of fully validated FA elevated in thrombin-activated cells. Accurate mass data extracted from Orbitrap Fourier transform mass spectrometry (FTMS) at 60,000 resolution were manually extracted and integrated. Heatmaps show the diversity of responses with lipids color-coded depending on class (Figure 4). As examples of divergent responses, donor 1 shows elevations by aspirin alone of several unsaturated FAs (AA, DHA, EPA, adrenic, DPA, and DTrA isomers) that is greater than the thrombin response. In contrast, donor 2 shows low level increases in a far smaller number of largely different FAs, including some that are saturated (arachidic, palmitic, NA, palmitoleic, and EPA). Last, donor 3 shows no elevation of any FAs by aspirin. This stimulation of FA release by aspirin in two donors occurs in the absence of COX activation and aggregation, and the mechanisms involved deserve further study.

In terms of oxidized FA, COX products tend to cluster with lipids that show the greatest inhibition by aspirin, while LOX products tend to show medium to low aspirin suppression (Figure 4). Monohydroxy fatty acids and other oxidized lipids are evenly spread, indicating they may originate from either pathway.

These data are also shown using a network analysis (Cytoscape 3.2.1). Nodes represent individual lipids, where degree (size) is determined by the number of links to others. Edge thickness represents the strength of correlation between individual nodes. Nodes with the highest degree clustered toward the center. These network diagrams illustrate that while there are clear donor variations, FA fluxes are significantly interconnected, with a major part of the pool changing in tandem. Related lipids cluster together, e.g., pink COX products always cluster toward the top left, while navy blue LOX products are further right. Unoxidized fatty acids link together but tended to show divergent responses to oxidized ones (Figure 4). Raw data on integration of all these lipids are presented in Data S6 to enable comparison of relative abundance of individual lipid species.

We also characterized the effect of aspirin on validated oxPLs, screening the 70 that were identified by the initial Sieve analysis, and found that the majority were unchanged or upregulated during aspirin treatment (comparing the thrombin with thrombin + aspirin datasets) (Data S1, tab 3). This agrees with the general view that aspirin selectively regulates platelet aggregation, but not coagulation.

### Effect of cPLA_2_ Blockade on FA and OxPL Generation

**Figure 5:**
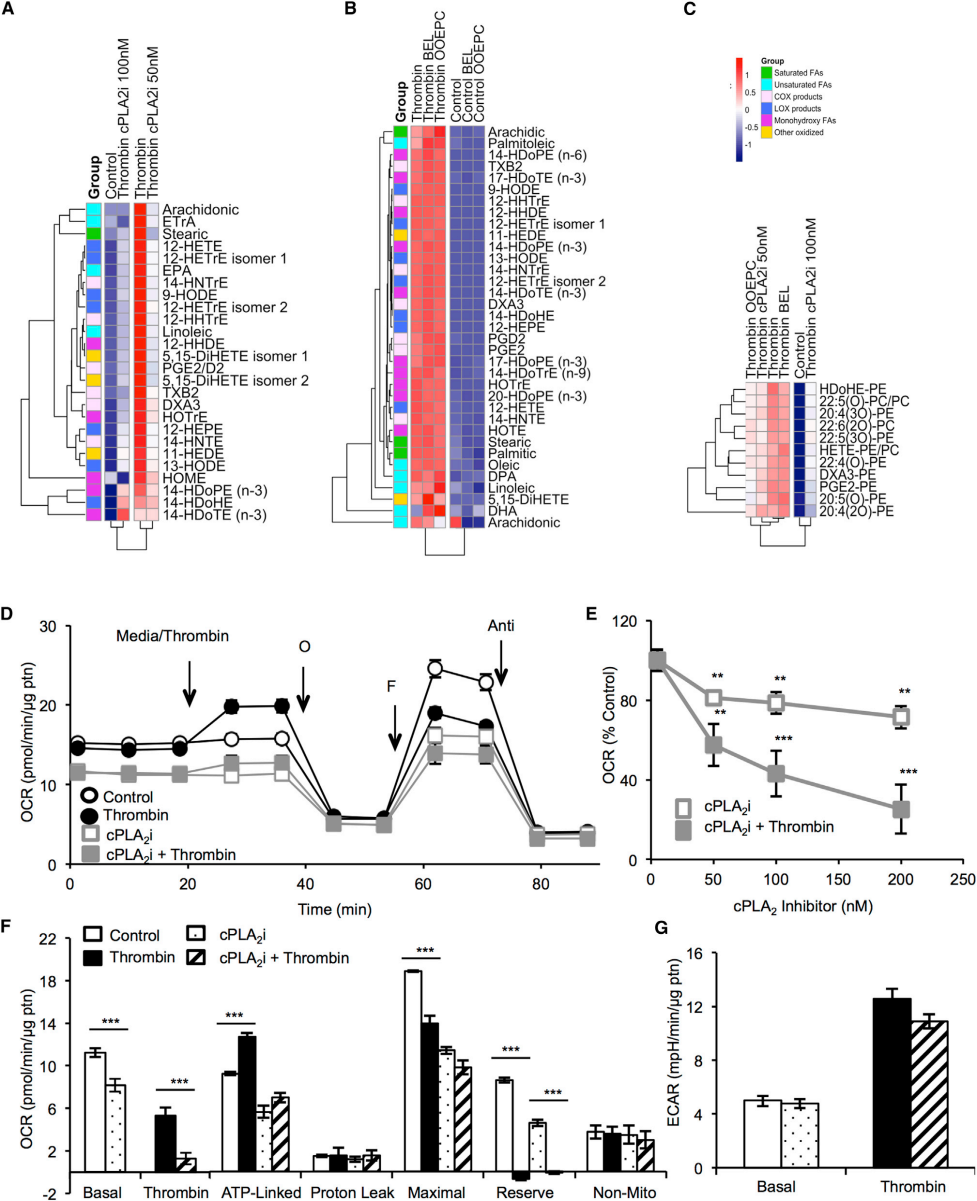
Thrombin-Stimulated Generation of FAs, Eicosanoids, OxPLs, and Mitochondrial Oxygen Consumption Is Sensitive to cPLA_2_ Inhibition, while sPLA_2_ Inhibition Suppresses OxPLs Only Heatmaps were generated using the pheatmap package in R and lipids color-coded according to lipid group. Levels of treatment response are represented by a color gradient ranging from blue to red (increase in response). Lipids are color-coded by group and clustered by similarity in overall response to the treatments (A–C) Effect of PLA_2_ inhibitors on generation of FAs and oxPLs by platelets. Washed platelets were incubated with inhibitors or vehicle for 15 min, then activated using 0.2 U/mL thrombin for 30 min and generation of lipids determined using LC/MS/MS as described in Supplemental Information. Inhibitors were as follows: (A) cPLA_2_, 50–100 nM cPLA_2_i; (B) iPLA_2_, 50 nM BEL; and sPLA_2_, 2 μM OOEPC. (C) Inhibition of oxPLs by PLA_2_ inhibitors. oxPLs are grouped depending on the *sn2* fatty acid to aid data visualization. Results are shown as heatmaps, with full data in Figures S3–S5. (D–G) cPLA_2_ inhibition suppresses mitochondrial oxygen consumption, but not glycolysis. Platelets were plated on Cell-Tak-coated XF96 plates, and pre-treated with cPLA_2_i (<200 nM) for 15 min prior to bioenergetic measurements. (D) Basal OCR of platelets was measured prior to injection of thrombin (0.5 U/mL), followed by 1 μg/mL oligomycin (O), 0.6 μM FCCP (F), and 10 μM antimycin A (Anti). (E) cPLA_2_i (0–200 nM) dose response of thrombin-linked OCR presented as a percentage of control. (F) Indices of mitochondrial function, basal, thrombin responsive, ATP-linked, proton leak, maximal, reserve capacity, and non-mitochondrial OCR were calculated. (G) Basal and thrombin-responsive ECARs were calculated from parallel ECAR measurements. Data expressed as mean ± SEM from one representative donor, n = 3–5 replicates per sample. ^∗^p < 0.05, ^∗∗^p < 0.01, and ^∗∗∗^p < 0.005 different from control (ANOVA and Bonferroni post hoc test).

Platelets express several isoforms of PLA_2_; thus, their role in regulating generation of FA and oxPL families was determined using inhibitors; for calcium-sensitive (cPLA_2_; cPLA_2_i), calcium-insensitive PLA_2_ (iPLA_2_; bromoenol lactone [BEL]), and secretory PLA_2_ (sPLA_2_; oleyloxyethylphosphocholine [OOEPC]). Overall, thrombin-stimulated generation of COX, LOX products, and FAs was effectively suppressed by cPLA_2_ inhibition but insensitive to iPLA_2_ or sPLA_2_ blockade (Figures 5A, 5B, S3, and S4). With cPLA_2_ blockade, free AA was inhibited to baseline levels (Figures 5A and S3B). However, platelets already contain free AA before activation, thus allowing up to 50% generation of COX or LOX products. OxPL generation was only partially sensitive to inhibition of cPLA_2_ but insensitive to iPLA_2_ blockade (Figures 5 C and S3D; individual isomers in Figure S5). Thus, sufficient eicosanoids are generated to allow formation oxPLs, even in the absence of AA release. Sensitivity of oxPLs, but not FAs, to sPLA_2_ inhibition was also seen (see Figure 5C for a summary and Figure S5 for all individual lipids). This suggests sPLA_2_ maybe the source of lyso-PL required for rapid eicosanoid esterification into PL pools.

Several saturated FAs were elevated in response to thrombin, although there was donor variability (Figure 4). These likely originate from the *sn1* position of PLs and may also be cleaved by the cPLA_2_α isoform, which is known to also have PLA_1_ activity (Loo et al., 1997). Platelets contain a large proportion of plasmalogen PLs and thus would not be considered a major source of hydrolysable saturated FAs. Biological actions and the cellular source of these lipids in the context of vascular health warrants further study.

### Blockade of cPLA_2_ Suppresses Basal- and Thrombin-Linked Mitochondrial Respiration, Linking Lipidomic Flux with Metabolism in Platelets

The diversity of FAs upregulated on platelet activation prompted further investigation. FAs are substrates for mitochondrial β-oxidation, which provides acetyl-coenzyme A for the citric acid cycle. We recently showed that oxygen consumption increases acutely on thrombin activation and is primarily supported by energy from β-oxidation (Ravi et al., 2015). Also, β-oxidation along with glycolysis is required for full aggregation to occur (Ravi et al., 2015). Thus, we queried if mobilization of FAs by cPLA_2_ upon thrombin activation might feed into acute regulation of mitochondrial respiration, linking lipidomic dynamics with acute changes in energy metabolism.

Accordingly, extracellular flux analysis showed that platelet basal oxygen consumption rate (OCR) decreased 20% with cPLA_2_i (Figure 5D). Thrombin then stimulated OCR, largely due to increased mitochondrial FA oxidation (Ravi et al., 2015). cPLA_2_i almost totally blocked thrombin stimulation of OCR compared to control (Figures 5D and 5E). To confirm the oxidative phosphorylation component, at 20–30 min, oligomycin was injected to inhibit complex V, leading to the expected decrease in OCR. Then, FCCP, a proton-ionophore, was injected to stimulate maximal respiration, leading to an increase in maximal OCR, but to a smaller extent in the cPLA_2_i-treated platelets (Figure 5D). Finally, antimycin A was added. ATP-linked respiration was calculated by subtracting OCR after oligomycin addition from basal. As shown recently, thrombin increased ATP-linked respiration, indicating an increased energetic requirement for aggregation (Figure 5F) (Ravi et al., 2015). Blockade of cPLA_2_ decreased this, both basally and in the presence of thrombin, and in the presence of cPLA_2_i, thrombin was unable to fully increase ATP-linked OCR (Figure 5F). This indicates that cPLA_2_ activity is required for ATP-linked oxygen consumption, both basally and on activation. Proton leak, calculated by subtracting OCR post-antimycin A from OCR post-oligomycin, was unchanged in all groups (Figure 5F). Reserve capacity was calculated by subtracting OCR before oligomycin from OCR after FCCP. As shown recently, thrombin decreases reserve capacity, since it is used to provide the mitochondrial function for the increased energy demand during aggregation, and we found this also occurred during cPLA_2_ inhibition, but to a lesser extent (Figure 5F) (Ravi et al., 2015). cPLA_2_ inhibition decreased reserve compared to basal (Figure 5F). Taken together, these data show that blockade of cPLA_2_ decreases mitochondrial electron transfer and lowers the cells’ ability to respond to increased energetic demands. It is fully consistent with a lower availability of energy-generating substrates in the absence of sufficient FAs provided by cPLA_2_.

Glycolysis was measured as the extracellular acidification rate (ECAR). We recently showed that ∼70% of the basal and thrombin-stimulated ECAR is inhibited by 2-deoxy-D-glucose (2DG), consistent with a high rate of glycolysis in platelets (Ravi et al., 2015). Both the basal and thrombin-stimulated ECARs were unchanged by cPLA_2_i (50 nM), indicating that they were not influenced by lipid metabolism (Figure 5G).

### Several Eicosanoids and FAs Act as Substrates for Mitochondrial β-Oxidation during Platelet Activation

**Figure 6:**
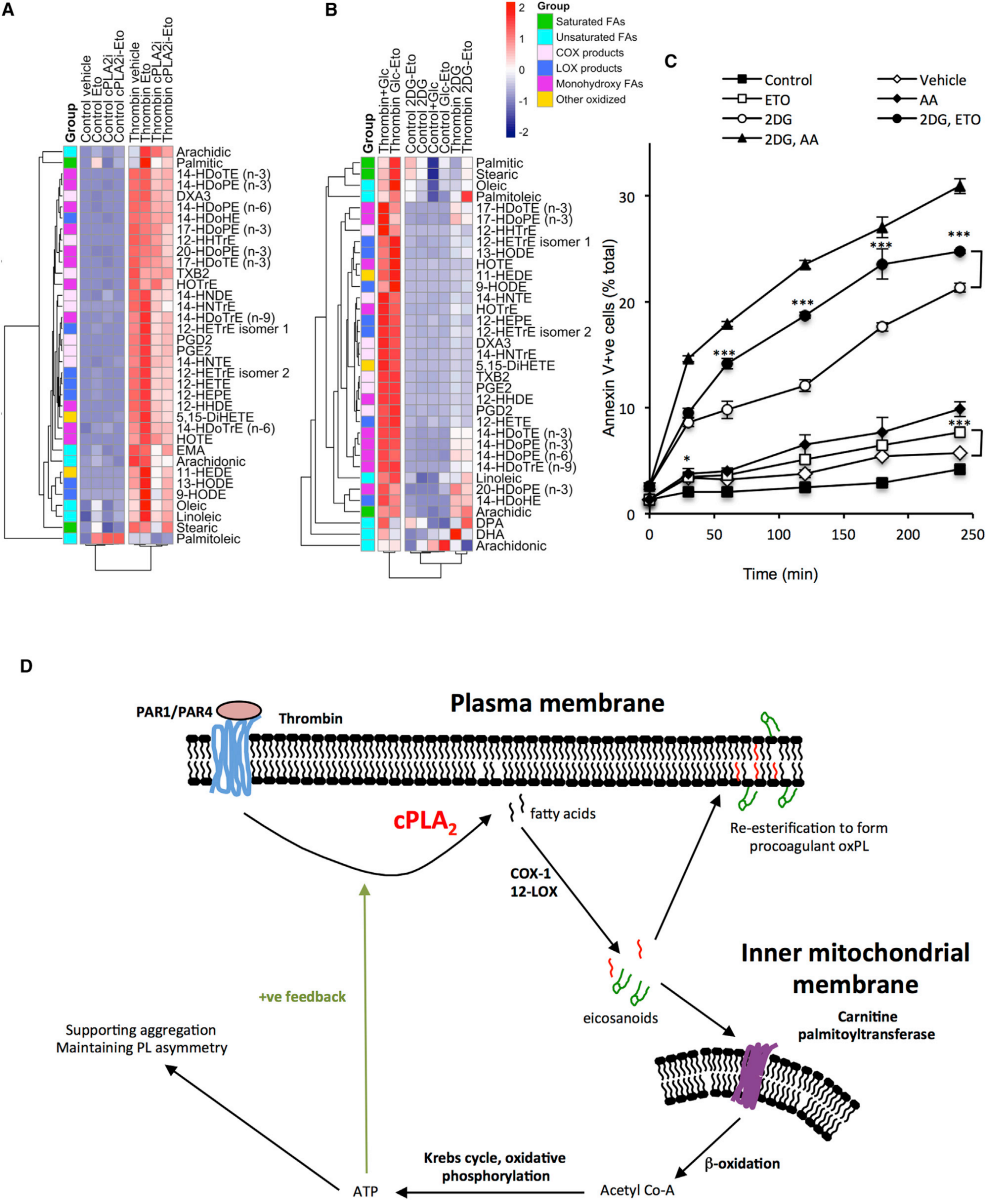
Eicosanoids and Fatty Acids Acutely Feed into β-Oxidation during Platelet Activation and Support Maintenance of Membrane Phospholipid Asymmetry Heatmaps were generated using the pheatmap package in R and lipids color-coded according to lipid group. Levels of treatment response are represented by a color gradient ranging from blue (decrease in response) to white (no change) to red (increase in response). Lipids are color-coded by group and clustered by similarity in overall response to the treatments. (A) Several eicosanoids are substrates for platelet β-oxidation on thrombin generation. Washed platelets were incubated with inhibitors or vehicle at room temperature (RT), then activated using 0.2 U/mL thrombin at 37°C for 30 min and generation of lipids determined using LC-MS/MS as described in Supplemental Information (n = 3). Inhibitors were as follows: etomoxir (Eto), 25 μM; cPLA_2_i, 100 nM. (B) Inhibition of glycolysis leads to a failure to generate free fatty acids and eicosanoids on thrombin activation of platelets. Washed platelets were incubated with inhibitors or vehicle at RT, then activated using 0.2 U/mL thrombin at 37°C for 30 min, with generation of lipids determined using LC-MS/MS as described in Supplemental Information (n = 3). Cells were incubated in the presence or absence of 5 mM glucose (Glc). Inhibitors were as follows: Eto, 25 μM; 2-deoxy-D-glucose (2DG), 120 mM. (C) Blocking β-oxidation leads to a failure to maintain phospholipid asymmetry in platelets. Washed platelets were incubated with inhibitors or vehicle for up to 240 min at RT, then stained for PS using Annexin V-FITC, for 15 min at RT in the dark, and analyzed by flow cytometry. Inhibitors were as follows: Eto, 25 μM; antimycin A (Anti), 10 μM; 2DG, 120 mM. Data were analyzed using one-way ANOVA with Bonferroni post hoc test (n = 3, mean ± SEM), comparing Eto with vehicle and Eto+2DG with 2DG. (D) Scheme for proposed role of cPLA_2_ in acutely feeding substrates into mitochondrial oxidative phosphorylation and generation of oxPLs.

We sought to determine flux of eicosanoids and FAs through β-oxidation in platelets using the carnitine palmitoyltransferase inhibitor etomoxir, which prevents transfer of FA across the inner mitochondrial membrane. Data are summarized in heatmaps with bar charts as Supplemental Information (Figures 6A and S6). In the resting platelet, increases in saturated and unsaturated FAs upon etomoxir inhibition show that these are ongoing β-oxidation substrates. However, thrombin-stimulated platelets utilized these and many oxidized eicosanoids at higher rates, as shown by the stronger response to etomoxir. Last, etomoxir elevated eicosanoids less when cPLA_2_ was inhibited (Figures 6A and S6).

### Platelet β-Oxidation and Glycolysis Are Required for FA and Eicosanoid Elevations during Platelet Activation, Representing a Positive Feedback Cycle Where Energy Metabolism Is Required for Acute Lipidomic Flux

Next, we sought to determine eicosanoid and FA flux via β-oxidation when glycolysis was blocked (glucose-free media + 2DG). Here, the expected thrombin-stimulated eicosanoid burst was absent (Figures 6B and S7). Furthermore, etomoxir failed to elevate eicosanoids or FAs, indicating that instead of accelerated consumption, there was a failure to generate FAs and eicosanoids when energy production from glycolysis was inhibited.

### Blocking β-Oxidation Leads to Aminophospholipid Externalization on the Platelet Surface

Since β-oxidation was recently shown to be required for full aggregation, we instead determined whether it was required for an additional energy-requiring pathway in platelets: maintenance of phospholipid asymmetry (Ravi et al., 2015). In this, flippases and floppases use ATP to keep PC on the outer leaflet of the plasma membrane and aminophospholipids (aPLs), phosphatidylserine (PS), and PE on the inner leaflet. Asymmetry is required for preventing aPL externalization, an event that occurs during platelet aggregation, apoptosis, and aging (storage lesion), supporting thrombosis and platelet clearance. This is measured using Annexin V, which binds to the aPL headgroups. Over time, etomoxir alone led to a small, but not significant, increase in aPL externalization, where glycolysis was able to compensate for blockade of β-oxidation (Figure 6C). In contrast, when glycolysis was blocked (glucose free medium + 2DG), large elevations in aPL externalization were noted. These were significantly enhanced by etomoxir, suggesting that β-oxidation is upregulated to compensate for the lack of glycolysis. Collectively, these data establish a functional link between β-oxidation/cPLA_2_ and mitochondrial metabolism that further extends our knowledge of how acute innate immune activation links to essential energy-requiring processes in platelets.

## Discussion

Currently, lipidomic approaches that simultaneously profile all lipids in the cellular or tissue lipidome, while ensuring that interfering artifact signals (e.g., isotopes, adducts, contaminating ions, and split-peak ions) have been thoroughly and robustly removed, are not widely available. This has made estimating the total lipidome in any mammalian cell extremely difficult. Herein, we generated and applied new informatics tools to address this gap. We estimated the total number and diversity of lipids in platelets and structurally identified many molecular species. An open resource is presented, allowing further mining of the lipidome for potential transducers of platelet-dependent activities, and it is described in more detail below.

We recently showed that β-oxidation contributes to the extra platelet energy demands that occur on activation (Ravi et al., 2015). Herein, we demonstrate using etomoxir that the substrates required for this are exclusively provided by cPLA_2_ (Figures 5D–5F). This links platelet lipid mobilization to acute mitochondrial bioenergetics and reveals a function for mitochondrial fatty oxidation in modulating the cellular lipidome (Figure 6D). A dynamic cycle is revealed where, via cPLA_2_, platelets dramatically upregulate both FA release and oxidation as well as their removal via β-oxidation on thrombin activation (Figure 6D). Several eicosanoids and FAs contributed to energy generation (Figures 6A and 6B). We note some apparent donor variability in terms of which FA and eicosanoids were utilized (Figures 6A and 6B). This may relate to differences in substrate supply for carnitine palmitoylacyltransferase between donors. Unexpectedly, when cPLA_2_ was inactivated, fewer eicosanoids were subsequently removed through β-oxidation (Figure S6, top). Thus, the ability of this pathway to consume eicosanoids is highly sensitive to their generation rates and thus, most likely, their availability as substrates.

When energy generation was blocked (via both glycolysis and β-oxidation inhibition), levels of eicosanoids fell dramatically (Figures 6B and S7). However, this was due to inhibition of their generation and not their increased consumption. This reveals a positive feedback loop where eicosanoid generation itself requires energy, as well as a dynamic mechanism for removal of eicosanoids that may also function to downregulate their signaling actions. This unexpected finding may be due to the requirement for ATP as a kinase substrate, e.g., for *src* tyrosine kinases and mitogen-activated protein kinase that are activated in response to thrombin upstream of cPLA_2_. Further studies are required in order to delineate this. Conducting a global lipidomic screen would determine whether this observation relates to lipidomic flux in general or is restricted to the effects on cPLA_2_ seen herein. To our knowledge, energetic communication or coupling between mitochondrial respiration and eicosanoid generation has not been reported for any cell type. Of relevance, we note that mitochondrial respiration and phosphorylation have been observed to be functionally coupled for nuclear transport (Dzeja et al., 2002).

To expand the functional relevance, the role of eicosanoids in supporting energy-dependent maintenance of phospholipid asymmetry in platelets via β-oxidation was characterized. Blocking β-oxidation inhibited asymmetry maintenance, particularly when combined with glycolysis inhibition. This form of anergy could be important if FA supply becomes limiting, e.g., at later time points following activation of cells during inflammation/injury, and may contribute to shutting down cell activities, promoting coagulation and clearance of dying and apoptotic platelets. We note that levels of lipids presented herein represent a steady state, impacted by both generation and removal rates, and thus do not provide information on total flux through the pathway. Thus, the actual amounts mobilized and ultimately used as substrate for energy generation are not yet known. It is possible that a reliance on phospholipids to meet energy requirements could deplete the cell of essential structural building blocks. However, phospholipids are the predominant platelet lipid class, and these cells are well known to undergo significant remodeling of their membranes during activation. Furthermore, once trapped in a clot, they are considered to have mediated their primary function and are ultimately removed by macrophage efferocytosis.

Our characterization is a much closer approximation compared to previous studies of the total platelet lipidome (herein, defining lipids as hydrophobic molecules extracted using standard lipidomic extraction protocols), in terms of basal size and diversity, and represents a best estimate with the following limitations: (1) instrument sensitivity may prevent all lipids being detected; (2) despite major manual efforts, insufficient cleanup of datasets may lead to some overestimation; (3) some lipid classes are difficult to extract and analyze using standard methods and maybe under-represented (e.g., some phosphoinositides, oxysterols, and sphingolipids); and (4) the dataset may include some unidentified species not classed as lipids (e.g., hydrophobic peptides). However, even with these limitations, we believe our strategy offers a major step forward in defining the total lipidome of a mammalian cell and demonstrates several findings about both diversity and dynamic changes in lipids during cell activation and inhibition. Many lipids from the most abundant classes were found, as well as thrombin-upregulated lipids in the low-nanogram range (PGE_2_, PGD_2_, and esterified eicosanoids), validating both the coverage and sensitivity of the approach. While several automated and manual steps to remove artifact ions were included, further optimization is still required. For example, we are currently implementing a machine-learning approach to parameter optimization that will further enhance data quality, and consolidating LC analysis into a single separation.

Given the size of lipidomics datasets, it is not feasible to structurally validate every ion. Thus, we selected a smaller number of ions for detailed structural characterization. We fully identified and established triple-quadrupole-based assays for 111 oxPLs and 81 FAs generated on thrombin activation, including several that have never been detected before in platelets or other mammalian cells. While a small number of oxPLs have been found previously, we show that membrane PL oxidation is a far more diverse and wide-ranging process in platelets than previously considered (Thomas et al., 2010). The most abundant oxPLs containing HETEs are pro-coagulant both in vitro and in vivo, through altering membrane charge and facilitating coagulation factor binding at the cell surface (Thomas et al., 2010; unpublished data). Thus, global oxidation of the unsaturated platelet membrane PL pool is considerable, may support hemostasis, and is analogous to the lipid whisker model proposed by Hazen and colleagues whereby activated macrophages become coated in oxPLs and visible to lipid scavenger receptors (Greenberg et al., 2008).

Three genetically unrelated donors showed considerable diversity in their basal and thrombin-stimulated lipidomes and responses to aspirin (Figures 1 and 4). Furthermore, one showed far higher activation by thrombin in terms of the number or amount of lipids generated (donor 3), while another showed far less inhibition of the thrombin-stimulated lipid pool by aspirin (donor 1), despite full blockade of the primary COX-1-derived eicosanoids TX and 12-HHTrE. Low-dose aspirin is considered platelet COX-1 selective, through acetylation of the active site. However, many aspirin-inhibitable lipids, including FAs, are generated via phospholipases upstream of COX-1. This underscores the importance of secondary activation as a positive feedback mechanism during platelet-aggregation-associated lipid mobilization. Specifically, COX-1-generated TXA_2_ activates the thromboxane receptor (TP), inducing release of secondary mediators such as ADP that then potentiate the thrombin-mediated global lipidomic changes observed. We also noted aspirin upregulation of FAs that was highly donor specific (Figure 4). How lipid variations are controlled at a genetic and environmental level is not understood and will require further study on the lipidomes of large numbers of people and in different cells or tissues. In particular, given the importance of aspirin as both a cardioprotective and possible cancer therapeutic, a full understanding of how it regulates platelet lipids will be the focus of a follow-up study with a larger number of volunteers. The stability of the global lipidome with age, diet, and over time is unknown, and the influence of external factors such as epigenetic control of lipid-metabolizing enzymes could be considerable. Of relevance, it was recently shown that cellular immune systems are highly variable between individuals but remain remarkably constant over time (Carr et al., 2016). Environmental factors were a key influence, and during infection, transient changes were observed that quickly reverted to baseline. Our understanding of intra and inter-individual variability in terms human cell biology of the immune and blood systems is in its infancy but currently a major topic of research interest. Our study represents a first look at a small group but importantly provides a platform for future longitudinal studies of platelet lipidomics.

The platelet lipidome contains large numbers of species that are absent from current databases (Figures S1A and S1C). These included structures characterized herein, such 14-HDTE, HDTrE, and HNDE, and many oxPLs. Most of the unknowns are above 1 kDa, and could include some classes absent from databases, such as glycolipids (Figure 1D). Developing informatics workflows to assist with identification of specific families from these large numbers of unknowns will be a major future endeavor. The presence of 14-hydroxy C19 lipids was intriguing, since platelets contain no detectable C19 unsaturated substrate (Figure 3). They were largely inhibited by aspirin, suggesting they originate via COX-1 (Data S1). Platelets generate significant amounts of the C17 12-hydroxyheptadecatrienoic acid (12-HHTrE) due to degradation of thromboxane A2 (TXA_2_), where part of the prostanoid ring is lost (malondialdehyde), generating the C17 monohydroxy lipid. We reasoned that platelets might generate analogous lipids through metabolizing C22 substrates with three, four, or five double bonds. Indeed, previous studies found exogenous C22 substrate can be metabolized by COX (Milks and Sprecher, 1985, Sprecher, 1986, Sprecher and Careaga, 1986); however, their generation from endogenous substrate on agonist activation has never been shown. Analogous lipids from 22:6 were not detected, and ions giving MS/MS fragmentation spectra consistent with resolvins, lipoxins (LXs), or protectins were absent from our dataset. While some lipids with parent *m/z* 351.2180 suggestive of lipoxins were detected (Data S5 [p28] and Data S6 [p 15, top three bars]), their MS/MS spectra were distinct from either LXA_2_ or B_2_. These were highly sensitive to aspirin inhibition, indicating they likely originated via COX-1. We propose these to be additional prostanoids based on the presence of some common daughter ions, but at this stage, their structures are unknown. Thus, pro-resolving mediators, including aspirin-triggered lipoxins, were not generated by acutely activated platelets.

In summary, we surveyed the global diversity of human lipids in a single cell type, including both knowns and unknowns and to a high degree of sensitivity. We then used the information to discover many lipids and link acute lipidomic changes with metabolism. Specifically, cPLA_2_ blockade acutely decreased β-oxidation and mitochondrial respiration, indicating a link between inflammatory signaling and regulation of the cellular metabolome. These data represent the first full estimate of total lipid molecular species for any cell type to date.

## Experimental Procedures

### Chemicals

These are provided in Supplemental Information.

### Human Platelet Isolation, Activation, and Inhibition

For lipidomic studies, platelets were isolated from three genetically unrelated healthy volunteers, as described in Supplemental Information. For baseline samples, donors were free from nonsteroidal anti-inflammatory drugs for at least 14 days and the study was approved by the Cardiff University School of Medicine Ethics Committee (SMREC 12/13). For aspirinized samples, the same donors were administered 75 mg/day aspirin for 7 days before donation. For mitochondria experiments, platelets were isolated from concentrates obtained from the blood bank by centrifugation then washed 2× with PBS and counted as described previously (Chacko et al., 2013). Collection and use of these samples was approved by University of Alabama at Birmingham Institutional Review Board.

### Lipid Extraction

Lipids were extracted as described in Supplemental Information, resuspended in 200 μL methanol and stored at −80°C until analysis. For time course and MRM experiments, internal standards (at 10 ng each of PGE2-*d4*, 12-HETE-*d8*, DMPE, and DMPC) were added to the extraction solvent of time course activation.

### Global Lipidomics and Analysis of Lipids

Untargeted global analysis of lipids was performed, separately in positive- or negative-ion mode, using LC coupled to an Orbitrap Elite mass spectrometer (UPLC-Orbitrap Elite MS; Thermo Fisher Scientific). Lipids extracts were separated on two different reverse-phase (RP) columns and gradient methods, as described in Supplemental Information.

### Targeted Analysis of Lipids

Selected lipids upregulated on thrombin activation in human platelets were analyzed for temporal dynamics of their generation using LC-MS/MS (ABI 4000 or 6500 QTRAP) by monitoring precursor-to-product ion transitions in MRM mode, as described in Supplemental Information.

### Data Processing

Global scanning data, chromatographic alignment, peak or feature detection, and isotopic filtering used SIEVE 2.0 (Thermo Fisher Scientific), as described in Supplemental Information. SIEVE-aligned data were further processed using in-house-generated software as described in Supplemental Information.

### Statistical Analysis and Data Visualization

Treatment effect on donor grouping was projected using PCA from mixOmics package in R. This dataset (features/mass ions common in two or more donors) was scaled to unit variance (UV). Global distribution of features and mass ions among donors was shown using VennDiagram in R (vennDiagram). Radar plots were generated using Excel. Heatmaps were generated for each donor using the pheatmap package in R, and lipids were color-coded according to lipid group. Network analysis was done using Cytoscape 3.2.1. For each donor, pairwise correlations between lipids were calculated in R. Due to the high number of interactions, the network diagram shows only positive correlations with a Pearson product-moment correlation coefficient value (*r*) > 0.9.

### Structural Interpretation and Assignment of Lipids

Lipid species were putatively identified by matching their accurate mass to records in the following databases: (1) the HMDB (Wishart et al., 2013; http://www.hmdb.ca/), (2) Lipidhome (Foster et al., 2013; http://www.ebi.ac.uk/apweiler-srv/lipidhome/), (3) LipidMaps (Lipid Metabolites and Pathways Strategy) (Sud et al., 2007; http://www.lipidmaps.org/), and (4) METLIN (metabolite and tandem MS database) (Smith et al., 2005; http://metlin.scripps.edu/) (as described in Supplemental Information).

### Seahorse Extracellular Flux Analysis

Bioenergetic measurements of platelets were performed as previously described (Chacko et al., 2013, Ravi et al., 2015). In brief, platelets suspended in XF DMEM assay buffer (DMEM with 1 mM pyruvate, 5.5 mM D-glucose, and 4 mM L-glutamine [pH 7.4]), were plated on Cell-Tak coated platelets and then pre-incubated with cPLA_2_i (0–200 nM) for 15 min prior to the assay. First basal bioenergetics were measured, followed by injection of either thrombin (0.5 U/mL) or media, oligomycin (1 μg/mL), FCCP (0.6 μM), and antimycin A (10 μM).

## Author Contributions

D.A.S., C.J.B., M.A., S.M., S.R., G.A.B., and V.B.O. conducted experiments. D.A.S., V.B.O., V.D.U., and S.A. designed experiments. A.O.C. and C.J.B. wrote software, conducted analysis, and generated figures. R.C.M. and V.D.U. analyzed data. V.B.O., D.A.S., and A.O.C. drafted the manuscript. All authors edited the manuscript.

## Supplemental Information

Document S1. Supplemental Experimental Procedures, Figures S1–S7, Tables S1–S5, and Instructions for Viewing GoogleVis Files in a Browser

Data S1. Full List of Ions Detected in Basal- and Thrombin-Activated Platelets, Related to Figure 1

Data S2. GoogleVis Interactive Diagrams, Related to Figure 1G

Data S3. GoogleVis Interactive Diagrams, Related to Figure 1H

Data S4. Full MS/MS Information on oxPL, Related to Table S2

Data S5. Full MS/MS Information on All FA, Related to Table S4

Data S6. Response of All FA to Thrombin and Aspirin, Related to Figure 3D

Document S2. Article plus Supplemental Information
