# Supplementary material for: Mapping the Human Platelet Lipidome Reveals Cytosolic Phospholipase A2 as a Regulator of Mitochondrial Bioenergetics during Activation
Source: Cell Metab. 2016 May 10;23(5):930–44. doi: 10.1016/j.cmet.2016.04.001 (PMC4873619; doi:10.1016/j.cmet.2016.04.001)
Supplement: Document S1. Supplemental Experimental Procedures, Figures S1–S7, Tables S1–S5, and Instructions for Viewing GoogleVis Files in a Browser [file mmc1.pdf]

Cell Metabolism, Volume *23*

Supplemental Information

Mapping the Human Platelet Lipidome Reveals Cytosolic Phospholipase A_2 as a Regulator of Mitochondrial Bioenergetics during Activation

David A. Slatter, Maceler Aldrovandi, Anne O'Connor, Stuart M. Allen, Christopher J. Brasher, Robert C. Murphy, Sven Mecklemann, Saranya Ravi, Victor Darley-Usmar, and Valerie B. O'Donnell

Supplementary Methods, Tables and Figures.

Supplementary Methods

Chemicals Lipid standards, 12(S)-hydroxyeicosatetraenoic acid (12s-HETE), 12-HETE-d_8, prostaglandin E_2-d_4 (PGE_2-d_4), 1-stearoyl-2-arachidonoyl-*sn*-glycero-3-phosphatidylethanolamine (18:0a/20:4-PE), 1,2-Dimyristoyl-*sn*-glycero-3-phosphatidylcholine (DMPC), 1,2-Dimyristoyl-*sn*-glycero-3-phosphatidylethanolamine (DMPE) were purchased from Avanti Polar Lipids or Cayman Chemical. Phospholipase inhibitors were from Calbiochem. HPLC grade solvents and acids were purchased from Fisher Scientific UK Ltd. All other reagents were obtained from Sigma-Aldrich unless otherwise stated.

Lipid extraction Lipids were extracted by adding a solvent mixture (1 M acetic acid/propan-2-ol/hexane; 2:20:30, v/v) to platelets at a ratio of 2.5 ml of solvent mixture/ml platelets in 10 ml extraction vial and vortexed for 30 seconds. 2.5 ml of hexane was added and then vortexed and centrifuged (500 *g* for 5 minutes at 4 °C) to recover lipids in the upper hexane layer. Aqueous samples were re-extracted by addition of 2.5 ml hexane. The combined hexane layers were dried in a RapidVap (Labconco) at room temperature.

Human platelet isolation and activation/inhibition. For baseline samples, donors were free from nonsteroidal anti-inflammatory drugs for at least 14 days and the study was approved by the Cardiff University School of Medicine Ethics Committee (SMREC 12/13). For aspirinized samples, donors were administered 75 mg/day aspirin for 7 days before donation. Briefly, blood was collected into ACD (Acid-Citrate-Dextrose; 85 mM trisodium citrate, 65 mM citric acid and 100 mM glucose) at a blood/ACD ratio of 8.1:1.9 (v/v) and centrifuged at 250 *g* for 10 minutes at room temperature (22 °C). Platelet-rich plasma was collected and centrifuged at 900 *g* for 10 minutes, and the pellet resuspended in Tyrode's buffer (134 mM NaCl, 12 mM $NaHCO_3$, 2.9 mM

KCl, 0.34 mM Na_2HPO_4, 1.0 mM $MgCl_2$, 10 mM HEPES and 5 mM glucose, pH 7.4) containing ACD (9:1, v/v). The platelets were washed by centrifuging at 800 *g* for 10 minutes then re-suspended in Tyrode's buffer at a concentration of 2 × 10^8 cells/ml. Platelets were pre-incubated with 1 mM $CaCl_2$ at 37 °C for 5 minutes and then activated by thrombin (0.2 units/ml) at 37 °C for up to 30 min. Where used, inhibitors were pre-incubated for 10 min before thrombin activation, with appropriate vehicle controls used.

Global lipidomics/analysis of lipids Two methods were used: the first (for lipophilic species, termed "Non-Polar") used a Hypersil GOLD C_{18} RP UPLC column (150 x 2.1 mm I.D., 1.9 µm particle size) with gradient of mobile phase A (acetonitrile:water, 50:50 v/v, 1 mM ammonium acetate, 0.1 % glacial acetic acid) and B (iso-propanol:acetonitrile, 70:30 v/v, 1 mM ammonium acetate, 0.1 % glacial acetic acid) at 0.4 ml/min over 55 min. The elution gradient of B (%) over time was: 35 to 50 % for 10 min, 50 to 66 % for 6 min, 66 to 76 % for 22 min, 76 to 96 % for 10 min, held at 96 % for 4.5 min then equilibrated at 35 % for 2.5 min. The second (for non-lipophilic lipids, termed "Polar") used a Spherisorb ODS2 column (150 x 2.1 mm, 3 µm particle size) with solvent gradient of mobile phase A (water:acetonitrile, 75:25, v/v, 1mM ammonium acetate and 0.1 % glacial acetic acid) and B (methanol:acetonitrile, 60:40, v/v, 1mM ammonium acetate and 0.1 % glacial acetic acid) at 0.4 ml/min over 30 min. The elution gradient of B (%) over time was: 50 to 90 % for 20 min, held at 90 % for 5.1 min and equilibrated at 50 % for 4.9 min. Samples were maintained at 4 °C and the column at 25 °C. The MS conditions were as follows: HESI-II temperature 350 °C, N_2 as drying gas, sheath gas flow 52 arbitrary units, auxiliary gas flow 17 units, capillary temp 320 °C, spray voltage +/- 3.5 kV and S-lens RF level 69.8/65.60 % respectively for positive and negative ion mode. High resolution (60,000 at 400 amu) full-scan MS spectra were acquired over 100 to 900 *m/z* or 900 to 1800 *m/z* (for both lipid separation and ion polarity methods) in centroid mode. Samples were analyzed at random, with every fifth or sixth injection of solvent blank. A one-off column conditioning at the beginning was

done with two runs of solvent blank followed by five runs of unrelated platelet extract and then two runs of solvent blank using same solvent gradient.

Developing a method to analyze the complete platelet lipidome.

To determine the complete lipidome, features from several available lipidomics methods were combined to achieve broad coverage with high sensitivity across the full range of lipids. High resolution scanning was used to maximize detection of lipids with close *m/z* values and extensive chromatography to separate isobaric species and enable detection of low abundance lipids. Two reverse phase chromatographic methods were optimized, one for relatively lipophilic (e.g. phospholipids (PL), triglycerides, neutral lipids, called "Non-Polar") and the other for non-lipophilic species (e.g. eicosanoids, fatty acids (FA), called "Polar"). Chromatographic separation provides retention time information, important given the numerous isobaric species not distinguished using shotgun approaches. This applies not only to phospholipids/glycerides, but also for less abundant prostaglandins/eicosanoids, which can co-elute during shorter analyses. Lipids were extracted using a standard method that broadly extracts lipids from most classes (Zhang et al., 2002).

MS detection used an Orbitrap Elite in full-scan FTMS mode, at 60,000 resolution to enable sufficient scans (approx. 3 per second) to be acquired for preliminary identification of lipids, without compromising mass accuracy. Each sample was analyzed using 8 separate chromatographic runs: (i) to resolve lipophilic vs non-lipophilic lipids, (ii) in positive or negative mode, and (iii) at two mass ranges (100-900 and 900-1800 *m/z*). This enabled acquisition of enough high resolution scans to allow detection of lipids with similar mass but identical retention time to be distinguished solely based on *m/z*. Due to the comprehensive analyses, significant post acquisition processing of large data files was required (described below). For each donor, we analyzed 3-4 technical replicates (448 runs) in random order, using a blank solvent injection every 5th or 6th sample. Data files were processed using SIEVE 2.0 (ThermoFisher Scientific), for peak alignment, isotope removal, and data extraction, as described below. This allows all

features (ions) to be identified and compared between samples, based on accurate mass and retention time. Next, SIEVE-processed data was refined using an in-house custom-generated workflow that: removed noise peaks, combined features making up the same ion peak into one feature, identified and removed lipid duplicates detected by more than one run (e.g. that generate both positive and negative ions, generate adducts, or are retained by both columns). Adducts and artifact ions removed are listed in Table S1. A large number of peaks were checked manually to verify correct processing. The wokflow was initially implemented in Excel. To speed up processing and improve automation, it is currently being refactored into Python and will be available as a stand alone software package in 2016. The approach detected several well-known, but quantitatively minor, platelet lipids thus validating its sensitivity (shown later). We present GoogleVis interactive scatter diagrams for data mining and detailed visualization as Supplementary Data. These allow in-depth analysis of sub-sections of the lipidome based on *m/z,* lipid class and retention time, and are available with a short manual in Supplementary Data. We also include spreadsheets of *m/z* values and putative identifications.

For studies on aspirin inhibition, we compared the *thrombin* with *thrombin+aspirin* datasets for lipids that were suppressed at least 2-fold in 2 or more donors. All 753 thrombin-upregulated ions were manually verified to ensure data accuracy.

Data processing SIEVE 2.0 (ThermoFisher) parameters for chromatographic alignment and framing (feature identifier) were optimized according to the column type. SIEVE first aligns data from multiple runs, removing isotopes. It finds the highest intensity mass/time peak in all the aligned runs. All the counts within a specified time (“retention time width” or RT width) and mass tolerance (5 ppm) of that mass/time maxima are summed for each run in the analysis, and are extracted that as a “frame”. SIEVE then iteratively extracts additional frames until the highest remaining intensity peak is below a specified count value, or a specified frame maximum is reached. Because of this methodology, SIEVE does not attempt to group frames into peaks, or distinguish frames representing artifacts or solvent as opposed to lipids.

For non-lipophilic lipids separated on the ODS2 RP column (Polar), RT width was 0.4 min over 1.0 to 28.0 min and intensity threshold of 1000 counts. For lipophilic lipids separated on the HypersilGold RP column (Non-Polar), RT width was 0.8 min over 1.0 to 50.0 min and intensity threshold 1000 counts.

SIEVE aligned data was further processed using our in-house generated Excel-based workflow to eliminate solvent-like features, based-on compliance of at least three parameters from retention time-sorted accurate mass groups (mass tolerance 5 ppm), (1) mass ion/feature is continuously present over 1.2 min, (2) variance of mean peak intensity over this time is relatively low (SEM < 20 %), (3) more than 6 features with same accurate mass and (4) standard deviation of the elution times from all elements in mass group is more than 1 min. Next, peak finding was undertaken, based on local feature/frame maxima and concatenation of features separated due to occasional chromatographic shifts. Only single frames with highest peak intensity on either side of local maxima and within half-time of RT framing were added to the frame with local maxima. Next, adduct ions of low abundance within positive and negative mode data sets were removed. Elimination was only undertaken where adduct ions eluted at the same time as the molecular ion. Adduct species were removed from positive and negative ion data sets, along with additional contaminating *m/z* ions forming either a series of resolved or stacked multiple adduct species (see Table S1 for full details). Next, RT-matched redundant or duplicate features detected between positive and negative ion mode from same column type data set with low average peak intensity were eliminated. Early eluting lipid species/mass ions on the Hypersil Gold column were retained on the ODS2 column. These early eluting duplicate mass ions were eliminated from the Hypersil Gold column by matching progressive RT shift. The total number of unique features in resting and thrombin-treated platelets within all donors was estimated as follows. Features/mass ions above a fixed intensity threshold (200 counts for polar and 500 counts for non-polar) with relative standard deviation (RSD) below 35 % from resting and thrombin-treated data sets were retained. Differentially changed features/mass ions upon thrombin-activation from all three donors were identified by applying a 2-fold threshold.

Upregulated features/mass ions common to at least two out of three donors and with highest average peak intensity were used for database searches.

Structural interpretation and assignment of lipids

Lipid species were putatively identified by matching their accurate mass to records in the following databases: (1) the Human metabolome database (HMDB) (Wishart et al., 2013): http://www.hmdb.ca/, (2) Lipidhome (Foster et al., 2013): http://www.ebi.ac.uk/metabolights/lipidhome/, (3) LipidMaps (Lipid metabolites and pathways strategy) (Sud et al., 2007): http://www.lipidmaps.org/, *(4)* METLIN (metabolite and tandem MS database) (Smith et al., 2005): http://metlin.scripps.edu/ as described in Supplementary Data. To automate the identification process a python program was developed to search 3 of these databases (HMDB, Lipidhome and LipidMaps). METLIN was searched manually in batch mode (maximum 500 masses in one batch). A query was generated against each database using mass value, ion mode (plus adduct types) and MW tolerance (±0.005 Da). The only exception was LipidMaps, which does not allow for adduct ion selection. Thus, for LipidMaps, masses were converted to neutral (from positive or negative ions) prior to query. Then, resulting masses were converted back to positive or negative mode after the search. Output files were saved in Microsoft Excel .csv format. Additionally, a .csv file was produced for each database listing the mass values for which no match was found. Each record includes mass error, retention time and polarity. Output files were merged, during which entries were standardized. Entries were removed where the mass error value was ≥ ± 5 ppm. In addition, only entries containing adducts listed in Table S2 were retained, all other entries were removed. Lastly, duplicate records with the same mass and identification, but originating from a different database were removed. For this, the record with the lowest mass error value was retained.

Structural analysis of lipids. Lipids of interest were fragmented for structural analysis in data dependent acquisition (DDA) mode, where sequential MS^2 or MS^3 scans were triggered

following identification of precursor mass in the first full scan event. Target mass ion (precursor or product ion) isolation and fragmentation was done in the high pressure cell of Velos Pro and accurate mass measurements on resulting product ions acquired in the Orbitrap at 30,000 resolution. Parameters for target mass ion isolation and fragmentation were: isolation width 1.4 Da, collision energy 35 to 50 %, activation Q of 0.25 and activation time 10 ms. Helium was used as collision gas.

Targeted analysis of lipids. For oxPL, lipid extracts (20 µL) were separated on a LUNA RP C_{18} column (150×2.1 mm, 3 µm particle size) using a binary solvent gradient of mobile phase A (methanol:acetonitrile:water 60:20:20, v/v/v, 1 mM ammonium acetate) and B (methanol 100 %, 1mM ammonium acetate) at a flow rate 0.2 ml/min over 50 min. The elution gradient of B (%) over time was: 50 to 100 % for 10 min, 100 % for 30 min, reduced to 50 % in 2 min and held at 50% for next 8 min. Specific precursor-to-product ion transitions are listed in Table S2. Optimized ESI-MS/MS conditions were: source temperature 500 ℃, GS1 40, GS2 30, curtain gas (CUR) 20, ion spray voltage (IS) -4500 V, first quadrupole (Q1) at low resolution, third quadrupole (Q3) at unit resolution, dwell time 150 msec, declustering potential (DP) -140 V, entrance potential (EP) -10 V, collision energy (CE) -45 V and collision cell exit potential (CXP) at -7 V. The parent to daughter MRM transitions used are listed in Table S3. Dimyristoyl-PE (DMPE) internal standard was added at 10 ng per sample. Lipids were normalized to internal standards to correct for extraction efficiencies. For fatty acids and eicosanoids, lipids were analyzed using a C18 Spherisorb ODS2, 5 μ, 150 x 4.6 mm column (Waters, Hertfordshire, UK). The mobile phase was gradient of 50–90% B over 10 min (A, water:acetonitrile:acetic acid, 75:25:0.1; B, methanol:acetonitrile:acetic acid, 60:40:0.1) with a flow rate of 1 ml/min. MS was performed using a Sciex 4000 Q-Trap, using DP -55 V, CE -26 V. Internal standards were added at 10 ng per sample as follows: PGE_2-*d4*, 12-HETE-*d8* and AA-*d8*. Lipids were normalized to internal standards to correct for extraction efficiencies. For oxidized FA, MRM

transitions were determined using the MS/MS spectra for each lipid, while unoxidized FA were analyzed by LC/MS, using the parent mass and scanning in Q1 mode.

Annexin V binding to platelets. Platelets were incubated with inhibitors or vehicle in the presence or absence of 5 mM glucose for 120 min. Each sample was analysed by flow cytometry for annexin V binding (Annexin V-FITC; BioLegend). 5 µl of annexin V was added to $2x10^6$ platelets in Tyrodes'buffer containing 3 mM calcium. Platelets were then incubated for 15 min at RT in the dark. Platelets incubated with 4 % paraformaldehyde for 30 min were used as positive control.

References

Foster, J.M., Moreno, P., Fabregat, A., Hermjakob, H., Steinbeck, C., Apweiler, R., Wakelam, M.J., and Vizcaino, J.A. (2013). LipidHome: a database of theoretical lipids optimized for high throughput mass spectrometry lipidomics. PloS one *8*, e61951.

Smith, C.A., O'Maille, G., Want, E.J., Qin, C., Trauger, S.A., Brandon, T.R., Custodio, D.E., Abagyan, R., and Siuzdak, G. (2005). METLIN: a metabolite mass spectral database. Therapeutic drug monitoring *27*, 747-751.

Sud, M., Fahy, E., Cotter, D., Brown, A., Dennis, E.A., Glass, C.K., Merrill, A.H., Jr., Murphy, R.C., Raetz, C.R., Russell, D.W.*, et al.* (2007). LMSD: LIPID MAPS structure database. Nucleic acids research *35*, D527-532.

Wishart, D.S., Jewison, T., Guo, A.C., Wilson, M., Knox, C., Liu, Y., Djoumbou, Y., Mandal, R., Aziat, F., Dong, E.*, et al.* (2013). HMDB 3.0--The Human Metabolome Database in 2013. Nucleic acids research *41*, D801-807.

Zhang, R., Brennan, M.L., Shen, Z., MacPherson, J.C., Schmitt, D., Molenda, C.E., and Hazen, S.L. (2002). Myeloperoxidase functions as a major enzymatic catalyst for initiation of lipid peroxidation at sites of inflammation. The Journal of biological chemistry *277*, 46116-46122.

**Adduct ion series elimination**			
Negative mode		Positive mode	
**Adduct**	**m/z (amu)**	**Adduct**	**m/z (amu)**
M+Na-2H	20.97357	$M+NH_4$	10.033823
M+Cl-	34.9694	M+Na	22.989218
M+AcO-	59.0133	M+K	38.963158
2M-H	-1.00783	$2M+NH_4$	18.033823
		2M+Na	22.989218

**Additional repeating units**	
**Name**	**m/z (amu)**
Quadruply charged ion series (from propanal)	14.5104
Triply charged ion series (from propanal)	19.347
Doubly charged ion series (from propanal)	29.021
PEG related components	44.0262
PPG related components, NaCl	58.0419
Dimethylsiloxane $Si(CH_3)_2O$ from rubber	74.0188
Sodium acetate NaAc	82.003

Table S1, related to Experimental Procedures. List of adducts and contaminating ions removed from the data set.

Parent	RT	ID	Parent	RT	ID	Parent	RT	ID
738.5079	18.9	16:0p/15-HETE-PE	778.503	17.55	18:2a/HETE-PE	798.5291	18.03	18:0a/20:4(2O)-PE
738.5079	19.56	16:0p/11-HETE-PE	778.503	17.56	18:1a/20:5(O)-PE	798.5291	18.31	18:0a/20:4(2O)-PE
738.5079	19.78	16:0p/12-HETE-PE	778.503	17.71	16:0a/HDoHE-PE	798.5291	18.36	18:0p/20:4(3O)-PE
738.5079	20.4	16:0p/8-HETE-PE	778.503	17.75	16:0p/22:6(2O)-PE	798.5291	19.25	18:0a/20:4(2O)-PE
738.5079	20.95	16:0p/HETE-PE	778.503	18.1	18:2a/HETE-PE	798.5291	20.27	18:0a/20:4(2O)-PE
754.5027	16.5	16:0p/AA(2O)-PE	778.503	18.7	16:0p/22:6(2O)-PE	798.5291	20.71	18:0a/20:4(2O)-PE
754.5027	17.22	16:0p/AA(2O)-PE	778.503	20.11	18:1a/20:5(O)-PE	798.5291	20.71	18:0a/20:4(2O)-PE
754.5027	17.84	16:0a/HETE-PE	778.503	20.4	16:1a/22:5(O)-PE	804.5548	16.32	16:1p/22:5(O)-PC
754.5027	17.97	16:0p/AA(2O)-PE	780.5186	16.57	18:1p/20:4(2O)-PE	804.5548	16.32	16:0p/22:6(2O)-PC
754.5027	18.61	16:0a/12-HETE-PE	780.5186	16.79	16:0p/22:5(2O)-PE	804.5548	16.79	18:2a/22:5(O)-PE
754.5027	18.88	16:0p/AA(2O)-PE	780.5186	17.25	18:1p/20:4(2O)-PE	804.5548	16.79	18:1p/22:6(2O)-PE
762.508	17.67	18:2p/HETE-PE	780.5186	18.07	18:1p/20:4(2O)-PE	804.5548	17.54	18:1a/HDoHE-PE
762.508	18.19	16:0p/HDoHE-PE	780.5186	18.11	16:0a/22:5(O)-PE	804.5548	17.8	18:1a/HDoHE-PE
762.508	18.71	16:0p/HDoHE-PE	780.5186	18.59	18:1a/12-HETE-PE	804.5548	18.14	18:1a/HDoHE-PE
762.508	18.88	16:0p/HDoHE-PE	780.5186	18.97	18:1p/20:4(2O)-PE	806.5342	16.77	18:2a/HETE-PC
762.508	21.02	16:0p/HDoHE-PE	780.5186	19.74	18:0a/20:5(O)-PE	806.5342	17.12	18:1p/22:5(2O)-PE
762.508	21.23	18:1p/20:5(O)-PE	780.5186	22.72	18:0a/20:5(O)-PE	806.5342	17.8	16:0a/HDoHE-PC
764.5238	18.82	16:0p/22:5(O)-PE	782.5341	19.36	18:0p/20:(2O)-PE	806.5342	17.84	18:2a/22:4(O)-PE
764.5238	19.2	16:0p/22:5(O)-PE	782.5341	19.62	16:0a/22:4(O)-PE	806.5342	18.3	18:1a/22:5(O)-PE
764.5238	19.68	18:1p/12-HETE-PE	782.5341	20.11	16:0a/12-HETE-PC	806.5342	18.61	18:0p/22:6(2O)-PE
764.5238	21.1	18:0p/20:5(O)-PE	782.5341	21.02	18:0a/12-HETE-PE	806.5342	19.36	18:0a/HDoHE-PE
766.5392	19.7	16:0e/22:5(O)-PE	790.5392	19.18	18:1p/22:5(O)-PE	806.5342	19.66	18:0a/HDoHE-PE
766.5392	20.88	16:0p/22:4(O)-PE	790.5392	20.38	18:0p/HDoHE-PE	806.5342	20.04	18:0a/HDoHE-PE
766.5392	21.33	18:0p/15-HETE-PE	790.5392	20.75	18:0p/HDoHE-PE	808.5501	18.45	16:0a/22:5(O)-PC
766.5392	22.38	18:0p/12-HETE-PE	792.5547	20.88	18:1p/22:4(O)-PE	808.5501	18.9	18:1a/12-HETE-PC
770.4978	13.42	16:0p/20:4(3O)-PE	792.5547	21.27	18:0p/22:5(O)-PE	808.5501	19.23	18:0p/22:5(2O)-PE
770.4978	13.81	16:0p/20:4(3O)-PE	792.5547	21.73	18:0p/22:5(O)-PE	808.5501	19.9	18:1a/22:4(O)-PE
770.4978	14.85	16:0p/DXA3-PE	792.5547	22.08	18:0p/22:5(O)-PE	808.5501	20.99	18:0a/22:5(O)-PE
770.4978	15.41	16:0a/20:4(2O)-PE	794.5701	23.87	18:0p/22:4(O)-PE	808.5501	21.28	20:1a/12-HETE-PE
770.4978	16.05	16:0a/20:4(2O)-PE	794.5701	25.8	20:0p/HETE-PE	810.5657	20.95	18:0p/22:4(2O)-PE
770.4978	16.1	16:0p/20:4(3O)-PE	796.5134	13.9	18:1p/20:4(3O)-PE	810.5657	21.12	18:0a/12-HETE-PC
770.4978	17.05	16:0a/20:4(2O)-PE	796.5134	13.97	16:0p/22:5(3O)-PE	814.5242	14.56	18:0a/PGE2/D2-PE
770.4978	17.84	16:0a/20:4(2O)-PE	796.5134	15.29	18:1p/DXA3-PE	814.5242	15.36	18:0p/20:4(4O)-PE
778.503	15.71	16:0p/22:6(2O)-PE	798.5291	16.45	18:0p/20:4(3O)-PE	814.5242	15.58	18:0a/20:4(3O)-PE
778.503	16.34	16:0p/22:6(2O)-PE	798.5291	17.42	18:0p/DXA3-PE	814.5242	15.58	18:0a/DXA3-PE
778.503	16.8	18:2a/HETE-PE	798.5291	17.75	18:0a/20:4(2O)-PE	814.5242	16.64	18:0a/20:4(3O)-PE
778.503	17.04	16:0a/HDoHE-PE	798.5291	17.75	16:0a/20:4(2O)-PC	814.5242	17.58	18:0p/20:4(4O)-PE

Table S2, related to Experimental Procedures. Oxidized phospholipids generated acutely by thrombin activated platelets. All lipids were structurally verified via MS/MS, with spectra included in Data S5. Retention time is based on elution on Orbitrap Elite.

Parent	Daughter	Name	Parent	Daughter	Name	Parent	Daughter	Name
738.6	219.1	16:0p/15-HETE-PE	778.6	317.2	18:1a/20:5(O)-PE	804.7	345.2	18:2a/22:5(O)-PE
738.6	167.1	16:0p/11-HETE-PE	778.6	345.2	16:1a/22:5(O)-PE	804.7	359.2	18:1p/22:6(2O)-PE
738.6	179.1	16:0p/12-HETE-PE	780.6	335.2	18:1p/20:4(2O)-PE	804.7	343.2	18:1a/HDoHE-PE
738.6	155.1	16:0p/8-HETE-PE	780.6	361.2	16:0p/22:5(2O)-PE	804.7	345.2	16:1p/22:5(O)-PC
738.6	319.2	16:0p/HETE-PE	780.6	345.2	16:0a/22:5(O)-PE	804.7	359.2	16:0p/22:6(2O)-PC
754.6	335.2	16:0p/20:4(2O)-PE	780.6	179.1	18:1a/12-HETE-PE	806.7	319.2	18:2a/HETE-PC
754.6	319.2	16:0a/HETE-PE	780.6	317.2	18:0a/20:5(O)-PE	806.7	361.2	18:1p/22:5(2O)-PE
754.6	179.1	16:0a/12-HETE-PE	782.6	335.2	18:0p/20:4(2O)-PE	806.7	343.2	16:0a/HDoHE-PC
762.6	319.2	18:2p/HETE-PE	782.6	347.2	16:0a/22:4(O)-PE	806.7	347.2	18:2a/22:4(O)-PE
762.6	343.2	16:0p/HDoHE-PE	782.6	179.1	16:0a/12-HETE-PC	806.7	345.2	18:1a/22:5(O)-PE
762.6	317.2	18:1p/20:5(O)-PE	782.6	179.1	18:0a/12-HETE-PE	806.7	359.2	18:0p/22:6(2O)-PE
764.6	345.2	16:0p/22:5(O)-PE	790.6	345.2	18:1p/22:5(O)-PE	806.7	343.2	18:0a/HDoHE-PE
764.6	179.1	18:1p/12-HETE-PE	790.6	343.2	18:0p/HDoHE-PE	808.7	345.2	16:0a/22:5(O)-PC
764.6	317.2	18:0p/20:5(O)-PE	792.6	347.2	18:1p/22:4(O)-PE	808.7	179.2	18:1a/12-HETE-PC
766.6	345.2	16:0e/22:5(O)-PE	792.6	345.2	18:0p/22:5(O)-PE	808.7	361.2	18:0p/22:5(2O)-PE
766.6	347.2	16:0p/22:4(O)-PE	794.6	347.2	18:0p/22:4(O)-PE	808.7	347.2	18:1a/22:4(O)-PE
766.6	219.1	18:0p/15-HETE-PE	794.6	319.2	20:0p/HETE-PE	808.7	345.2	18:0a/22:5(O)-PE
766.6	179.1	18:0p/12-HETE-PE	796.6	351.2	18:1p/20:4(3O)-PE	808.7	179.1	20:1a/12-HETE-PE
770.6	351.2	16:0p/20:4(3O)-PE	796.6	377.2	16:0p/22:5(3O)-PE	810.7	359.2	18:0p/22:4(2O)-PE
770.6	351.2	16:0p/DXA3-PE	796.6	351.2	18:1p/DXA3-PE	810.7	179.1	18:0a/12-HETE-PC
770.6	335.2	16:0a/20:4(2O)-PE	798.6	16.45	18:0p/20:4(3O)-PE	814.7	271.1	18:0a/PGE2/D2-PE
778.6	359.2	16:0p/22:6(2O)-PE	798.6	351.2	18:0p/DXA3-PE	814.7	367.2	18:0p/20:4(4O)-PE
778.6	319.2	18:2a/HETE-PE	798.6	335.2	18:0a/20:4(2O)-PE	814.7	351.2	18:0a/20:4(3O)-PE
778.6	343.2	16:0a/HDoHE-PE	798.6	335.2	16:0a/20:4(2O)-PC	814.7	351.2	18:0a/DXA3-PE

Table S3, related to Experimental Procedures. Parent to daughter transitions used for measuring oxPL generation by platelets. OxPL generation was measured using a 6500 Q-Trap as outlined in Methods. Note that several ions are detected more than once due to the presence of isobaric lipids, as seen in Table S2.

m/z	Full Name	Abbreviation/casual name	m/z	Full Name	Abbreviation/casual name
239.1656	Hydroxytetradecadienoic acid.	HTDE mixture	331.2645	Docosatetraenoic acid.	Adrenic
251.2021	Hexadecadienoic acid.	Palmitolinoleic	333.2073	Prostaglandin B2-like, 4.3min.	PGB2-like, 4.3 min
253.2177	Hexadecenoic acid isomer, 16.5min.	Palmitoleic isomer, 16.5 min	333.2073	Prostaglandin B2-like, 4.8min.	PGB2-like, 4.8 min
253.2177	Hexadecenoic acid isomer, 16.9min.	Palmitoleic isomer, 16.9 min	333.2073	Unknown m/z 333.2, 5.2min	Unknown m/z 333.2, 5.2 min
255.2332	Hexadecanoic acid.	Palmitic	333.2073	Unknown m/z 333.2, 5.9 min	Unknown m/z 333.2, 5.9 min
275.2019	Octadecatetraenoic acid.	Stearidonic	333.2802	Docosatrienoic acid isomer, 13.6min.	DTrA, 13.6 min
277.2176	Octadecatrienoic acid.	α-Linolenic	333.2802	Docosatrienoic acid isomer, 14.0min.	DTrA, 14.0 min
279.1964	12-Hydroxyheptadecatrienoic acid	12-HHTrE	335.2230	5,15-Dihydroxyeicosatetraenoic acid isomer,4.2min.	5,15-DiHETE isomer, 4.2 min
279.2332	Octadecadienoic acid.	Linoleic	335.2230	5,15-Dihydroxyeicosatetraenoic acid isomer, 4.5min.	5,15-DiHETE isomer, 4.5 min
281.2125	12-Hydroxyheptadecadienoic acid	12-HHDE	335.2230	Dihydroxyeicosatetraenoic acid-like, 5.0min.	DiHETE -like, 5.0 min
281.2486	Octadecenoic acid.	Oleic	335.2230	Dihydroxyeicosatetraenoic acid-like, 5.9min.	DiHETE -like, 5.9 min
283.2641	Octadecanoic acid.	Stearic	335.2230	Hepoxilin B3-like.	Hepoxilin B3 -like
291.1969	Hydroxyoctadecatetraenoic acid.	HOTE	335.2955	Docosadienoic acid.	DDA
293.2125	Hydroxyoctadecatrienoic acid.	HOTrE	337.2386	Dihydroxyeicosatrienoic acid-like, 4.9min.	DiHETrE -like, 4.9 min
295.2279	9/13-Hydroxyoctadecadienoic acid mixture.	9/13-HODE mixture	337.2386	Dihydroxyeicosatrienoic acid-like, 6.1min.	DiHETrE -like, 6.1 min
297.2435	9-Hydroxyoctadecenoic acid.	9-HOME	337.2386	Deoxy-ProstaglandinF2a-like.	Deoxy-PGF2a-like.
295.2644	Nonadecenoic acid .	NMA	341.2104	Unknown m/z 341.2, 9.9 min	Unknown m/z 341.2, 9.9 min
297.2801	Nonadecanoic acid.	NA	343.2282	hydroxy-docosahexaenoic acid mixture	HDoHE mixture
301.2176	Eicosapentaenoic acid.	EPA	343.2282	14-Hydroxydocosahexaenoic acid.	14-HDoHE
303.2331	Eicosatetraenoic acid.	Arachidonic	345.2438	20-Hydroxydocosapentaenoic (n-3) acid.	20-HDoPE (n-3)
305.2126	14-Hydroxynonadecatetraenoic acid isomer.	14-HNTE isomer	345.2438	17-Hydroxydocosapentaenoic (n-3) acid.	17-HDoPE (n-3)
305.2126	14-Hydroxynonadecatetraenoic acid.	14-HNTE	345.2438	14-Hydroxydocosapentaenoic (n-3) acid.	14-HDoPE (n-3)
305.2486	Eicosatrienoic acid isome, 18.7min.	ETrA isomer, 18.7 min	345.2438	14-Hydroxydocosapentaenoic (n-6) acid.	14-HDoPE (n-6)
305.2486	Eicosatrienoic acid isomer, 19.3min.	ETrA isomer, 19.3 min	347.2595	17-Hydroxydocosatetraenoic (n-3) acid.	17-HDoTE (n-3)
307.1918	Dihydroyxyoctadecatetraenoic acid -like	DiHOTE -like	347.2595	13-Hydroxydocosatetraenoic (n-3) acid.	13-HDoTE (n-3)
307.2282	14-Hydroxynonadecatrienoic acid.	14-HNTrE	347.2595	14-Hydroxydocosatetraenoic (n-3) acid.	14-HDoTE (n-3)
307.2644	Eicosadienoic acid.	EDA	349.2752	14-Hydroxydocosatrienoic (n-6) acid.	14-HDoTrE (n-6)
309.2438	14-Hydroxynonadecadienoic acid.	14-HNDE	349.2752	14-Hydroxydocosatrienoic (n-9) acid.	14-HDoTrE (n-9)
309.2801	Eicosenoic acid.	EMA	351.2180	Prostaglandin E2.	PGE2
311.2957	Eicosanoic acid.	Arachidic	351.2180	Prostaglandin D2.	PGD2
315.1970	Prostaglandin A2-like.	PGA2-like, 4.1 min	351.2180	Prostanoid m/z 351.2, 2.8 min	Prostanoid m/z 351.2, 2.8 min
315.1970	Prostaglandin A2-like.	PGA2-like, 4.3 min	351.2180	Prostanoid m/z 351.2, 3.0 min	Prostanoid m/z 351.2, 3.0 min
317.2125	12-Hydroxyeicosapentaenoic acid.	12-HEPE	351.2180	Prostanoid m/z 351.2, 3.2 min	Prostanoid m/z 351.2, 3.2 min
319.2276	12-Hydroxyeicosatetraenoic acid.	12-HETE	351.2180	DXA3-like.	DXA3-like
321.2437	12-Hydroxyeicosatrienoic acid isomer, 11.1min.	12-HETrE isomer, 11.1 min	351.2180	8-hydroxyl-9,11-dioxolane A3.	DXA3
321.2437	12-Hydroxyeicosatrienoic acid isomer,12.1min.	12-HETrE isomer, 12.1 min	353.2335	Prostaglandin F2-like, 2.7min.	PGF2 -like, 2.7 min
323.2595	11-Hydroxyeicosadienoic acid isomer, 12.6min.	11-HEDE isomer, 12.6 min	353.2335	Prostaglandin F2-like, 2.9min.	PGF2 -like, 2.9 min
323.2595	11-Hydroxyeicosadienoic acid isomer, 13.4min.	11-HEDE isomer, 13.4 min	353.2335	Prostaglandin F2-like, 3.2min.	PGF2 -like, 3.2 min
327.2334	Docosahexaenoic acid.	DHA	353.2335	Unknown m/z 353.2, 3.42 min	Unknown m/z 353.2, 3.42 min
329.2486	Docosapentaenoic acid isomer.	DPA	369.2285	Thromboxane B2.	TXB2
329.2486	Docosapentaenoic acid-like.	DPA -like			

Table S4, related to Figure 1H. Structures of fatty acids and eicosanoids generated by thrombin-activated platelets. All these lipids were manually verified via MS/MS on the Orbitrap Elite platform. Spectra are provided in Data S6.

Ion	Mode
[M-H]-	Negative
[M+H]+	Positive
[M+Na]+	Positive
[M+NH_4]+	Positive
[M+CH_3COO]-	Negative

Table S5, related to Experimental Procedures. Adduct ions retained after database searching.

Figure S1

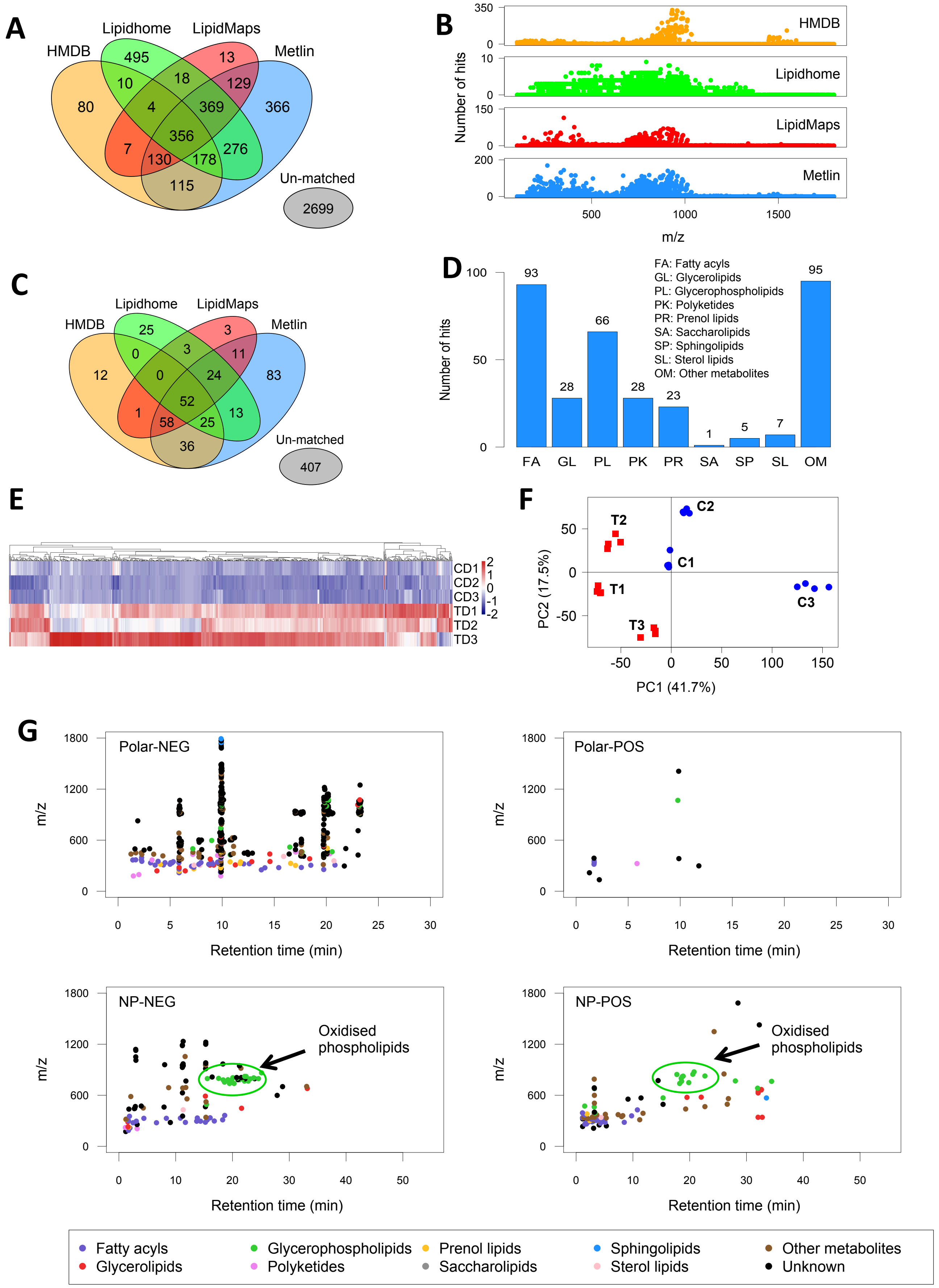
A
HMDB
Lipidhome
LipidMaps
Metlin
495
13
10
18
129
80
4
369
366
356
7
276
130
178
115
Un-matched
2699
B
Number of hits
HMDB
Lipidhome
LipidMaps
Metlin
350
0
10
0
150
0
200
0
500
1000
1500
m/z
C
HMDB
Lipidhome
LipidMaps
Metlin
25
3
0
3
11
12
0
24
83
52
1
13
58
25
36
Un-matched
407
D
Number of hits
FA: Fatty acyls
GL: Glycerolipids
PL: Glycerophospholipids
PK: Polyketides
PR: Prenol lipids
SA: Saccharolipids
SP: Sphingolipids
SL: Sterol lipids
OM: Other metabolites
93
28
66
28
23
1
5
7
95
100
50
0
FA
GL
PL
PK
PR
SA
SP
SL
OM
E
CD1
CD2
CD3
TD1
TD2
TD3
2
1
0
-1
-2
F
PC2 (17.5%)
PC1 (41.7%)
T2
C2
C1
T1
C3
T3
50
0
-50
-50
0
50
100
150
G
Polar-NEG
Polar-POS
NP-NEG
NP-POS
m/z
Retention time (min)
1800
1200
600
0
0
5
10
15
20
25
30
0
10
20
30
40
50
Oxidised phospholipids
Fatty acyls
Glycerolipids
Glycerophospholipids
Polyketides
Prenol lipids
Saccharolipids
Sphingolipids
Sterol lipids
Other metabolites
Unknown

Figure S1, related to Figure 1. Putative identification of lipids present basally and on thrombin activation of platelets. *Panel A. Results of database searches on basal ions.* Ions common to ≥ 2 donors are shown that were assigned at least one putative match, and those with no match. *Panel B. Number of hits in databases per m/z value*. *Panel C. Results of database searchers on thrombin-upregulated ions*. Ions common to ≥ 2 donors are shown that were assigned at least one putative match, and those with no match. *Panel D. Predominant lipid classes upregulated by thrombin in platelets.* Ions were grouped according to the most predominant classification family based on classifications in databases *Panel E. Heat map showing the diversity of thrombin responses for individual donors*. Lipids that upregulated on thrombin activation from control (C1-3) and thrombin-activated (T1-3) platelet extracts were plotted using a heat map. Note that Donor 3 upregulates many lipids differentially and more strongly than either Donors 1 or 2. *Panel F. Principal Component Analysis showing the effect of thrombin on platelet lipids.* Donors' basal (C1-3) and thrombin-elevated (T1-3) lipids were analyzed, with four technical replicates per donor. Donors are separated in both PC1 and PC2, but thrombin causes similar changes in PC1 for all donors. *Panel G. Scatter diagrams showing elution of lipids from Polar or NP columns, in either negative or positive ion mode*. Lipids are color coded according to classification from databases. Putative identifications for all are in Supplementary Data File 1.

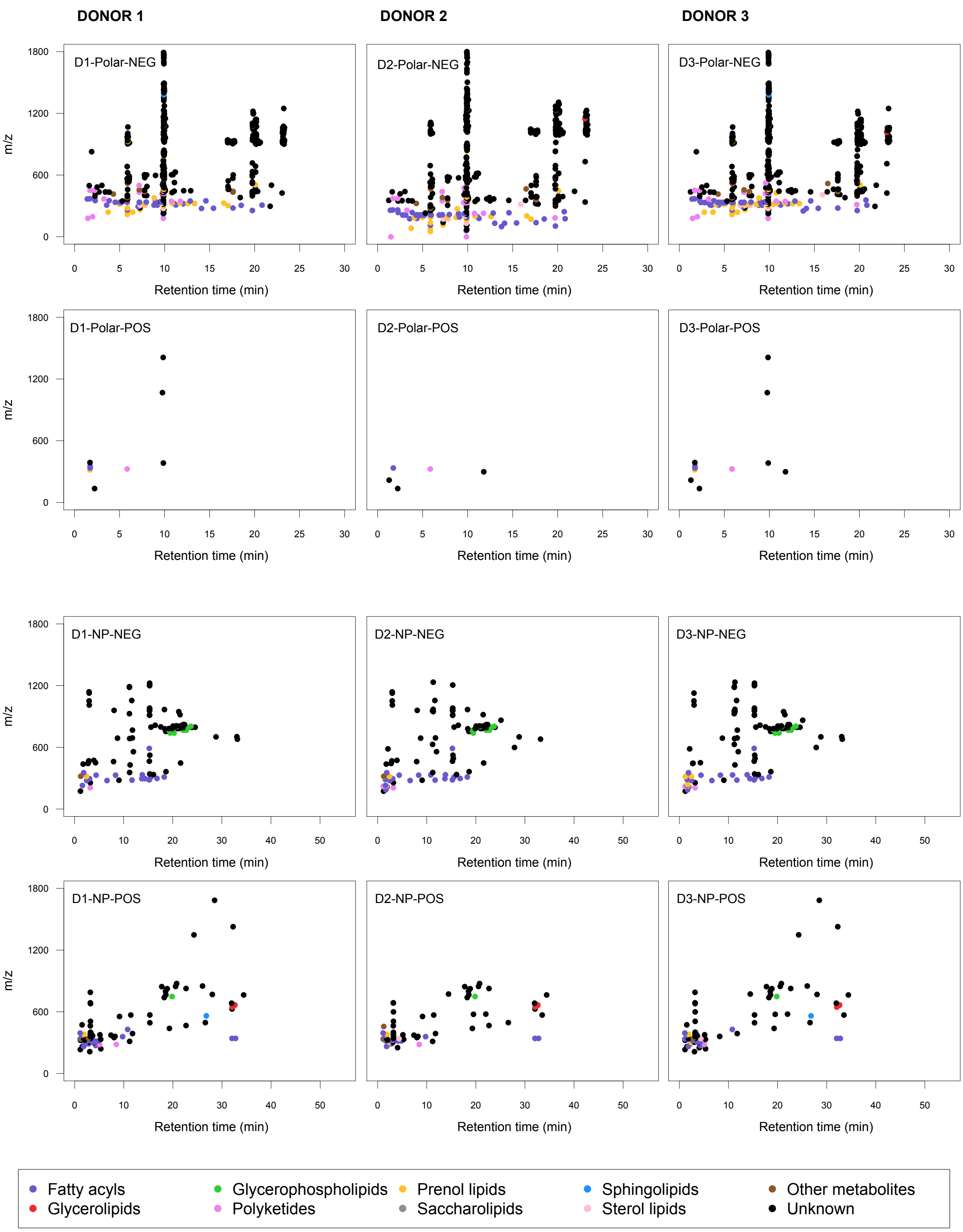

Figure S2, related to Figure 1. Donor specific thrombin-upregulated lipids shown as scatter diagrams. Scatter diagrams were generated as described in Figure 2 H, following LC/FTMS analysis of control and thrombin-activated platelet lipid extracts as described in Methods.

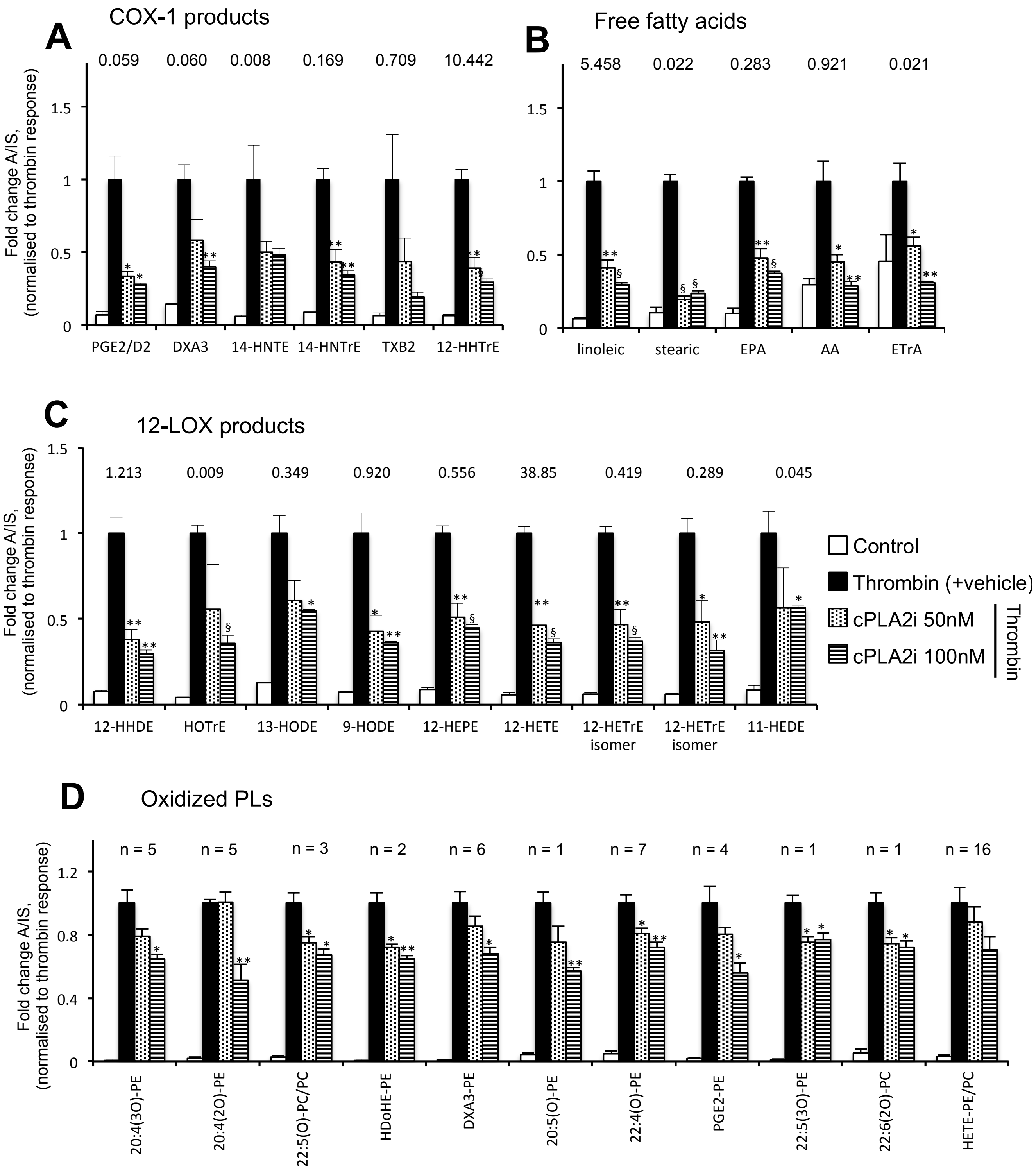

Figure S3, related to Figure 5. Inhibition of $cPLA_2$ suppresses generation of FAs and oxPLs. Washed platelets were incubated with 50-100 nM $cPLA_2i$ or vehicle for 15 min, then activated using 0.2 U/ml thrombin for 30 min and generation of lipids determined using LC/MS/MS as described in Supplementary Methods. For Panel D, oxPL are grouped depending on the *sn2* fatty acid to aid data visualization. Individual lipids for this data are shown in Supplementary 5. Figure A-C: Numbers above each refer to the analyte:internal standard integrated area for the thrombin activated sample.

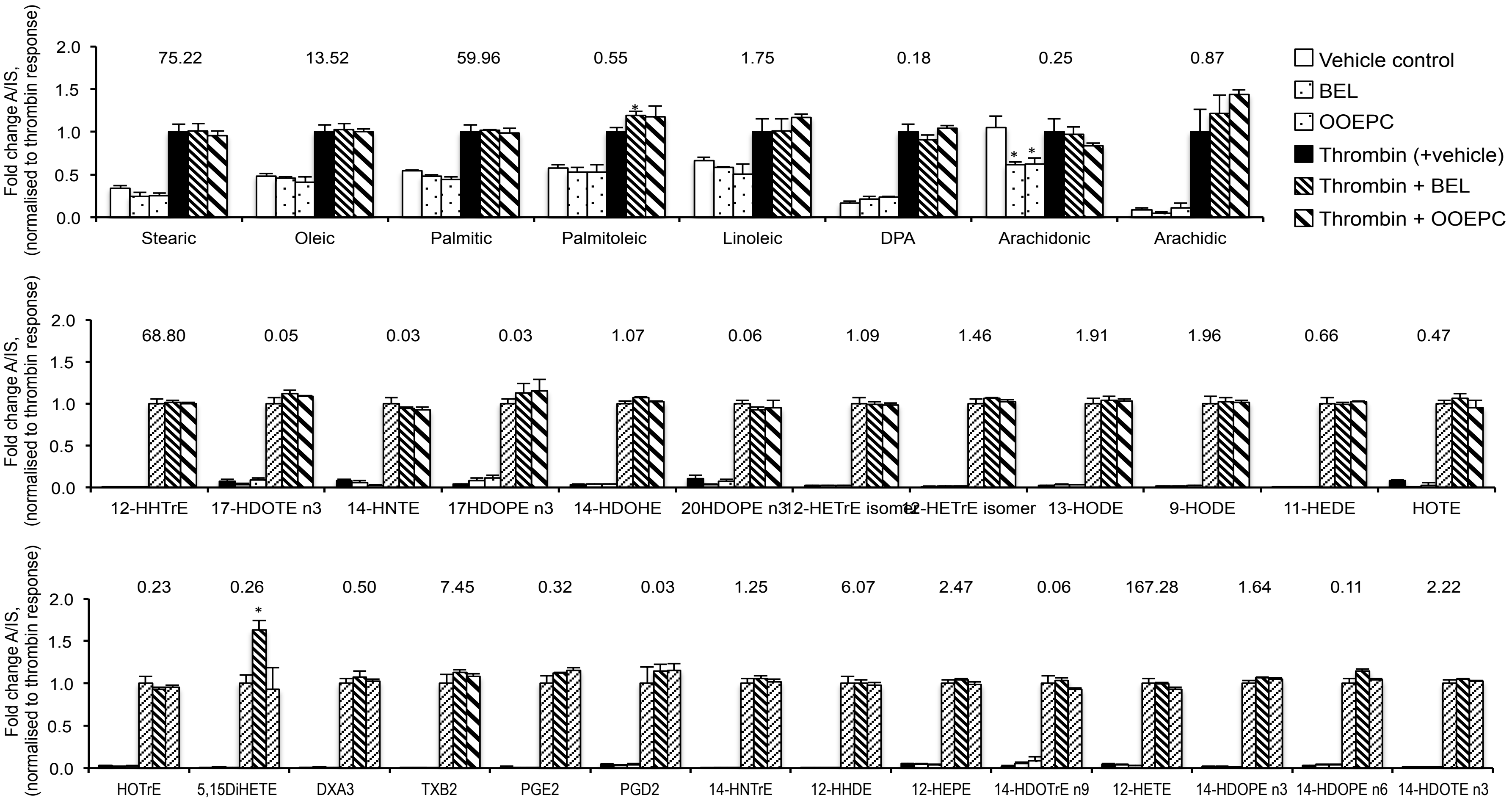

Figure S4, related to Figure 5. Effect of PLA$_2$ inhibitors on generation of FAs by platelets. Washed platelets were incubated with inhibitors or vehicle for 15 min, then activated using 0.2 U/ml thrombin for 30 min and generation of lipids determined using LC/MS/MS as described in Supplementary Methods. Inhibitors were as follows: iPLA$_2$: 50 nM BEL and sPLA$_2$: 2 μM OOEPC. Numbers above each refer to the analyte:internal standard integrated area for the thrombin activated sample.

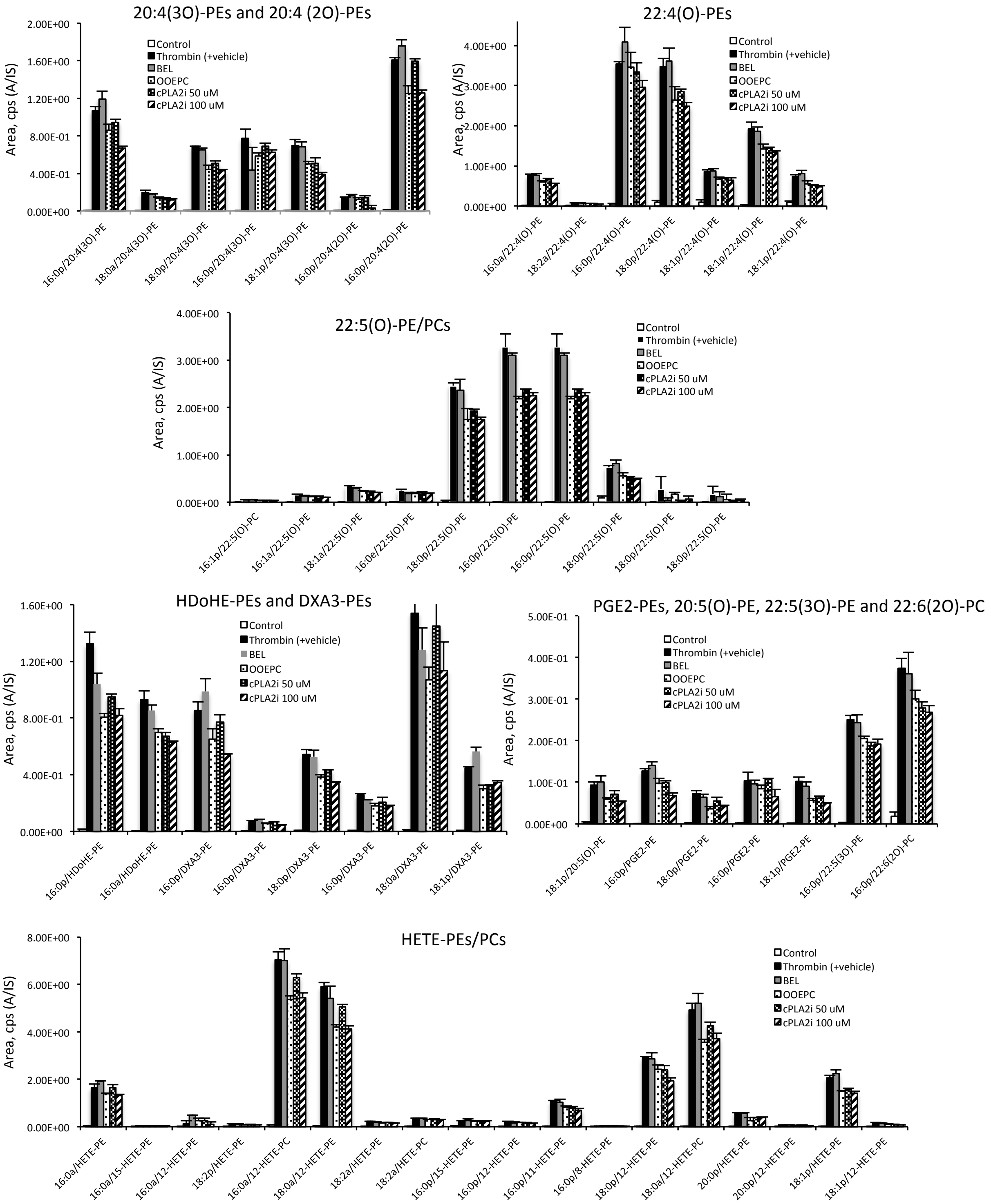

Figure S5, related to Figure 5. Bar charts showing effects of PLA_2 inhibitors on levels of individual oxPL. Lipids were analyzed by LC/MS/MS, on a 6500 Q-Trap, as described in Methods. Some of this data is combined based on *sn2* functional group and summarized in Supplementary Figure 3 D.

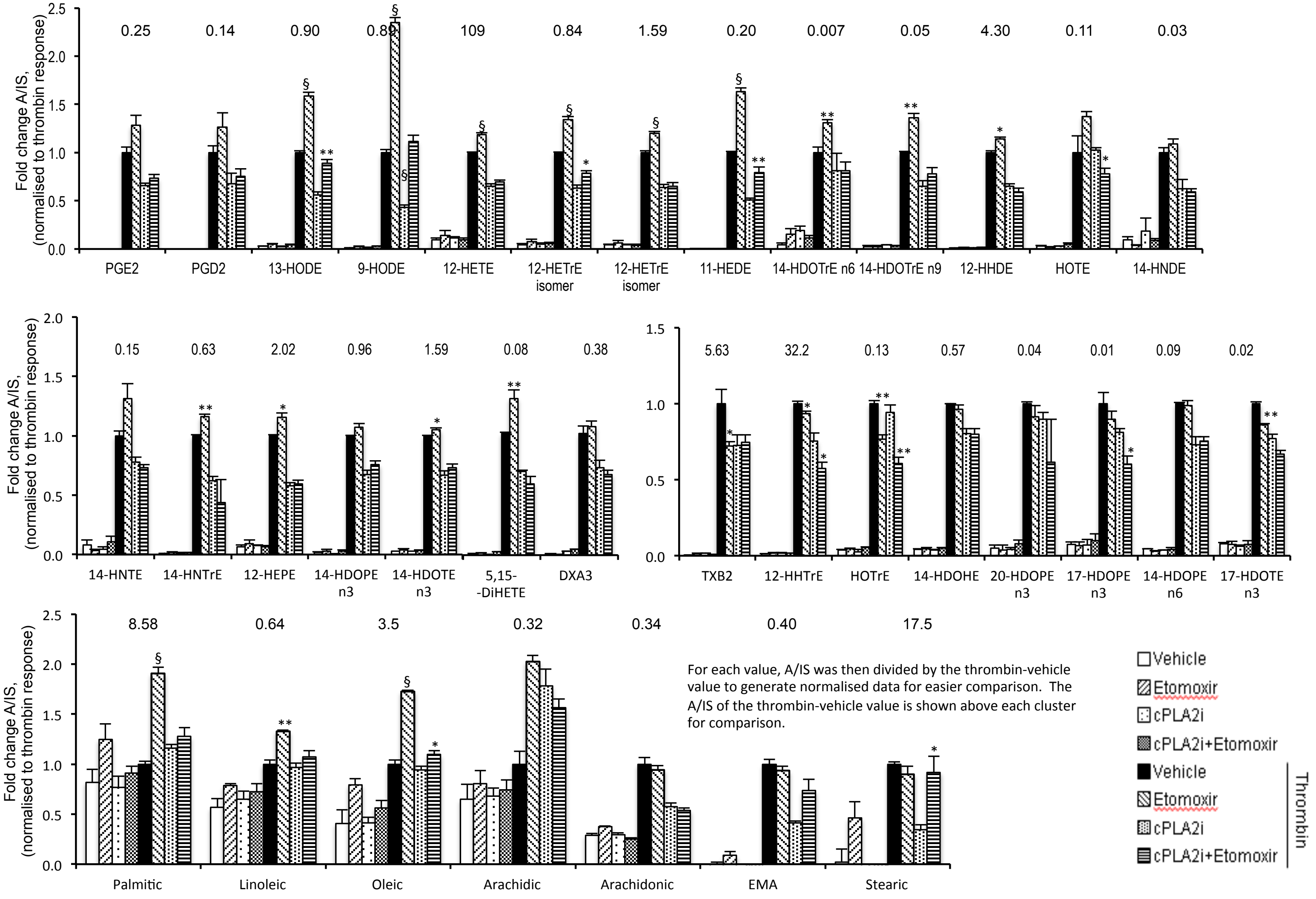

Figure S6, related to Figure 6. Several eicosanoids are substrates for platelet β-oxidation on thrombin generation. Washed platelets were incubated with inhibitors or vehicle, then activated using 0.2 U/ml thrombin at 37 ^{0}C for 30 min and generation of lipids determined using LC/MS/MS as described in Supplementary Methods (n = 3). Inhibitors were as follows: Etomoxir (Eto): 25 µM, cPLA$_2$i: 100 nM. n = 3, mean ± SEM, 1-way ANOVA with Bonferroni post hoc test. Numbers above each refer to the analyte:internal standard integrated area for the thrombin activated sample.

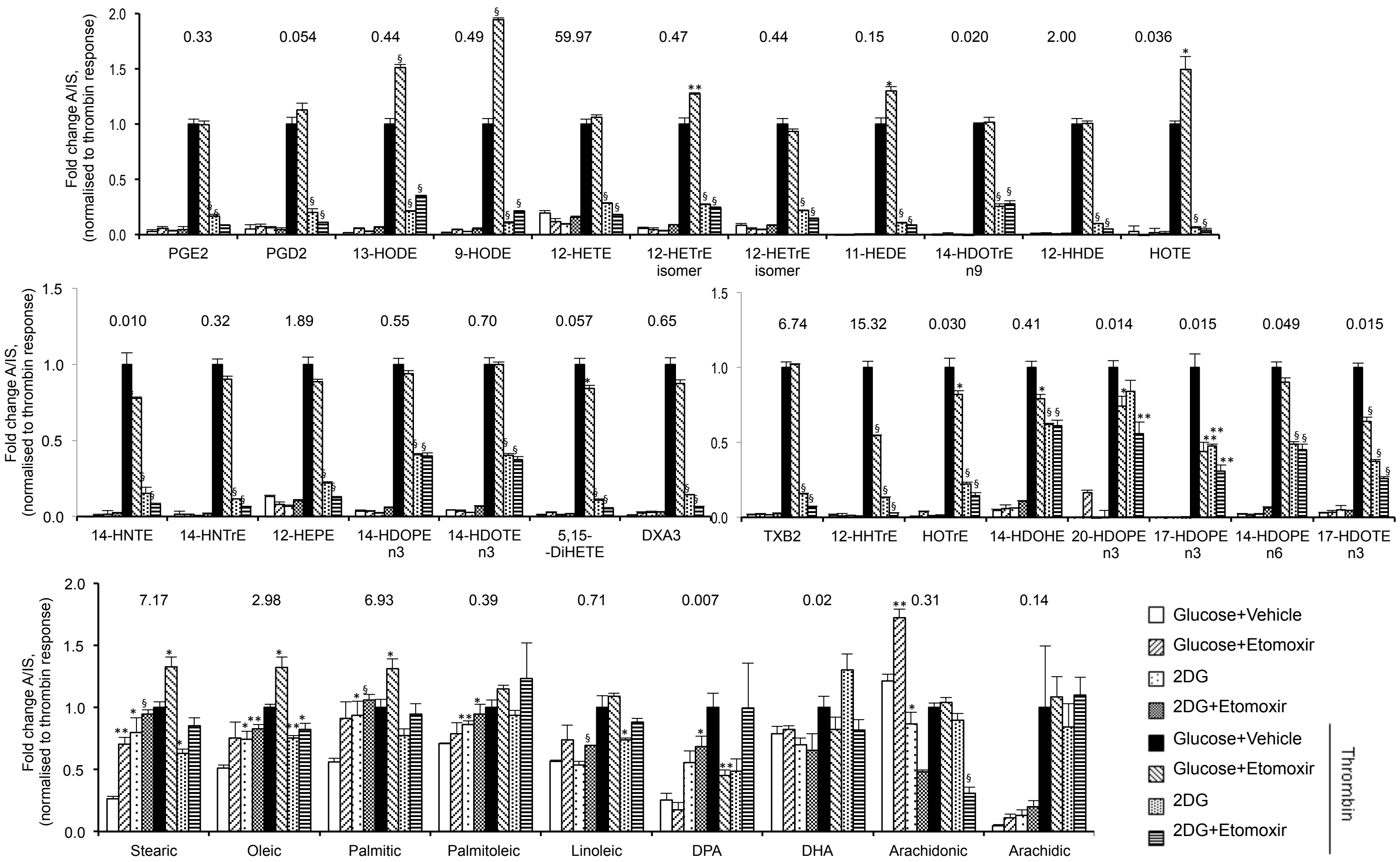

Figure S7, related to Figure 6. Inhibition of glycolysis leads to a failure to generate free fatty acids and eicosanoids on thrombin activation of platelets. Washed platelets were incubated with inhibitors or vehicle for 60 min at RT, then activated using 0.2 U/ml thrombin at 37 ^{0}C for 30 min and generation of lipids determined using LC/MS/MS as described in Supplementary Methods (n = 3). Cells were either incubated in the presence (Glc) or absence of 5 mM glucose (Glc). Inhibitors were as follows: Eto: 25 µM, 2-deoxy-D-glucose (2DG): 120 mM. n = 3, mean ± SEM, 1-way ANOVA with Bonferroni post hoc test. Numbers above each refer to the analyte:internal standard integrated area for the thrombin activated sample.

Viewing GoogleVis files in a browser

To view GoogleVis interactive html files in a browser the settings for Adobe (Macromedia) Flash need to be updated.

Open the URL:
http://www.macromedia.com/support/documentation/en/flashplayer/help/settings_manager04.html

The Global Security Settings panel will be displayed as below.
Click on the third tab (Global Security Settings).

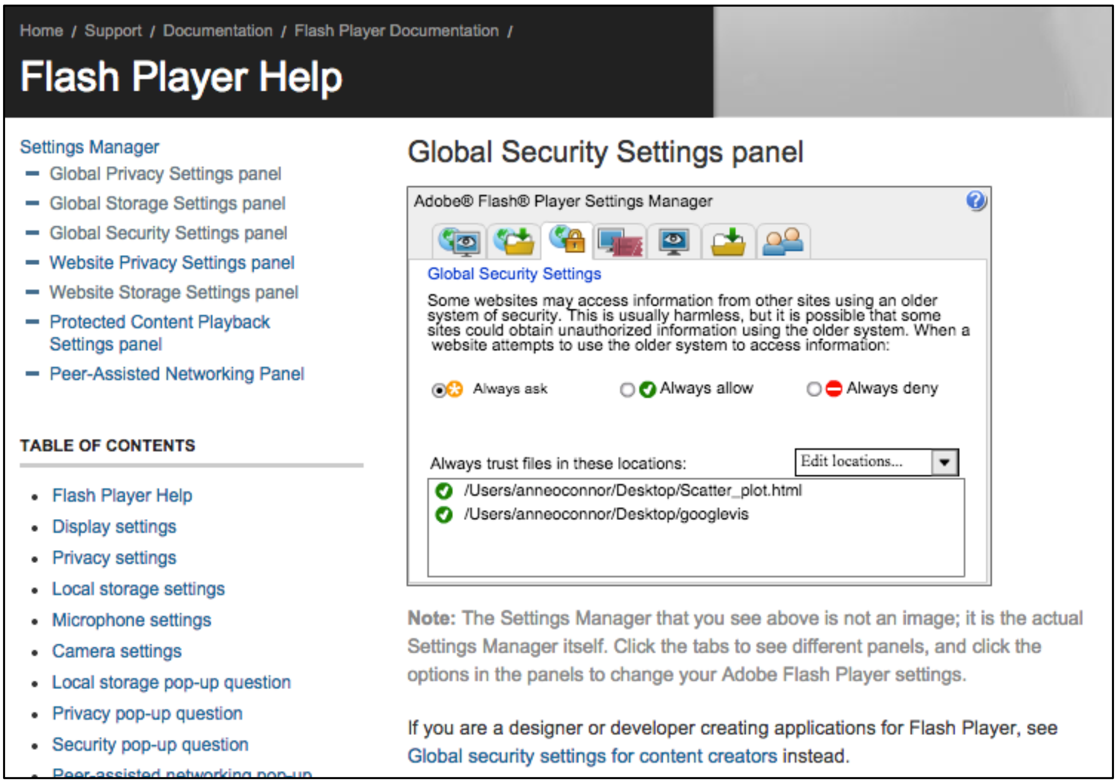

In the 'Edit Locations' dropdown box, select 'Add Location'.
Select 'Browse for files…' (or 'Browse for folder…').

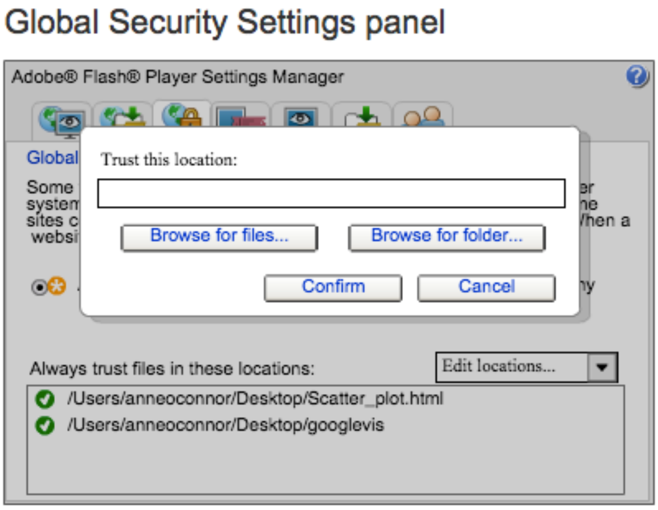

Navigate to the location of the GoogleVis html file(s), select file(s) and click Open.
The file(s) that you selected should now be listed in the box 'Always trust files in these locations:"

You can now open the html file(s) by clicking on them. Each point on the scatter diagram represents one lipid species. You can hover over a point to get the lipid category for this species, the retention time and the *m/z* value.

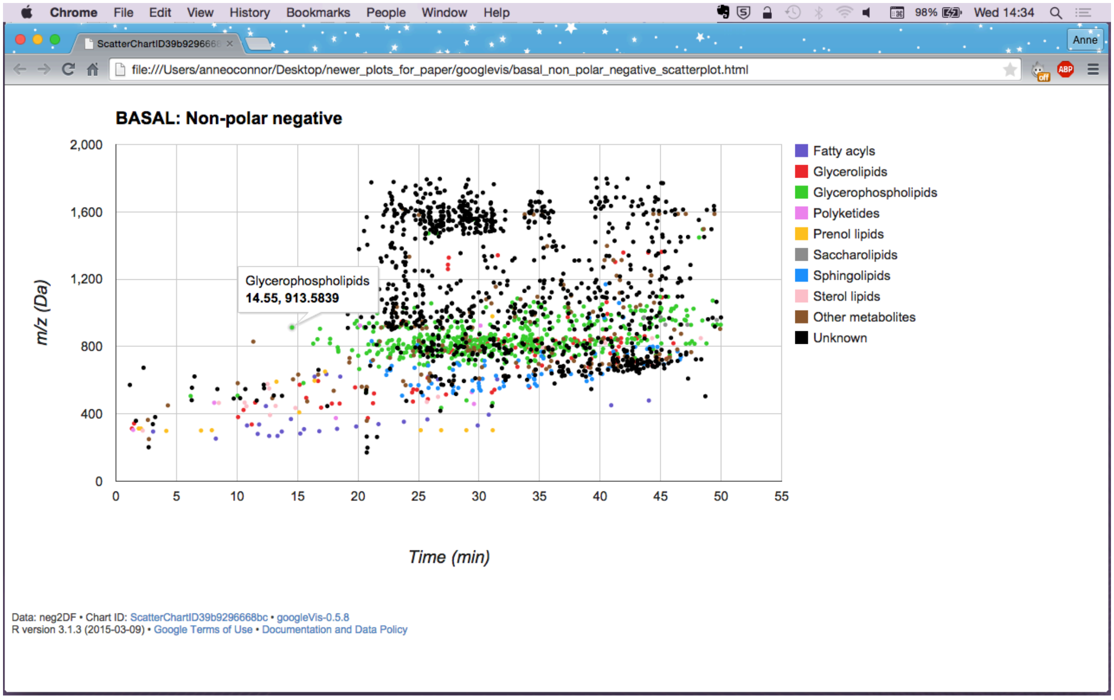

You can zoom in on sections of a plot to see points clearer. Just drag the cursor over an area to zoom into.

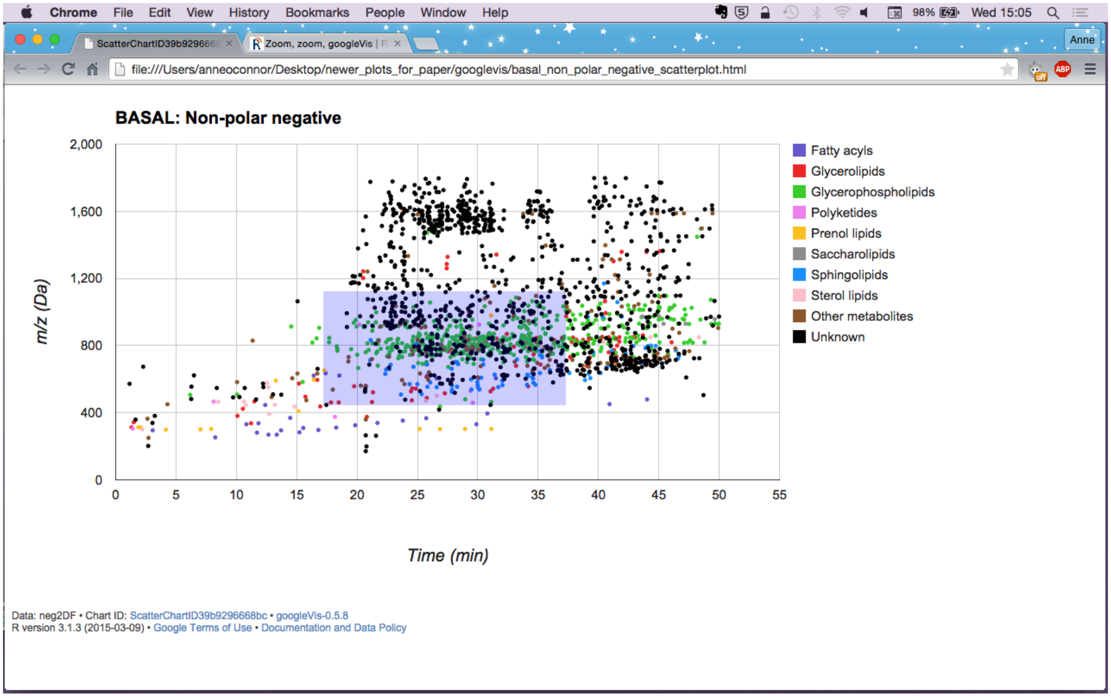

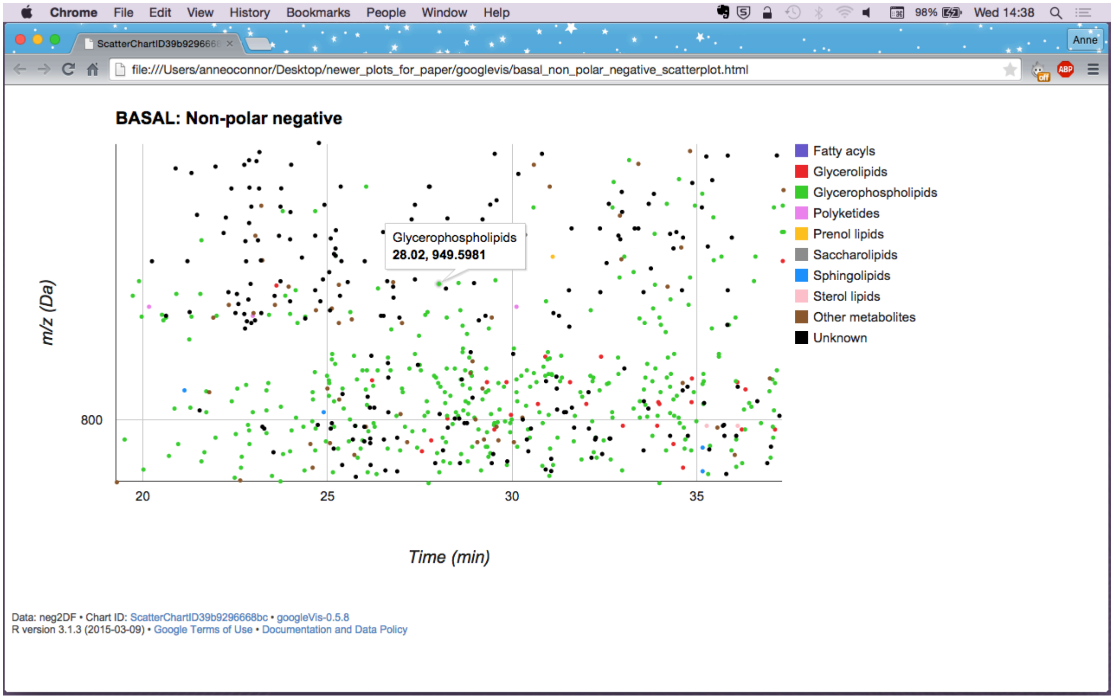

You can also hover over a category in the legend to highlight all points in that category on the scatter diagram.

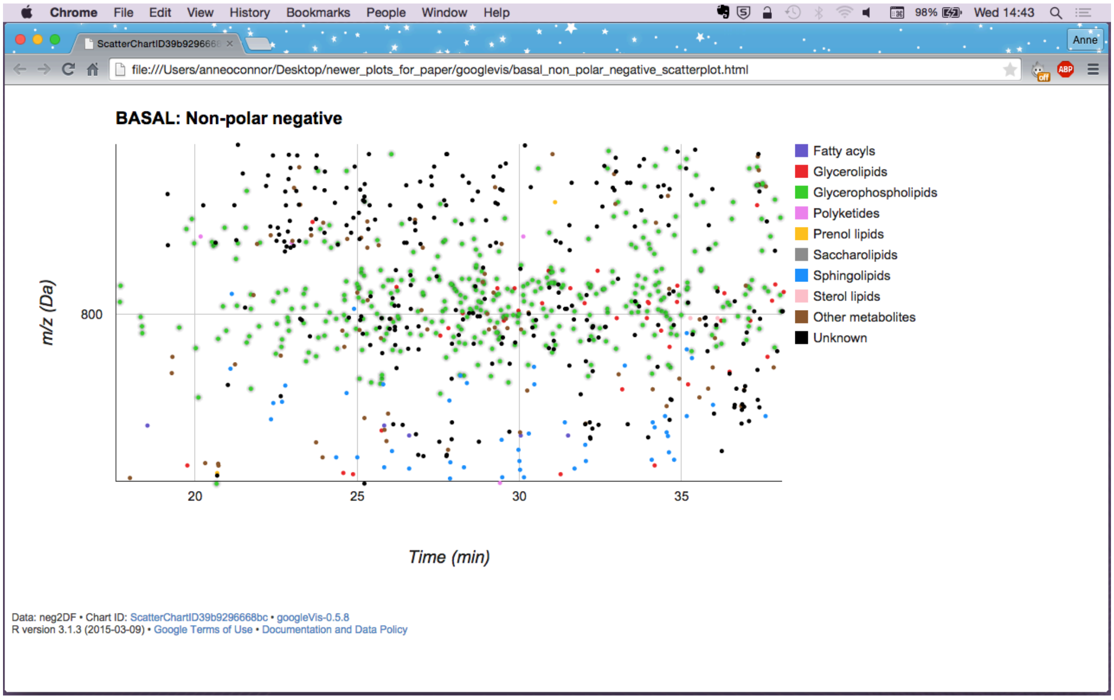

To zoom back out you can just refresh the page or right click.

NOTE: This has been tested on Google Chrome and Safari only.
