# Supplementary material for: Mapping the Human Platelet Lipidome Reveals Cytosolic Phospholipase A2 as a Regulator of Mitochondrial Bioenergetics during Activation
Source: Cell Metab. 2016 May 10;23(5):930–44. doi: 10.1016/j.cmet.2016.04.001 (PMC4873619; doi:10.1016/j.cmet.2016.04.001)
Supplement: Data S2. GoogleVis Interactive Diagrams, Related to Figure 1G [file mmc3.zip › Data S2/Viewing GoogleVis files in a browser.pdf]

Viewing GoogleVis files in a browser

To view GoogleVis interactive html files in a browser the settings for Adobe (Macromedia) Flash need to be updated.

Open the URL:
http://www.macromedia.com/support/documentation/en/flashplayer/help/settings_manager04.html

The Global Security Settings panel will be displayed as below.
Click on the third tab (Global Security Settings).

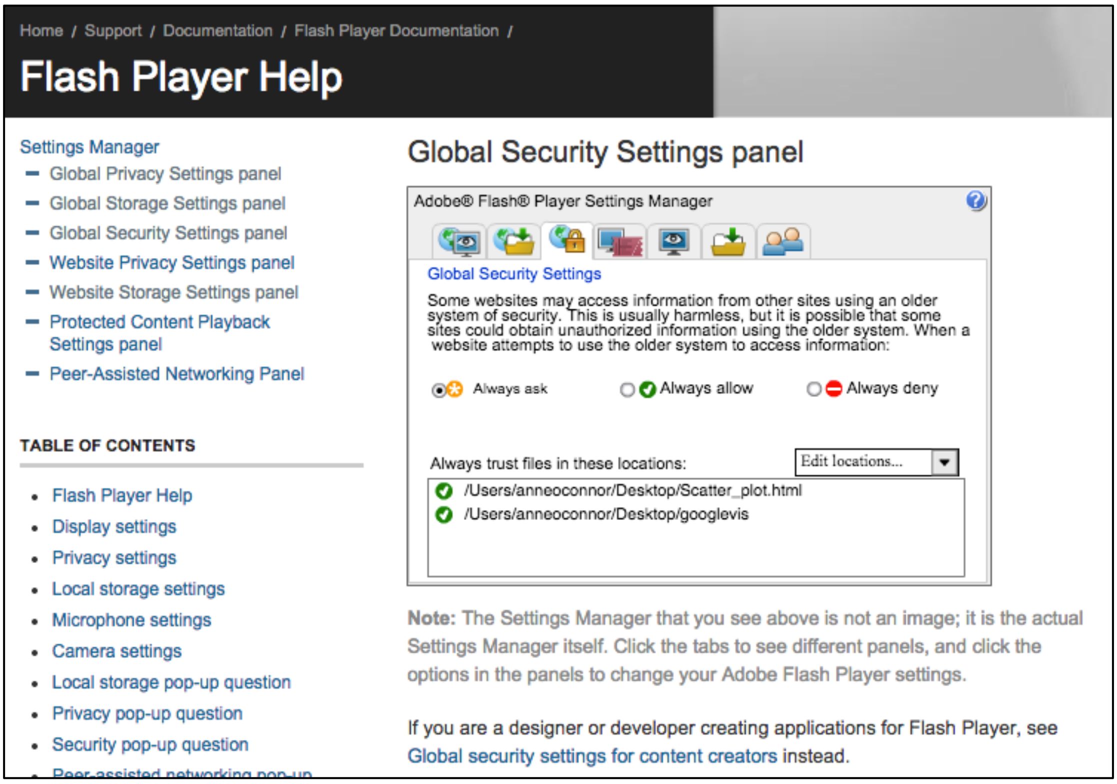

In the 'Edit Locations' dropdown box, select 'Add Location'.
Select 'Browse for files…' (or 'Browse for folder…').

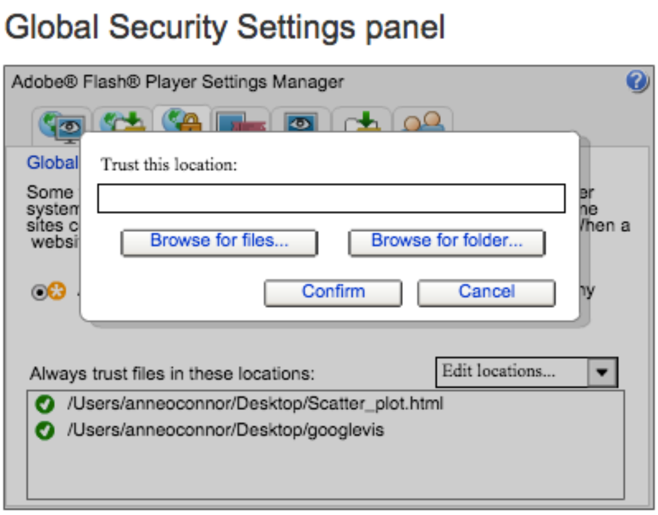

Navigate to the location of the GoogleVis html file(s), select file(s) and click Open.
The file(s) that you selected should now be listed in the box 'Always trust files in these locations:"

You can now open the html file(s) by clicking on them. Each point on the scatter diagram represents one lipid species. You can hover over a point to get the lipid category for this species, the retention time and the *m/z* value.

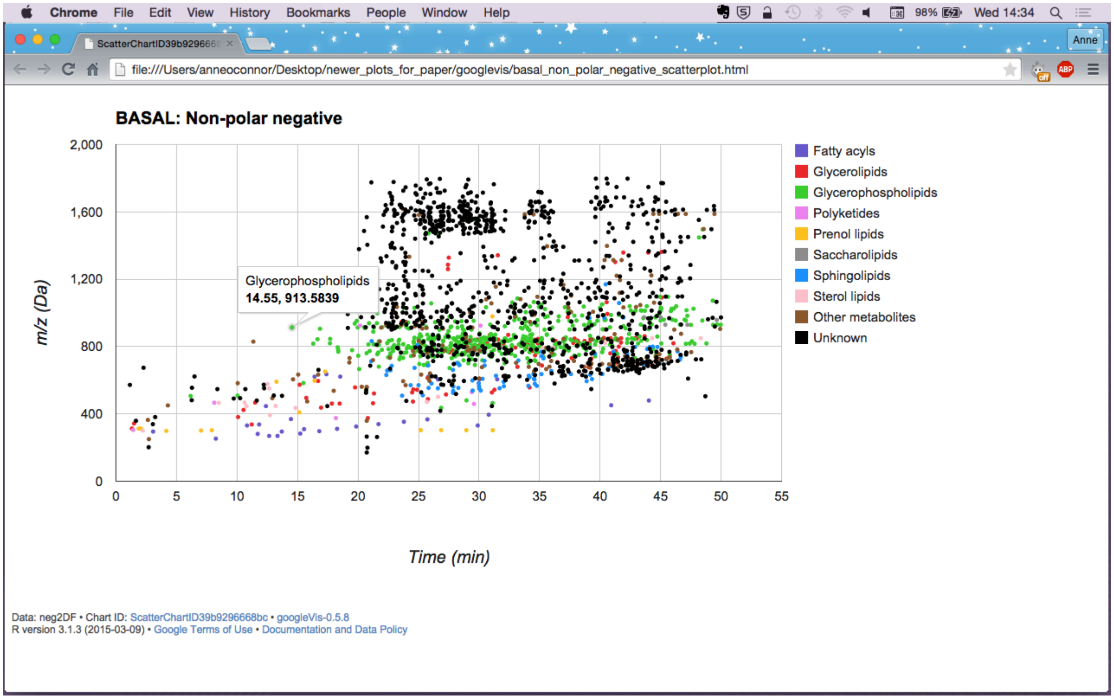

You can zoom in on sections of a plot to see points clearer. Just drag the cursor over an area to zoom into.

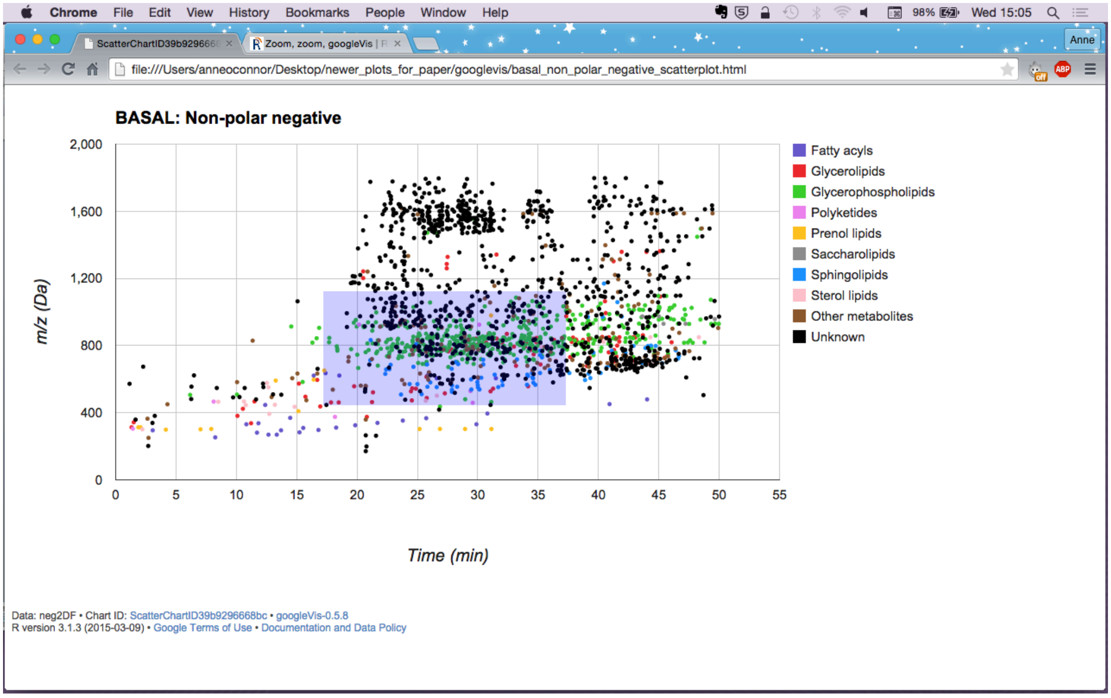

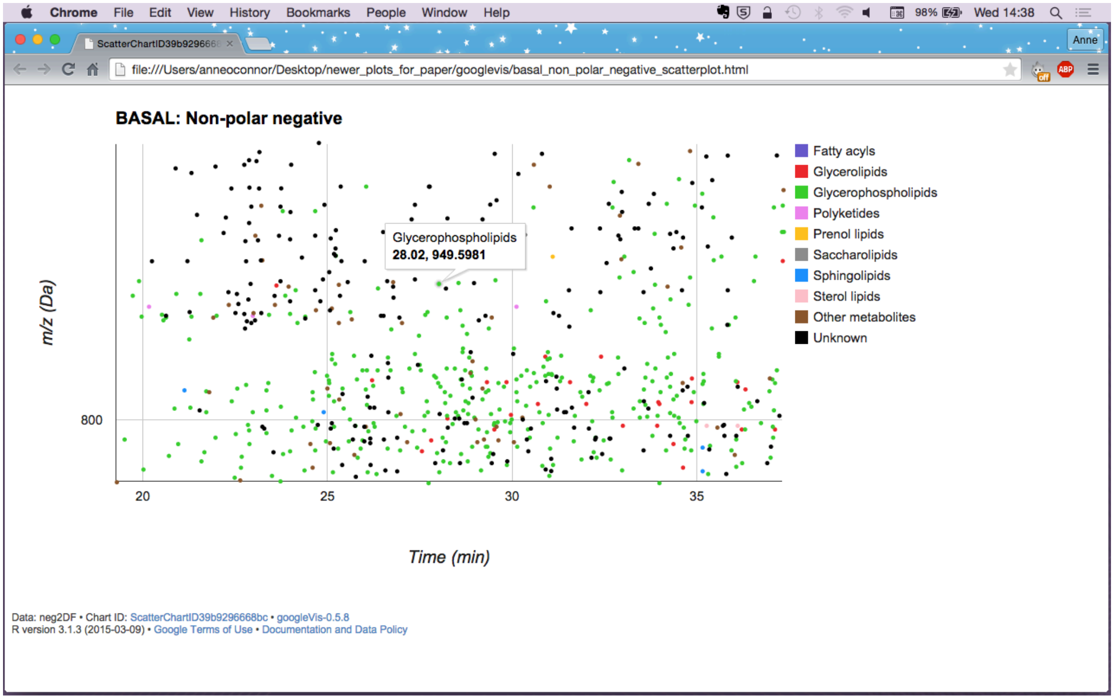

You can also hover over a category in the legend to highlight all points in that category on the scatter diagram.

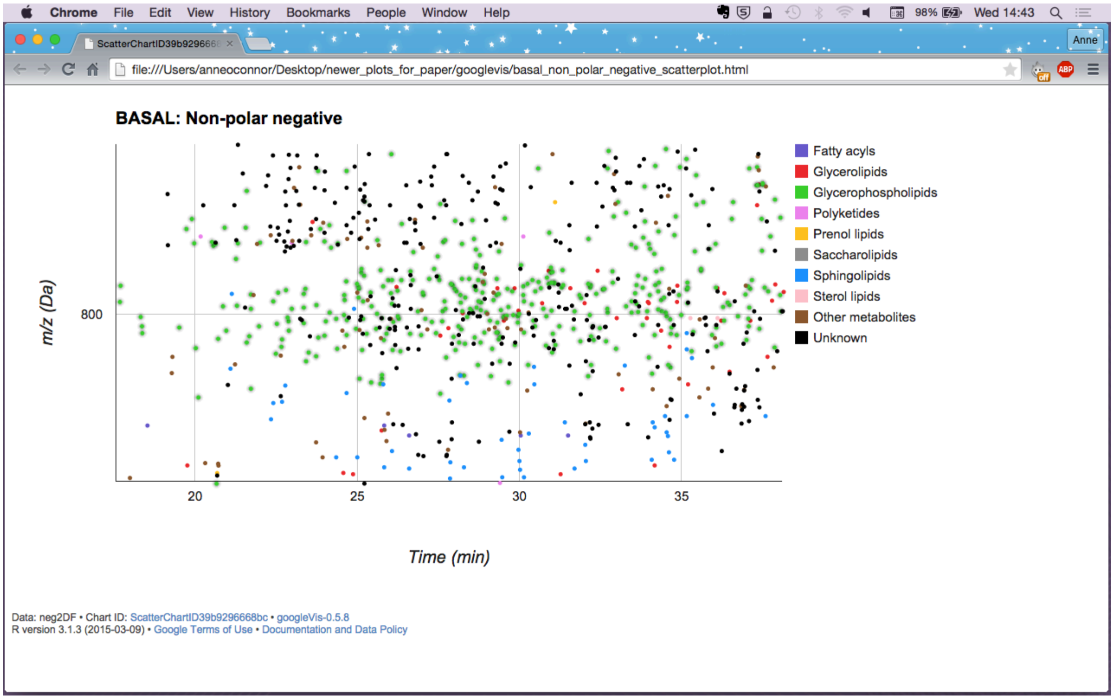

To zoom back out you can just refresh the page or right click.

NOTE: This has been tested on Google Chrome and Safari only.
