# Supplementary material for: Mapping the Human Platelet Lipidome Reveals Cytosolic Phospholipase A2 as a Regulator of Mitochondrial Bioenergetics during Activation
Source: Cell Metab. 2016 May 10;23(5):930–44. doi: 10.1016/j.cmet.2016.04.001 (PMC4873619; doi:10.1016/j.cmet.2016.04.001)
Supplement: Data S3. GoogleVis Interactive Diagrams, Related to Figure 1H [file mmc4.zip › Data S3/Supplementary Data File 3.4.html]

ScatterChartID39b921916959


Data: pos1DF • Chart ID: ScatterChartID39b921916959 • googleVis-0.5.8  


R version 3.1.3 (2015-03-09)
• Google Terms of Use • Documentation and Data Policy
