# Supplementary material for: Mapping the Human Platelet Lipidome Reveals Cytosolic Phospholipase A2 as a Regulator of Mitochondrial Bioenergetics during Activation
Source: Cell Metab. 2016 May 10;23(5):930–44. doi: 10.1016/j.cmet.2016.04.001 (PMC4873619; doi:10.1016/j.cmet.2016.04.001)

Structural identification of m/z 738.5079 as 16:0p/HETE-PE isomers

Five isomers are detected, with four identified as positional isomers using MS^3

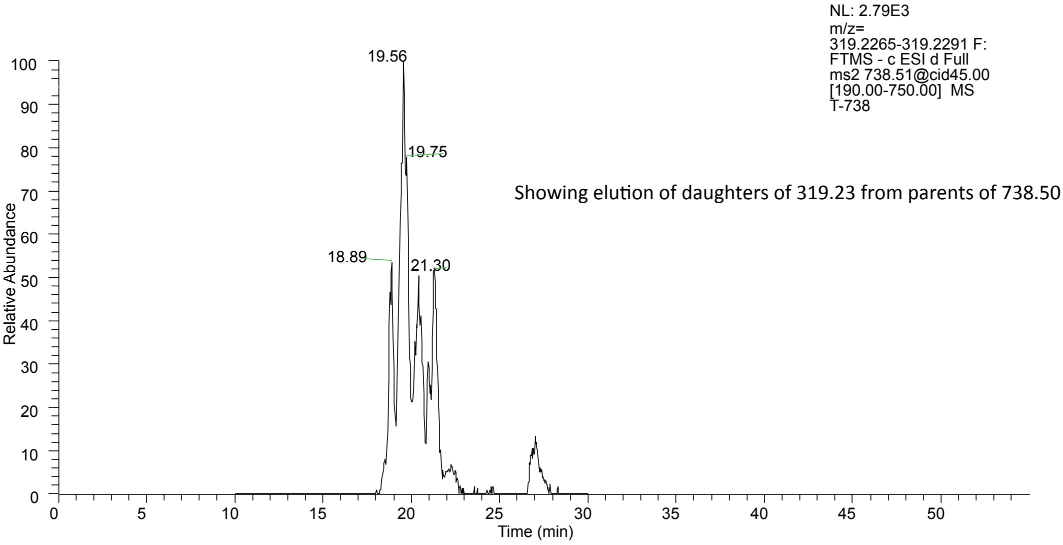

Ion eluting at 18.9 min, 16:0p/15-HETE-PE

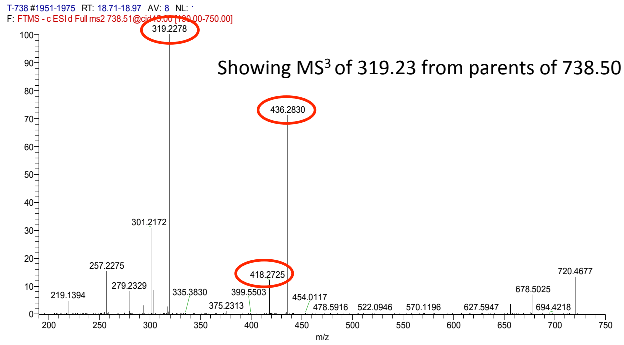

Ion eluting at 19.5 min, 16:0p/11-HETE-PE

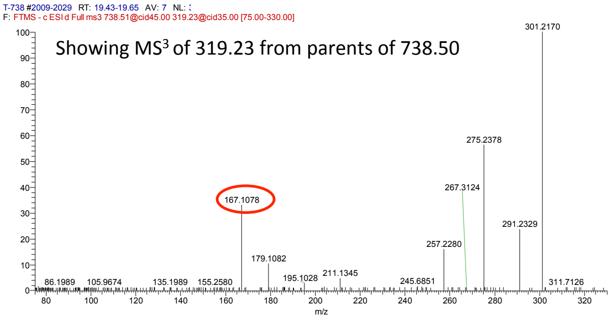

Ion eluting at 19.8 min, 16:0p/12-HETE-PE

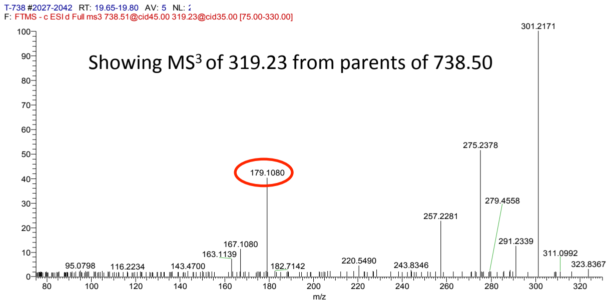

Ion eluting at 20.4 min, 16:0p/8-HETE-PE

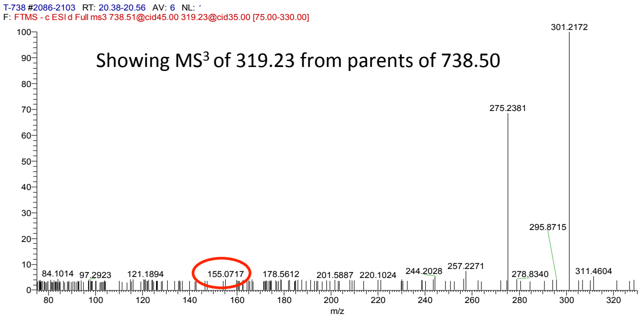

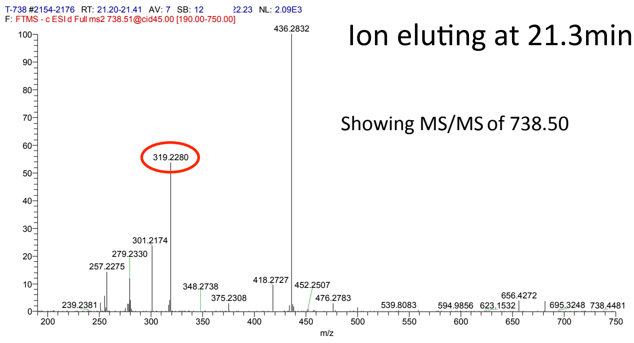

Ion eluting at 21.3min, 16:0p/HETE-PE

Showing MS/MS of 738.50

Structural identification of m/z 754.5027 as 16:0p/20:4(2O)-PE isomers

Four separate isomers are seen eluting between 16-20 min

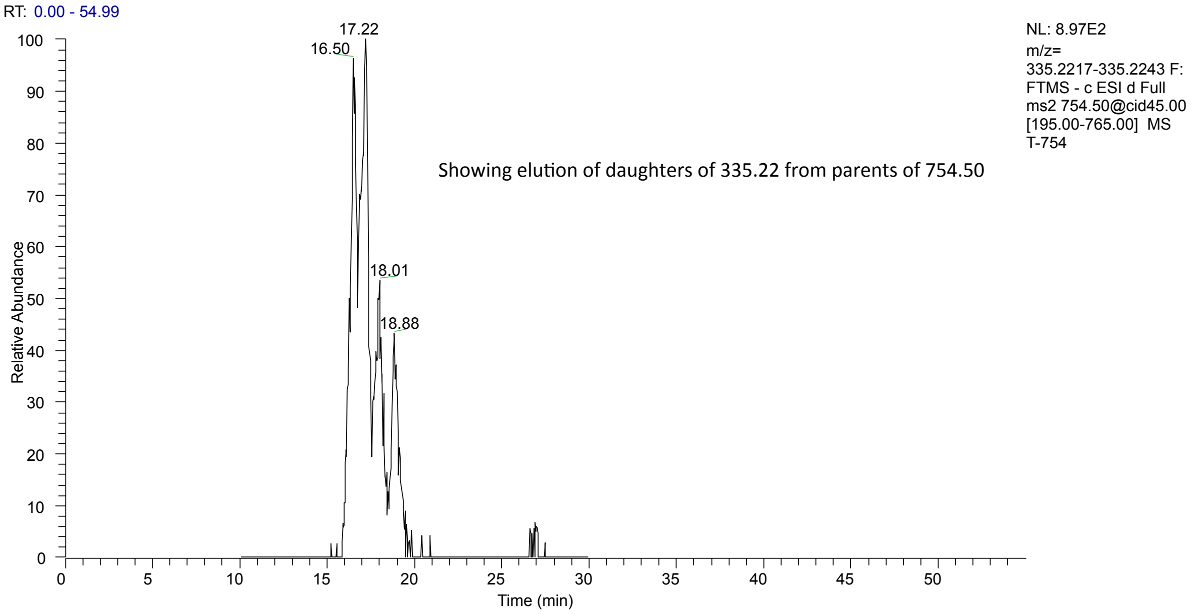

Ion eluting at 16.5 min, 16:0p/20:4(2O)-PE

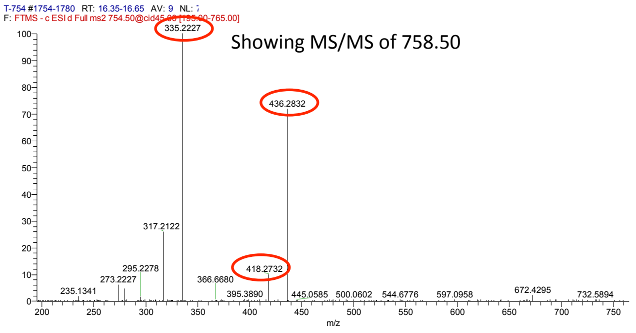

Ion eluting at 17.2 min, 16:0p/20:4(2O)-PE

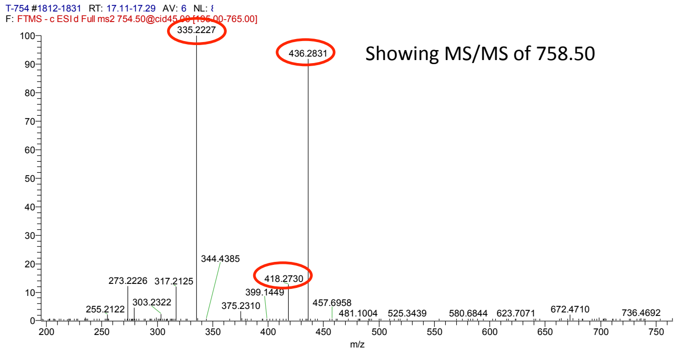

Ion eluting at 18.1 min, 16:0p/20:4(2O)-PE

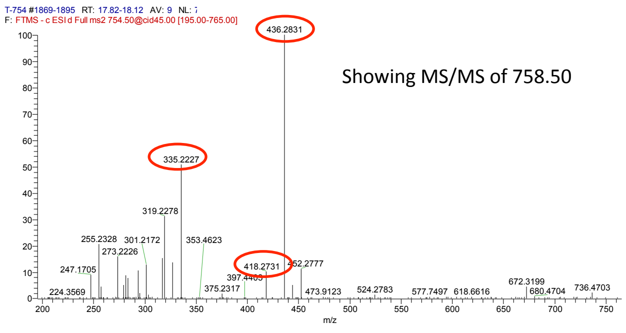

Ion eluting at 18.9 min, 16:0p/20:4(2O)-PE

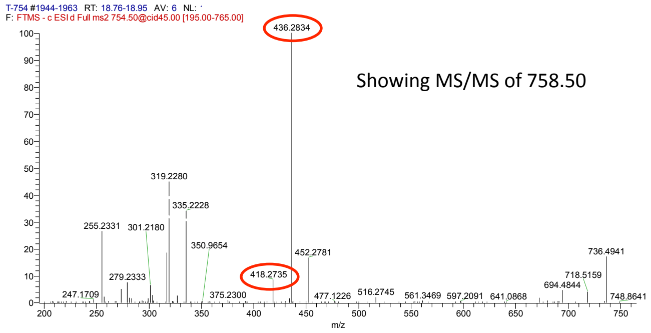

Structural identification of m/z 754.5027 as 16:0a/HETE-PE isomers

Two separate isomers are seen eluting between 17-19 min

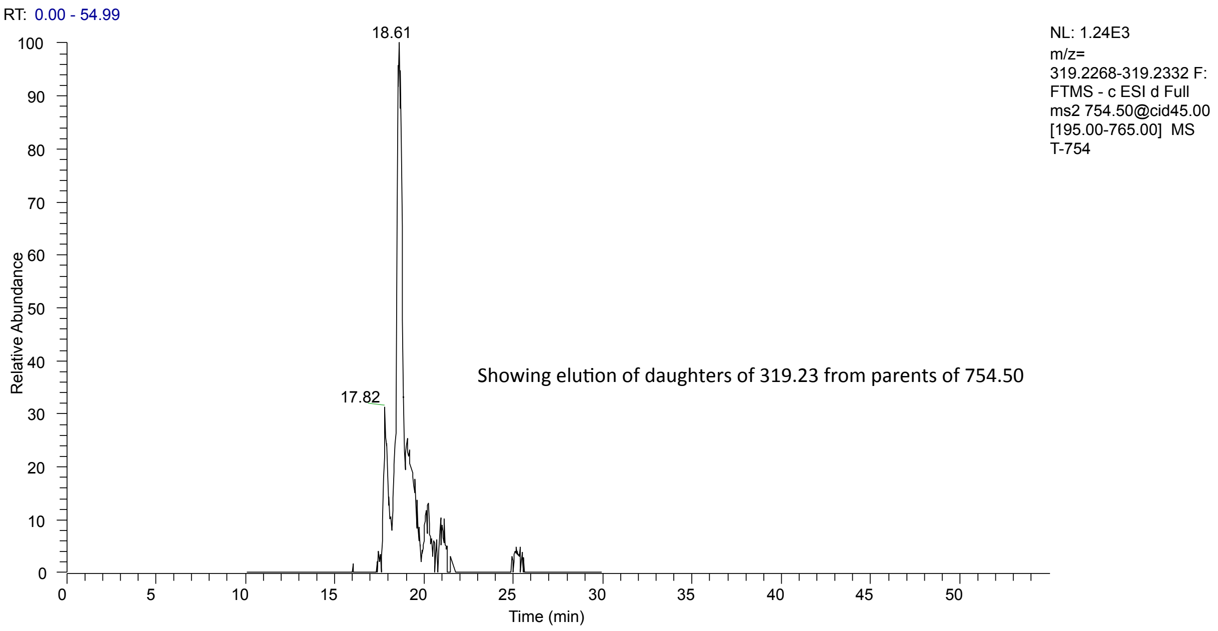

Ion eluting at 17.8 min, 16:0a/HETE-PE

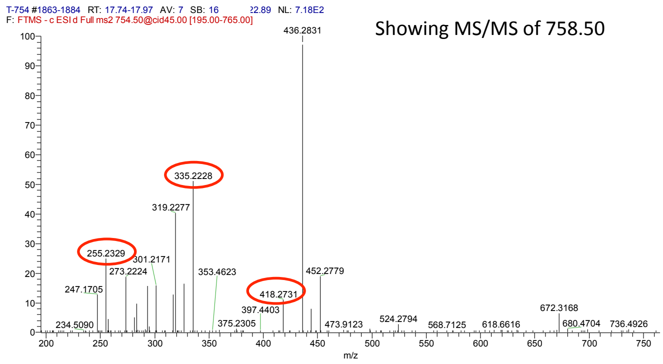

Ion eluting at 18.6 min, 16:0a/HETE-PE

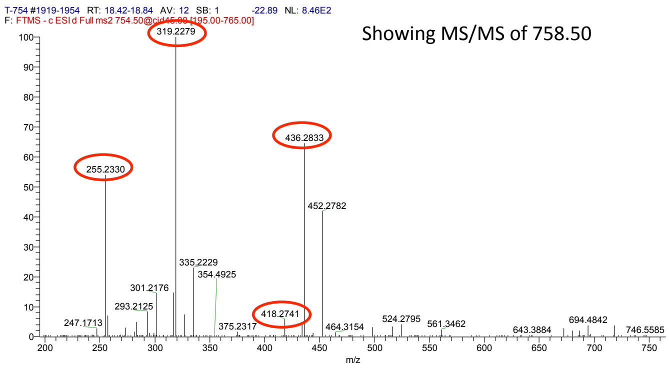

Ion eluting at 18.6 min, 16:0a/12-HETE-PE

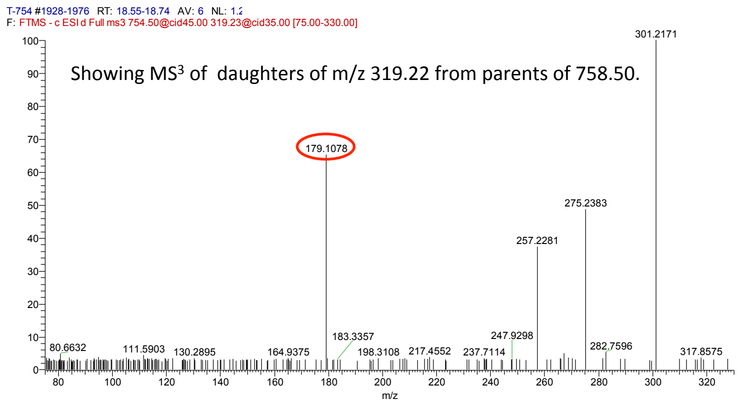

Structural identification of m/z 762.508 as 18:2p/HETE-PE

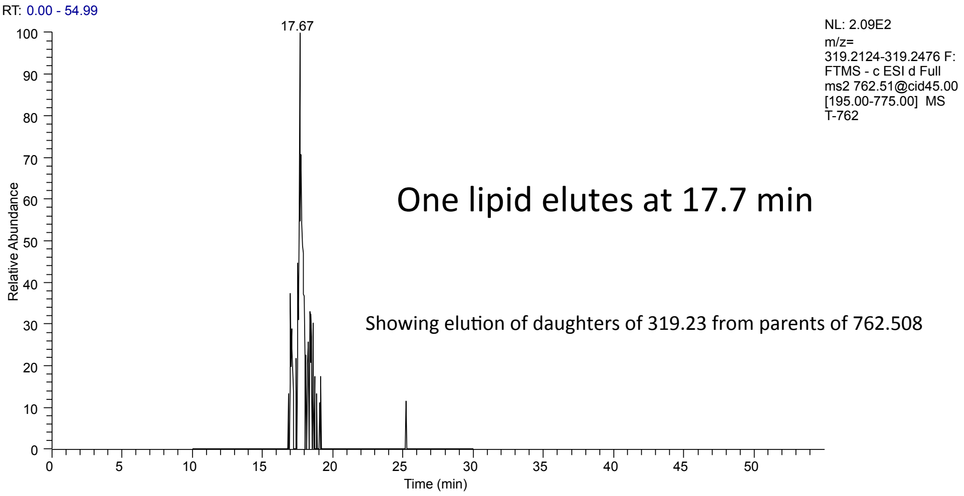

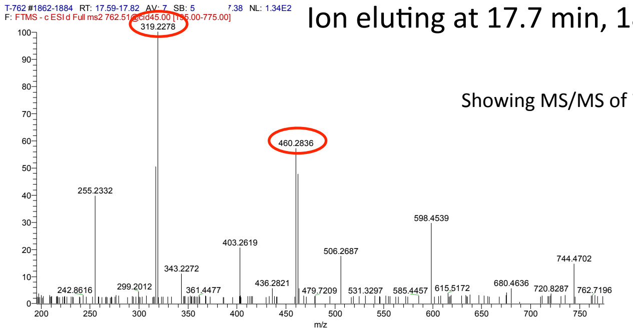

Structural identification of m/z 762.508 as 18:1p/20:5(O)-PE

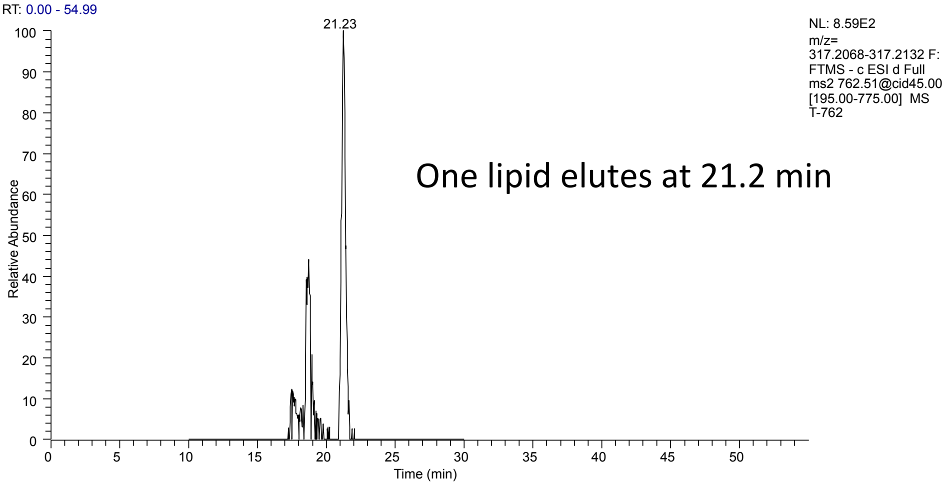

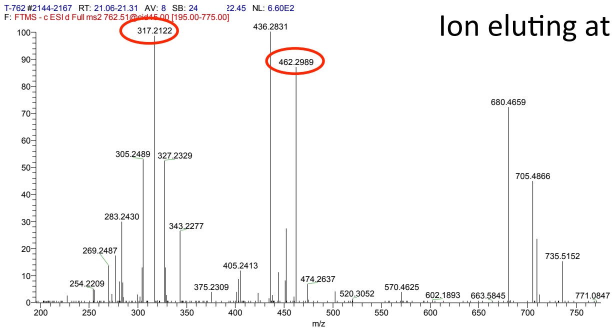

Structural identification of m/z 762.508 as 16:0p/HDoHE-PE isomers

Four separate isomers are seen eluting between 18-22 min

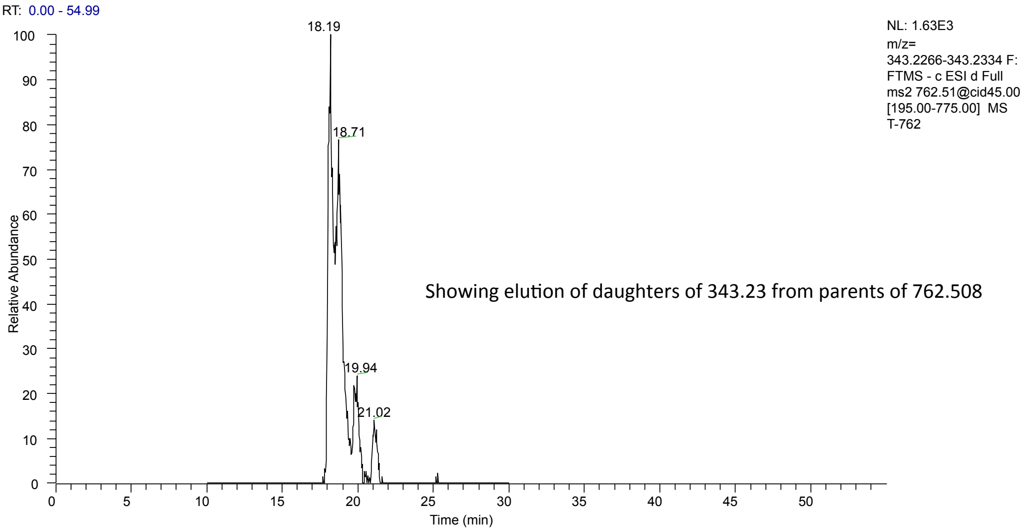

Ion eluting at 18.19 min, 16:0p/HDoHE-PE

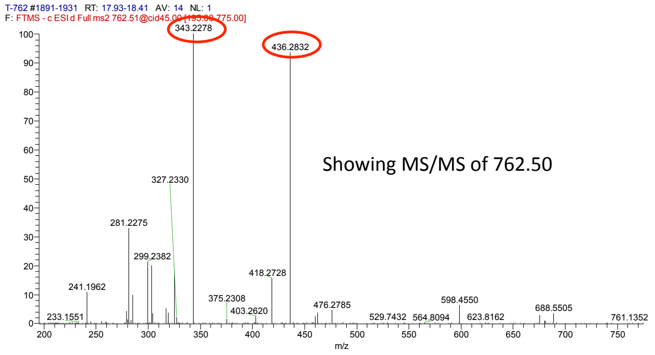

Ion eluting at 18.7 min, 16:0p/HDoHE-PE

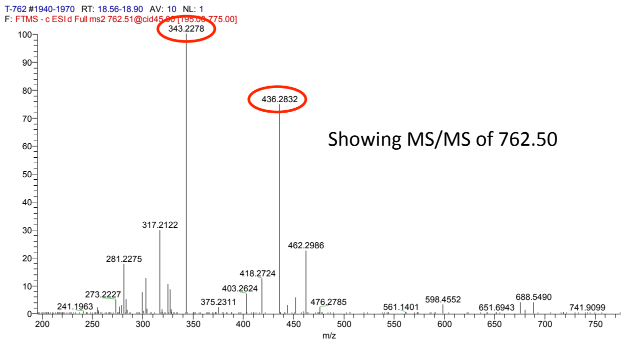

Ion eluting at 19.9 min, 16:0p/HDoHE-PE

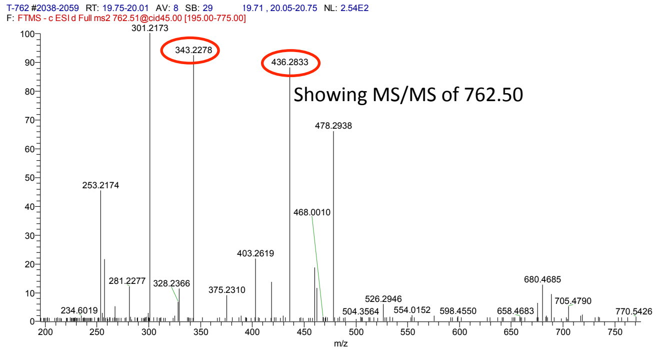

Ion eluting at 21.0 min, 16:0p/HDoHE-PE

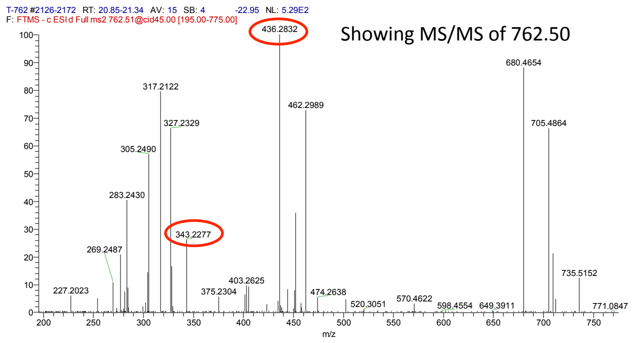

Structural identification of m/z 764.5238 as 16:0p/22:5(O)-PE

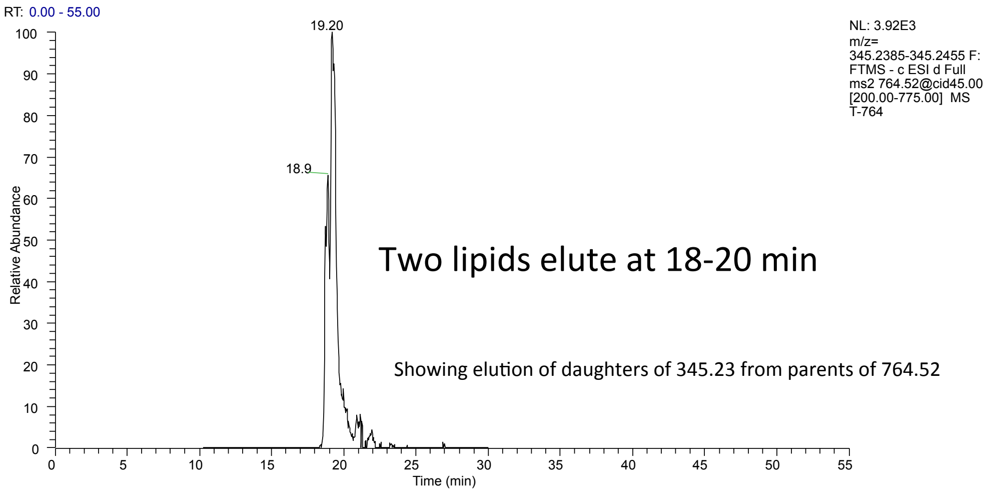

Ion eluting at 18.9 min, 16:0p/22:5(O)-PE

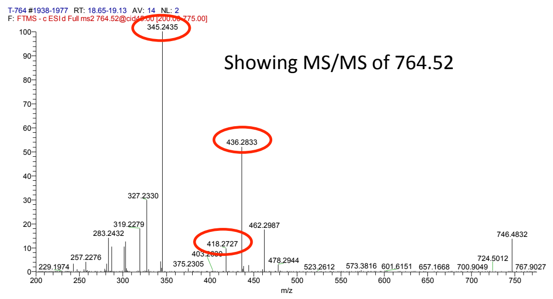

Ion eluting at 19.2 min, 16:0p/22:5(O)-PE

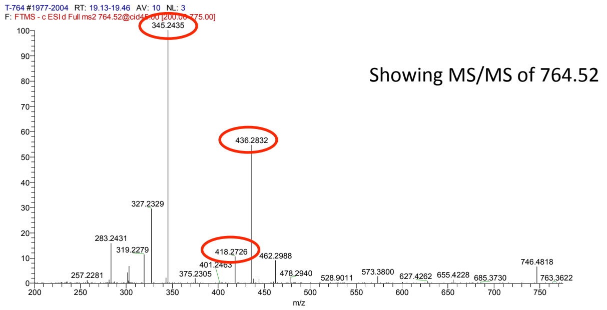

Structural identification of m/z 764.5238 as 18:1p/12-HETE-PE

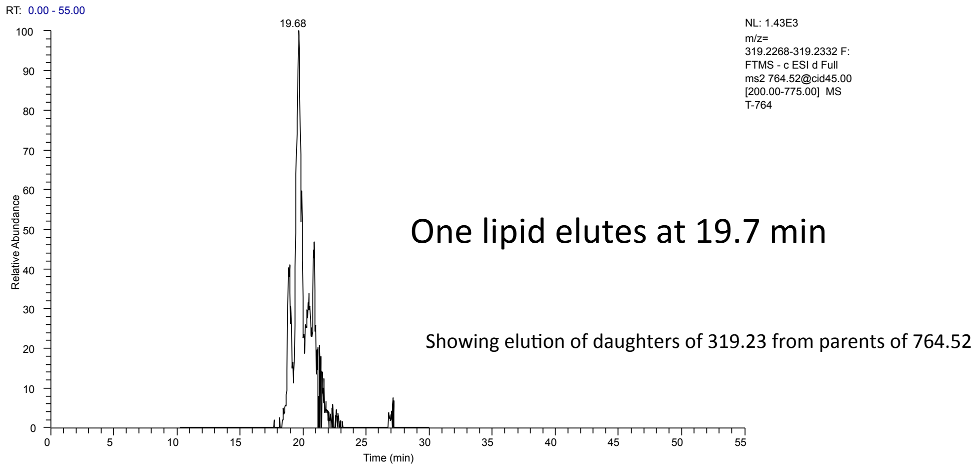

Ion eluting at 19.7 min, 18:1p/12-HETE-PE

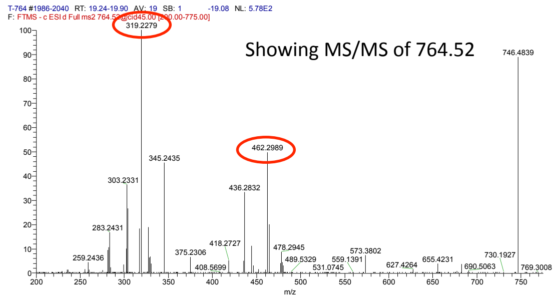

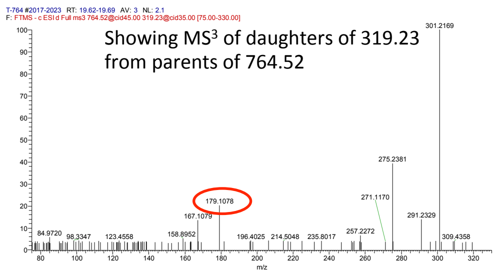

Structural identification of m/z 764.5238 as 16:0p/22:5(O)-PE

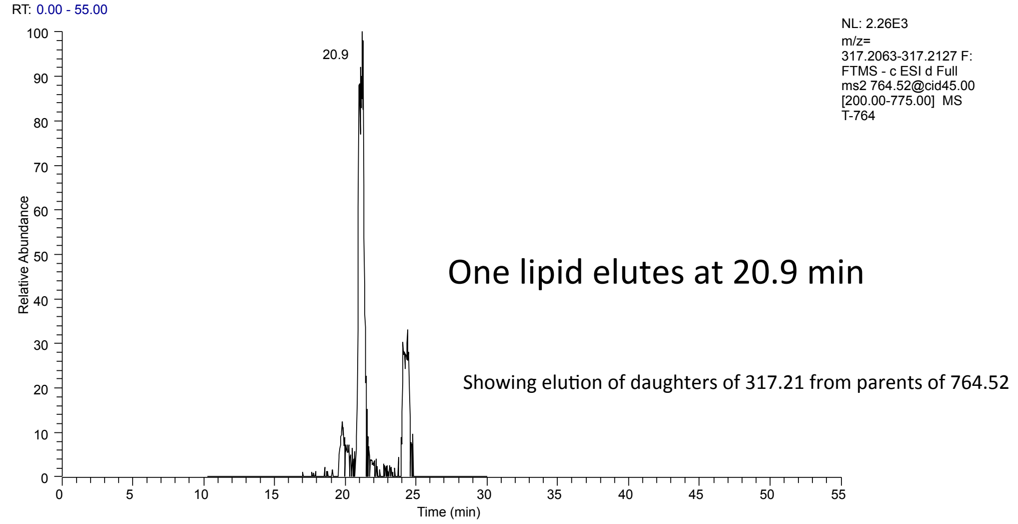

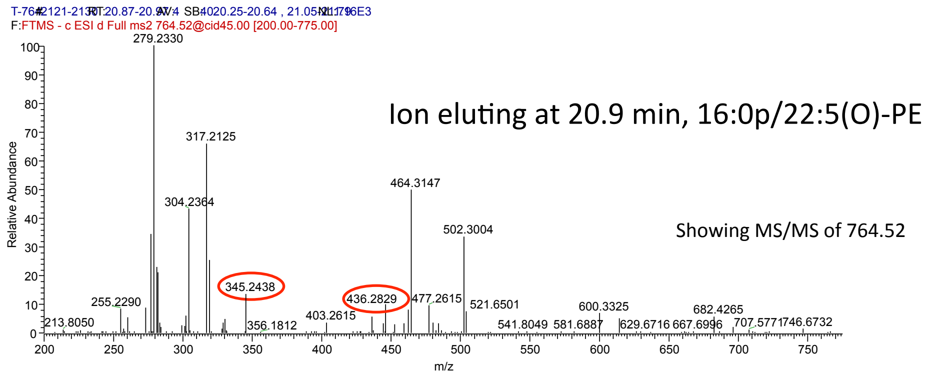

Structural identification of m/z 766.5392 as 16:0p/22:4(O)-PE

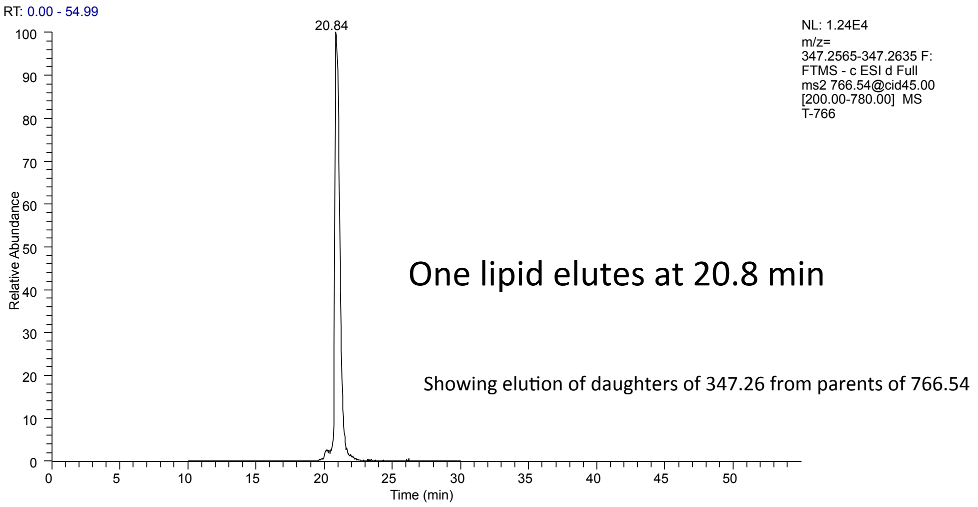

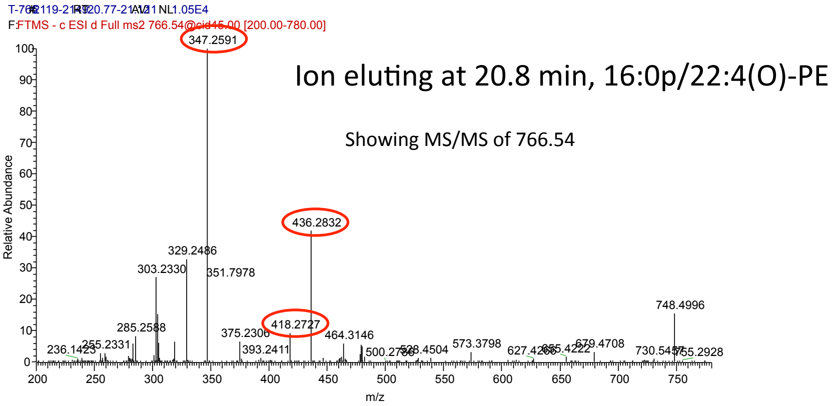

Structural identification of m/z 766.5392 as 18:0p/HETE-PE

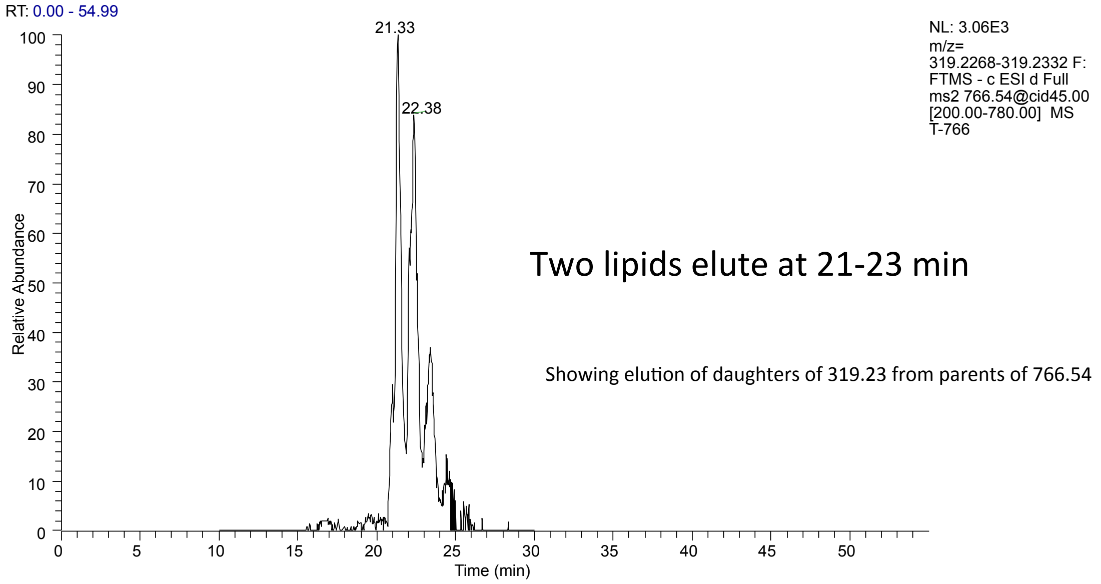

Ion eluting at 21.3 min, 18:0p/15-HETE-PE

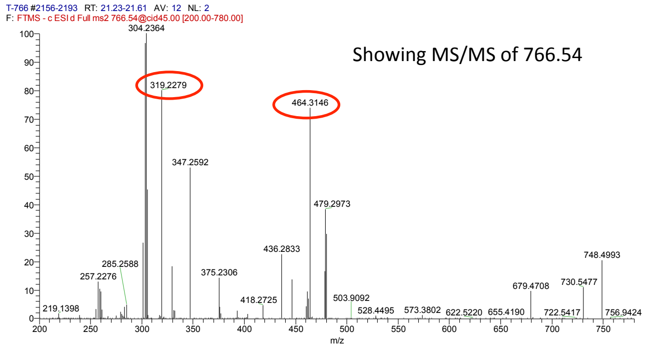

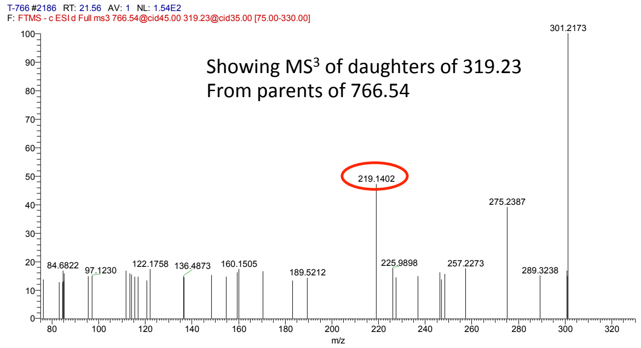

Ion eluting at 22.4 min, 18:0p/12-HETE-PE

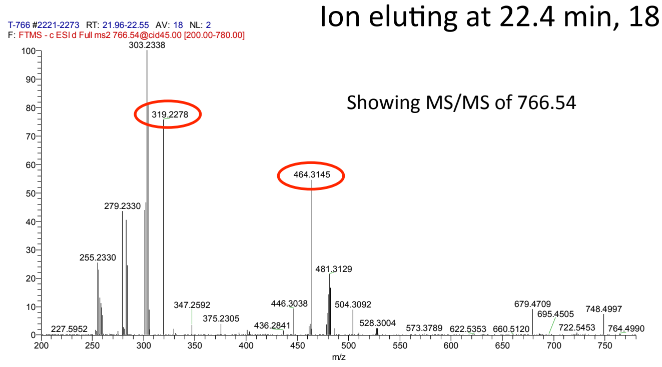

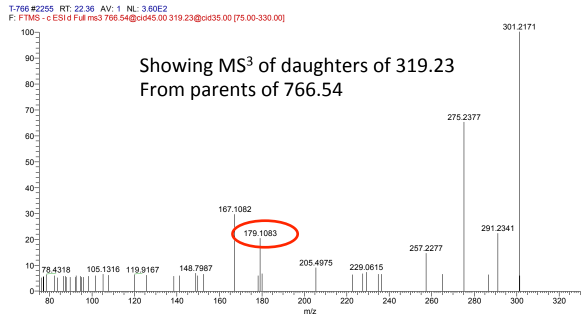

Structural identification of m/z 766.5392 as 16:0e/22:5(O)-PE

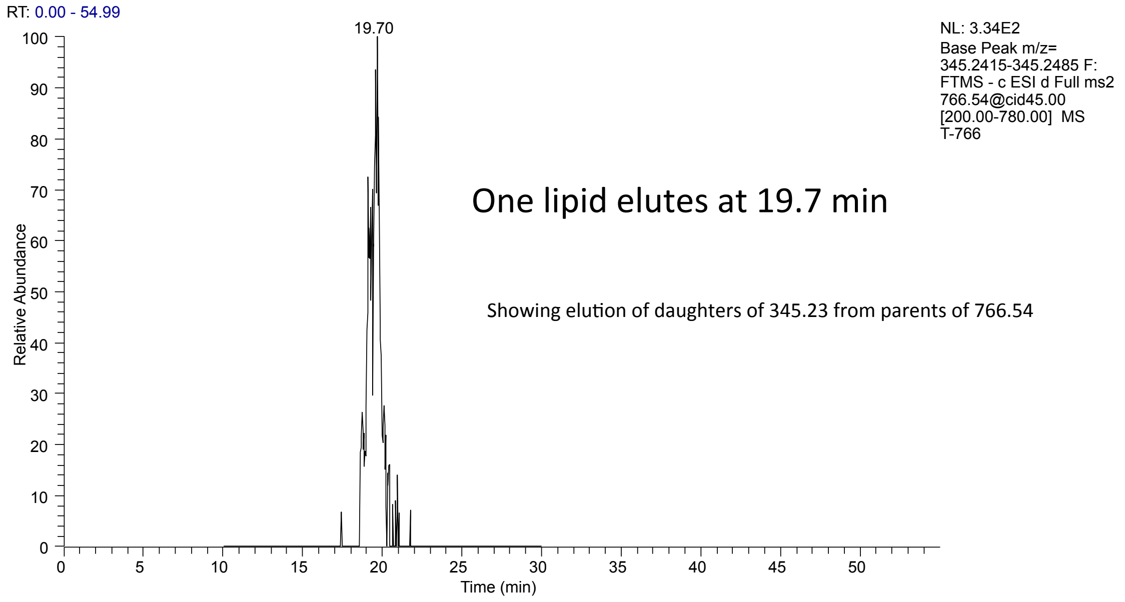

Ion eluting at 19.7min, 16:0e/22:5(O)-PE

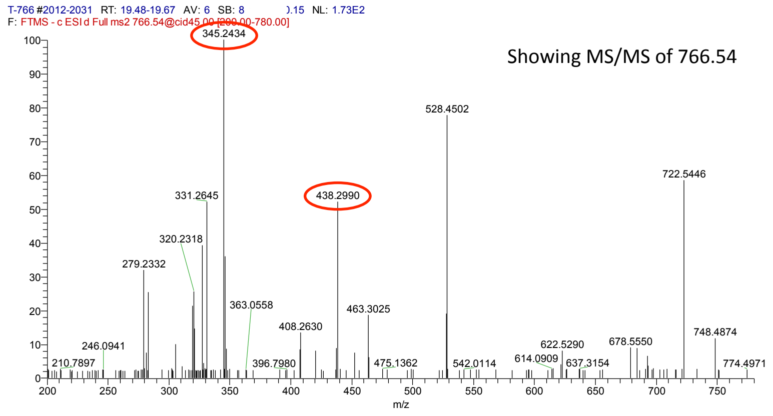

Structural identification of m/z 770.4978 as 16:0p/20:4(3O)-PE, Including esterified PGE2/D2 and DXA3

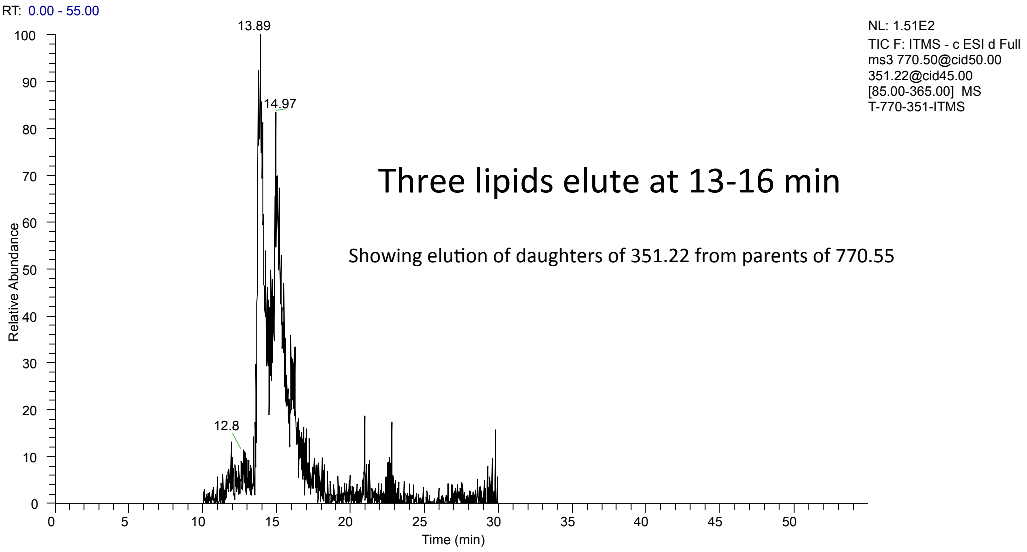

Ion eluting at 12.8 min, 16:0p/20:4(3O)-PE

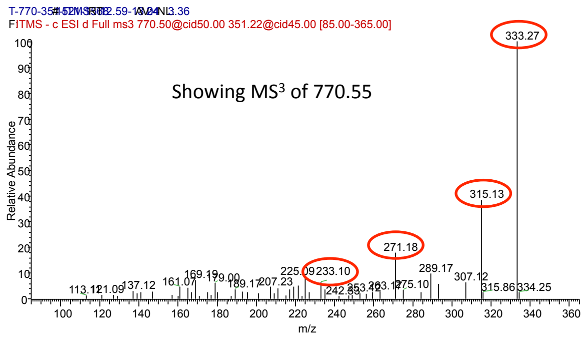

Ion eluting at 13.9 min, 16:0p/20:4(3O)-PE

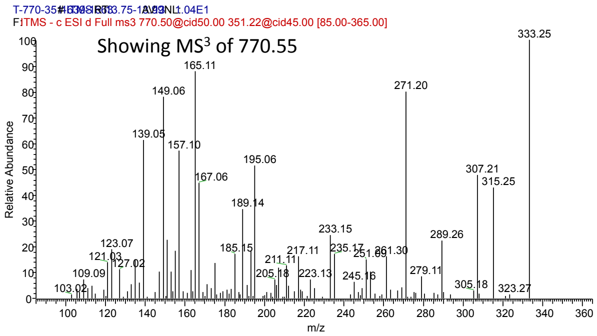

Ion eluting at 15.0 min, 16:0p/DXA3-PE

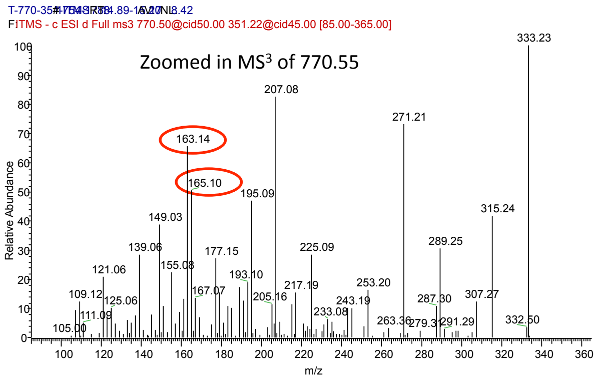

Ion eluting at 16.1 min, 16:0p/20:4(3O)-PE

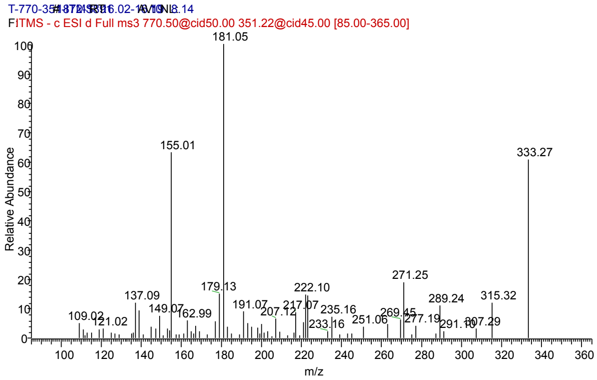

Ion eluting at 13.42 has largely fragmented during MS/MS and does not give a strong signal, but has been previously identified using MS (Aldrovandi et al JLR, 2013). Ions characteristic of PGE2/D2 are seen in the MS3 of this lipid. The ion at 13.8 is not identified, the ion at 15.0 has been identified as DXA3.

Structural identification of m/z 770.4978 as 16:0a/20:4(2O)-PE

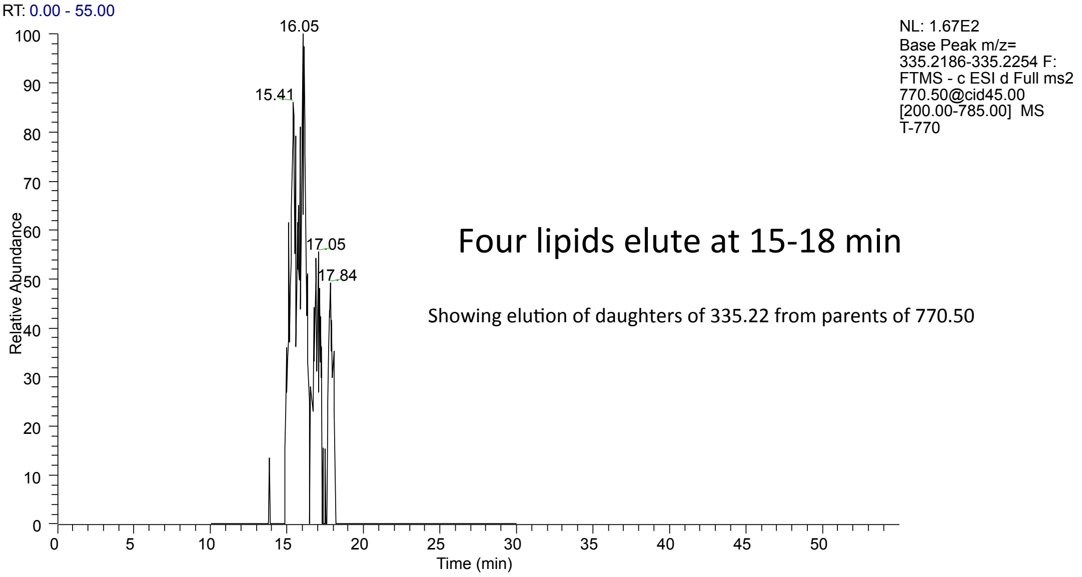

Ion eluting at 15.4 min, 16:0a/20:4(2O)-PE

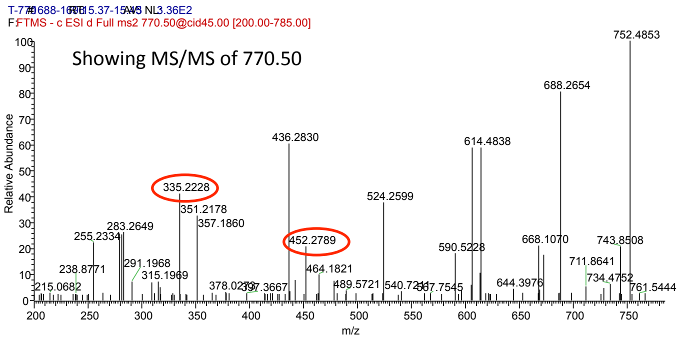

Ion eluting at 16.05 min, 16:0a/20:4(2O)-PE

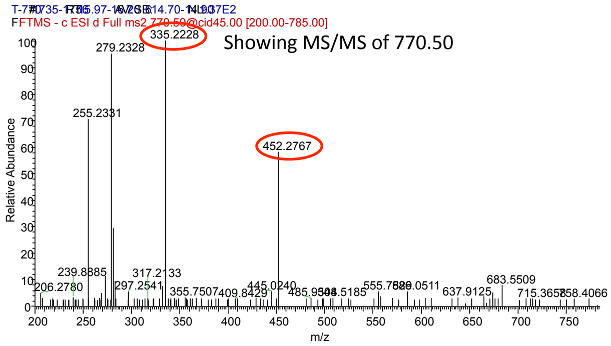

Ion eluting at 17.0 min, 16:0a/20:4(2O)-PE

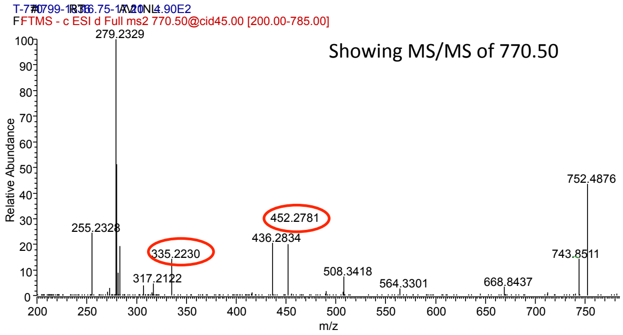

Ion eluting at 17.8 min, 16:0a/20:4(2O)-PE

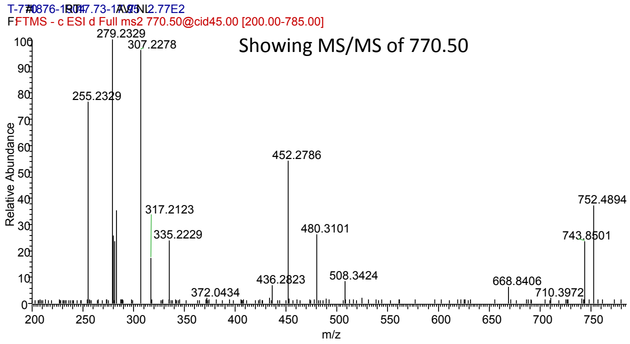

Structural identification of m/z 778.5030 as 16:0p/22:6(2O)-PE,

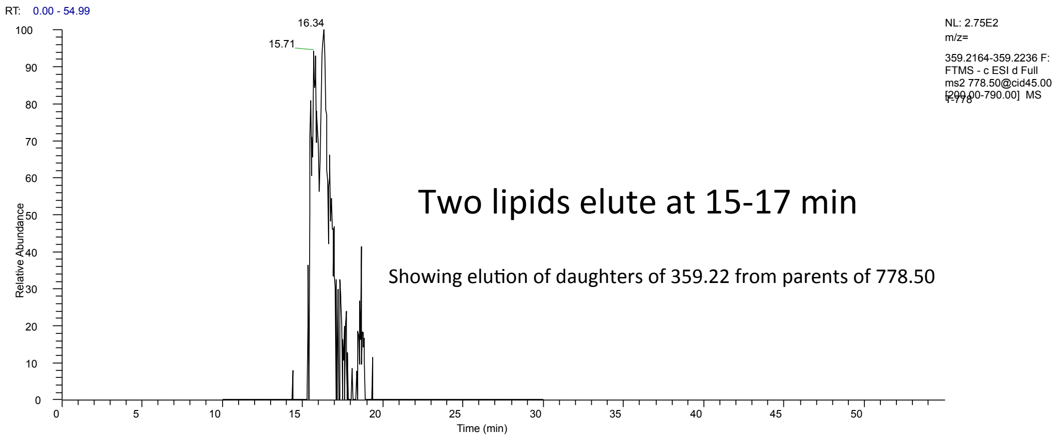

Ion eluting at 15.7 min, 16:0p/22:6(2O)-PE

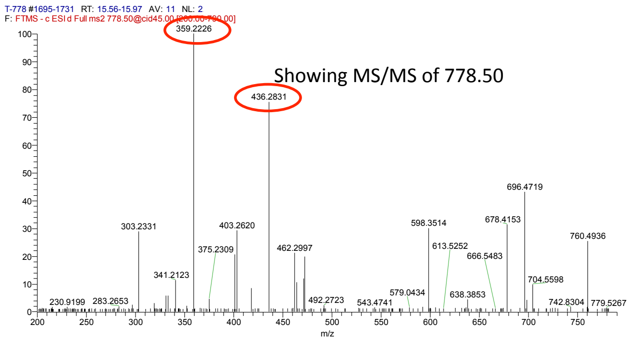

Ion eluting at 16.3 min, 16:0p/22:6(2O)-PE

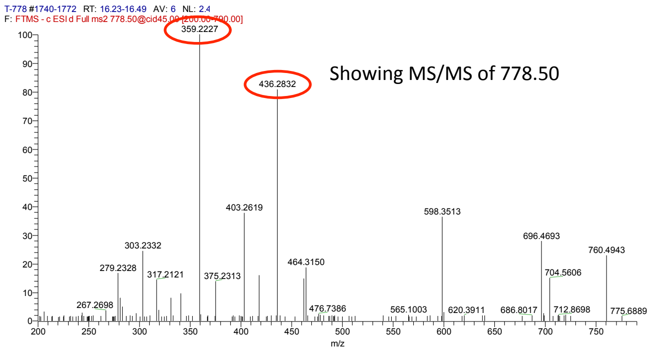

Ion eluting at 17.7 min, 16:0p/22:6(2O)-PE

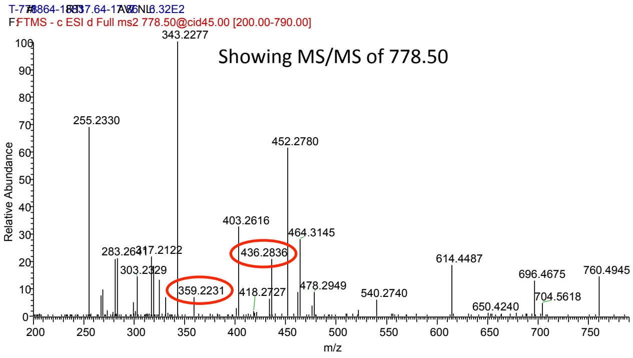

Ion eluting at 18.7 min, 16:0p/22:6(2O)-PE

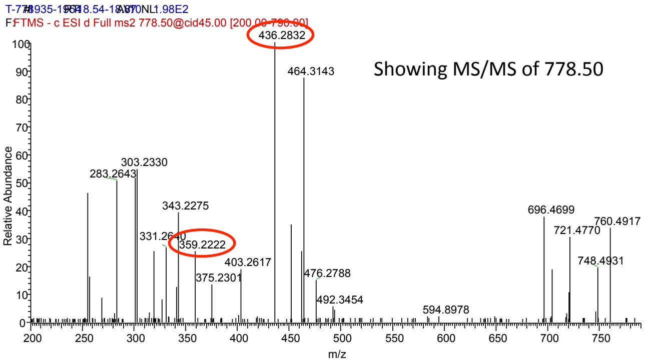

Structural identification of m/z 778.5030 as 18:2a/HETE-PE,

Three lipids elute at 16-19 min

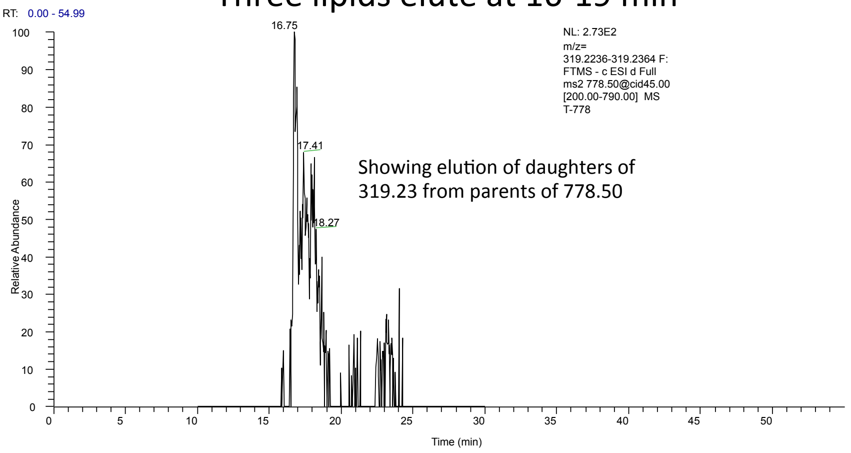

Ion eluting at 16.8 min, 18:2a/HETE-PE

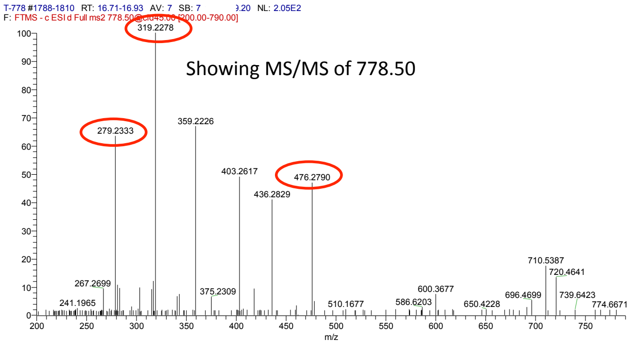

Ion eluting at 17.5 min, 18:2a/HETE-PE

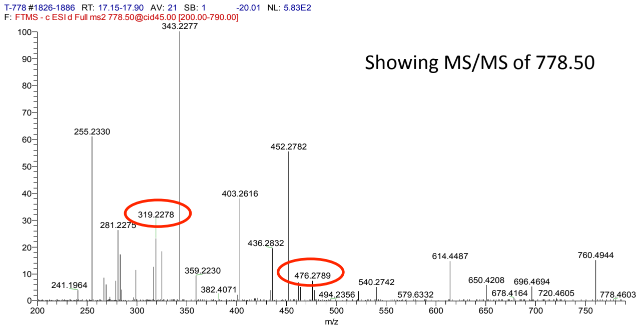

Ion eluting at 18.1 min, 18:2a/HETE-PE

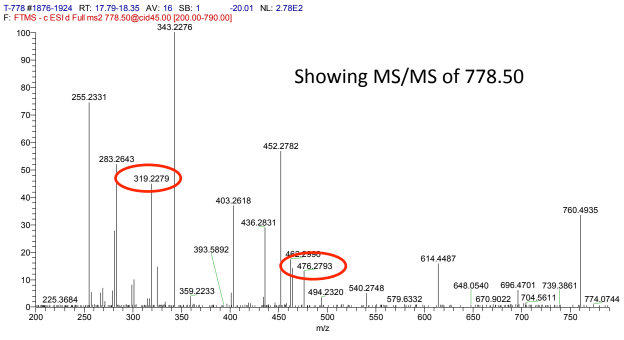

Structural identification of m/z 778.503 as 16:0a/HDoHE-PE

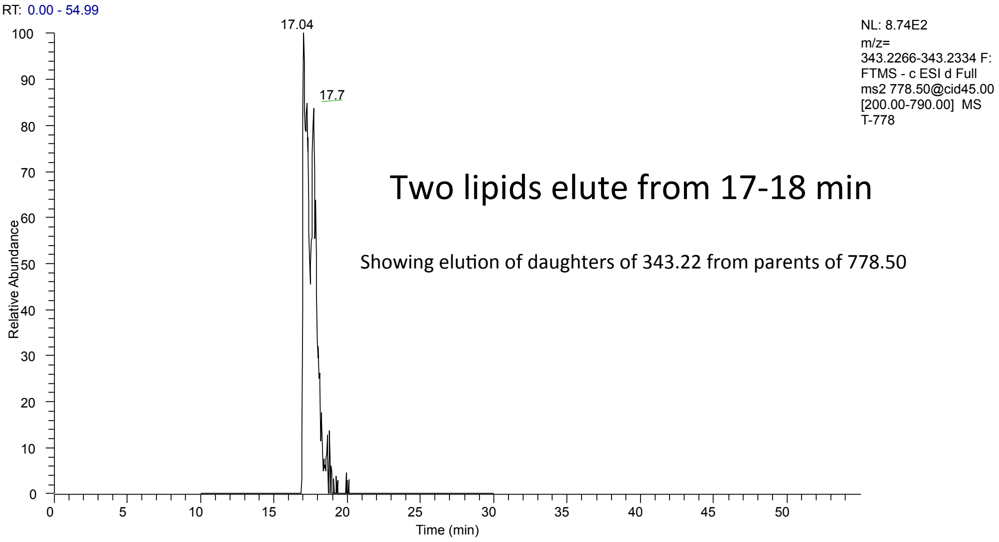

Two lipids elute from 17-18 min

Showing elution of daughters of 343.22 from parents of 778.50

Ion eluting at 17 min, 16:0a/HDoHE-PE

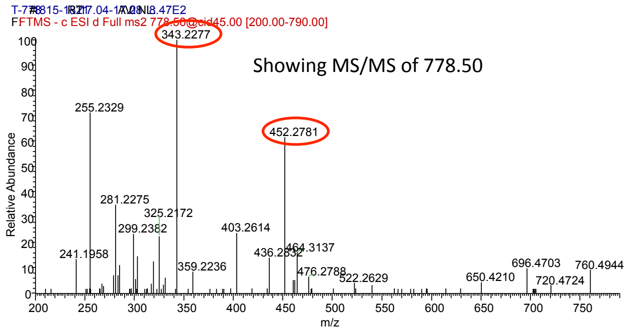

Showing MS/MS of 778.50

Ion eluting at 17.7 min, 16:0a/HDoHE-PE

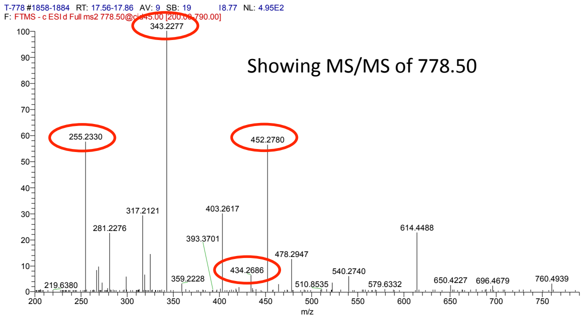

Showing MS/MS of 778.50

Structural identification of m/z 778.503 as 18:1a/20:5(O)-PE

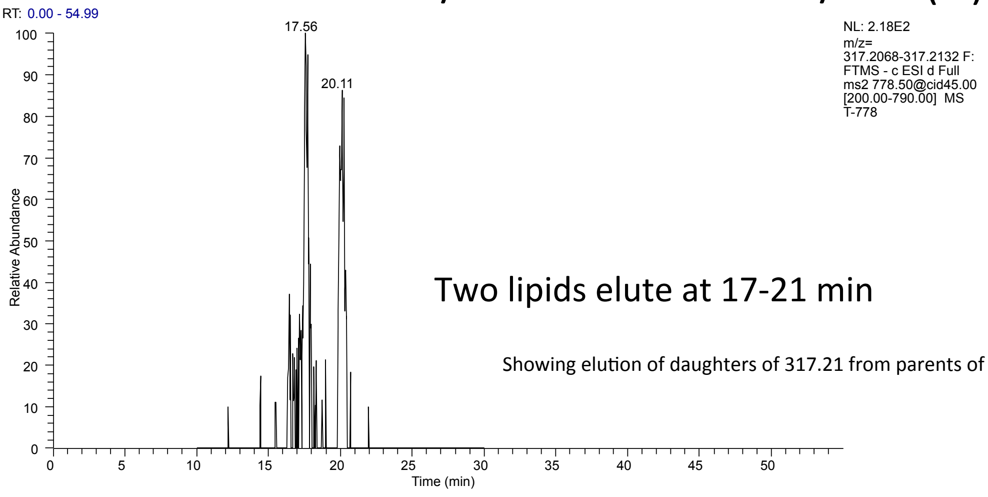

Two lipids elute at 17-21 min

Showing elution of daughters of 317.21 from parents of 778.50

Ion eluting at 17.5 min, 18:1a/20:5(O)-PE

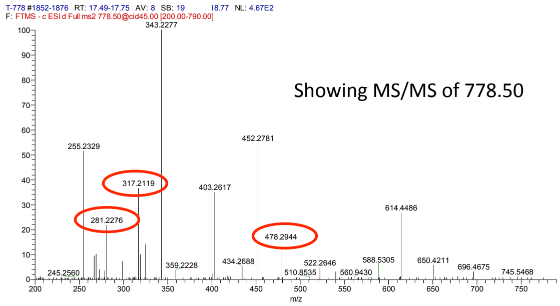

Showing MS/MS of 778.50

Ion eluting at 20.1 min, 18:1a/20:5(O)-PE

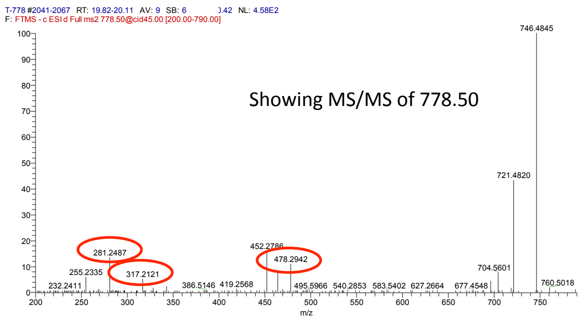

Showing MS/MS of 778.50

Structural identification of m/z 780.5186 as 18:1p/20:4(2O)-PE

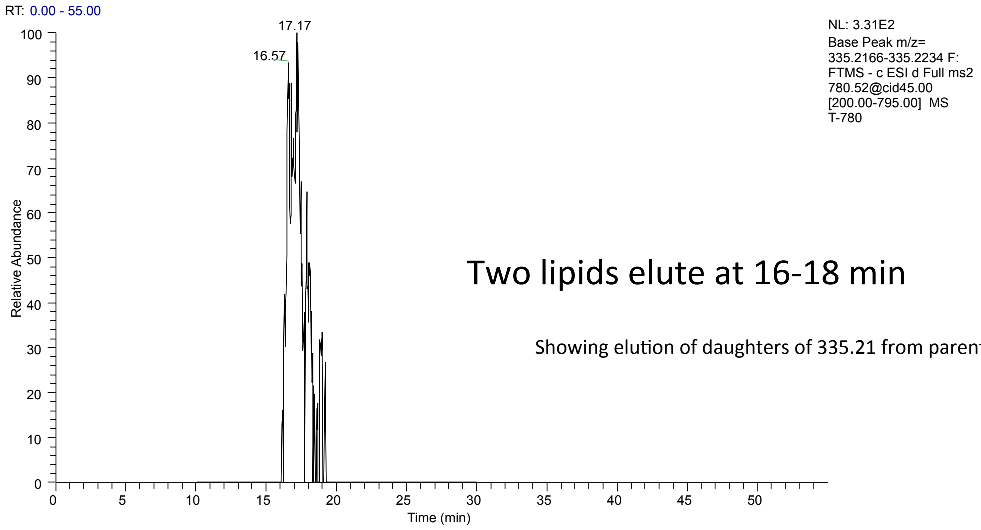

Ion eluting at 16.6 min, 18:1p/20:4(2O)-PE

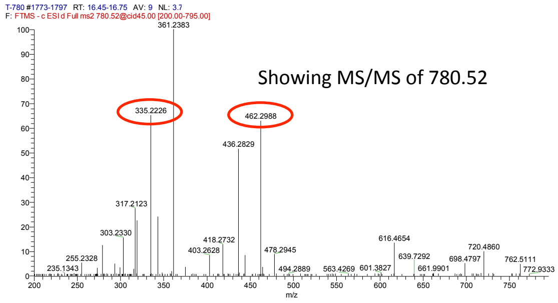

Ion eluting at 17.2 min, 18:1p/20:4(2O)-PE

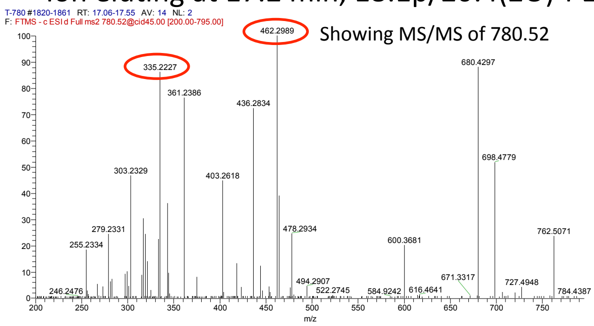

Structural identification of m/z 780.5186 as 16:0p/22:5(2O)-PE

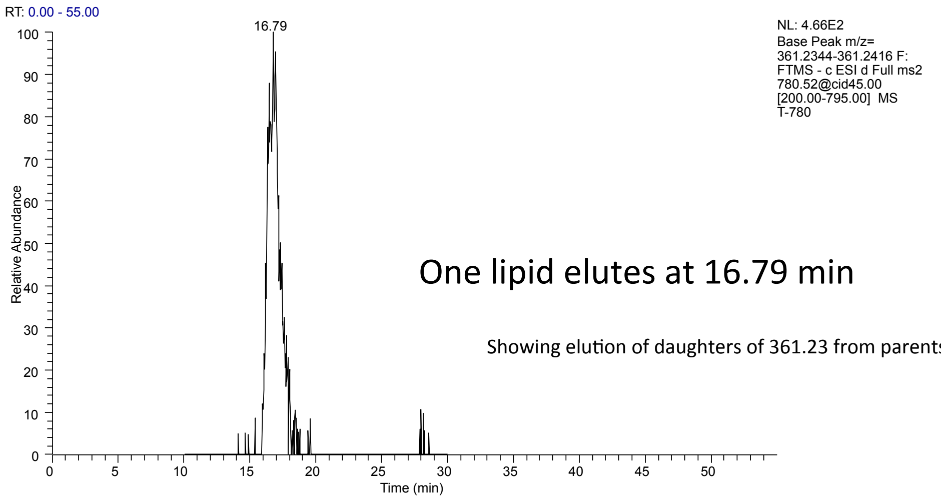

Ion eluting at 16.8 min, 16:0p/22:5(2O)-PE

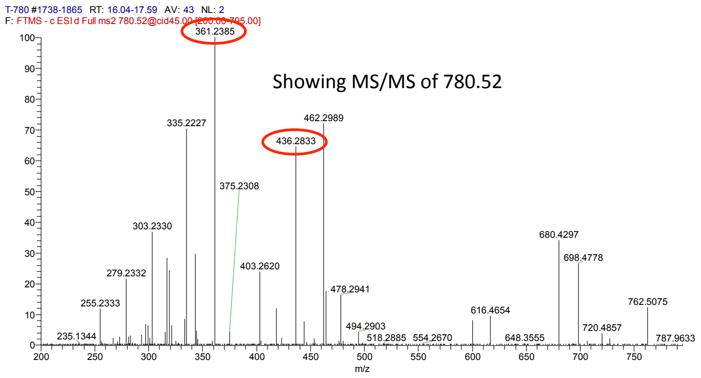

Structural identification of m/z 780.5186 as 16:0a/22:5(O)-PE

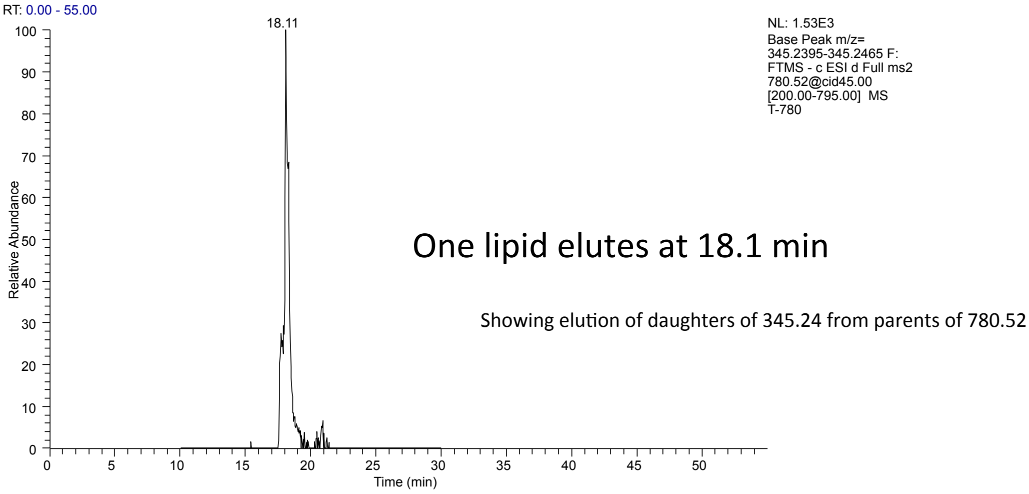

Ion eluting at 18.1 min, 16:0a/22:5(O)-PE

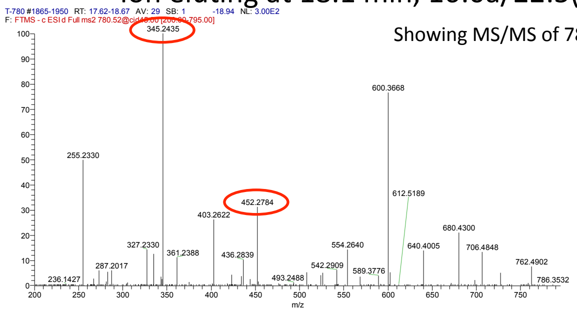

Structural identification of m/z 780.5186 as 18:1a/12-HETE-PE

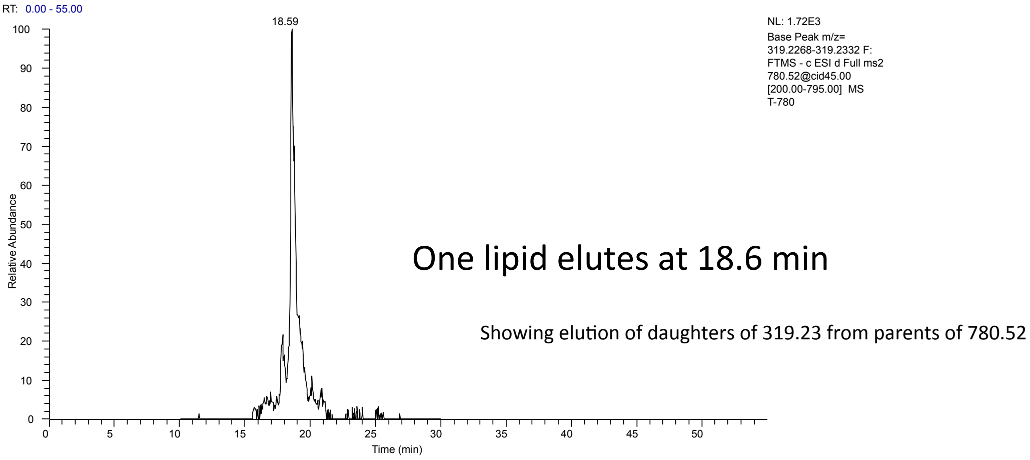

Ion eluting at 18.6 min, 18:1a/12-HETE-PE

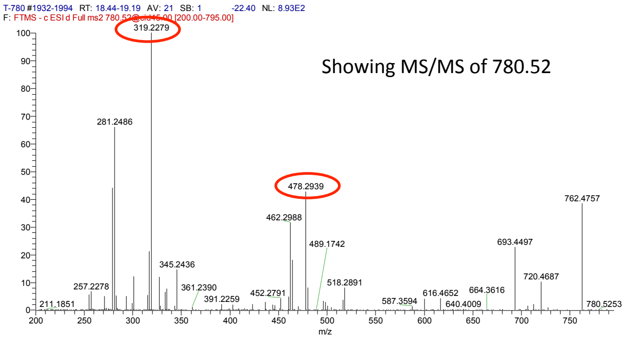

Ion eluting at 18.6 min, 18:1a/12-HETE-PE

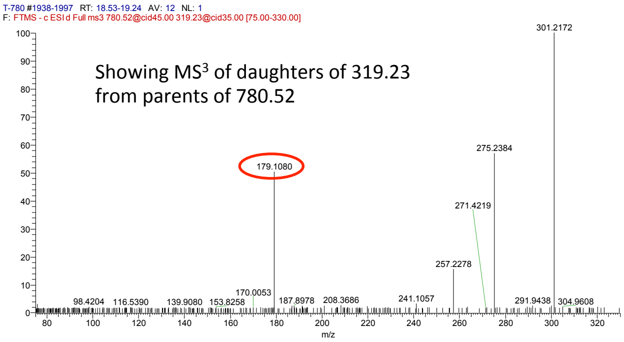

Structural identification of m/z 782.5341 as 18:0p/20:4(2O)-PE

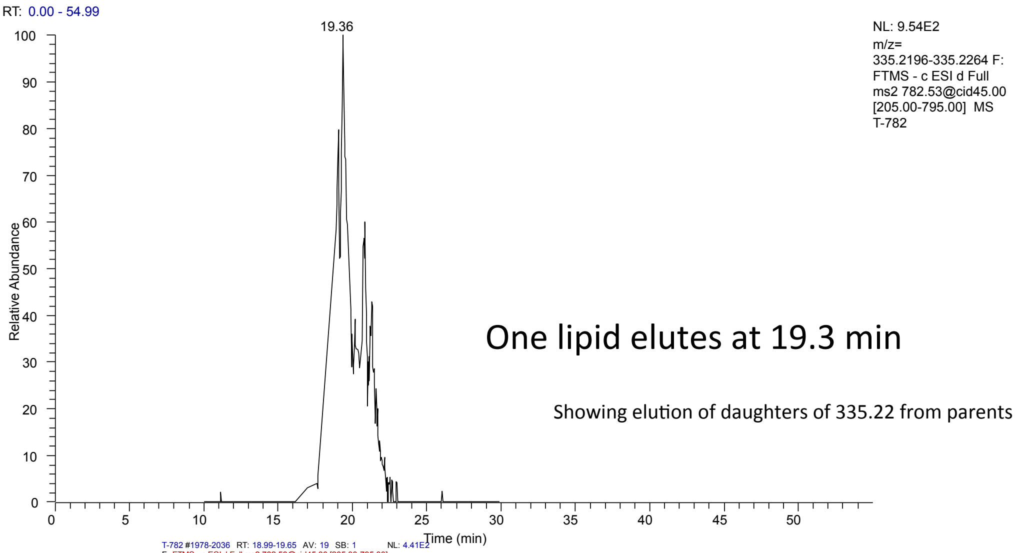

One lipid elutes at 19.3 min

Showing elution of daughters of 335.22 from parents of 782.53

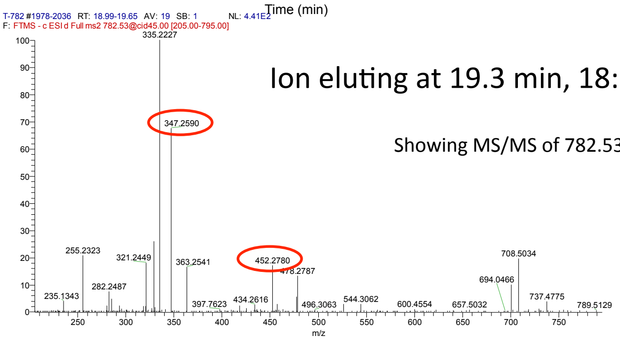

Ion eluting at 19.3 min, 18:0p/20:4(2O)-PE

Showing MS/MS of 782.53

Structural identification of m/z 782.5341 as 16:0a/12-HETE-PC or 18:0a/12-HETE-PE

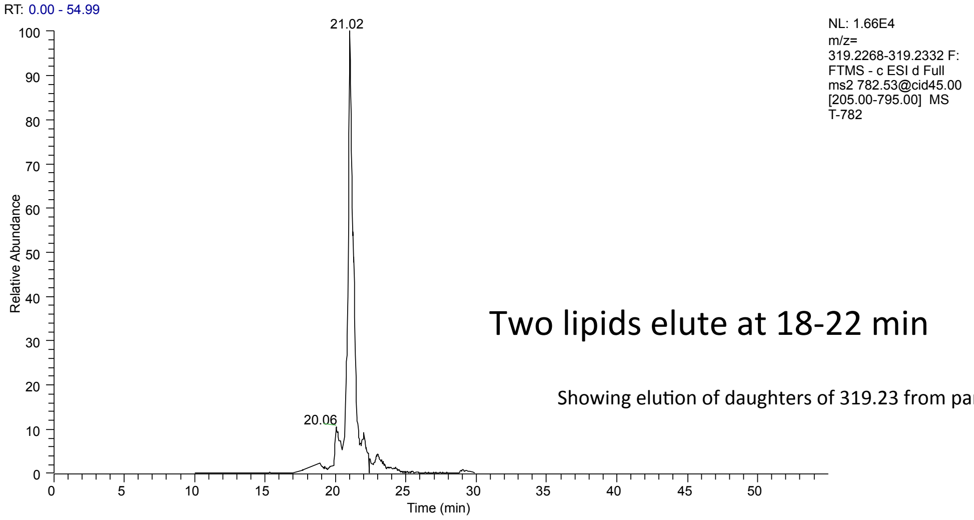

Two lipids elute at 18-22 min

Showing elution of daughters of 319.23 from parents of 782.53

Ion eluting at 20.0 min, 16:0a/12-HETE-PC

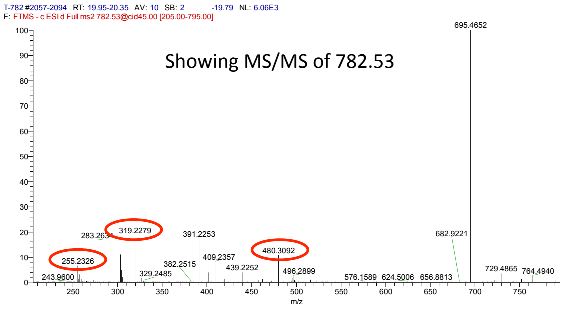

Ion eluting at 21.0 min, 18:0a/12-HETE-PE

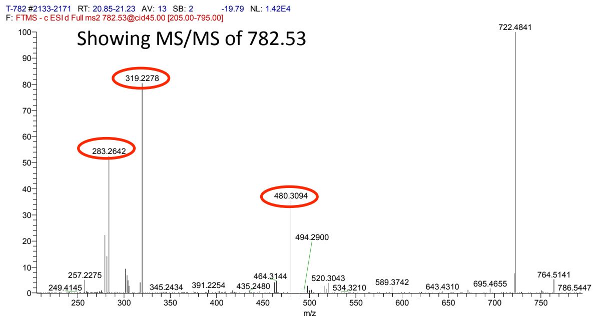

Showing MS³ of daughters of 319.23 at 20.06 min of m/z 782.53

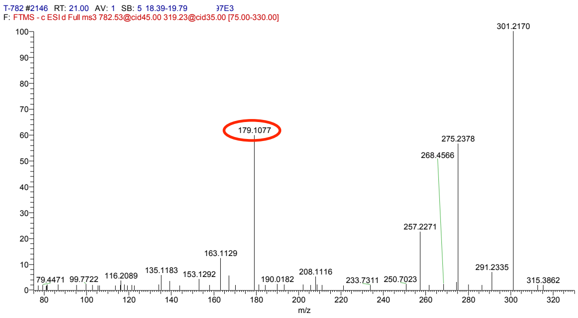

Showing MS³ of daughters of 319.23 at 20.06 min of m/z 782.53

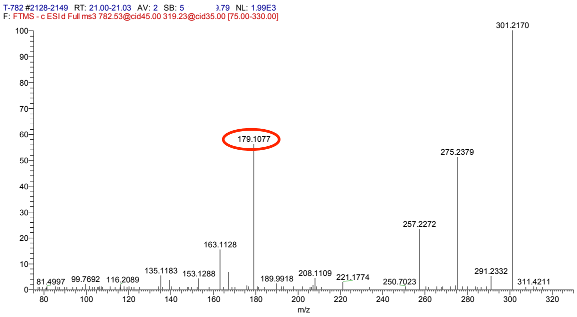

Structural identification of m/z 782.5341 as 16:0a/22:4(O)-PE

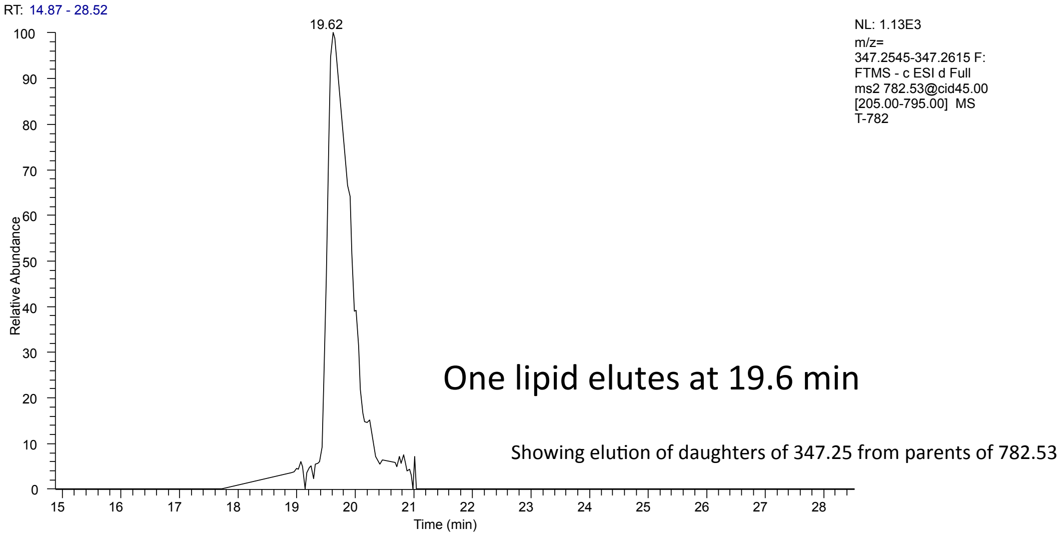

Ion eluting at 19.6 min, 16:0a/22:4(O)-PE

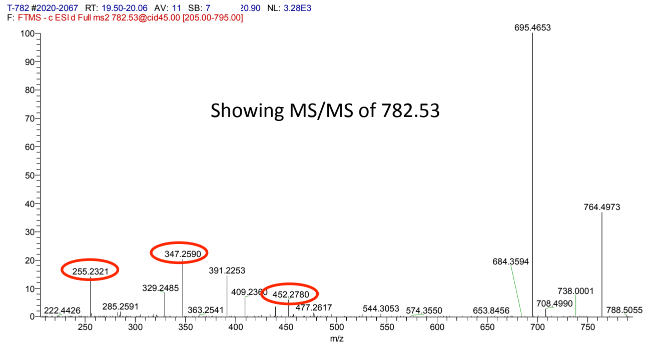

Structural identification of m/z 790.5392 as 18:1p/22:5(O)-PE

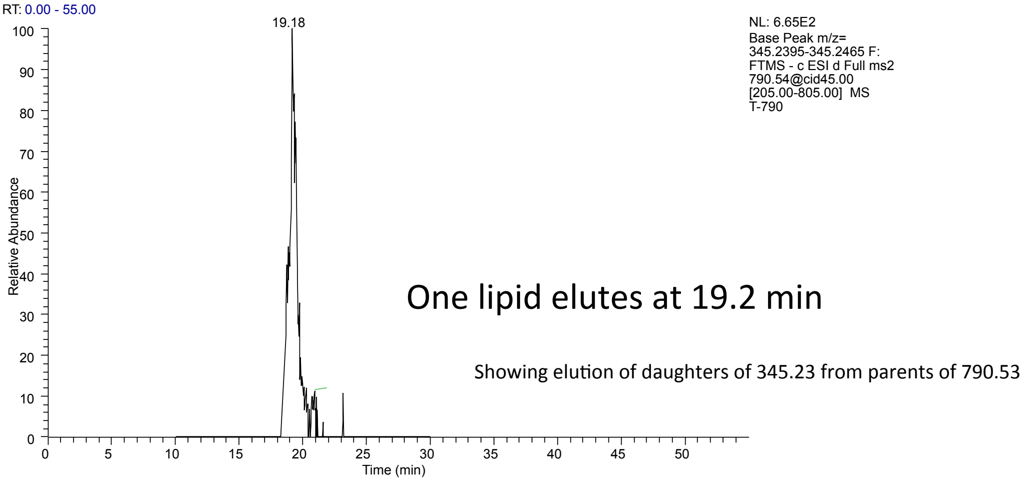

Ion eluting at 19.2 min, 18:1p/22:5(O)-PE

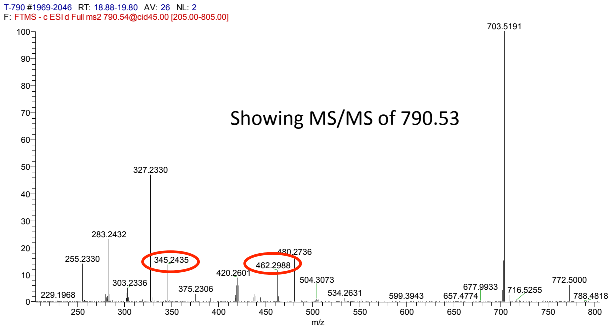

Structural identification of m/z 790.5392 as 18:0p/HDoHE-PE

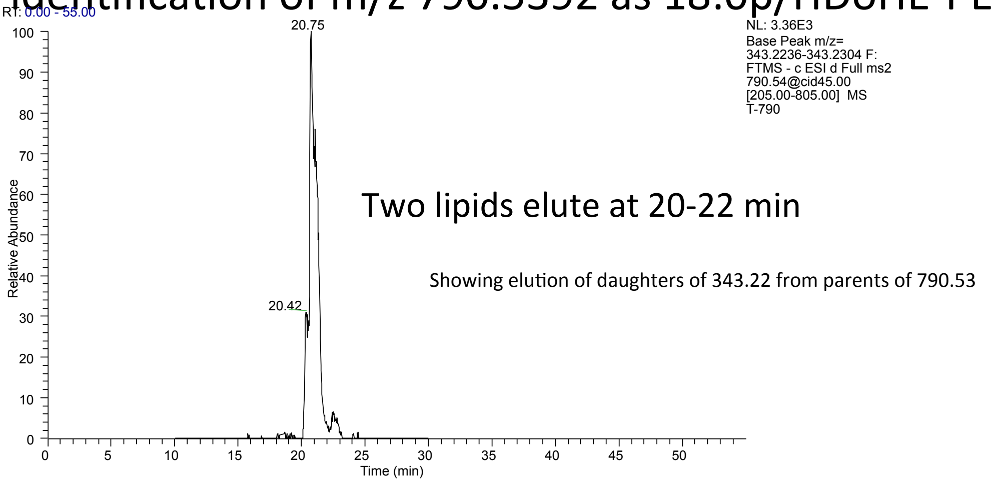

Ion eluting at 20.7 min, 18:0p/HDoHE-PE

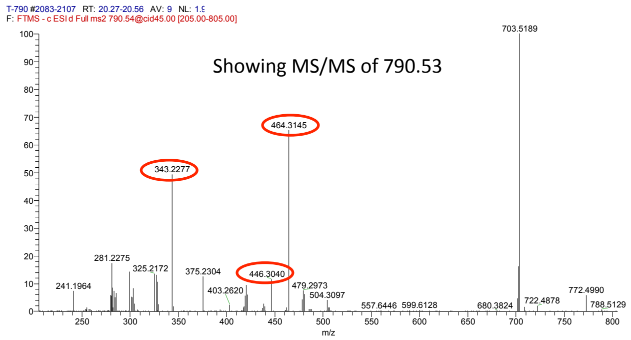

Ion eluting at 20.7 min, 18:0p/HDoHE-PE

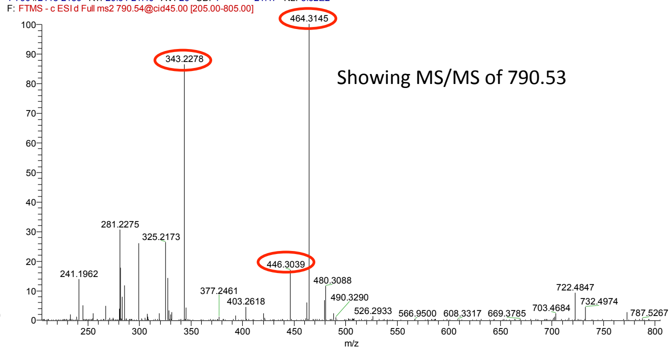

Structural identification of m/z 792.5547 as 18:1p/22:4(O)-PE

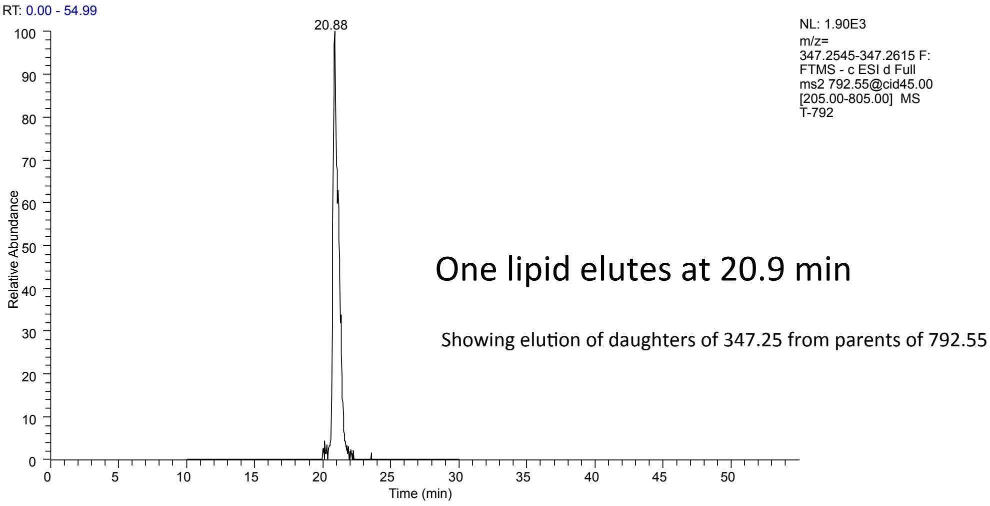

One lipid elutes at 20.9 min

Showing elution of daughters of 347.25 from parents of 792.55

Ion eluting at 20.9 min, 18:1p/22:4(O)-PE

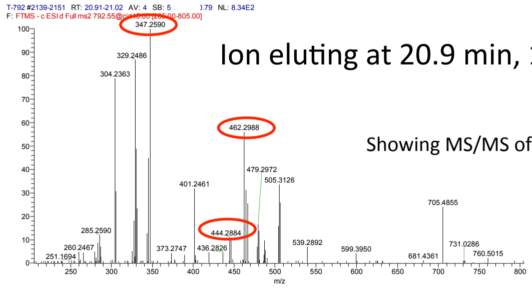

Showing MS/MS of 792.55

Structural identification of m/z 792.5547 as 18:0p/22:5(O)-PE

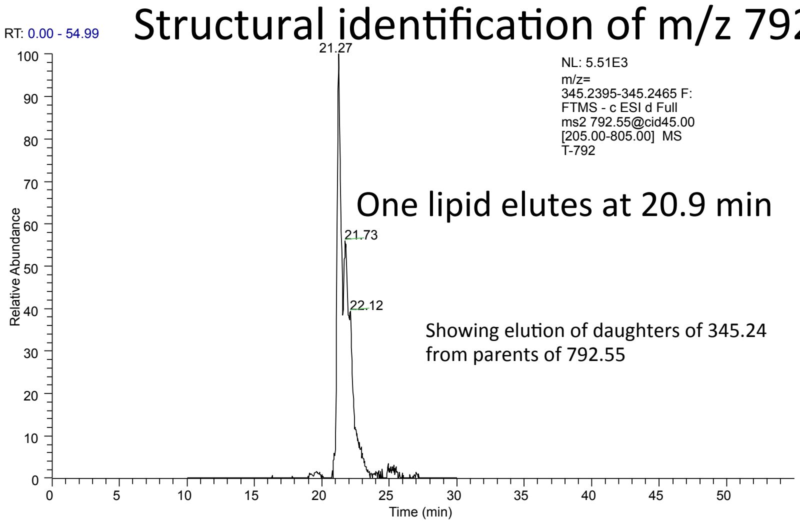

One lipid elutes at 20.9 min

Showing elution of daughters of 345.24 from parents of 792.55

Ion eluting at 21.3 min, 18:0p/22:5(O)-PE

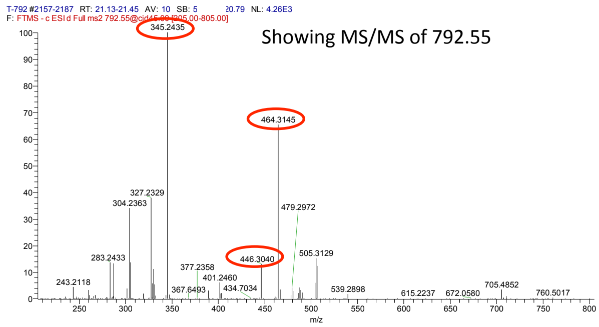

Showing MS/MS of 792.55

Ion eluting at 21.7 min, 18:0p/22:4(O)-PE

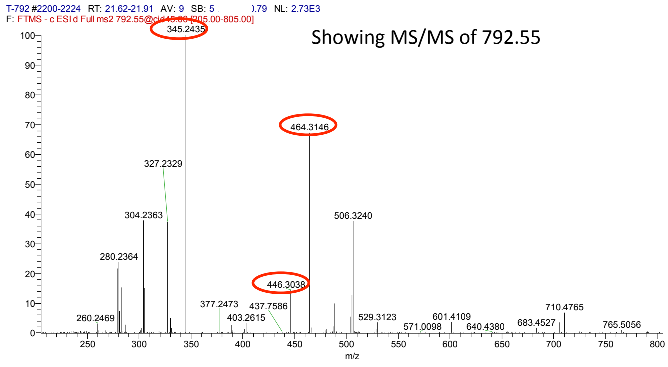

Showing MS/MS of 792.55

Ion eluting at 22.1 min, 18:0p/22:4(O)-PE

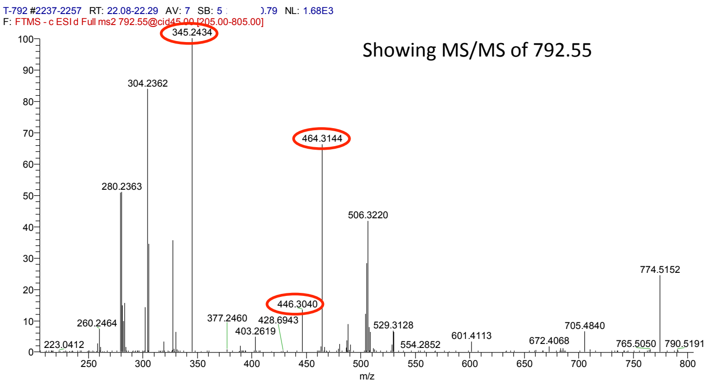

Showing MS/MS of 792.55

Structural identification of m/z 794.5701 as 18:0p/22:4(O)-PE

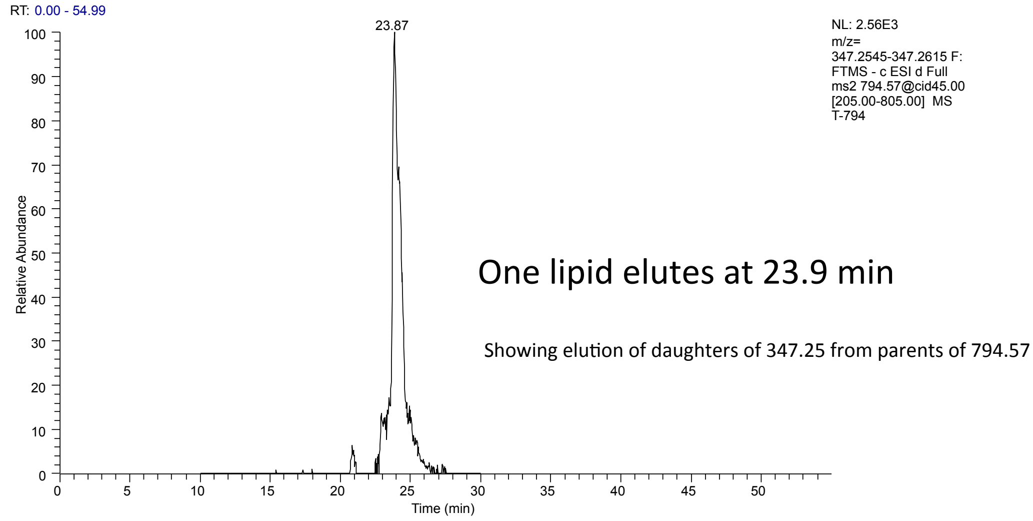

One lipid elutes at 23.9 min

Showing elution of daughters of 347.25 from parents of 794.57

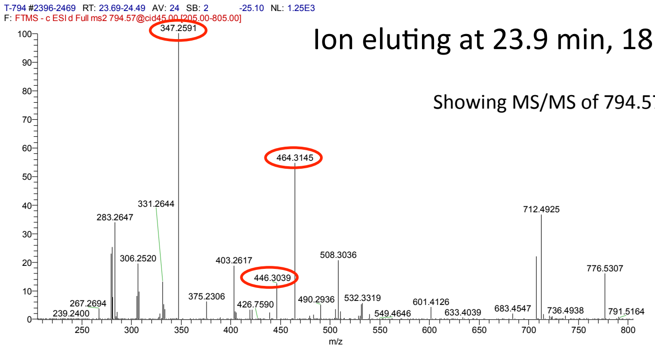

Ion eluting at 23.9 min, 18:0p/22:4(O)-PE

Showing MS/MS of 794.57

Structural identification of m/z 794.5701 as 20:0p/HETE-PE

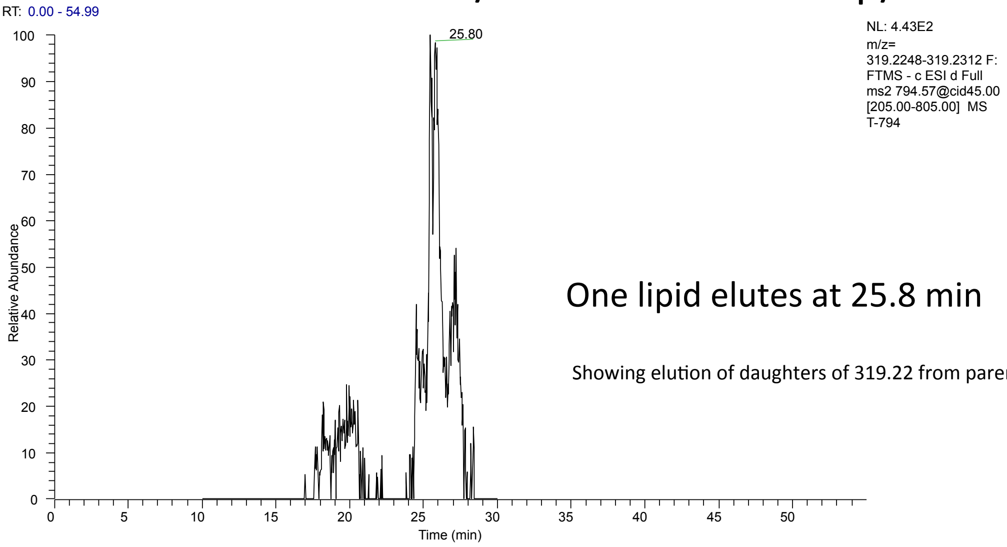

One lipid elutes at 25.8 min

Showing elution of daughters of 319.22 from parents of 794.57

Ion eluting at 25.8 min, 20:0p/HETE-PE

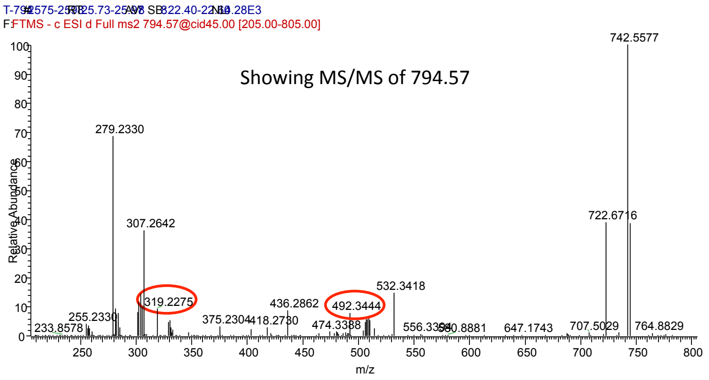

Showing MS/MS of 794.57

Structural identification of m/z 796.5134 as 18:1p/20:4(3O)-PE or 18:1p/DXA3-PE

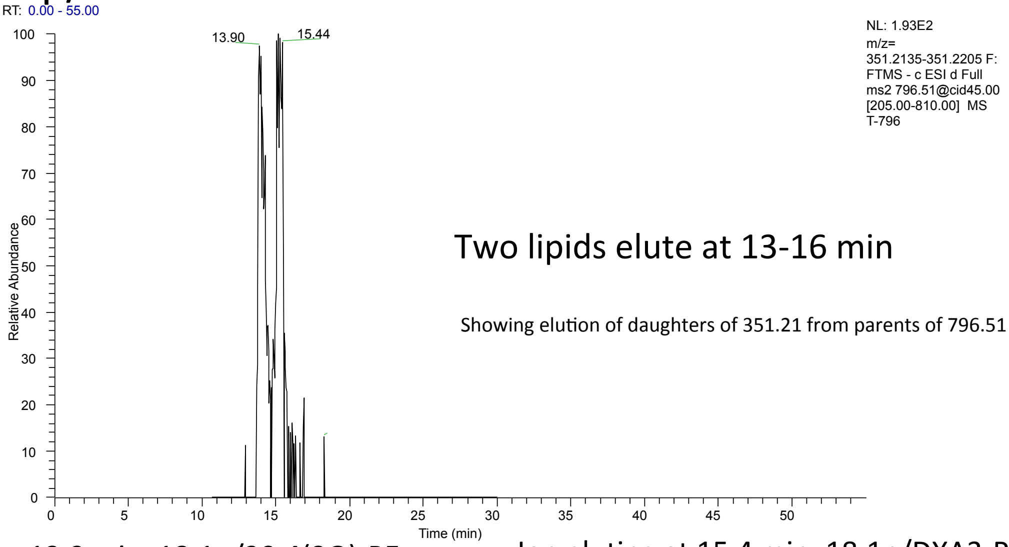

Ion eluting at 13.9 min, 18:1p/20:4(3O)-PE

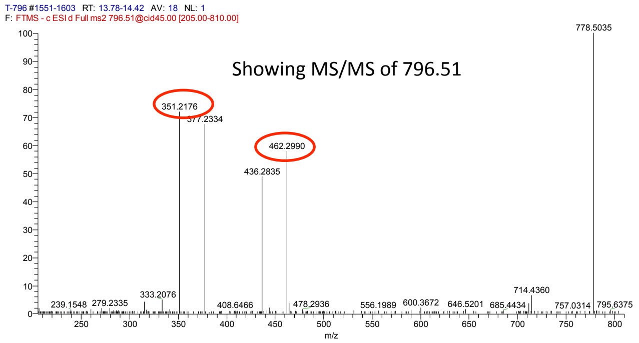

Ion eluting at 15.4 min, 18:1p/DXA3-PE

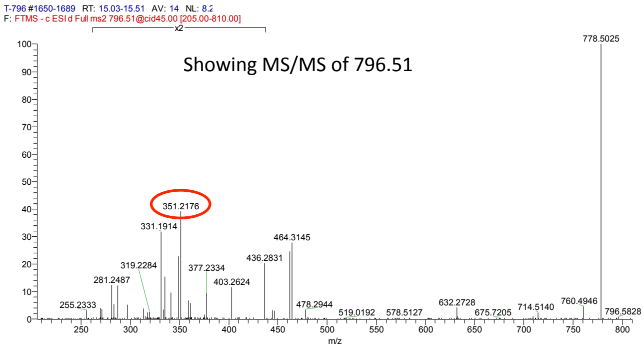

Ion eluting at 13.9 min, 18:1p/20:4(3O)-PE

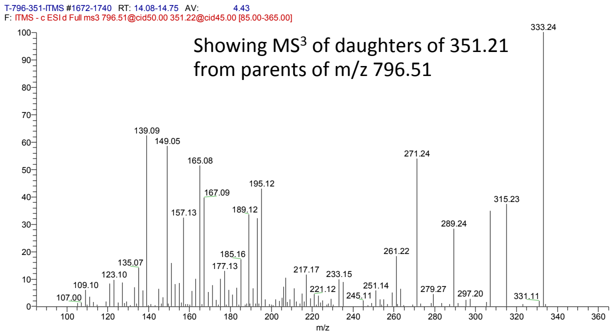

Ion eluting at 15.4 min, 18:1p/DXA3-PE

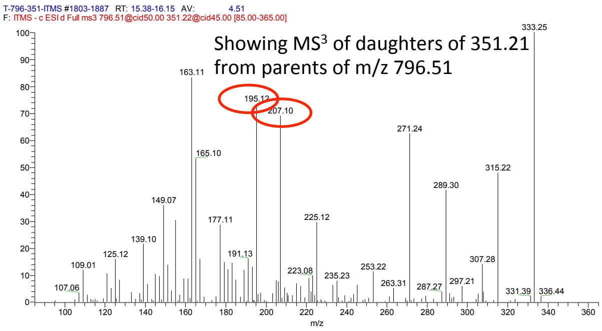

Structural identification of m/z 796.5134 as 16:0p/22:5(3O)-PE

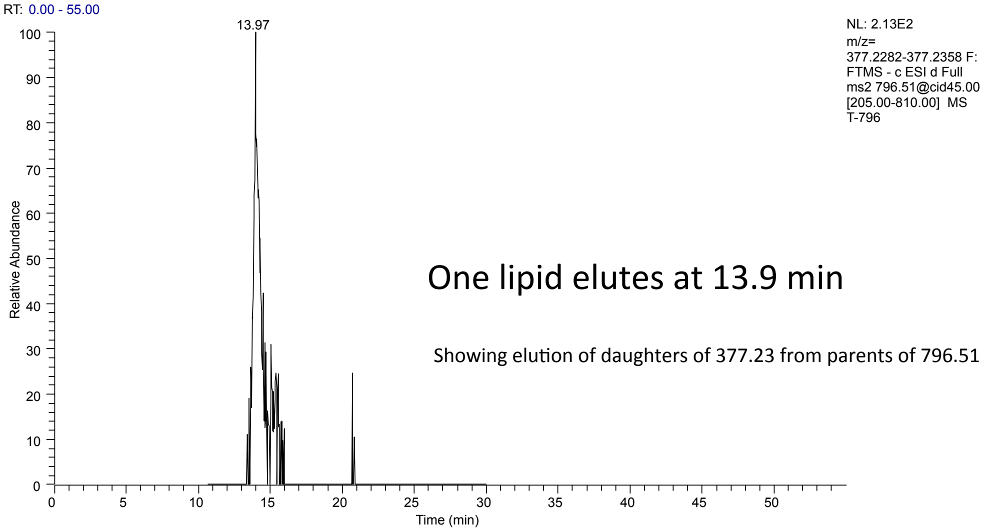

Ion eluting at 13.9 min, 16:0p/22:5(3O)-PE

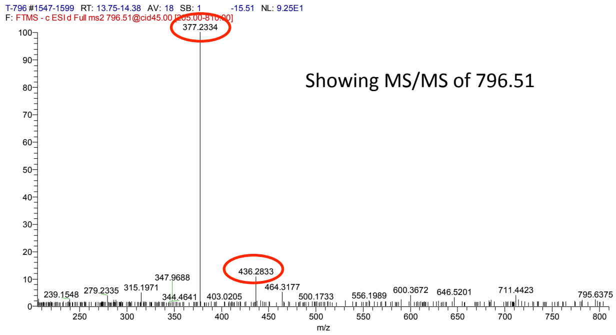

Structural identification of m/z 798.5291 as 18:0p/20:4(3O)-PE or 18:0p/DXA3-PE

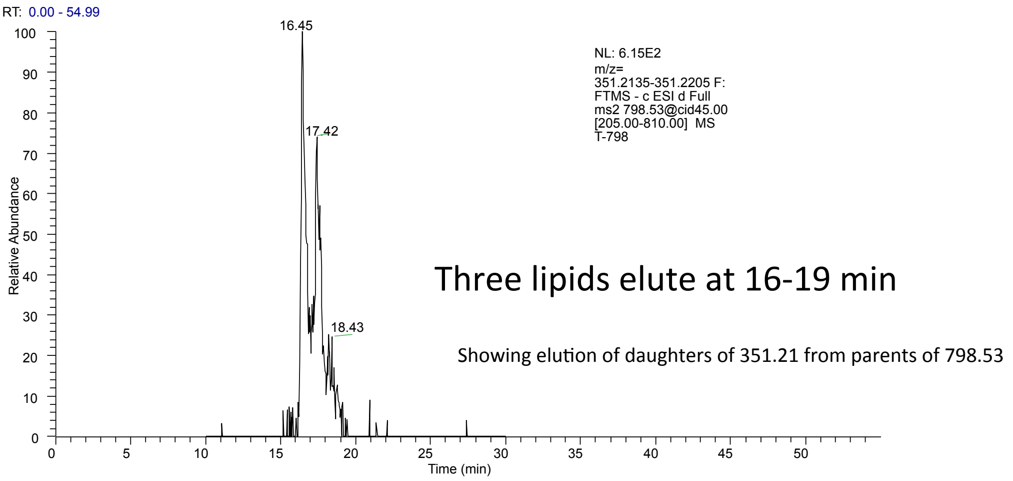

Ion eluting at 16.45 min, 18:0p/20:4(3O)-PE

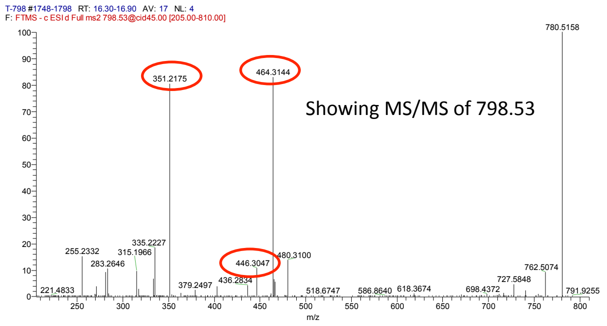

Ion eluting at 16.45 min, 18:0p/20:4(3O)-PE

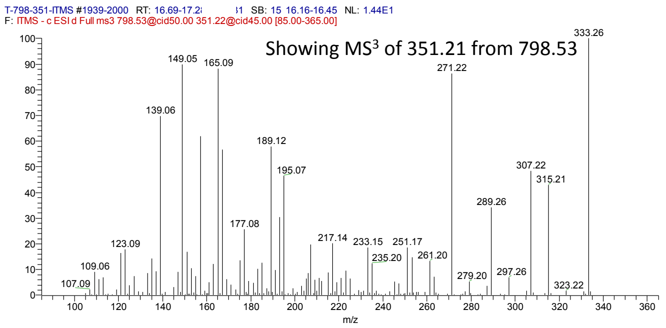

Ion eluting at 17.4 min, 18:0p/DXA3-PE

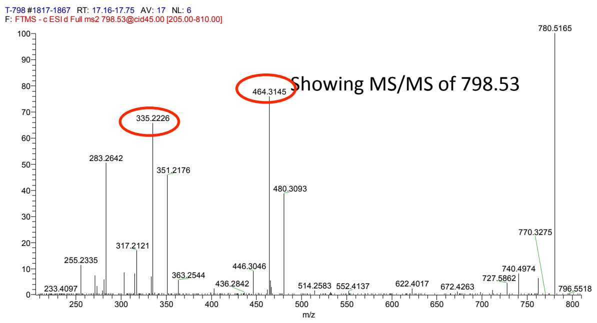

Ion eluting at 17.4 min, 18:0p/DXA3-PE

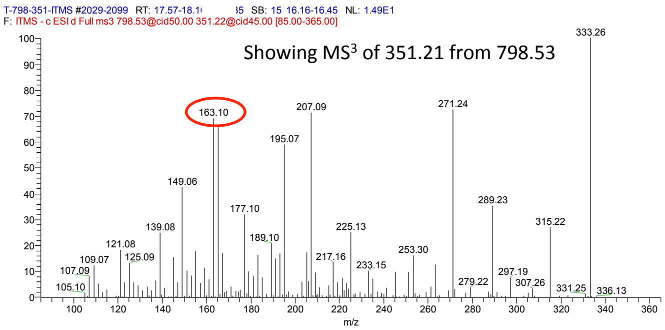

Ion eluting at 18.4 min, 18:0p/20:4(3O)-PE

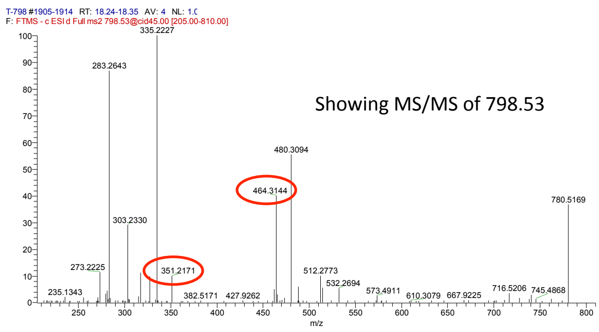

Ion eluting at 18.4 min, 18:0p/20:4(3O)-PE

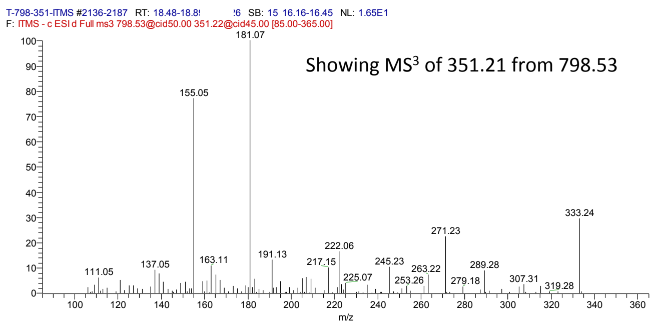

Structural identification of m/z 798.5291 as 18:0a/20:4(2O)-PE isomers

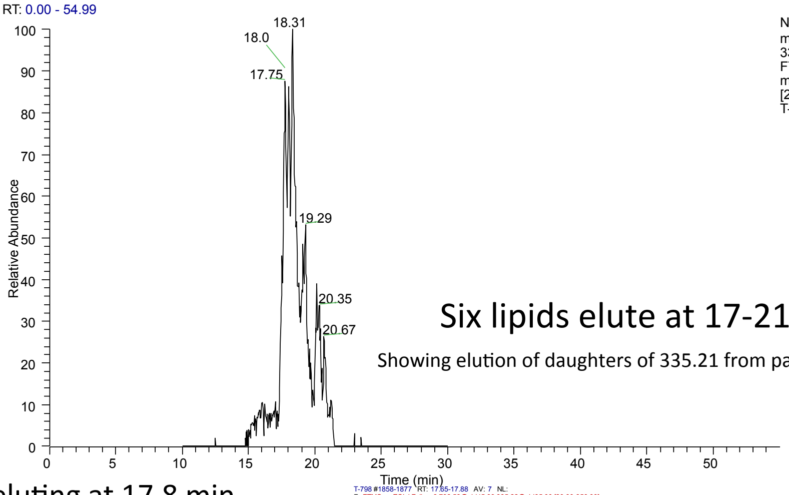

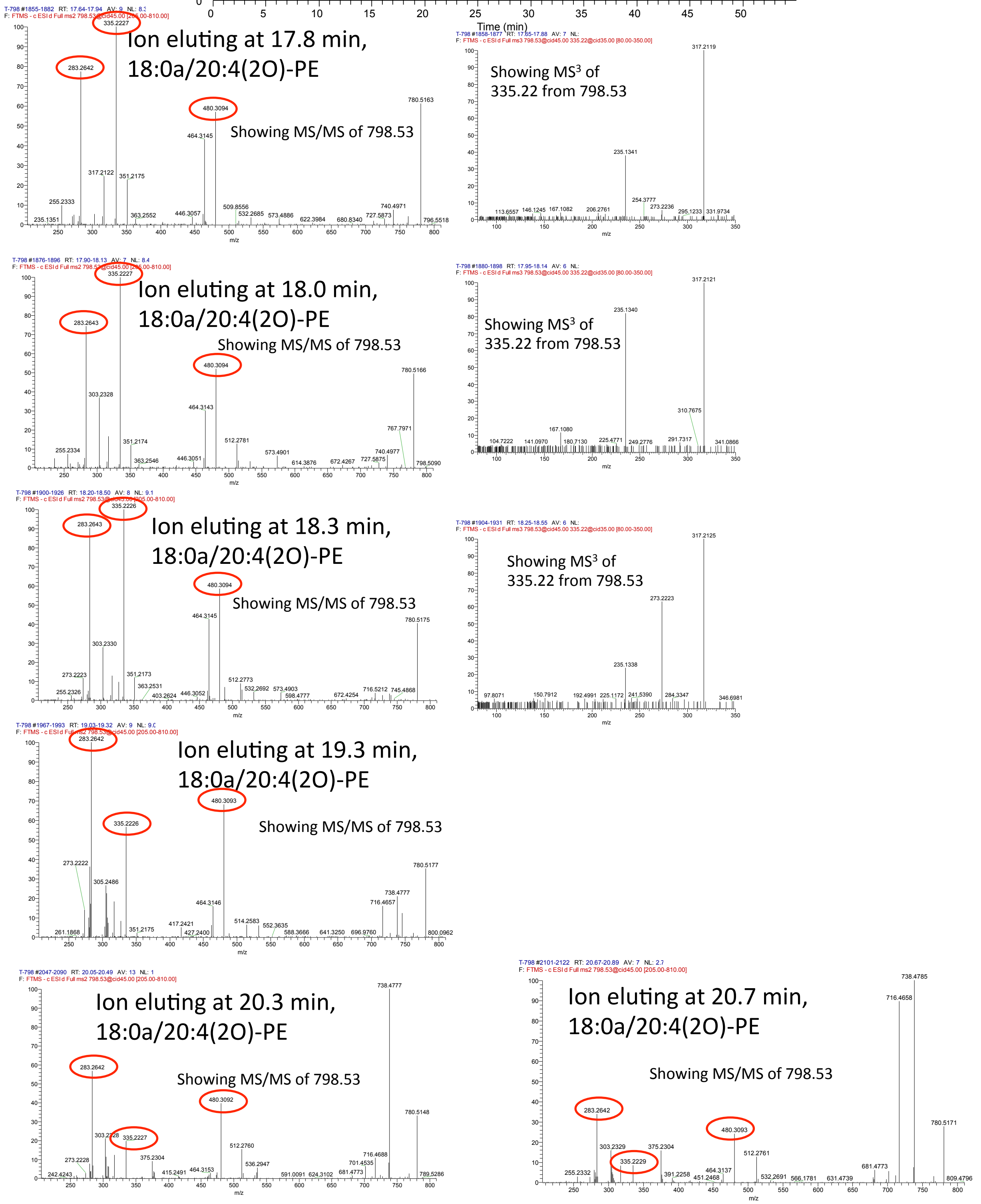

Structural identification of m/z 804.5187 as 18:1a/HDoHE-PE isomers

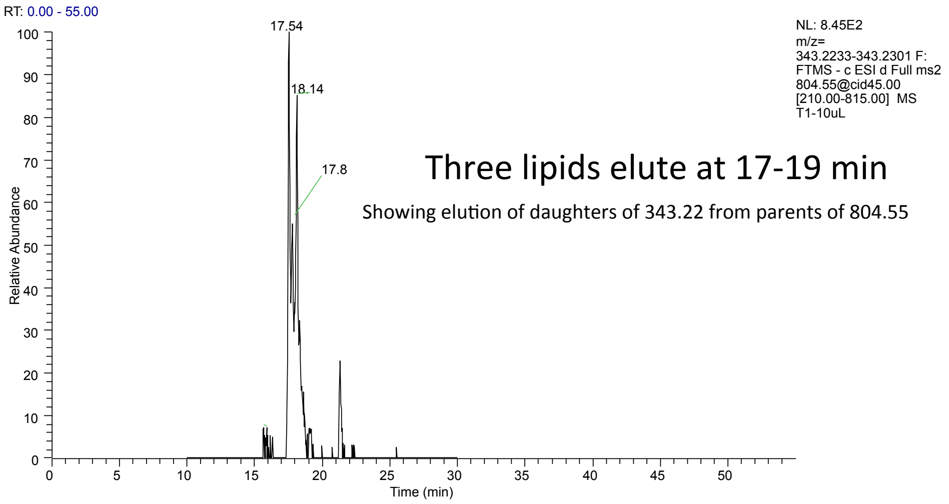

Three lipids elute at 17-19 min

Showing elution of daughters of 343.22 from parents of 804.55

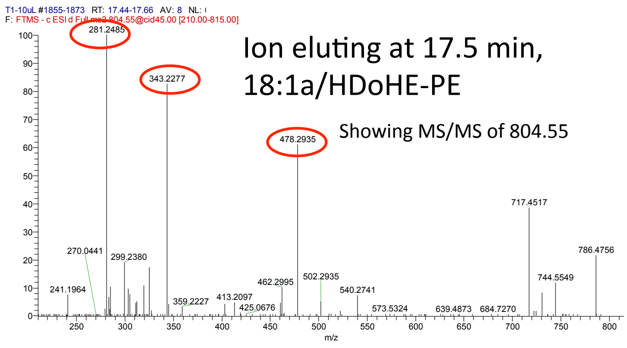

Ion eluting at 17.5 min, 18:1a/HDoHE-PE

Showing MS/MS of 804.55

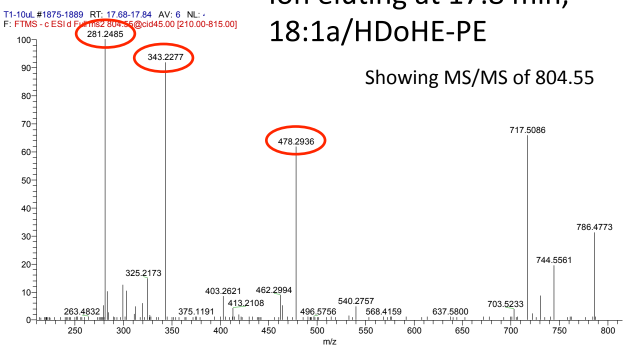

Ion eluting at 17.8 min, 18:1a/HDoHE-PE

Showing MS/MS of 804.55

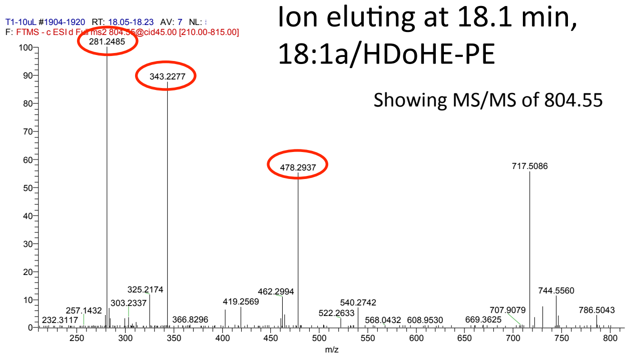

Ion eluting at 18.1 min, 18:1a/HDoHE-PE

Showing MS/MS of 804.55

Structural identification of co-eluting m/z 804.5187 at 16.79 min

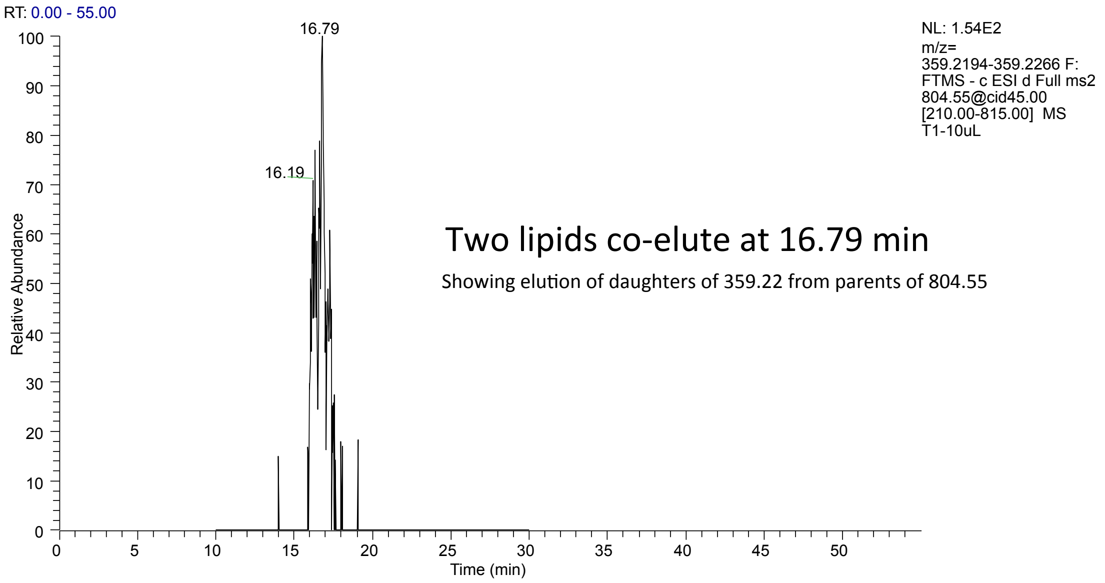

Structural identification of m/z 804.5187 as 18:2a/22:5(O)-PE

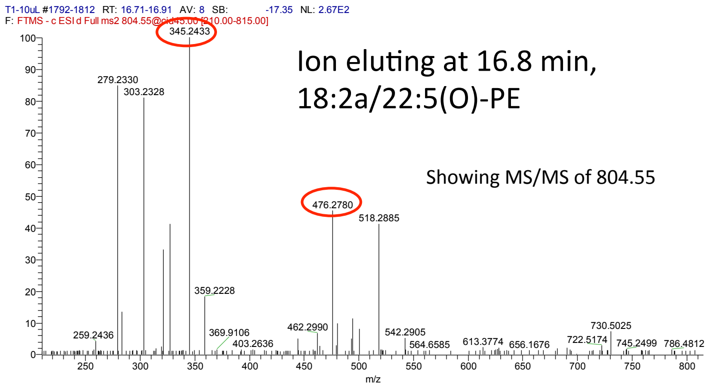

Structural identification of m/z 804.5187 as 18:1p/22:6(2O)-PE

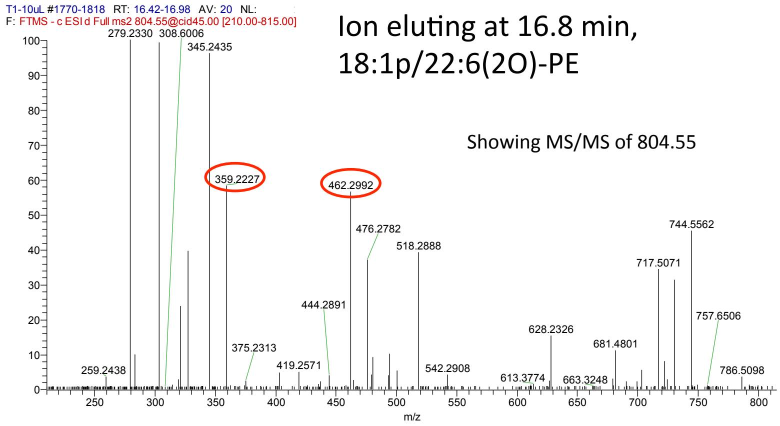

Structural identification of co-eluting m/z 804.5548 at 16.32 min

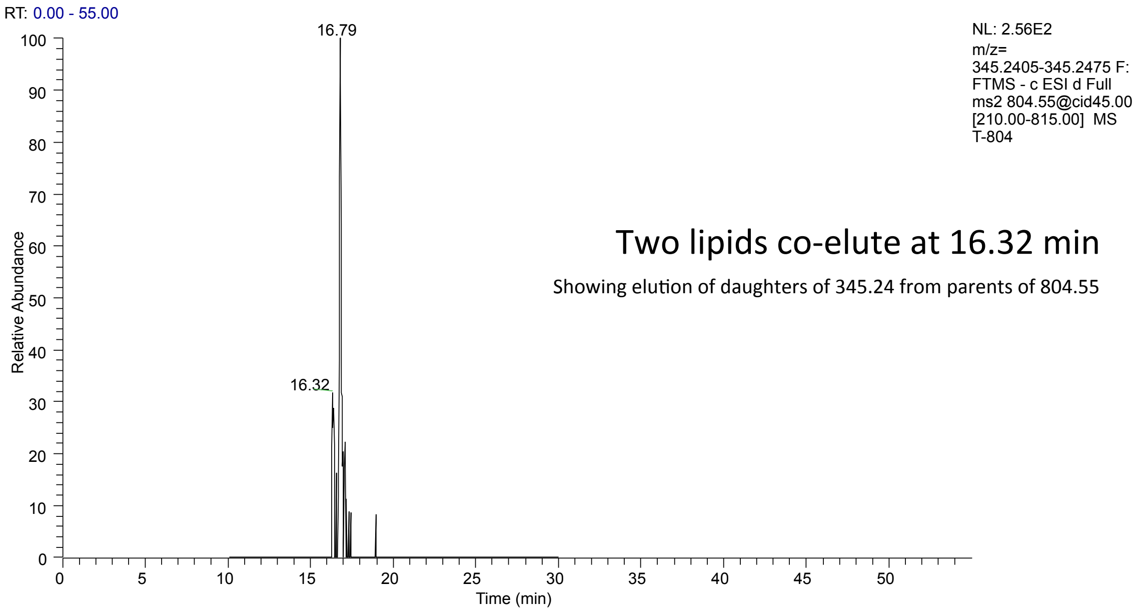

Structural identification of m/z 804.5548 as 16:1p/22:5(O)-PC

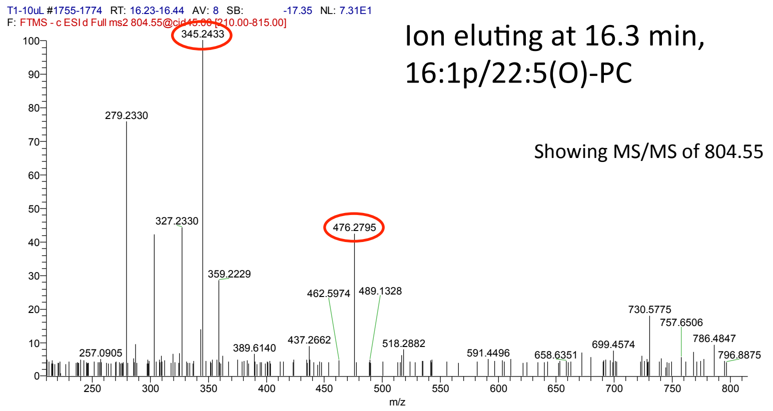

Structural identification of m/z 804.5548 as 16:0p/22:6(2O)-PC

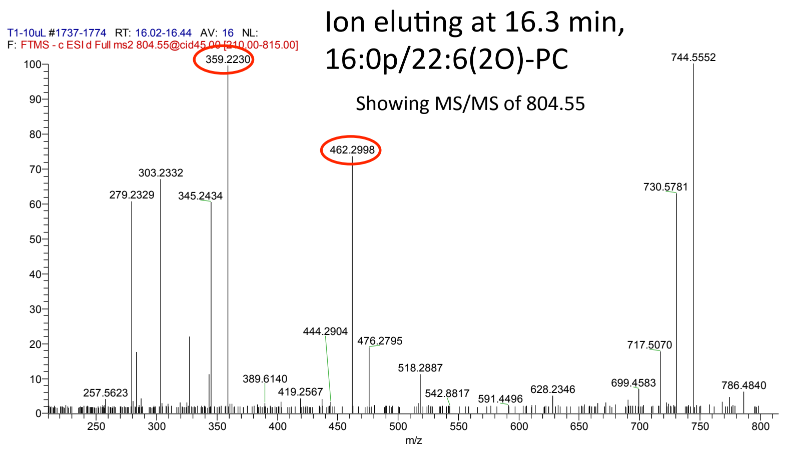

Structural identification of m/z 806.5342 as 18:2a/HETE-PC

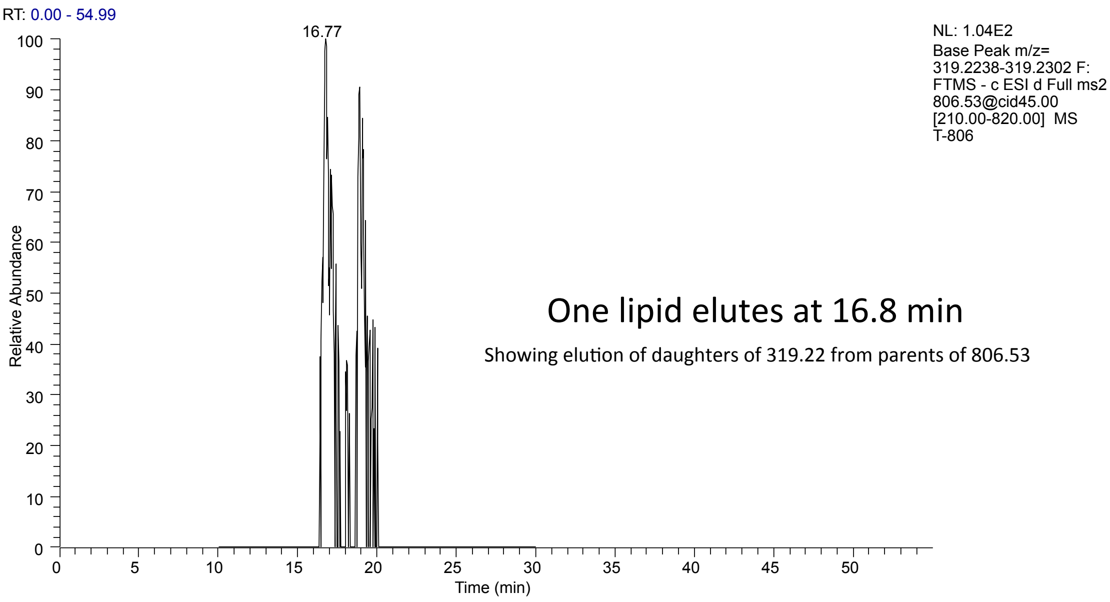

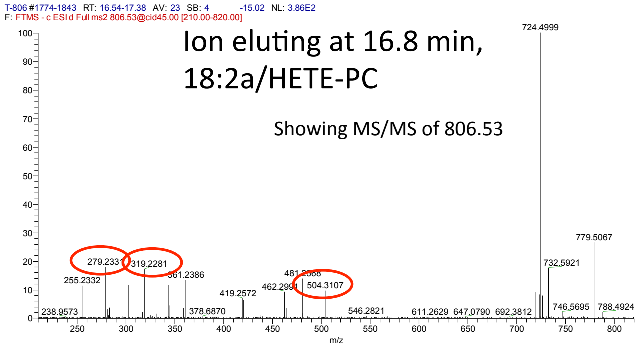

Structural identification of m/z 806.5342 as 18:1p/22:5(2O)-PE

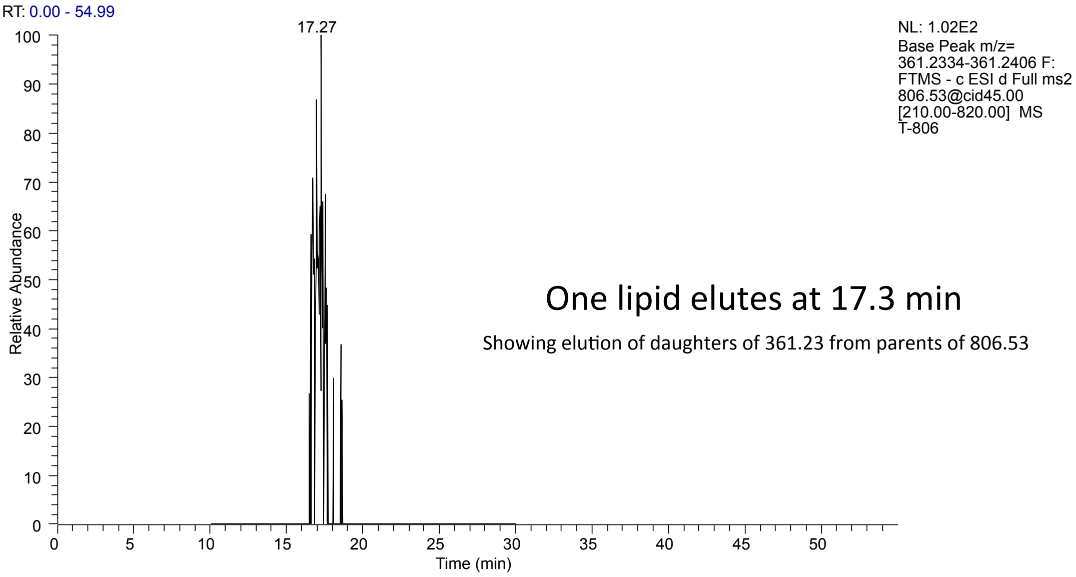

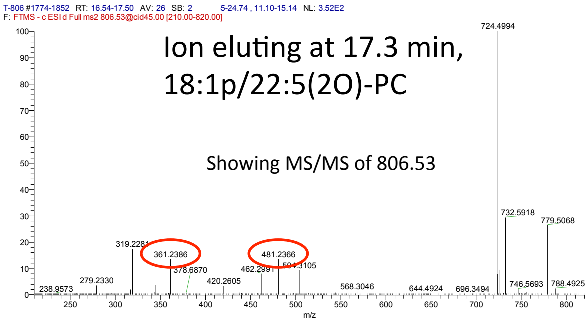

Structural identification of m/z 806.5342 as HDoHE-PE/PC isomers

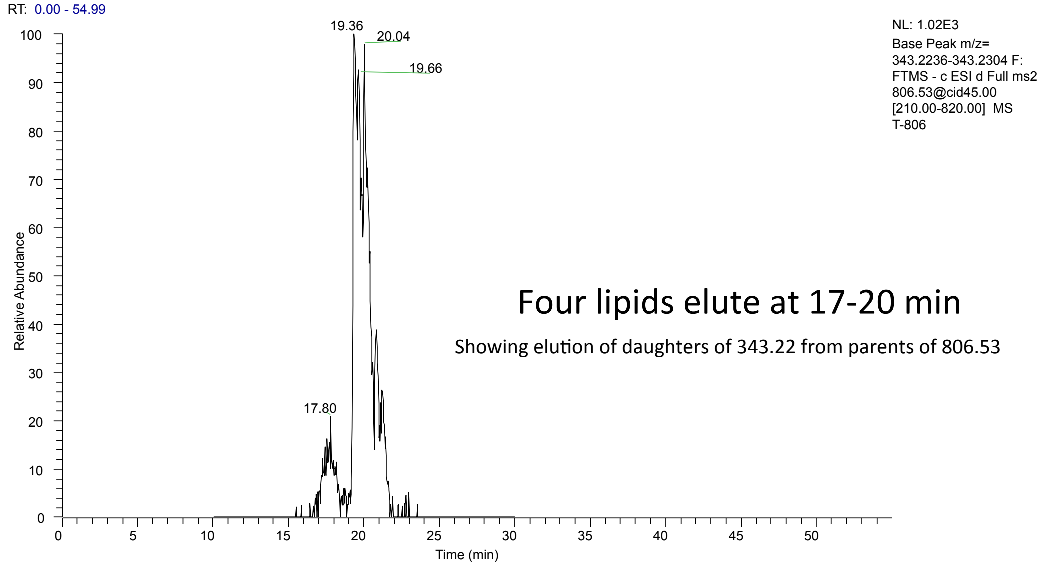

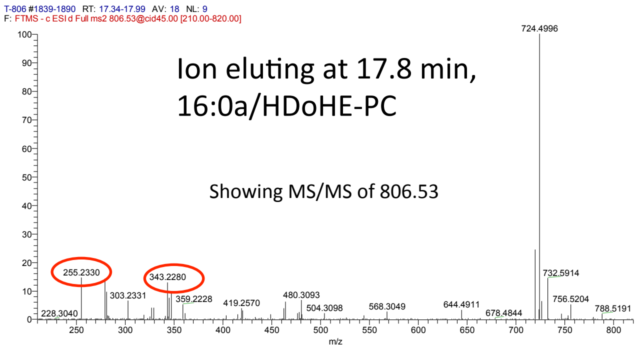

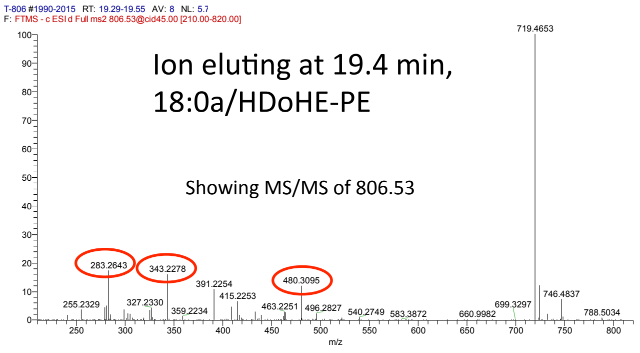

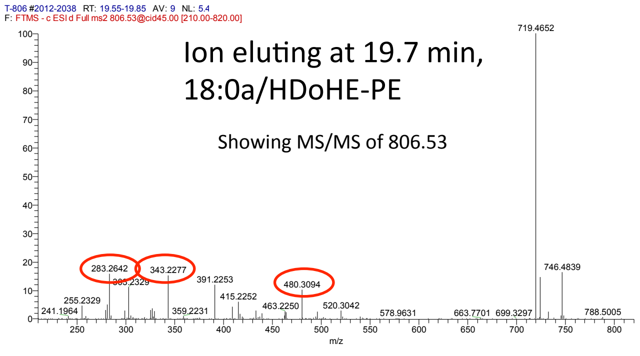

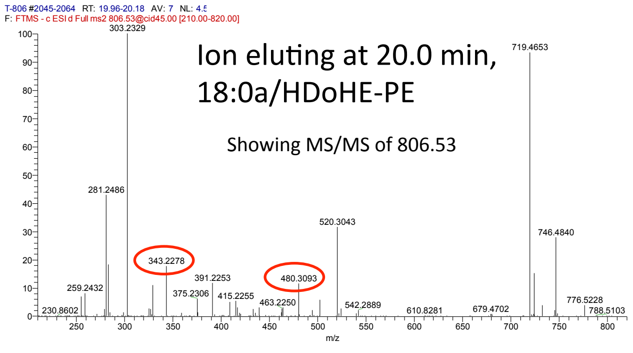

Structural identification of m/z 806.5342 as 18:2a/22:4(O)-PE

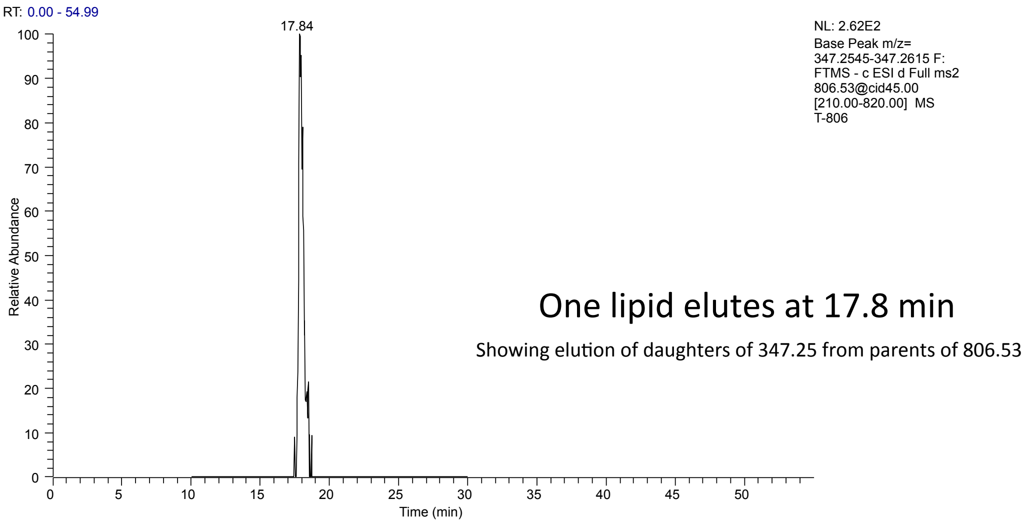

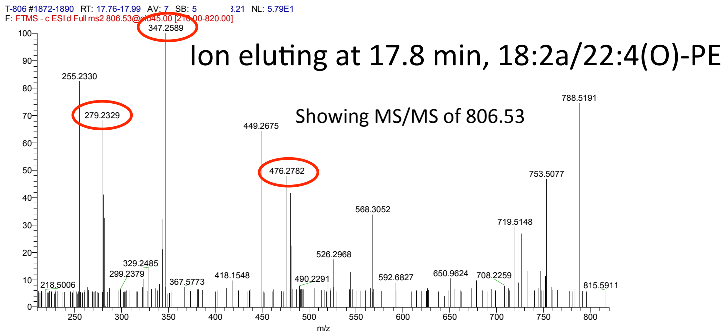

Structural identification of m/z 806.5342 as 18:1a/22:5(O)-PE

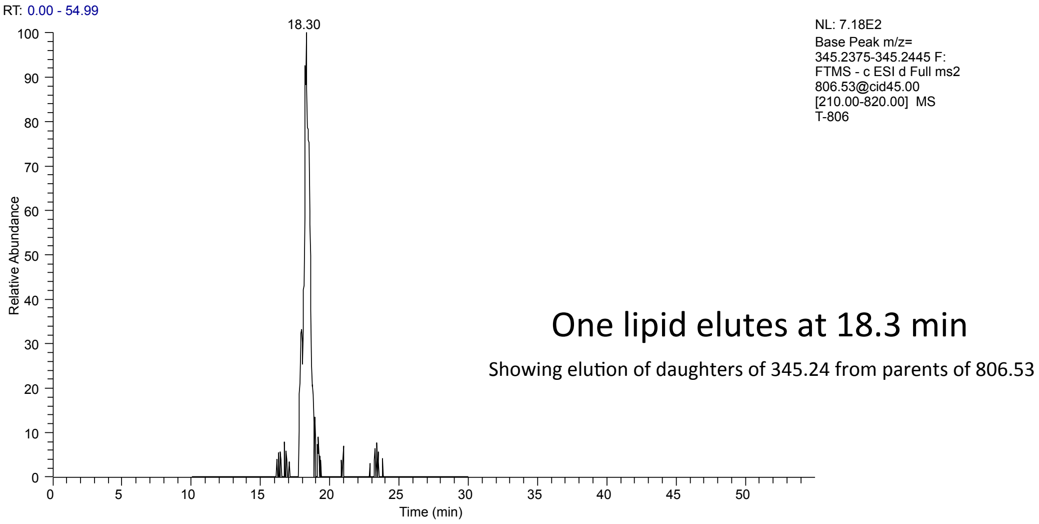

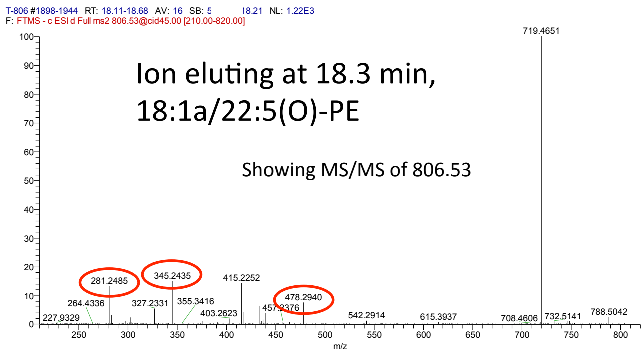

Structural identification of m/z 806.5342 as 18:0p/22:6(2O)-PE

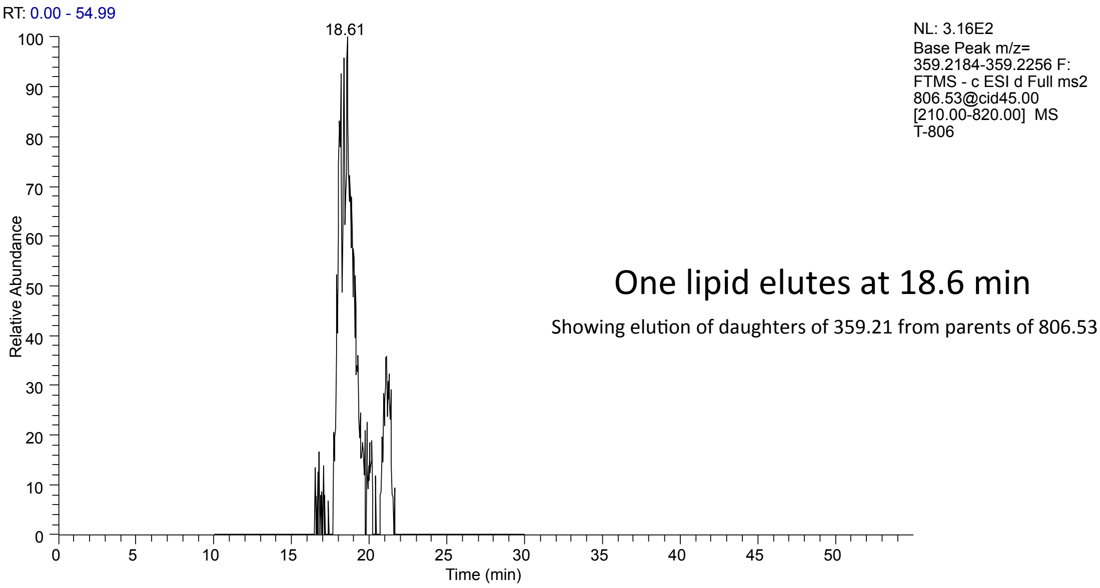

One lipid elutes at 18.6 min

Showing elution of daughters of 359.21 from parents of 806.53

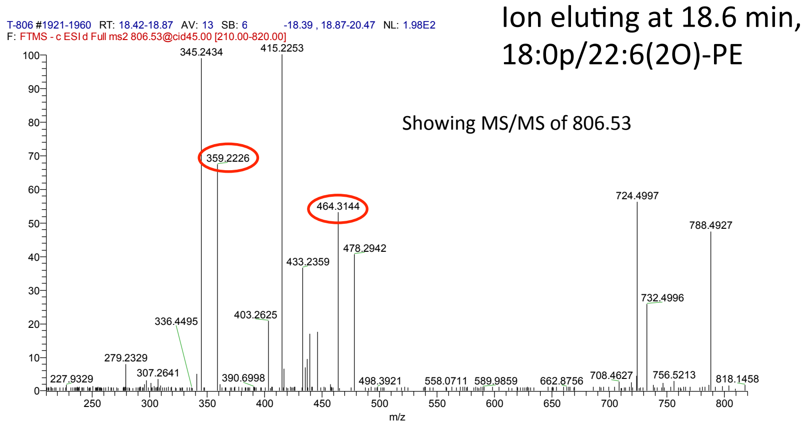

Ion eluting at 18.6 min, 18:0p/22:6(2O)-PE

Showing MS/MS of 806.53

Structural identification of m/z 808.5501 as 22:5(O)-PE/PC isomers

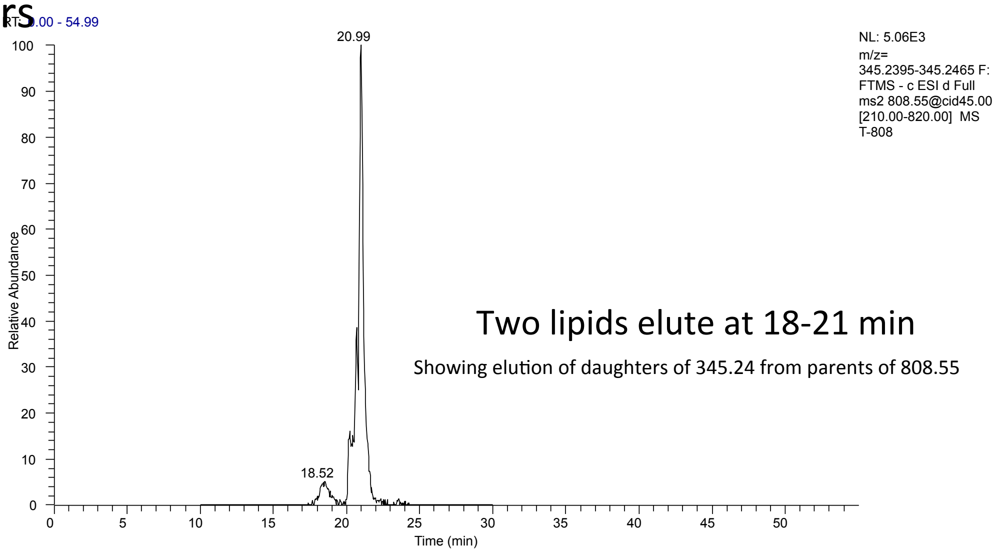

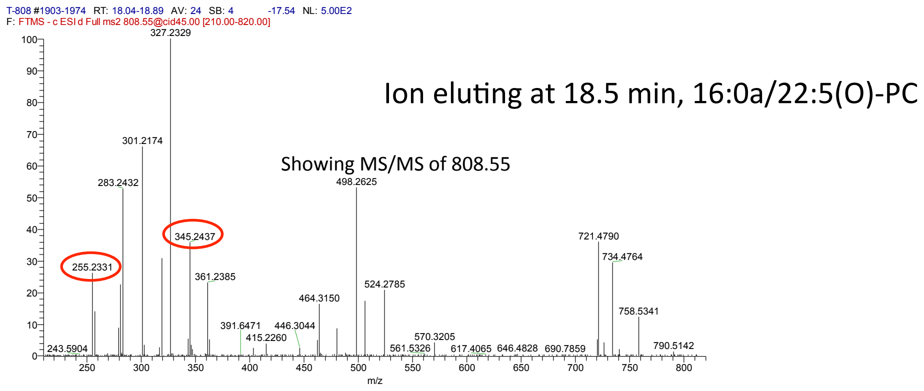

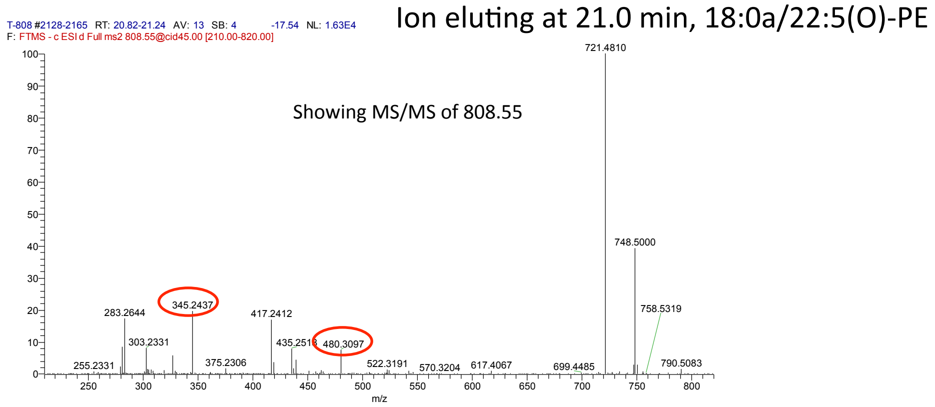

Structural identification of m/z 808.5501 as 18:0p/22:5(2O)-PE isomers

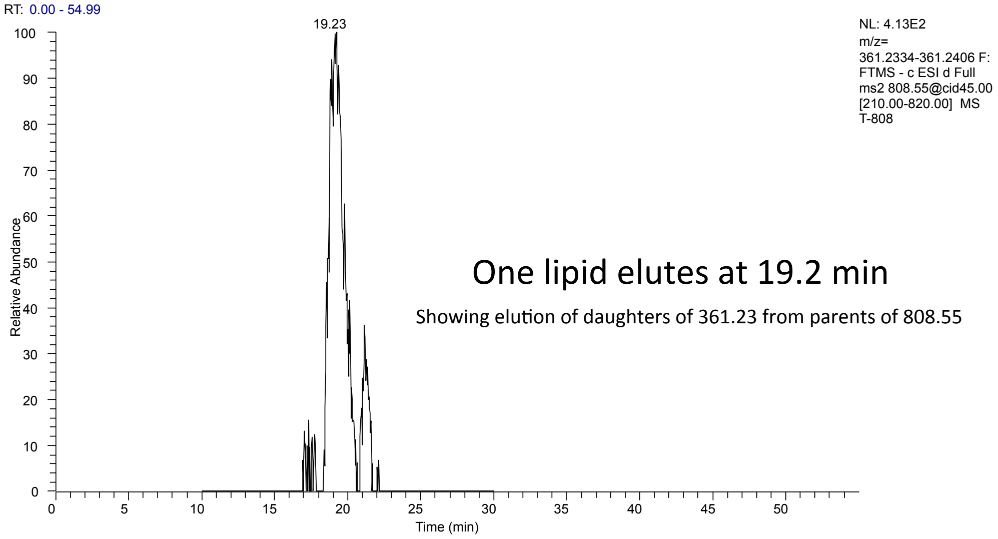

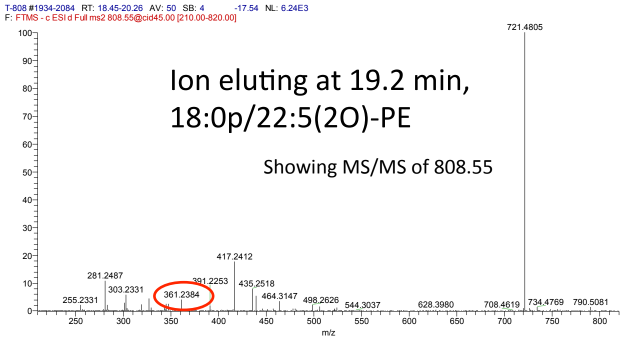

Structural identification of m/z 808.5501 as HETE-PE/PC isomers

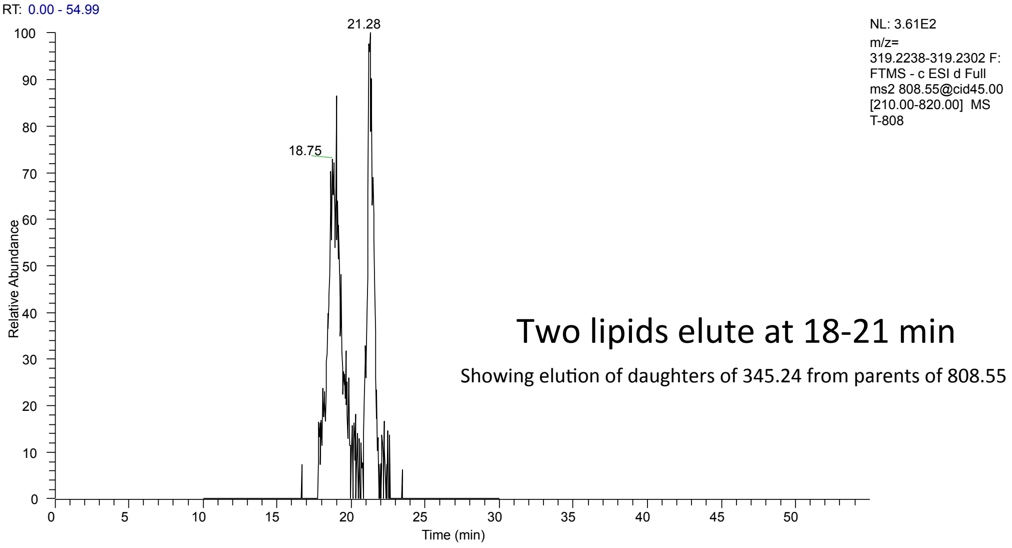

Two lipids elute at 18-21 min

Showing elution of daughters of 345.24 from parents of 808.55

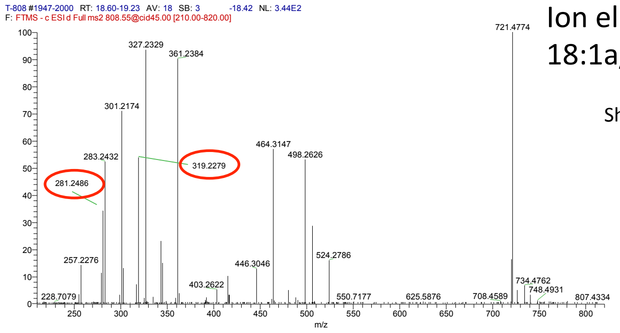

Ion eluting at 18.8 min, 18:1a/12-HETE-PC

Showing MS/MS of 808.55

Ion eluting at 21.3 min, 20:1a/12-HETE-PE

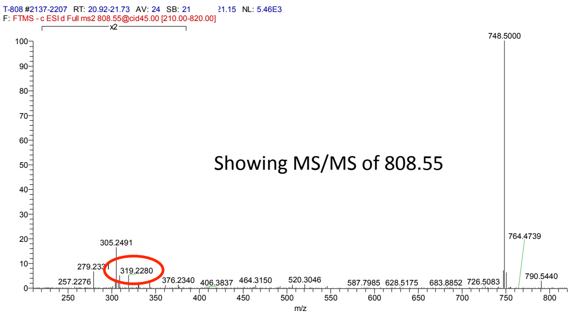

Showing MS/MS of 808.55

Ion eluting at 21.3 min, 20:1a/12-HETE-PE

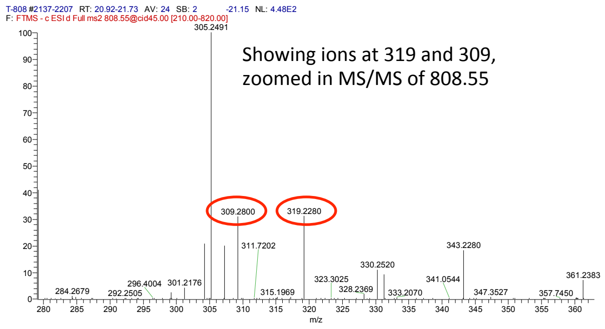

Showing ions at 319 and 309, zoomed in MS/MS of 808.55

Structural identification of m/z 808.5501 as 18:1a/22:4(O)-PE

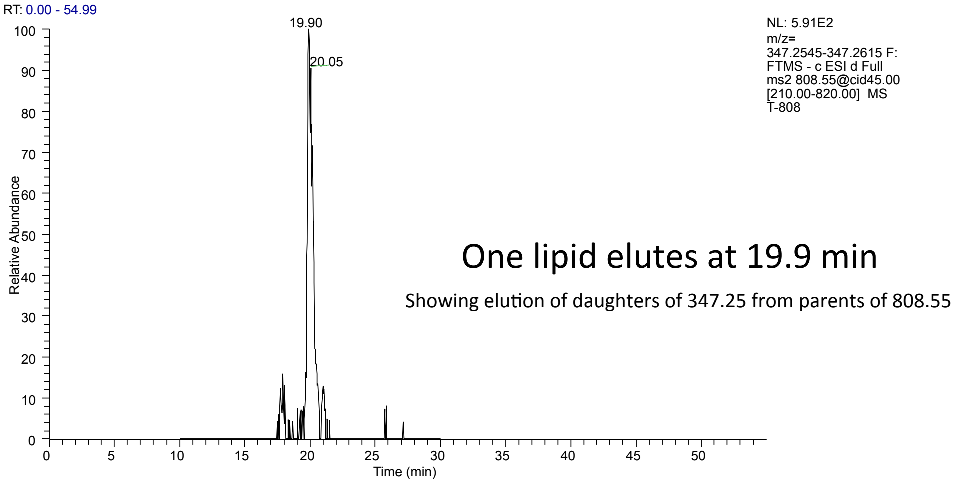

One lipid elutes at 19.9 min

Showing elution of daughters of 347.25 from parents of 808.55

Ion eluting at 19.9 min, 18:1a/22:4(O)-PE

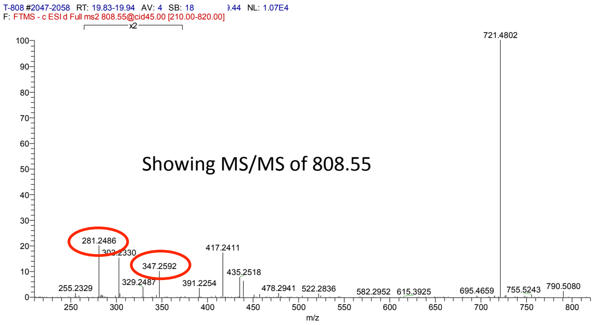

Showing MS/MS of 808.55

Structural identification of m/z 810.5657 as 18:0p/22:4(2O)-PE

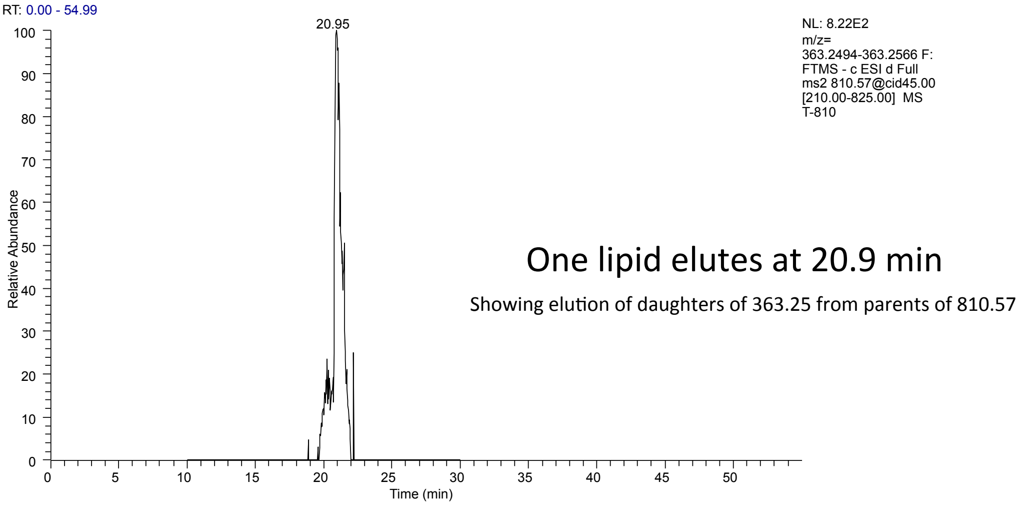

Ion eluting at 20.9 min, 18:0p/22:4(2O)-PE

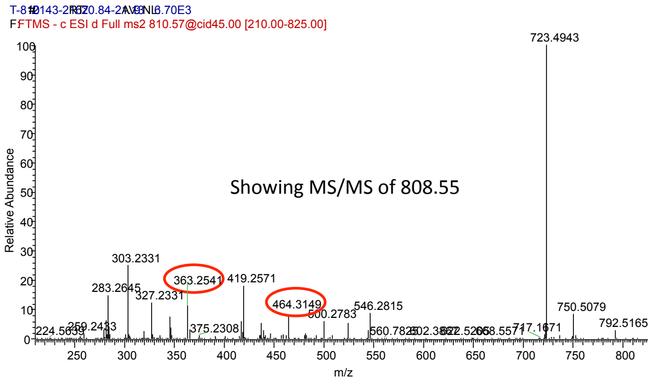

Structural identification of m/z 814.5242 as 18:0p/20:4(4O)-PE

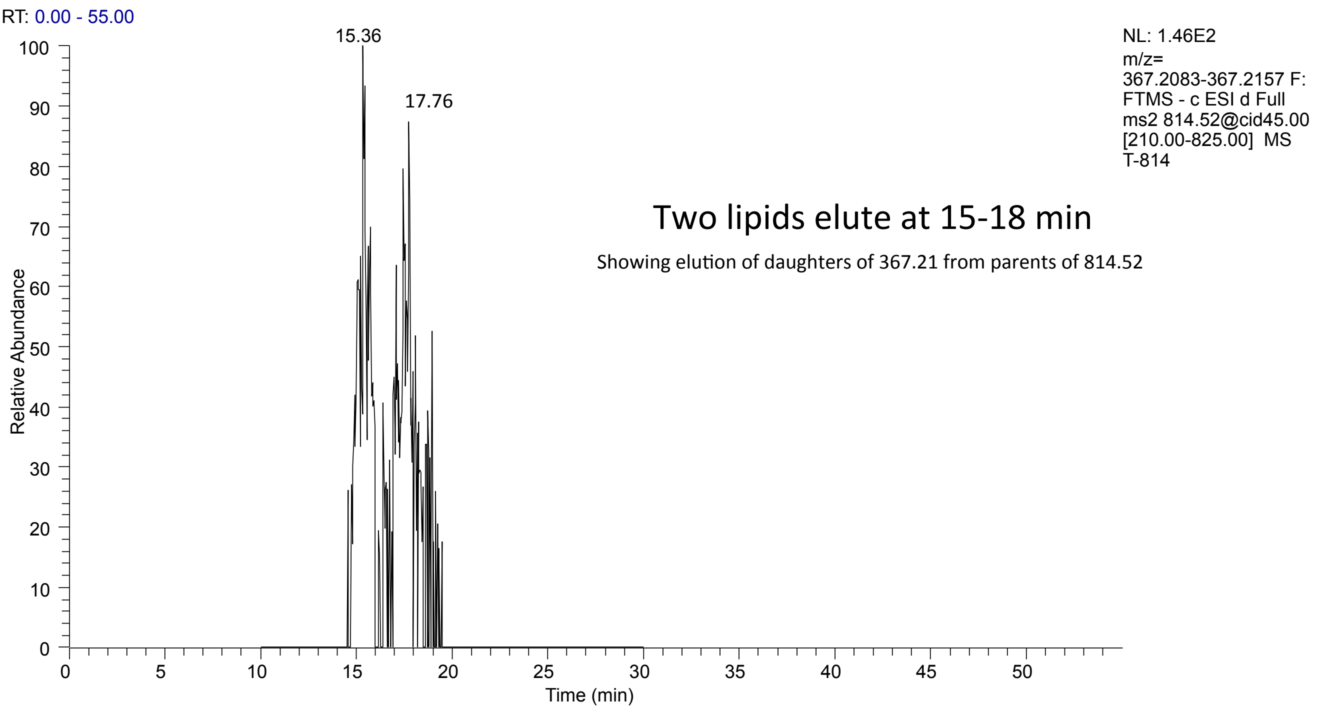

Ion eluting at 15.4 min, 18:0p/20:4(4O)-PE

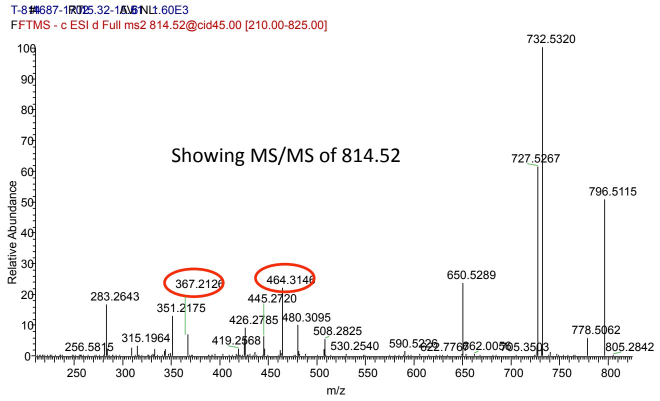

Ion eluting at 17.7 min, 18:0p/20:4(4O)-PE

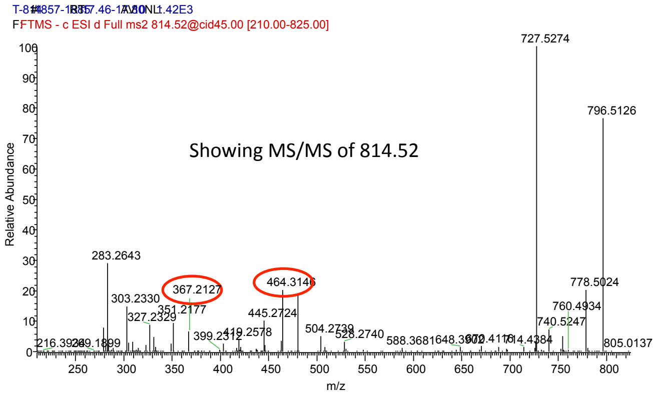

Structural identification of m/z 814.5242 as 18:0a/20:4(3O)-PE isomers (as PGE2/D2 and/or DXA3-PEs)

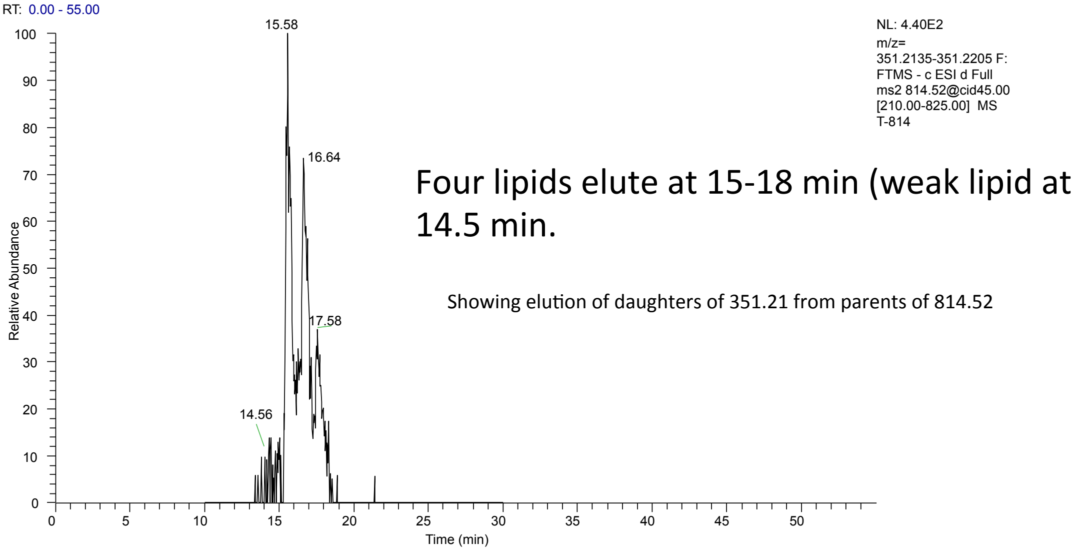

Four lipids elute at 15-18 min (weak lipid at 14.5 min.

Showing elution of daughters of 351.21 from parents of 814.52

Ion eluting at 14.5 min, 18:0a/20:4(3O)-PE

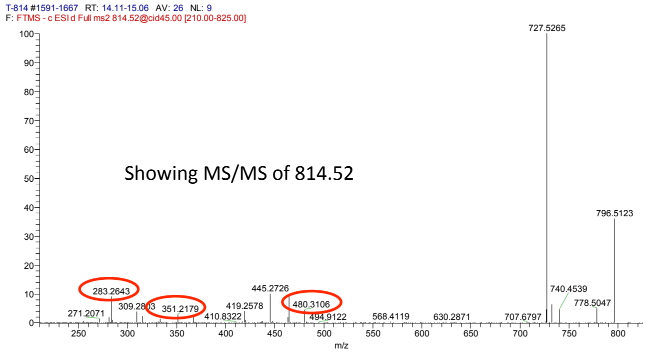

Showing MS/MS of 814.52

Ion eluting at 15.6 min, 18:0a/20:4(3O)-PE

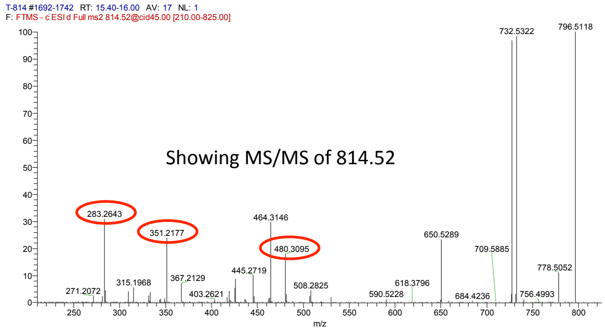

Showing MS/MS of 814.52

Ion eluting at 16.6 min, 18:0a/20:4(3O)-PE

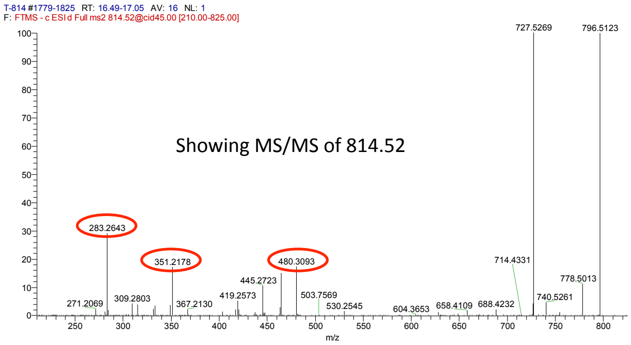

Showing MS/MS of 814.52

Ion eluting at 17.6 min, 18:0a/20:4(3O)-PE

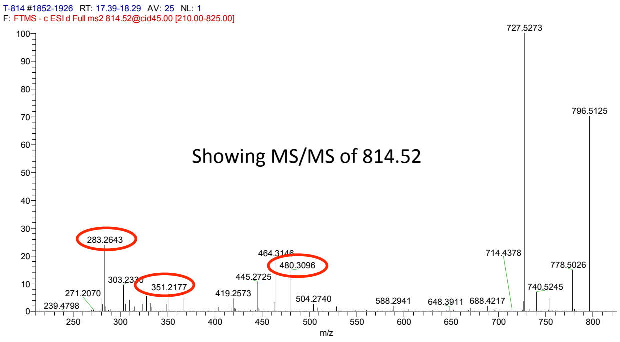

Showing MS/MS of 814.52

Structural identification of m/z 814.5242 as 18:0a/20:4(3O)-PE isomers (including DXA3 and/or PGE2/D2-PEs)

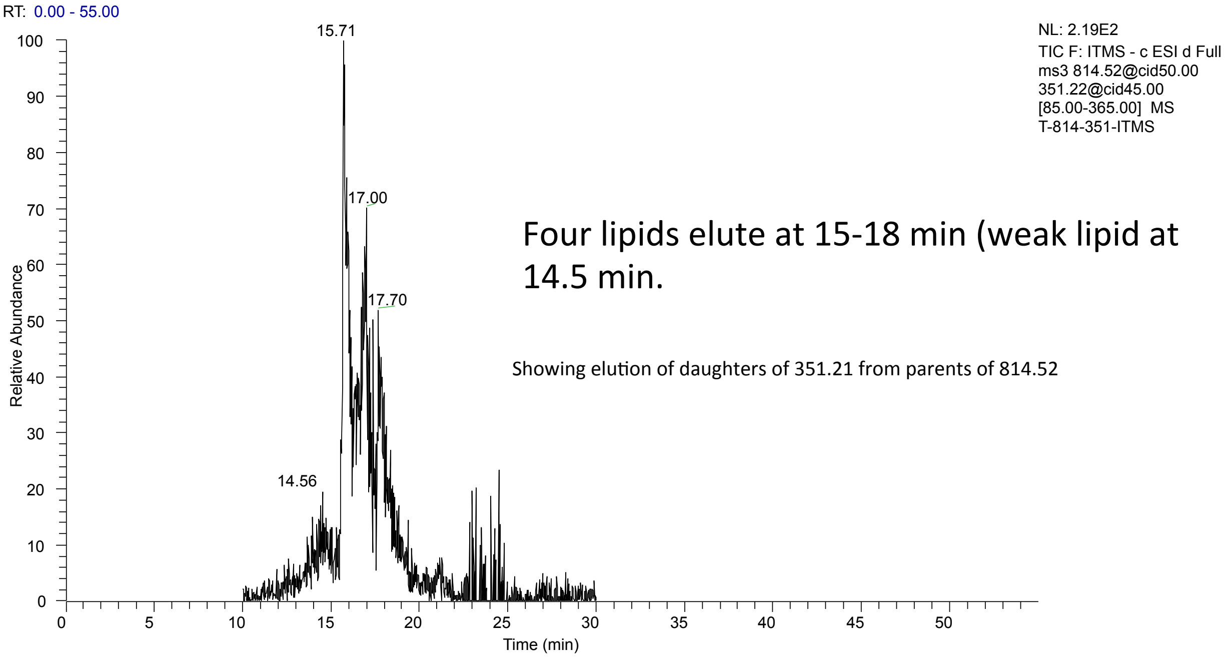

Ion eluting at 14.5 min, 18:0a/PGE2/D2-PE

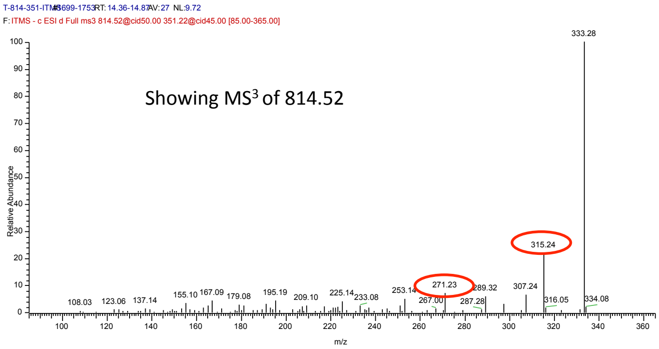

Ion eluting at 15.7 min, 18:0a/20:4(3O)-PE

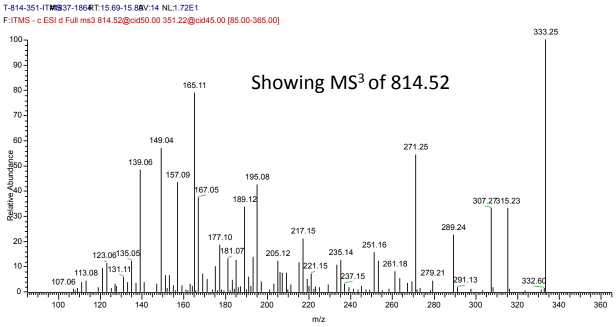

Ion eluting at 17 min, 18:0a/DXA3-PE

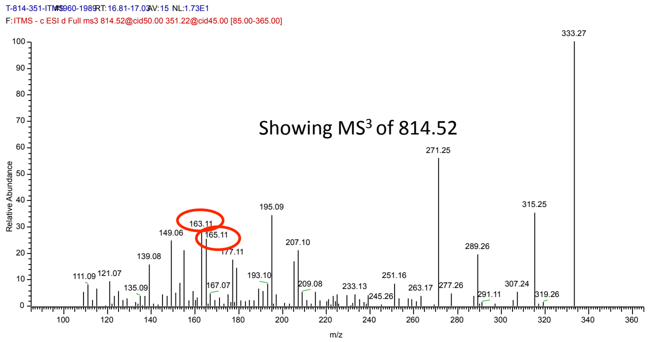

Ion eluting at 17.7 min, 18:0a/20:4(3O)-PE

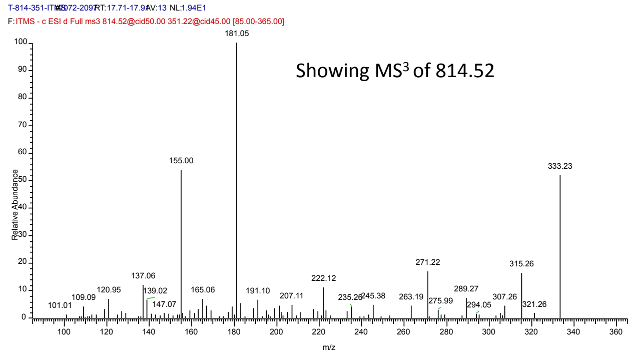

Supplement: Data S4. Full MS/MS Information on oxPL, Related to Table S2 [file mmc5.pdf]
