# Supplementary material for: Mapping the Human Platelet Lipidome Reveals Cytosolic Phospholipase A2 as a Regulator of Mitochondrial Bioenergetics during Activation
Source: Cell Metab. 2016 May 10;23(5):930–44. doi: 10.1016/j.cmet.2016.04.001 (PMC4873619; doi:10.1016/j.cmet.2016.04.001)
Supplement: Data S5. Full MS/MS Information on All FA, Related to Table S4 [file mmc6.pdf]

## HTDE mixture  m/z 239.1656  $C_{14}H_{23}O_3$

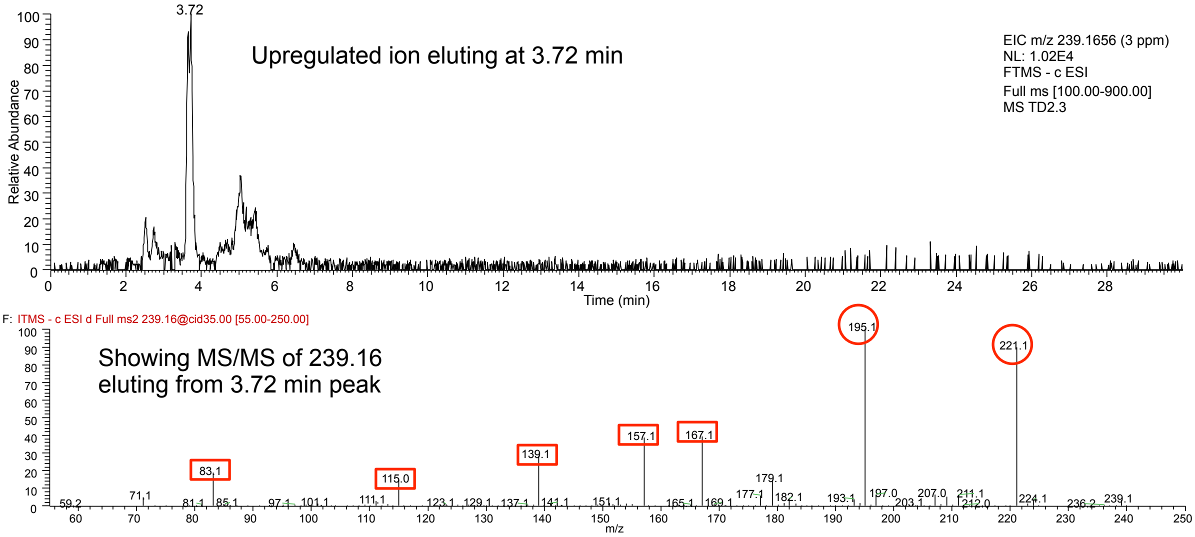


## Palmitolinoleic Acid  m/z 251.2021  $C_{16}H_{27}O_2$

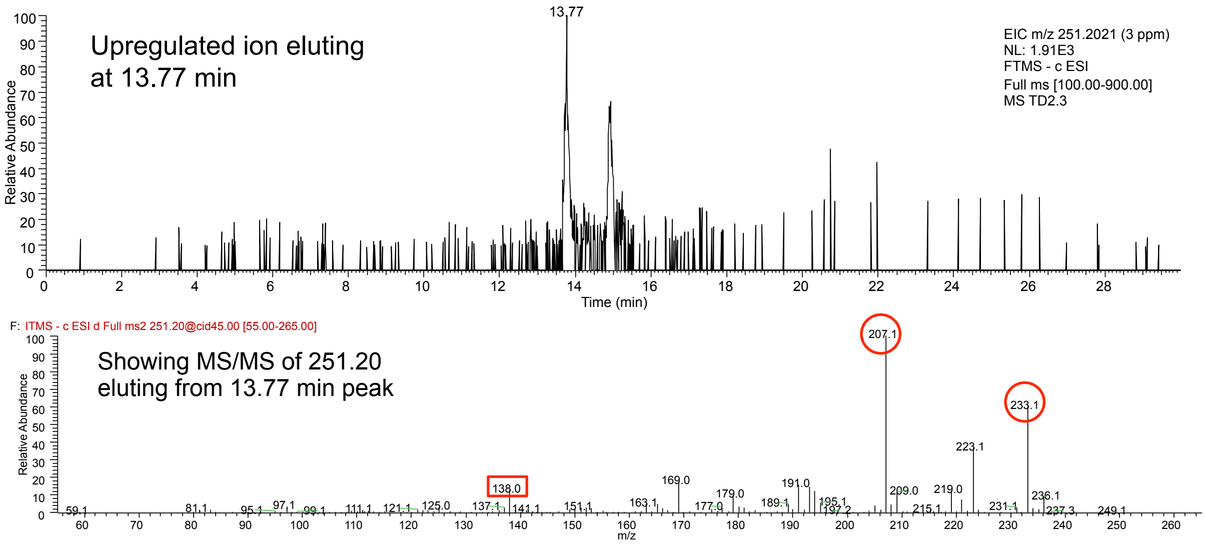


## Palmitic Acid  m/z 255.2332  $C_{16}H_{31}O_2$

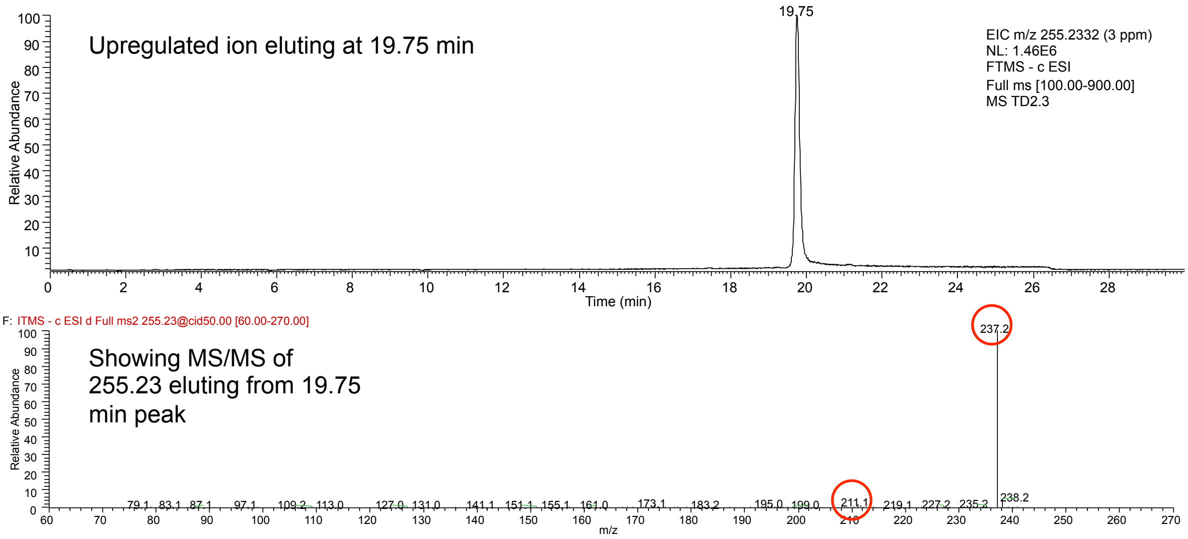

## Palmitoleic acid isomers — m/z 253.2177 — $C_{16}H_{29}O_2$

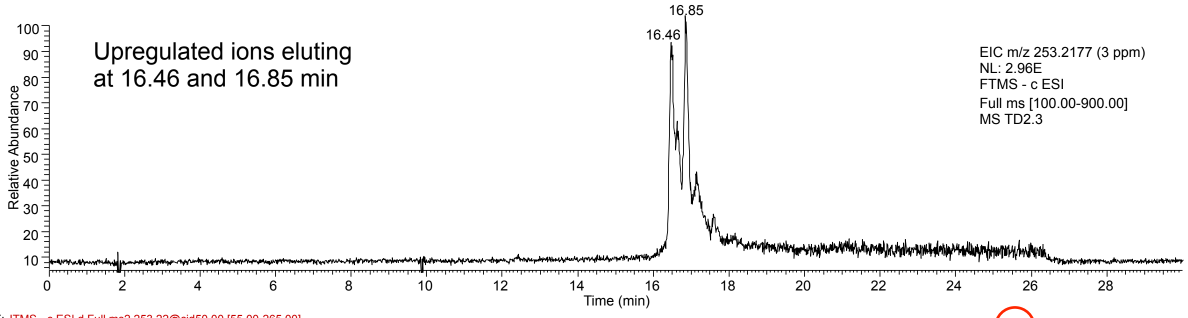


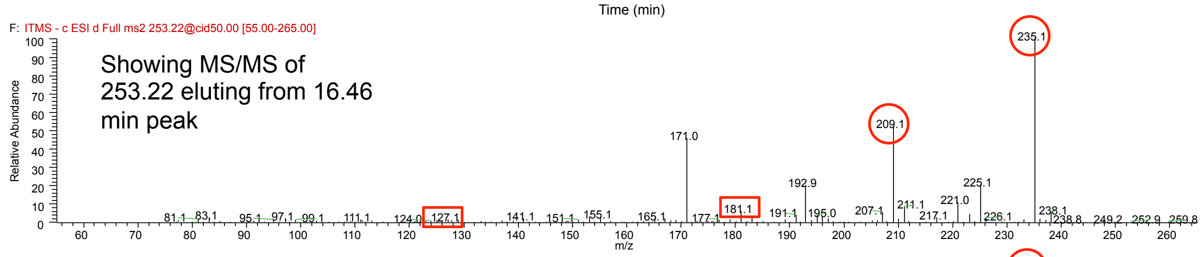


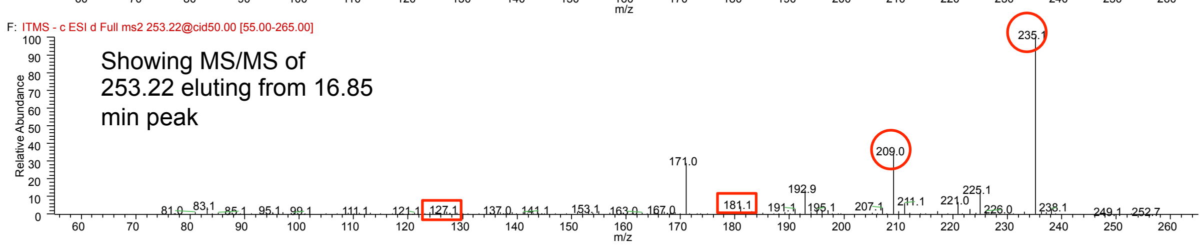


## Stearidonic Acid — m/z 275.2019 — $C_{18}H_{27}O_2$

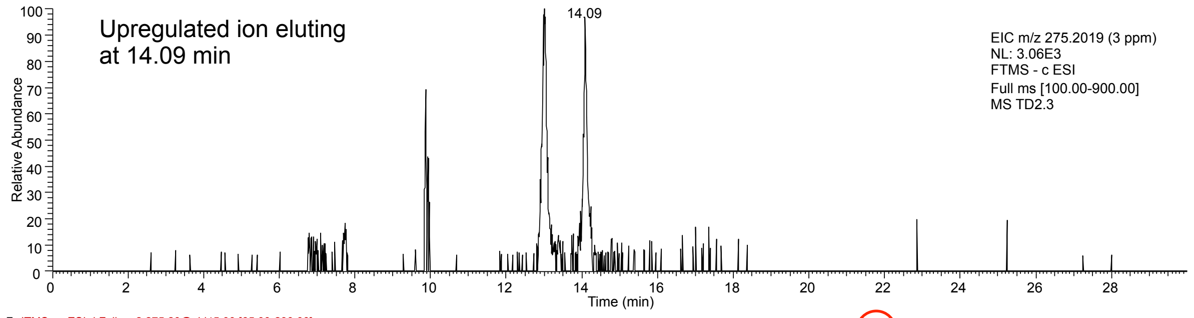


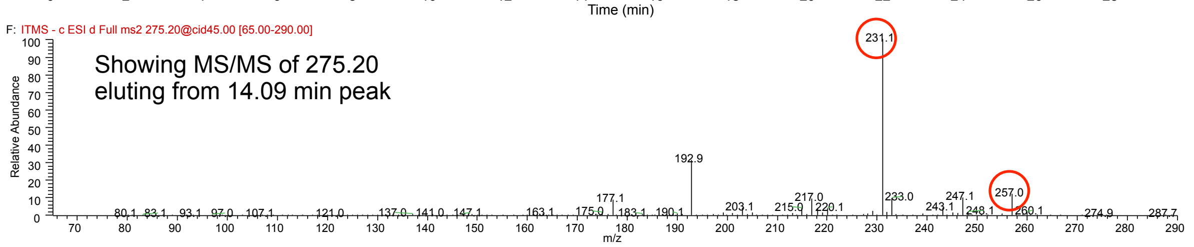

## α-Linolenic Acid m/z 277.2175 $C_{18}H_{29}O_2$

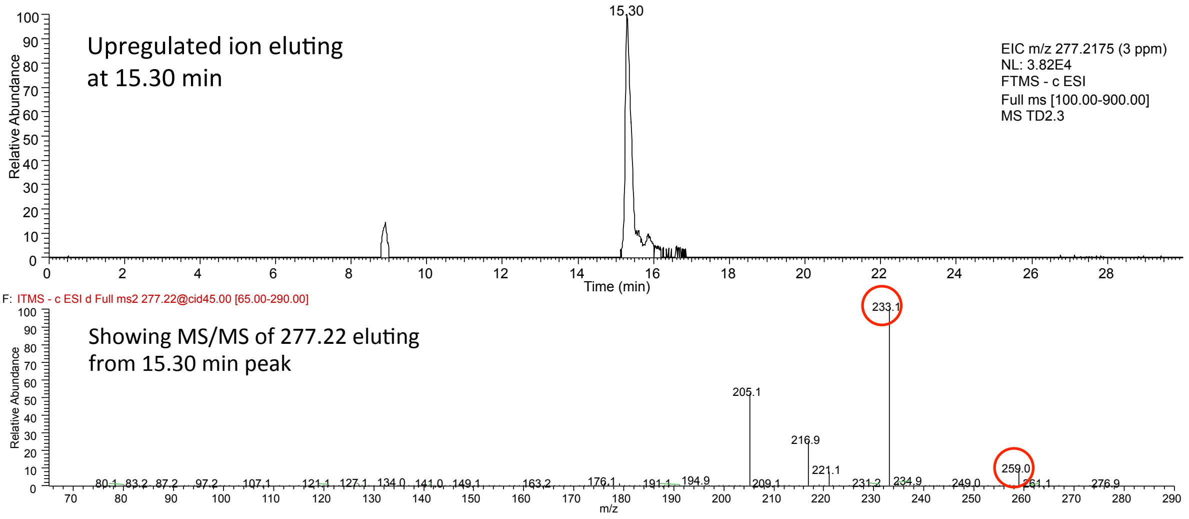


## 12-HHTrE m/z 279.1964 $C_{17}H_{27}O_3$

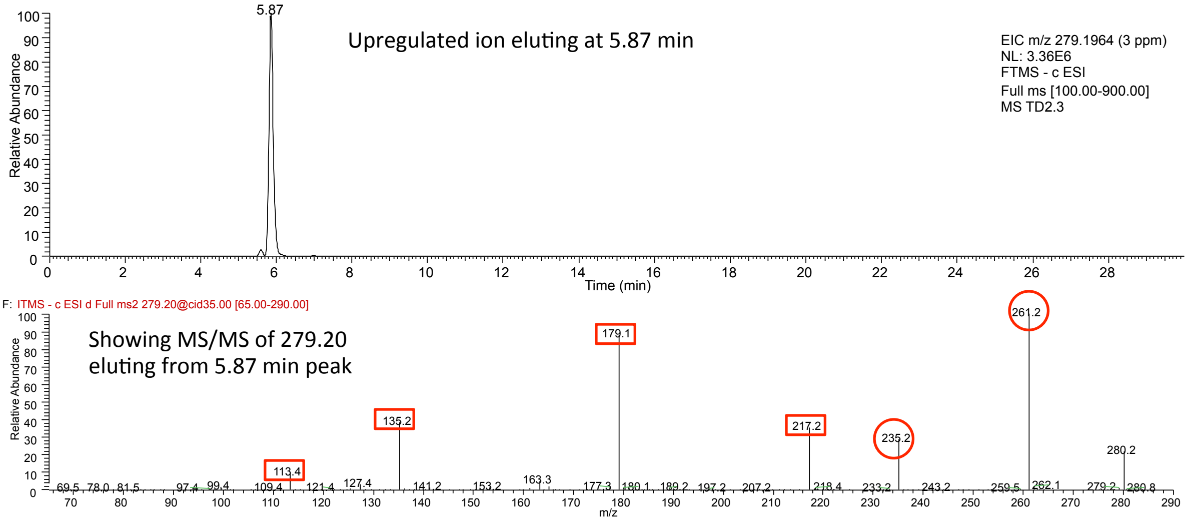


## Linoleic Acid m/z 279.2330 $C_{18}H_{31}O_2$

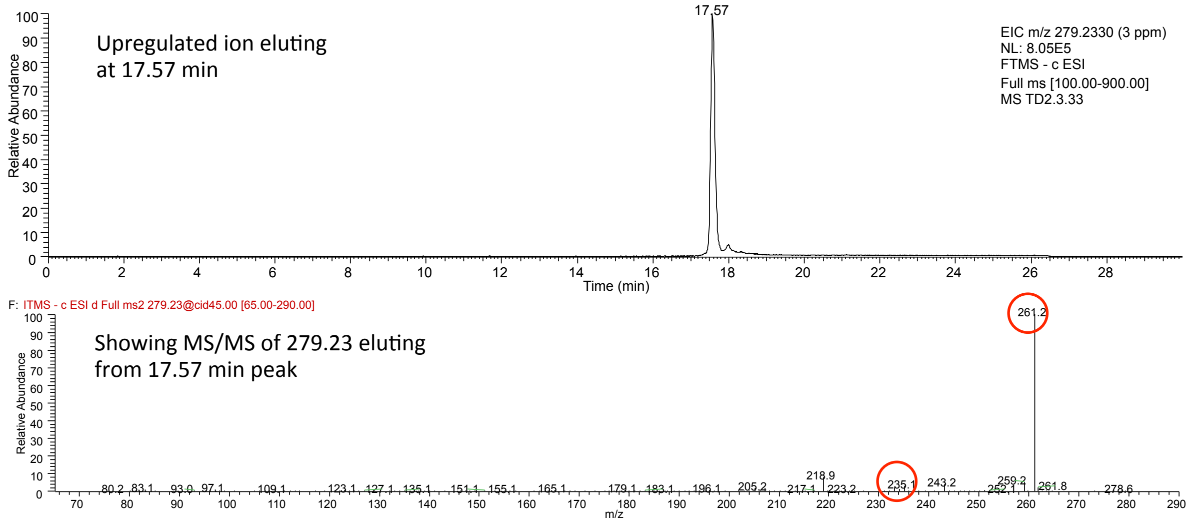

## 12-HHDE m/z 281.2125 $C_{17}H_{29}O_3$

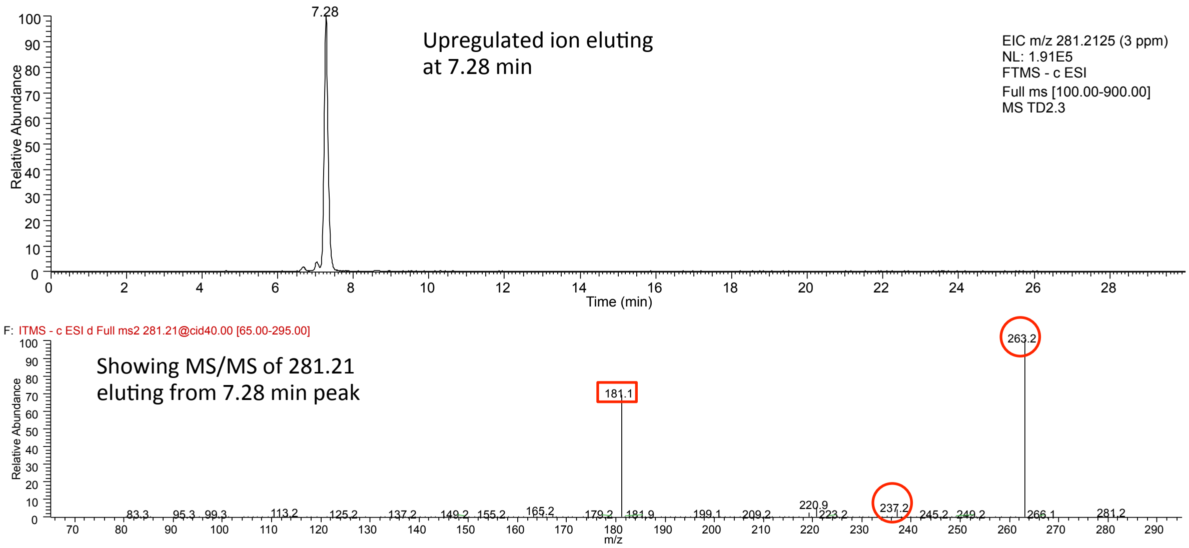


## Oleic acid m/z 281.2486 $C_{18}H_{33}O_2$

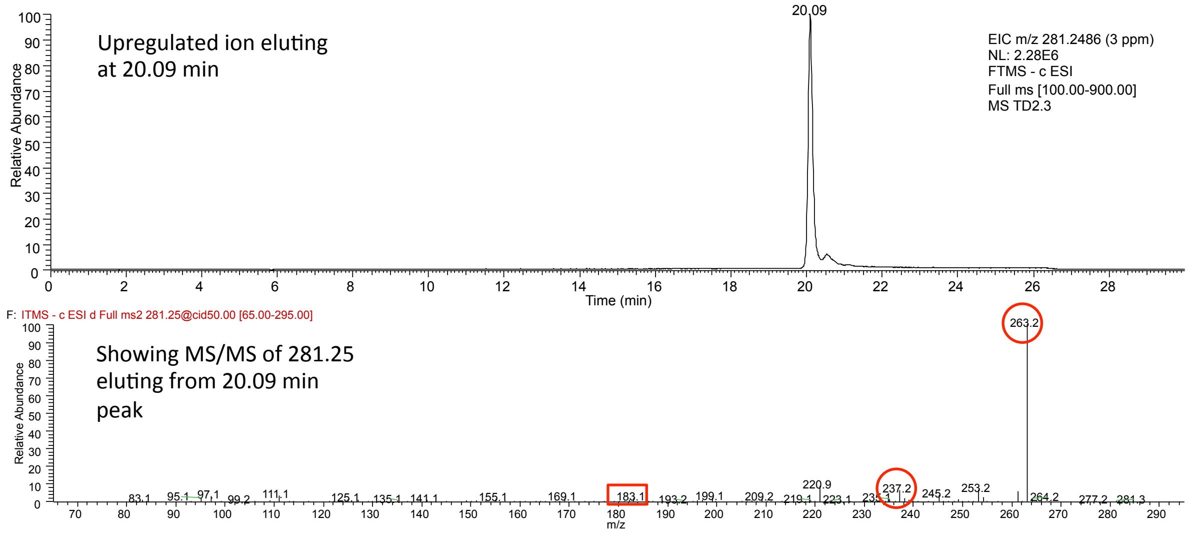


## Stearic acid m/z 283.2639 $C_{18}H_{35}O_2$

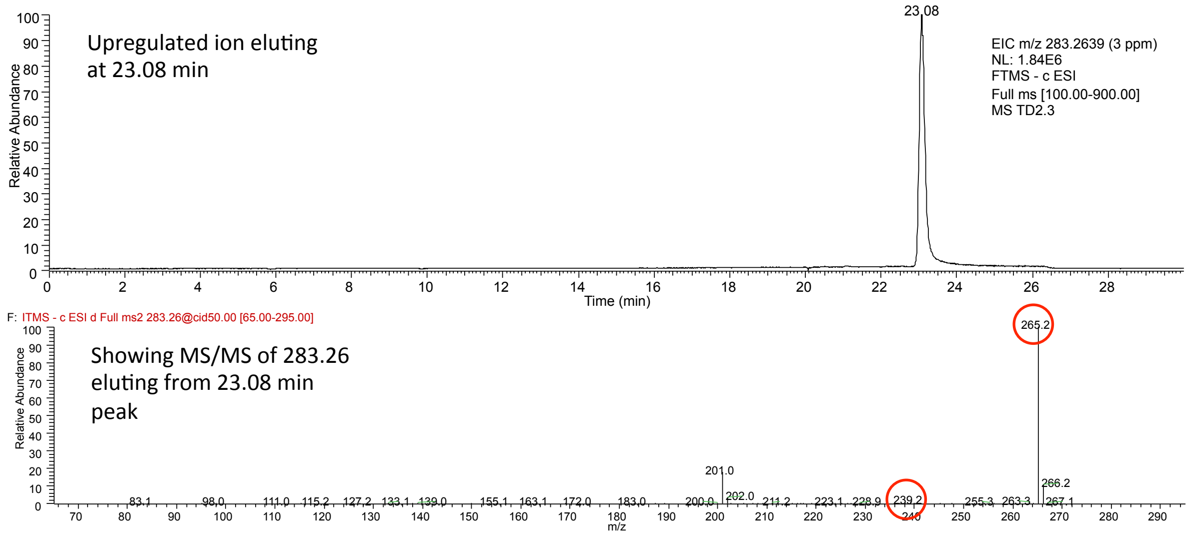

HOTE m/z 291.1969 $C_{18}H_{27}O_3$

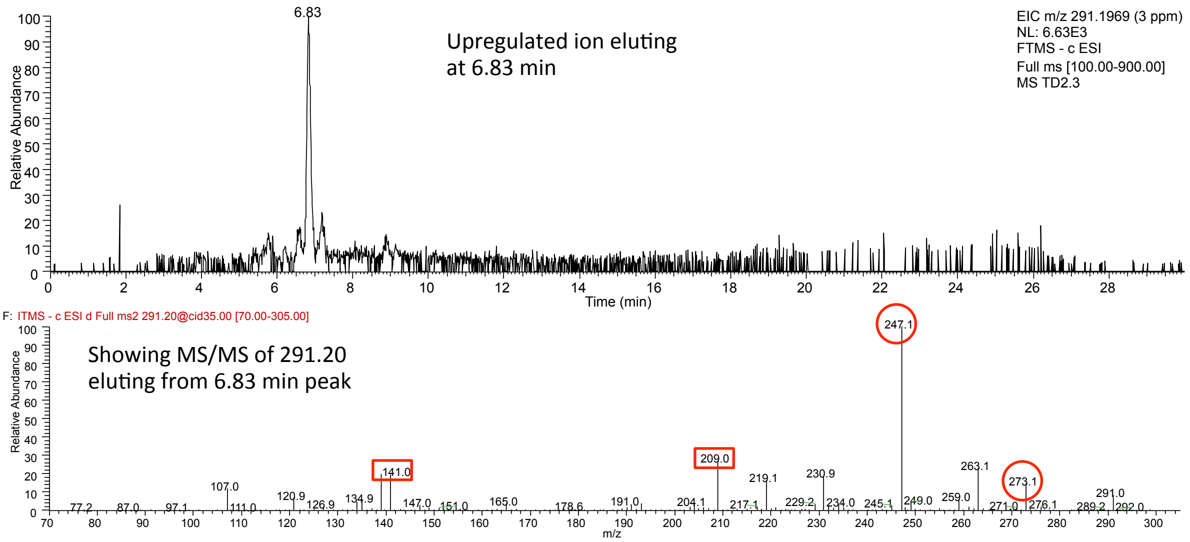


HOTrE m/z 293.2125 $C_{18}H_{29}O_3$

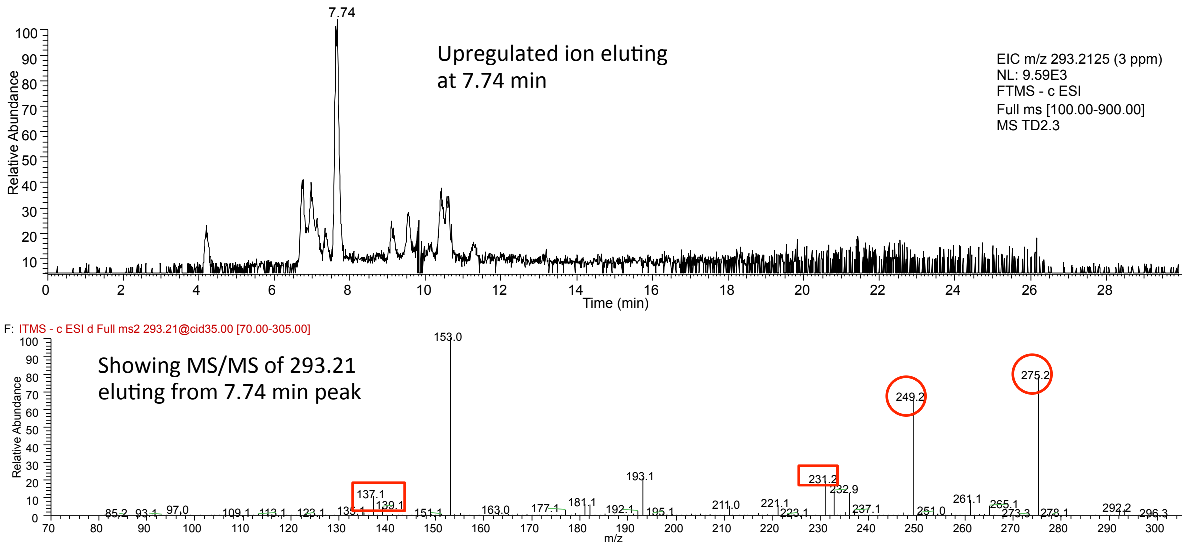


9/13-HODE mixture m/z 295.2279 $C_{18}H_{31}O_3$

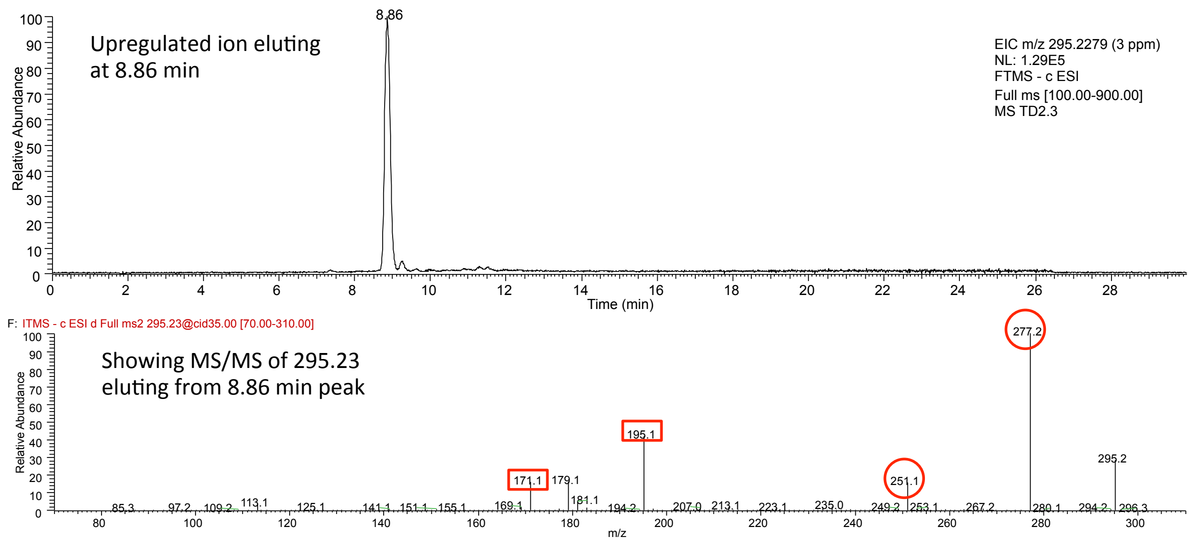

9-HOME m/z 297.2435 $C_{18}H_{33}O_3$

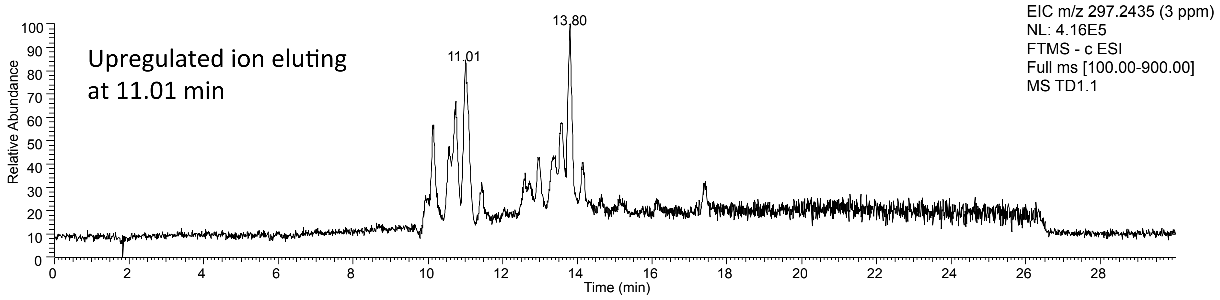


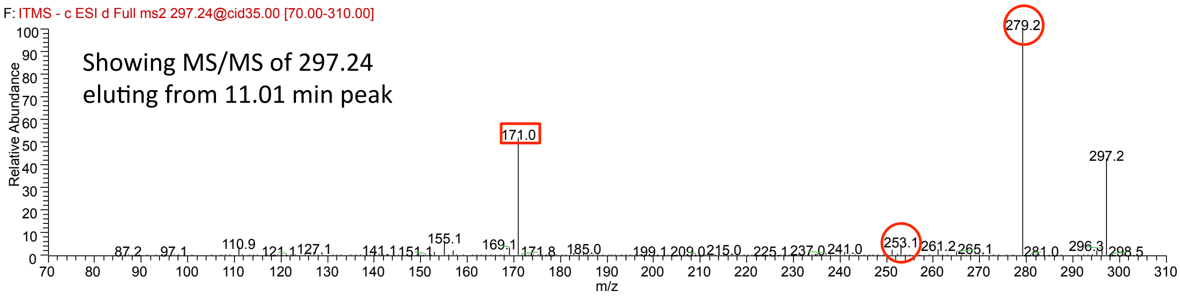

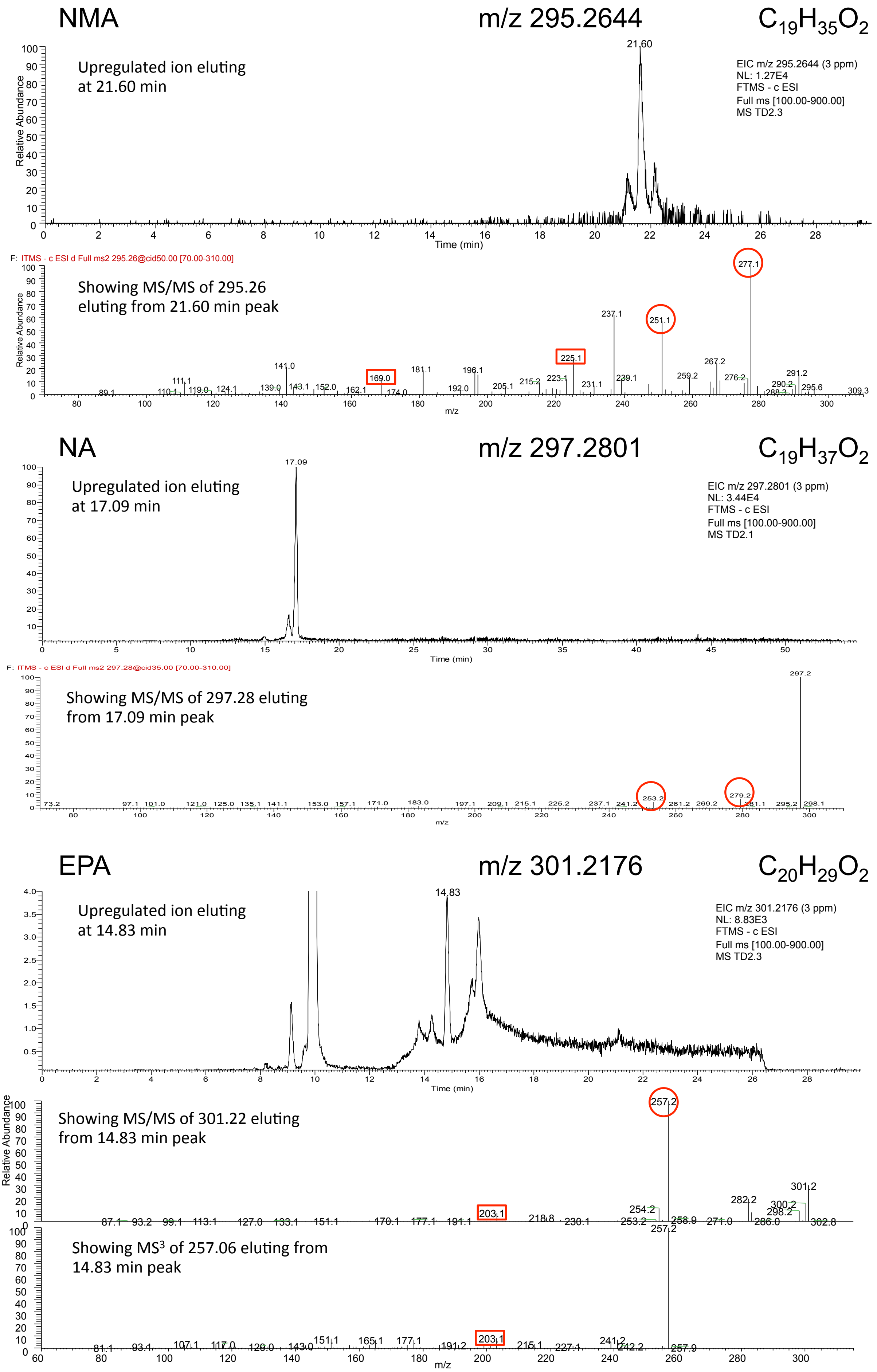
NMA
m/z 295.2644
$C_{19}H_{35}O_2$
Upregulated ion eluting at 21.60 min
EIC m/z 295.2644 (3 ppm)
NL: 1.27E4
FTMS - c ESI
Full ms [100.00-900.00]
MS TD2.3
Relative Abundance
Time (min)
F: ITMS - c ESI d Full ms2 295.26@cid50.00 [70.00-310.00]
Showing MS/MS of 295.26 eluting from 21.60 min peak
m/z
NA
m/z 297.2801
$C_{19}H_{37}O_2$
Upregulated ion eluting at 17.09 min
EIC m/z 297.2801 (3 ppm)
NL: 3.44E4
FTMS - c ESI
Full ms [100.00-900.00]
MS TD2.1
Time (min)
F: ITMS - c ESI d Full ms2 297.28@cid35.00 [70.00-310.00]
Showing MS/MS of 297.28 eluting from 17.09 min peak
m/z
EPA
m/z 301.2176
$C_{20}H_{29}O_2$
Upregulated ion eluting at 14.83 min
EIC m/z 301.2176 (3 ppm)
NL: 8.83E3
FTMS - c ESI
Full ms [100.00-900.00]
MS TD2.3
Time (min)
Relative Abundance
Showing MS/MS of 301.22 eluting from 14.83 min peak
Showing MS$^3$ of 257.06 eluting from 14.83 min peak
m/z

## Arachidonic acid m/z 303.2331 $C_{20}H_{31}O_2$

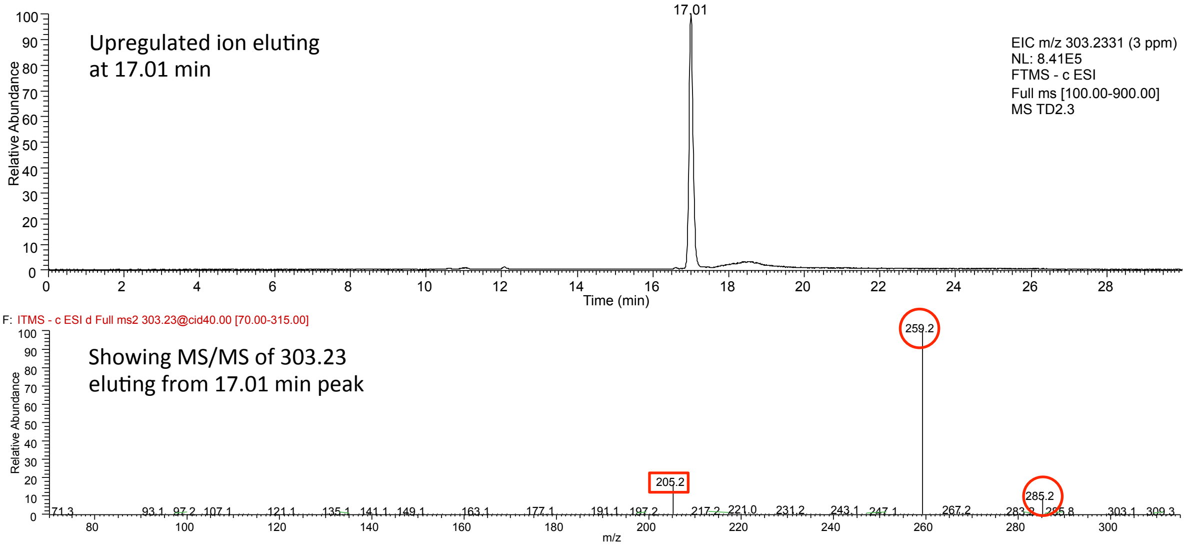


## ETrA isomers m/z 305.2486 $C_{20}H_{33}O_2$

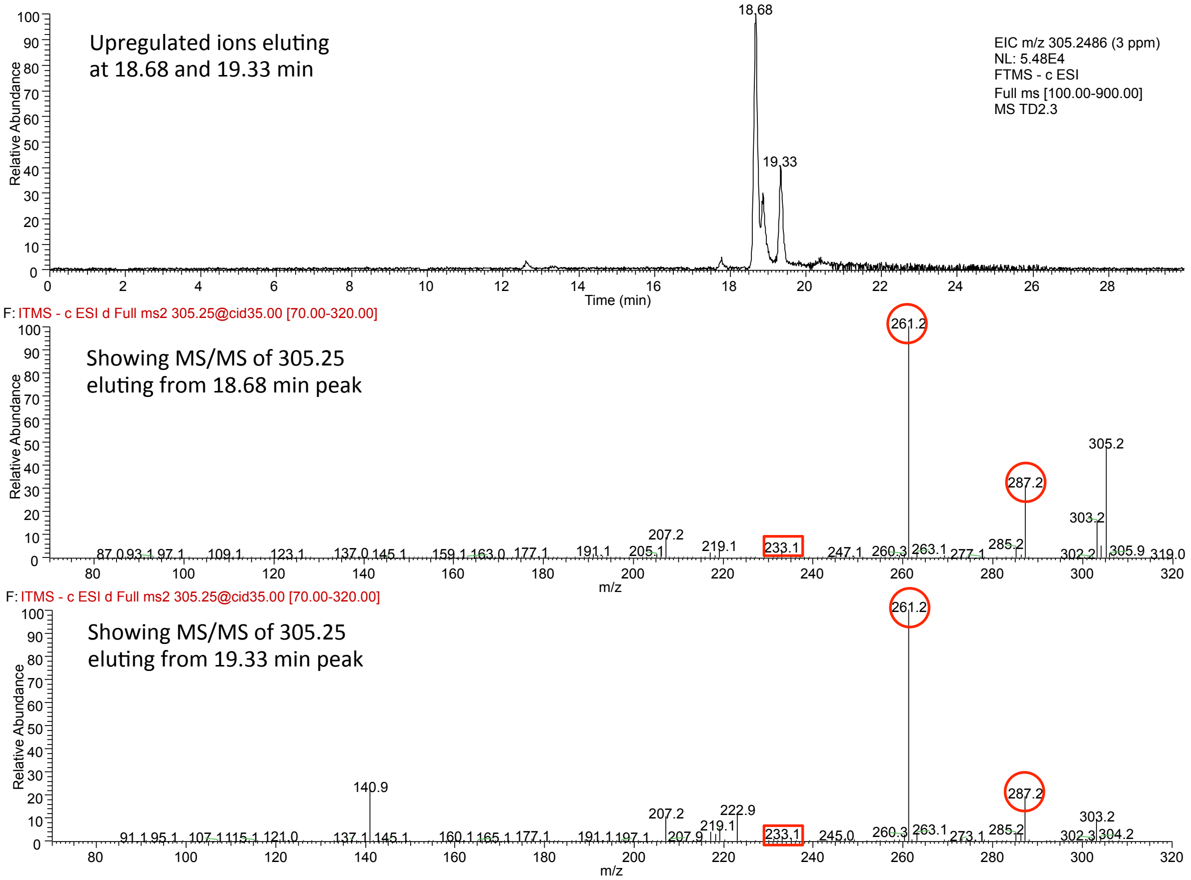

14-HNTE (7.86 min) and isomer    m/z 305.2126    $C_{19}H_{29}O_3$

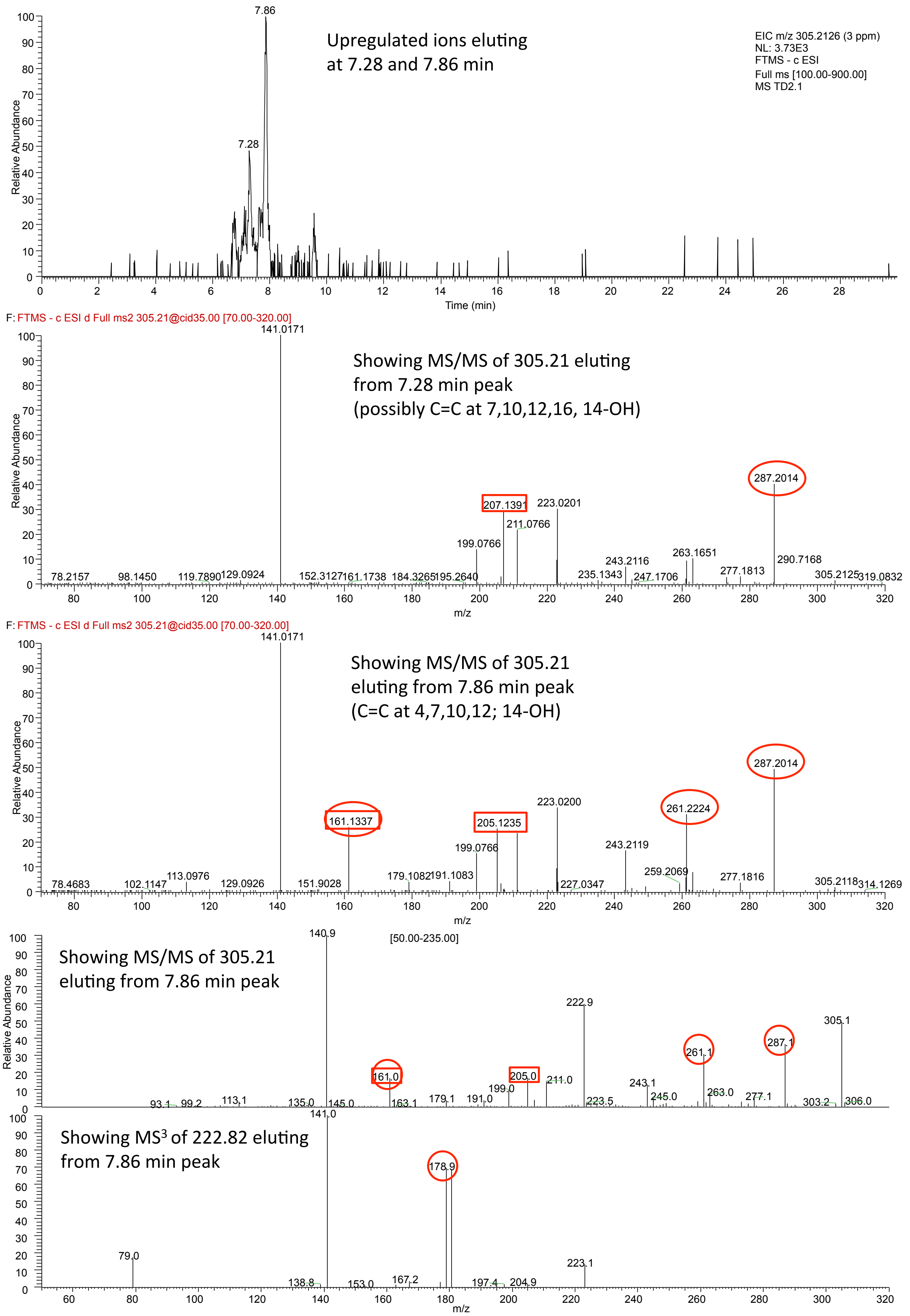

Di-HOTE-like m/z 307.1918 $C_{18}H_{27}O_4$

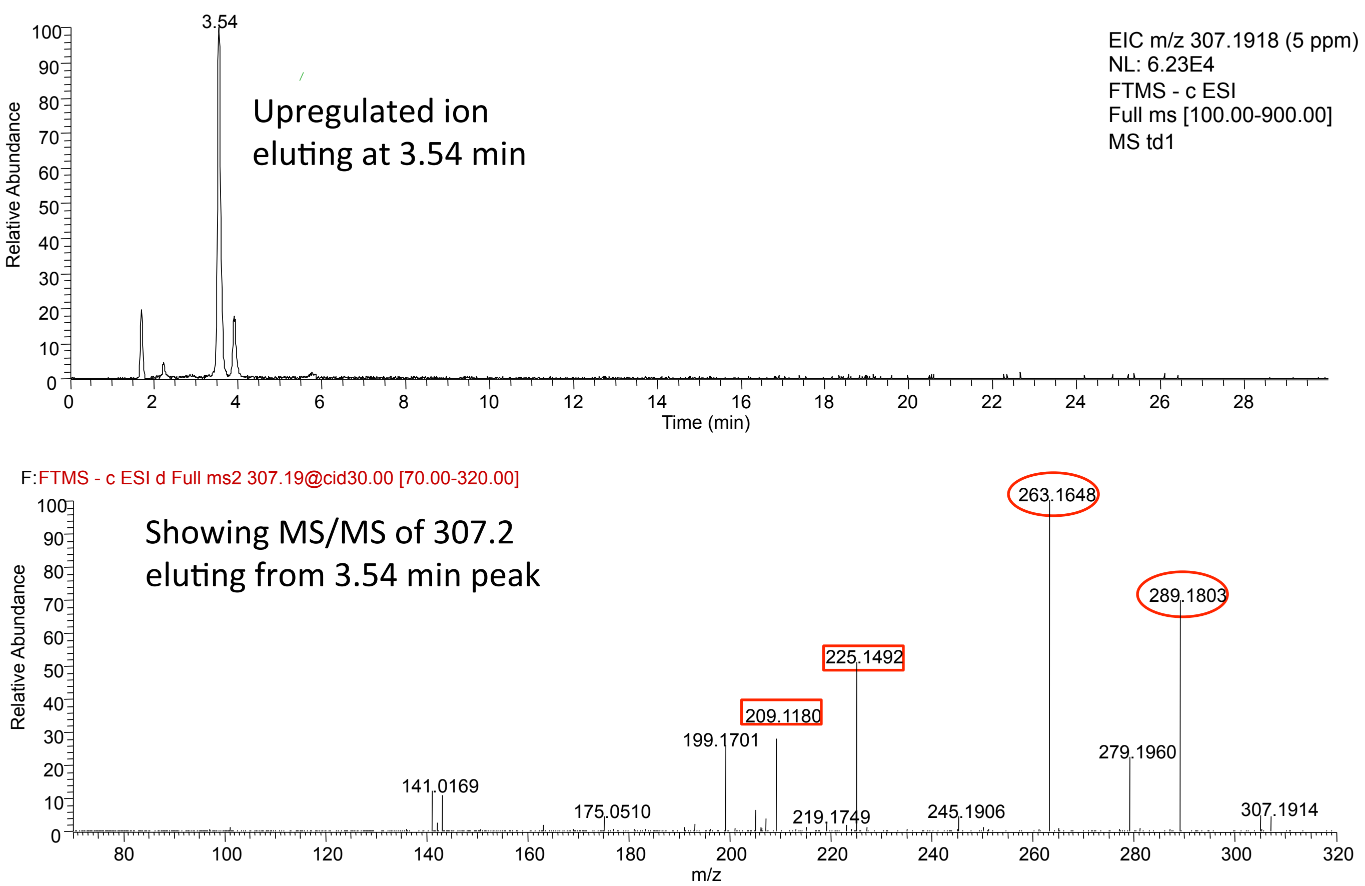

14-HNTrE m/z 307.2282 $C_{19}H_{31}O_3$

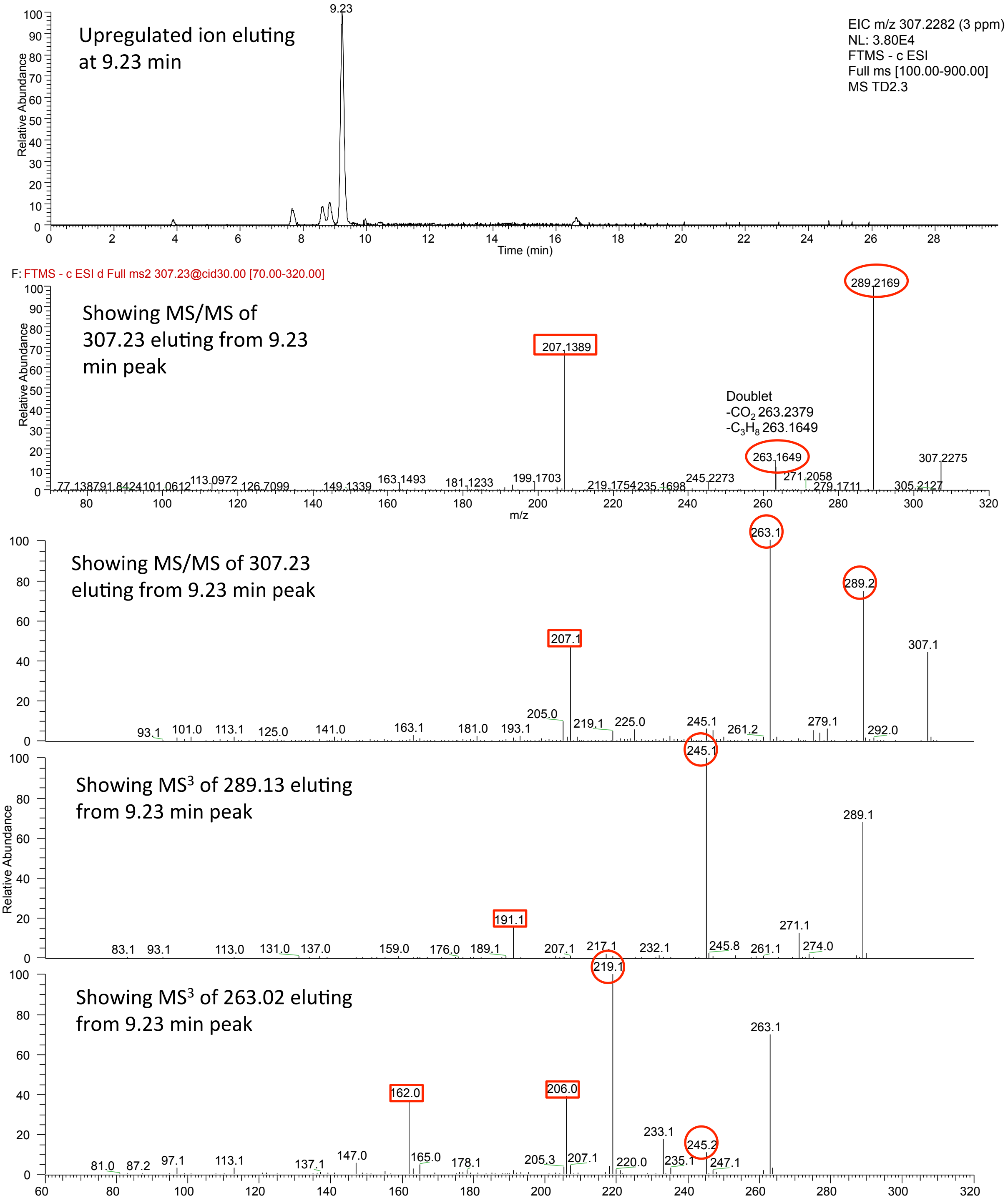

14-HNDE m/z 309.2438 $C_{19}H_{33}O_3$

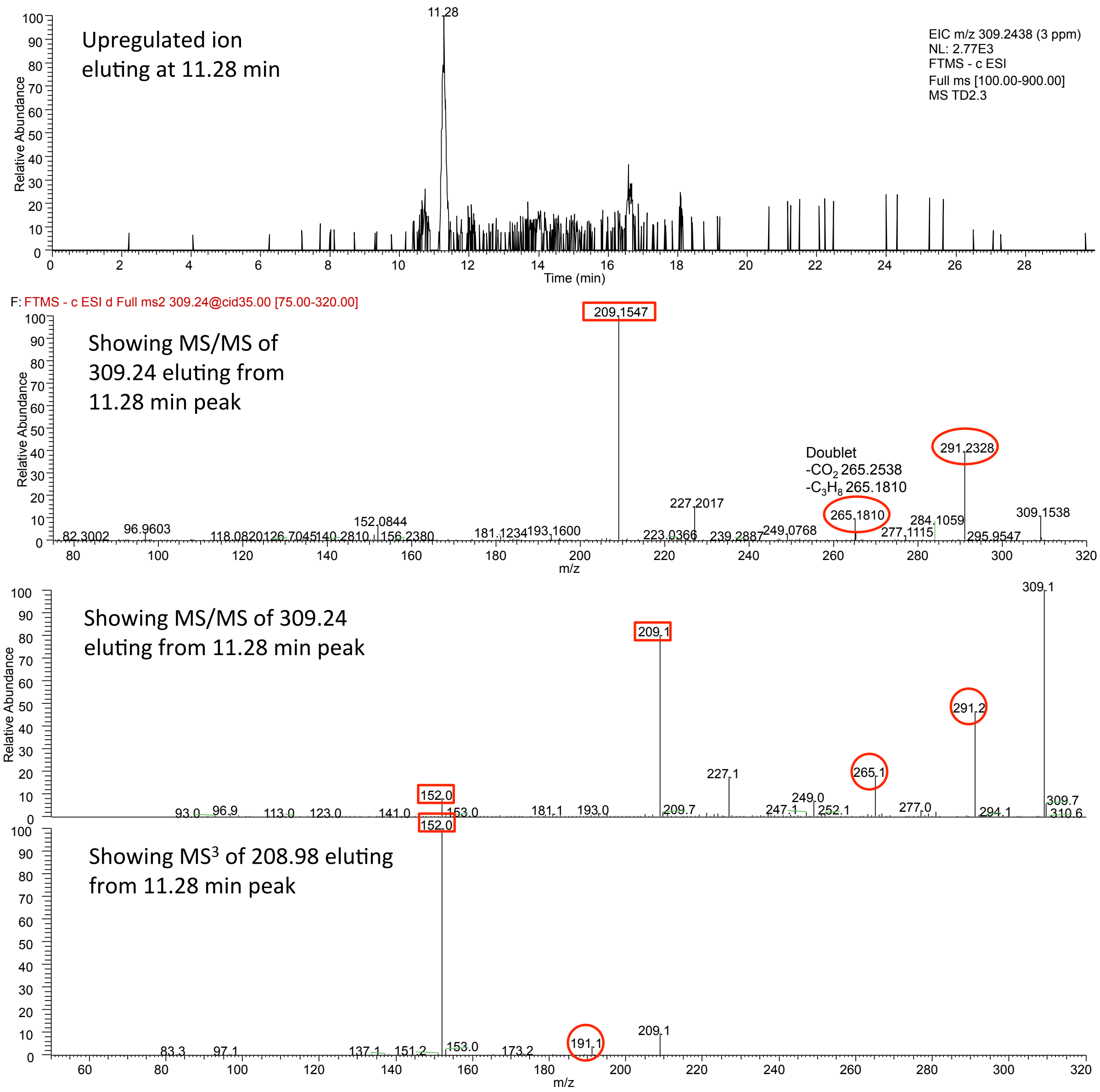

EDA m/z 307.2644 $C_{20}H_{35}O_2$

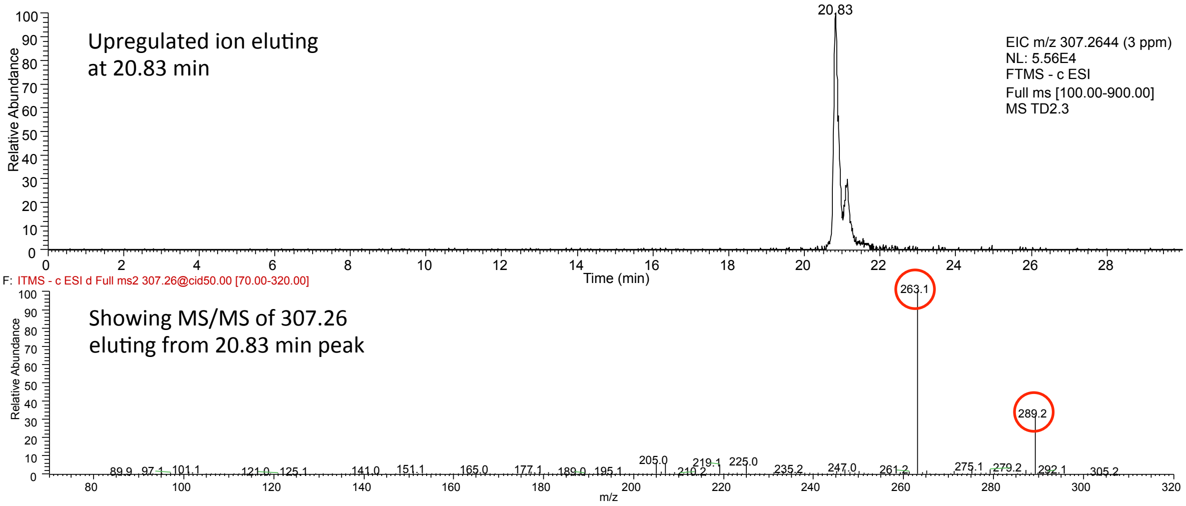


EMA m/z 309.2801 $C_{20}H_{37}O_2$

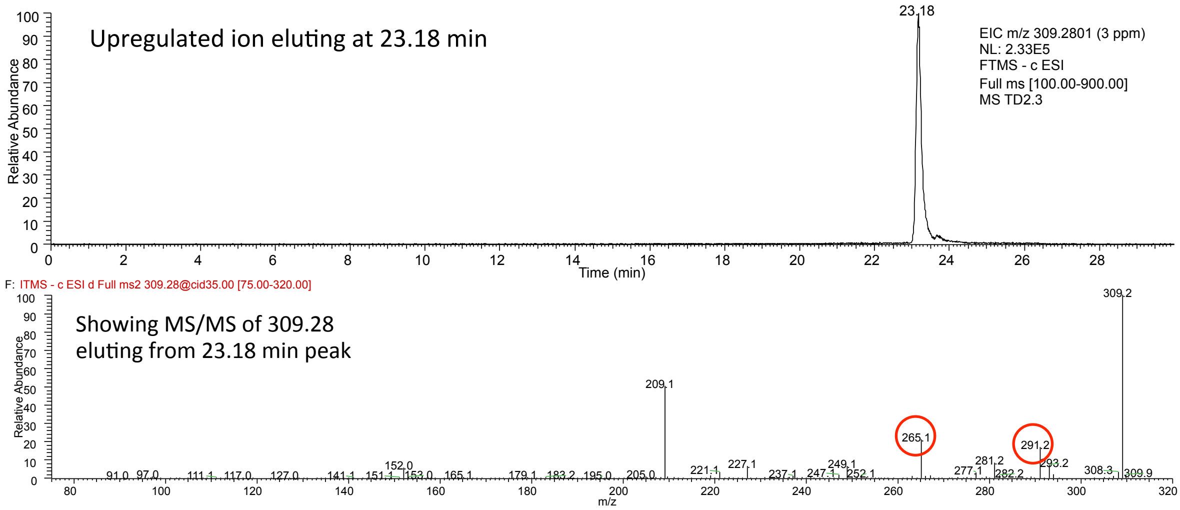


Arachidic acid m/z 311.2957 $C_{20}H_{39}O_2$

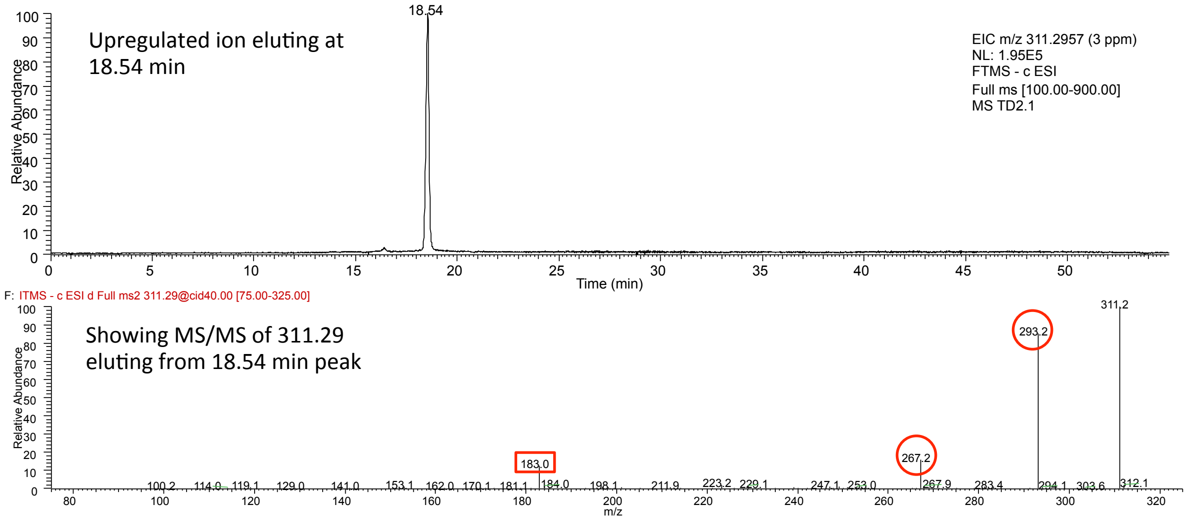

# In-source $PGE_2$ /$D_2$fragmentation* (2.04 , 2.23 min) $C_{20}H_{27}O_3$
# Prostaglandin $A_2$-like (4.07, 4.29 min) m/z 315.1970

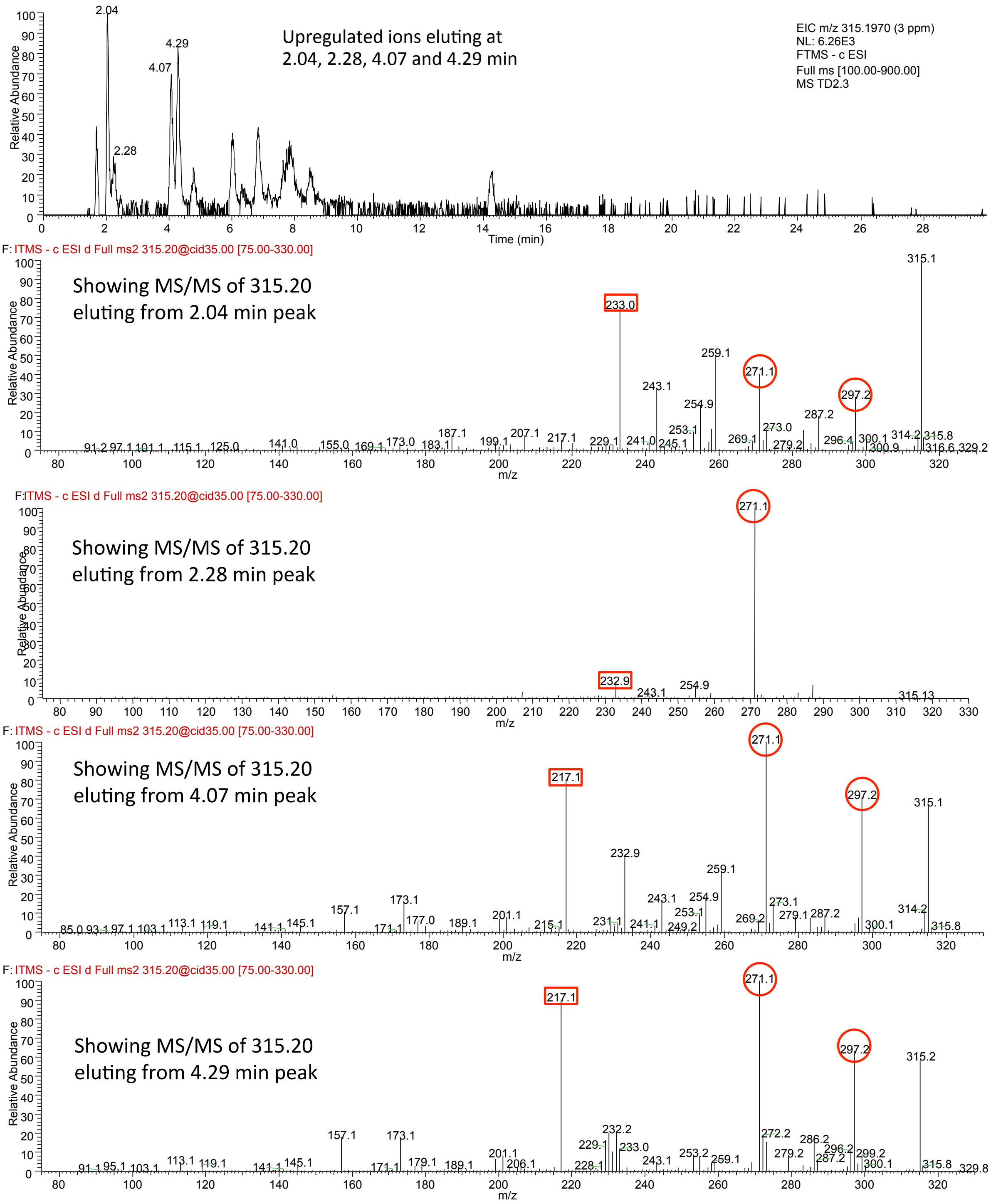


* $PGE_2$/$D_2$ are fragile molecules that fragment upon ionization, losing two water molecules. They are better detected at m/z 315.1970. In normal non-fragmentation runs, the peak at m/z 351.2180 is small compared to 315.1970, and these peaks superimpose at 2.03 and 2.28 min.

12-HEPE m/z 317.2124 $C_{20}H_{29}O_3$

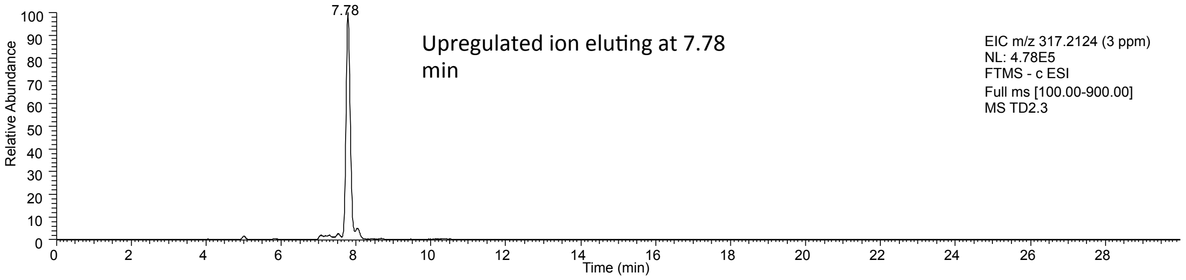


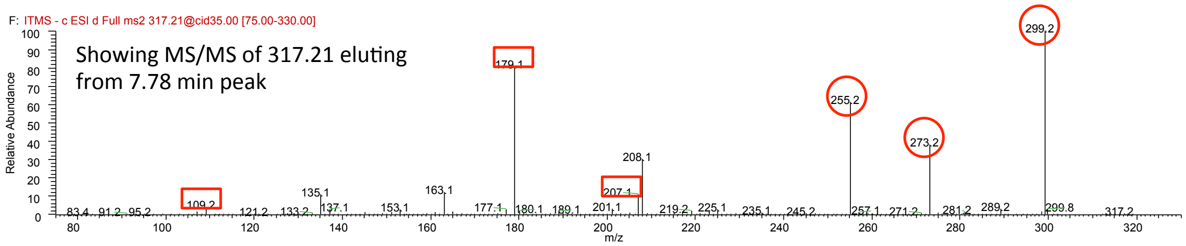

## 12-HETE m/z 319.2278 $C_{20}H_{31}O_3$

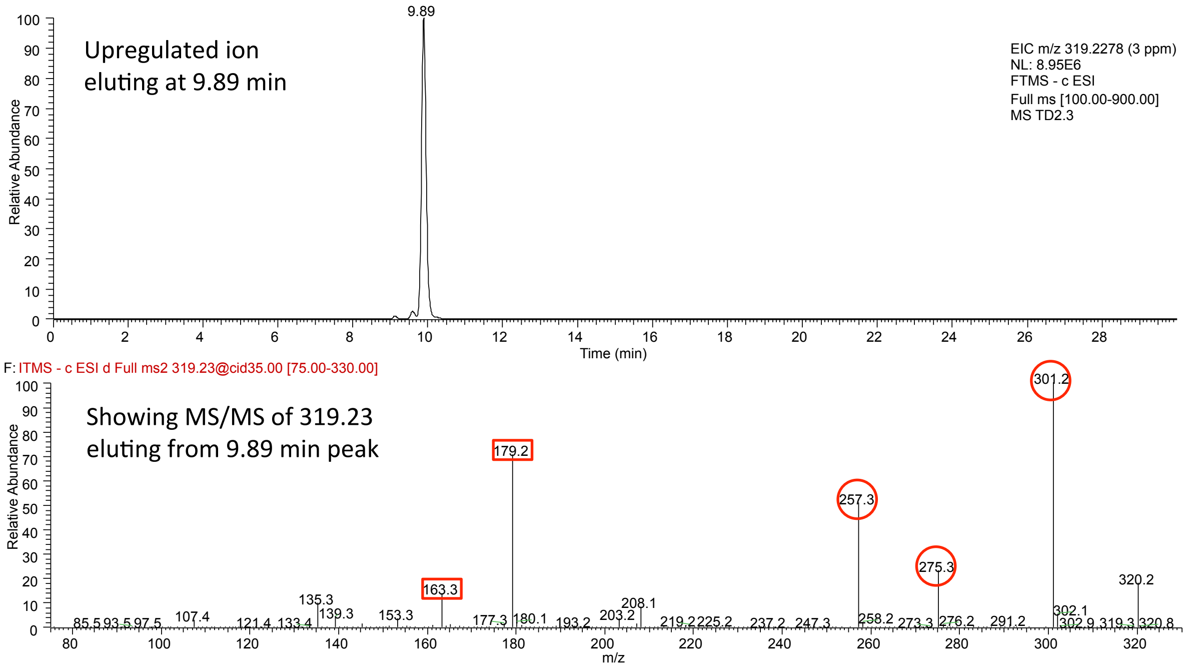


## 12-HETrE isomers m/z 321.2438 $C_{20}H_{33}O_3$

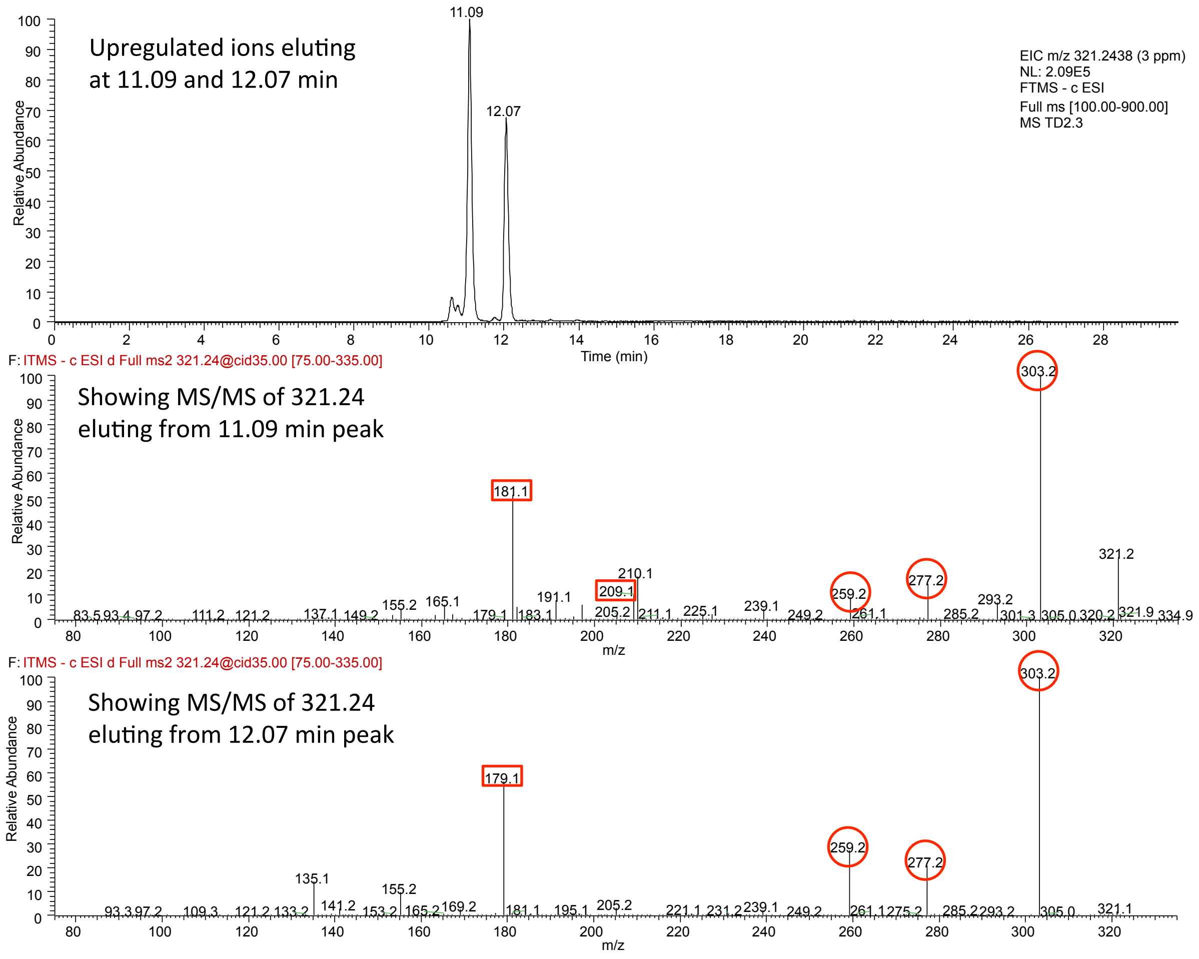

## 11-HEDE isomers m/z 323.2595 $C_{20}H_{35}O_3$

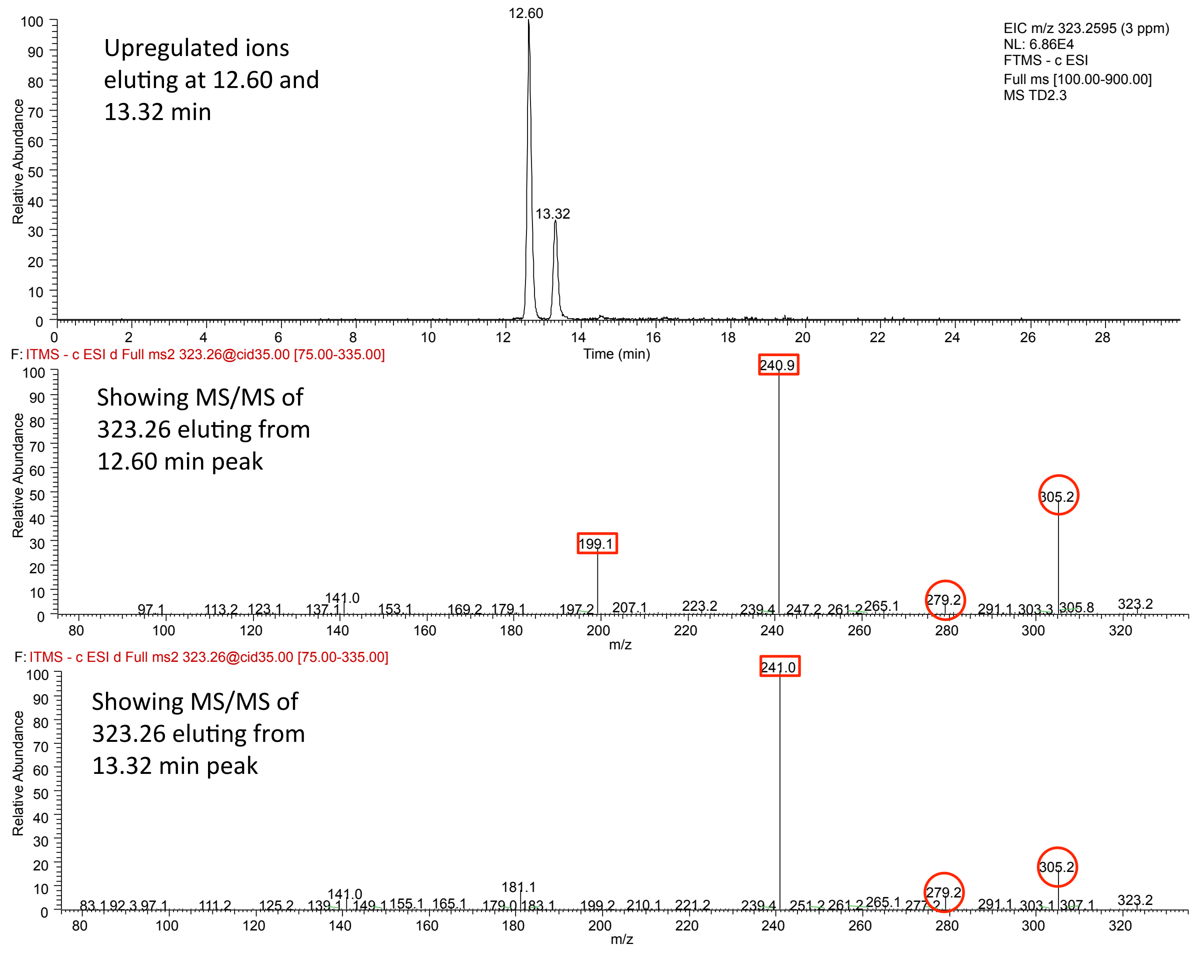


## DHA m/z 327.2334 $C_{22}H_{31}O_2$

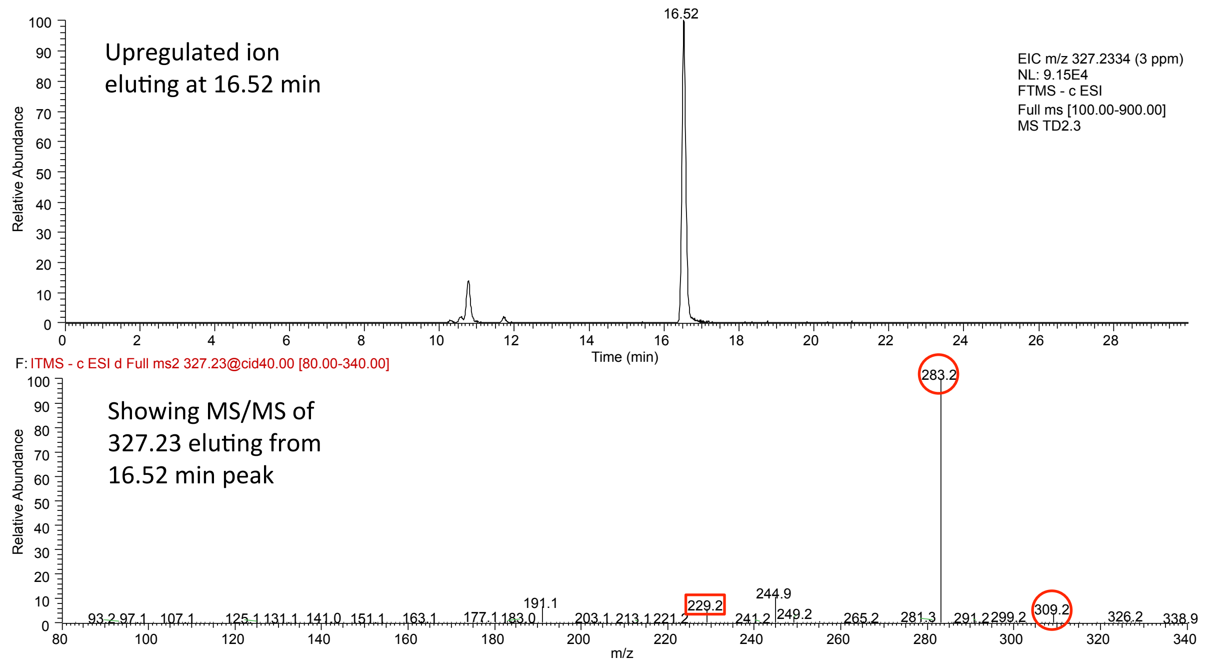

DPA (17.80 min)  m/z 329.2486  $C_{22}H_{33}O_2$
DPA-like (18.51 min)

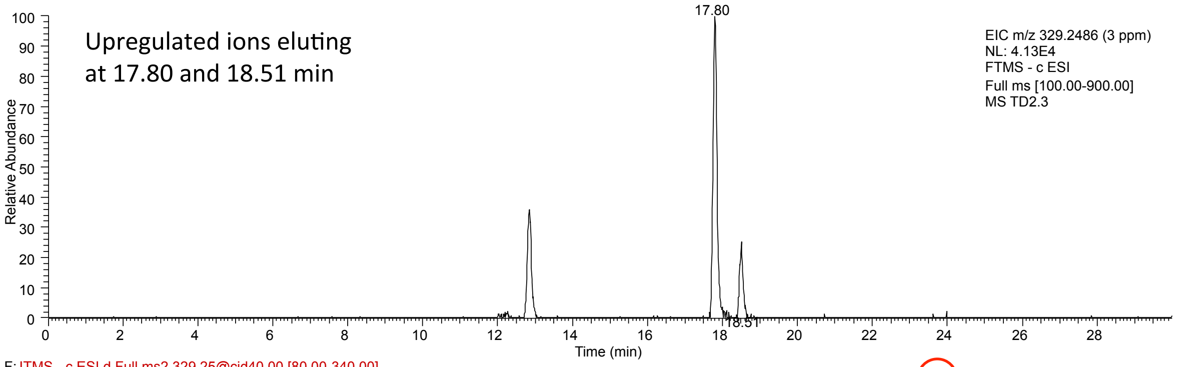


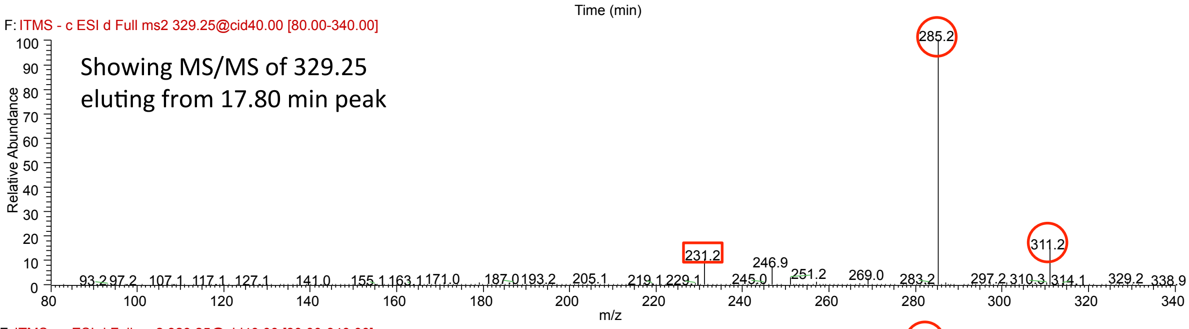


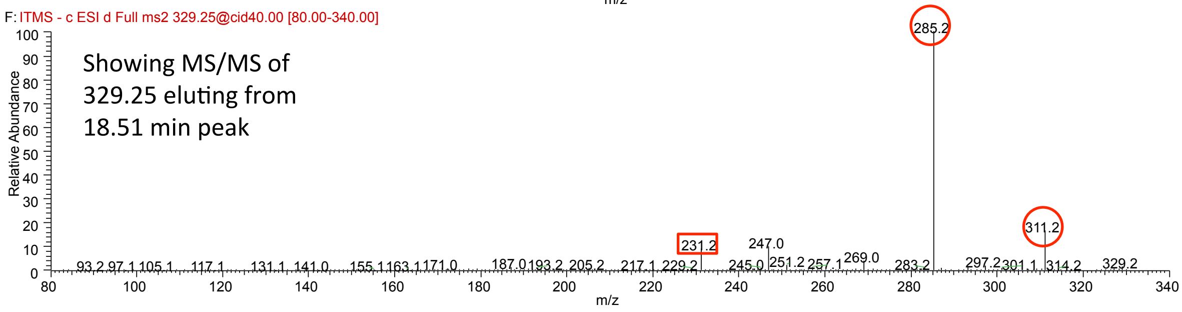


Adrenic acid  m/z 331.2640  $C_{22}H_{35}O_2$

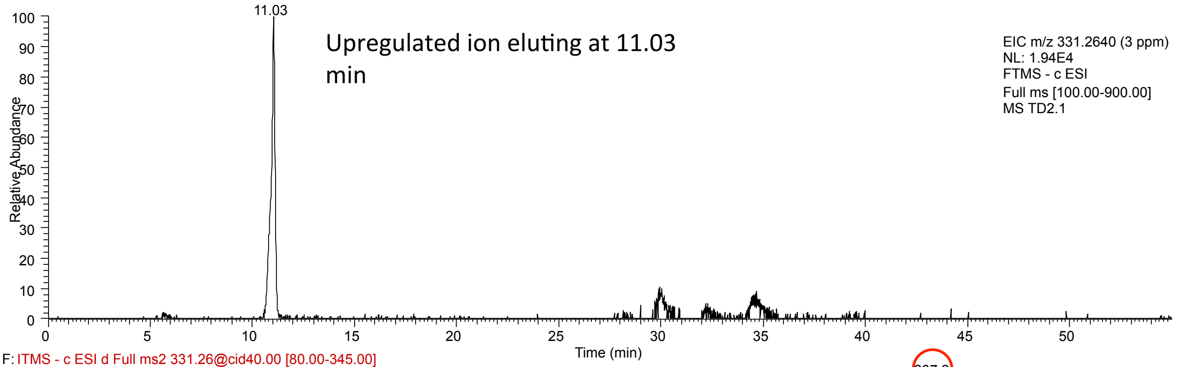


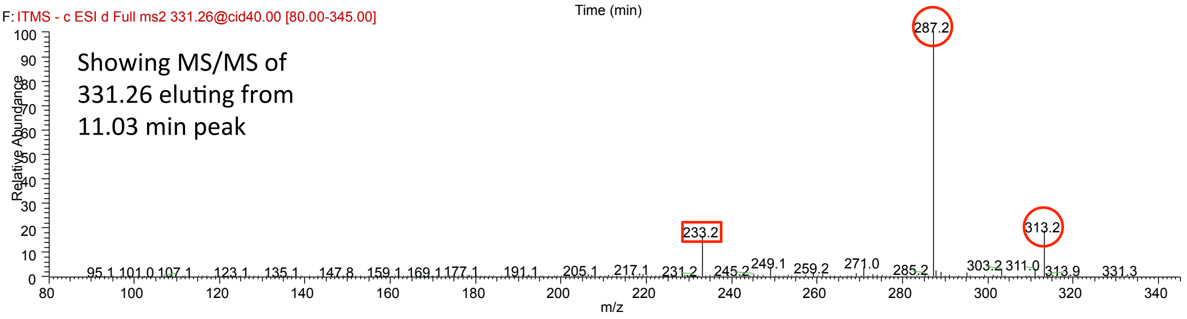

## PGB2-like (4.29, 4.80 min) Unknowns (5.15, 5.95 min)    m/z 333.2073    $C_{20}H_{29}O_4$

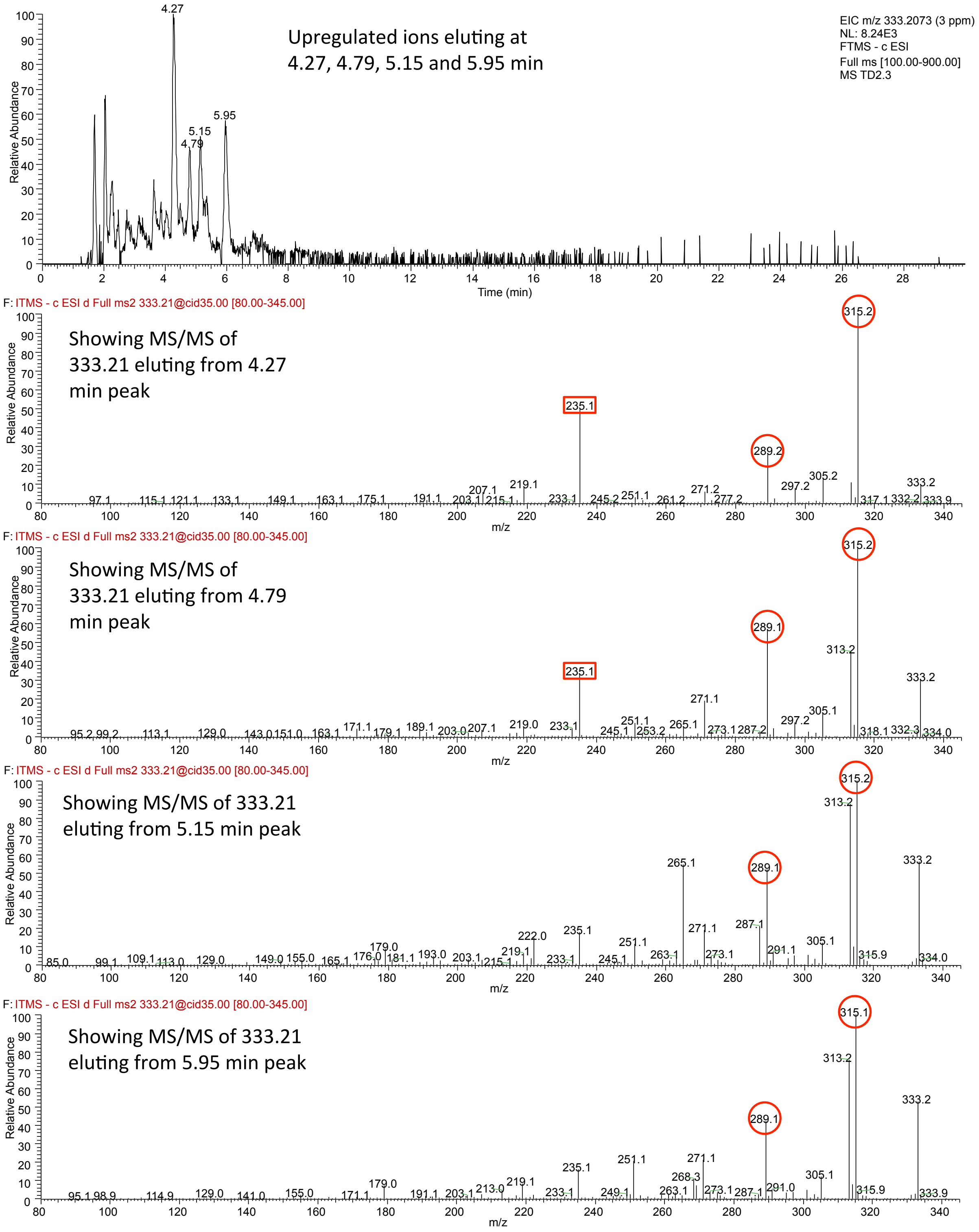

DTrA isomers m/z 333.2800 $C_{22}H_{37}O_2$

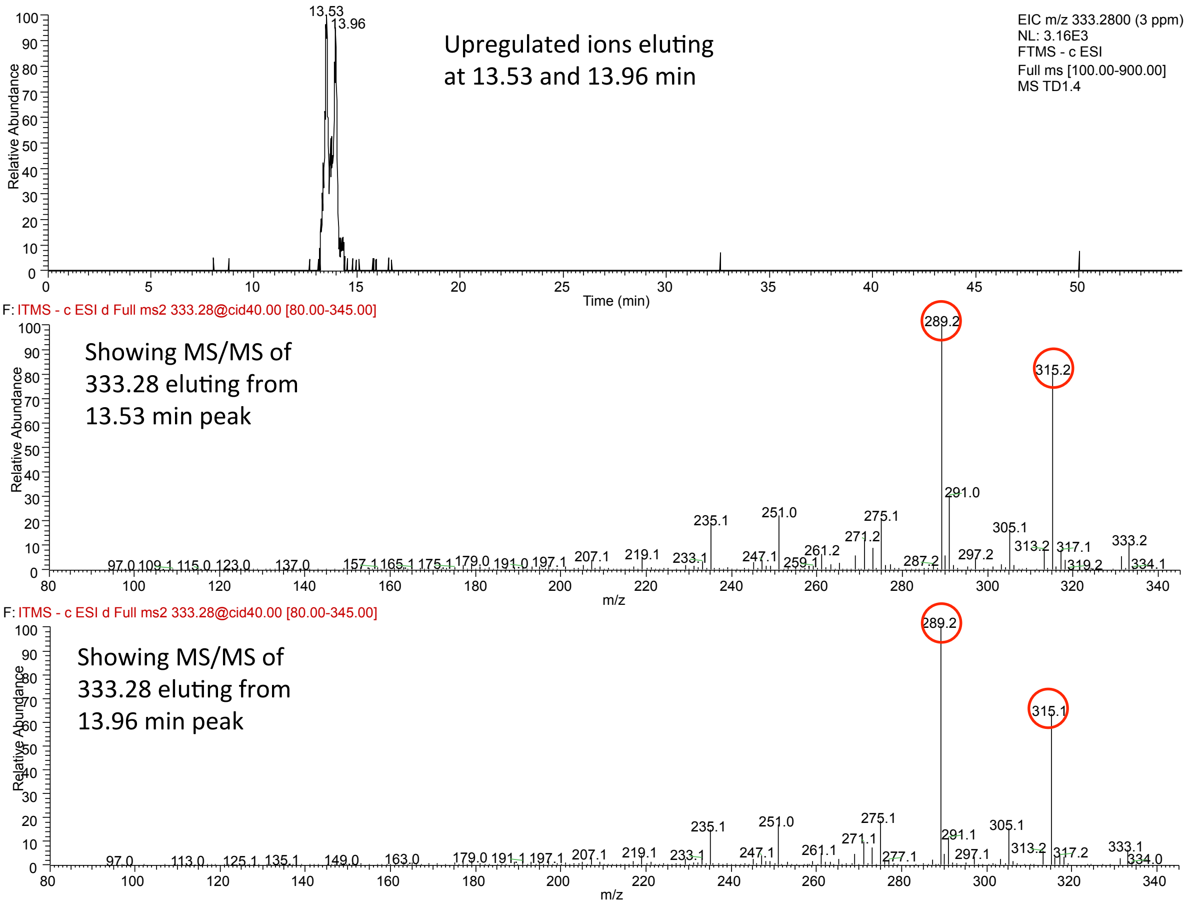

5,15-Di-HETE-isomers (4.15,4.48 min) m/z 335.2230 $C_{20}H_{31}O_4$
Prostaglandin-$A_1$-like (4.97,5.69 min)
Hepoxilin-$B_3$-like (7.14 min)

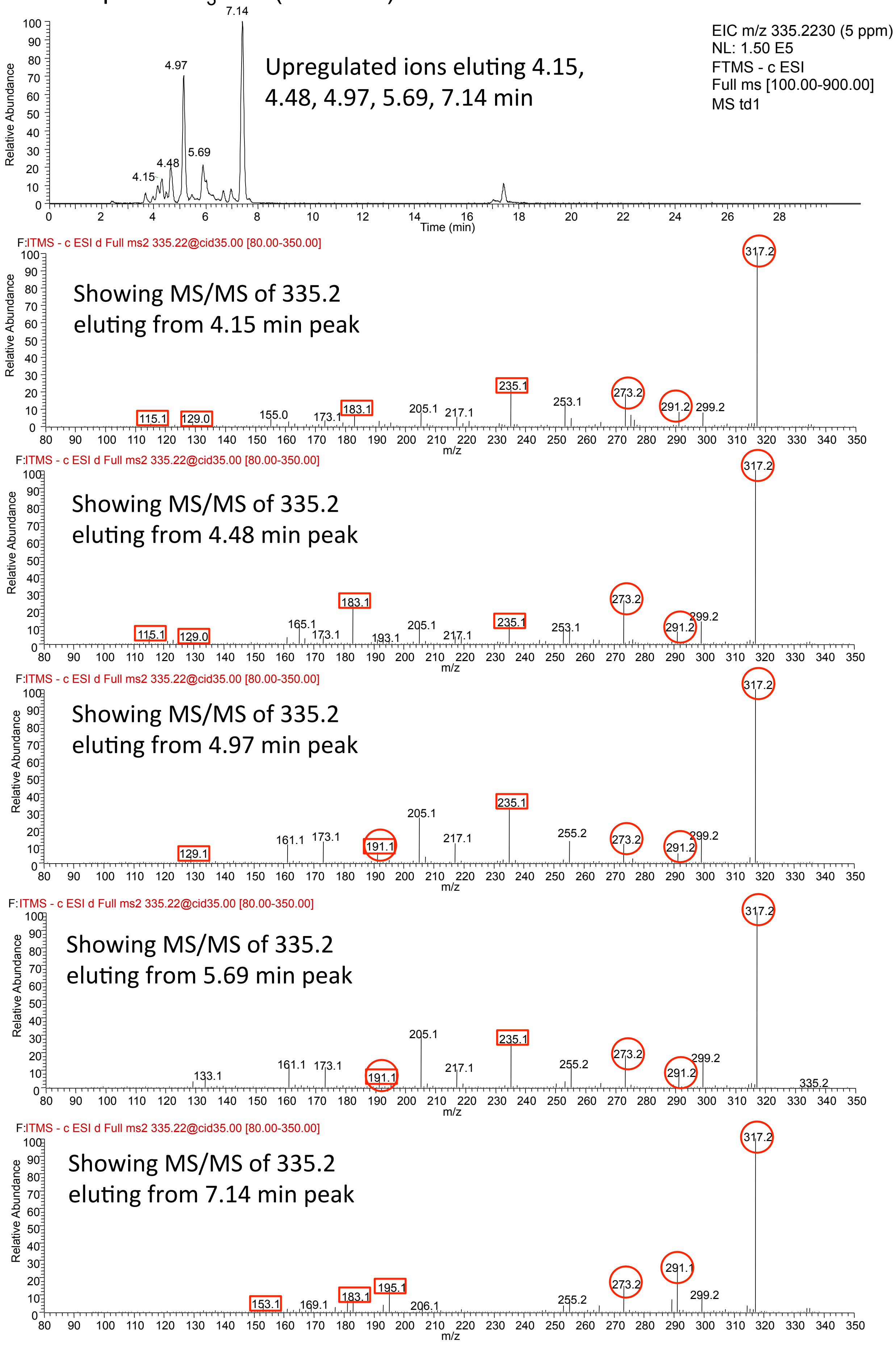

DDA m/z 335.2955 $C_{22}H_{39}O_2$

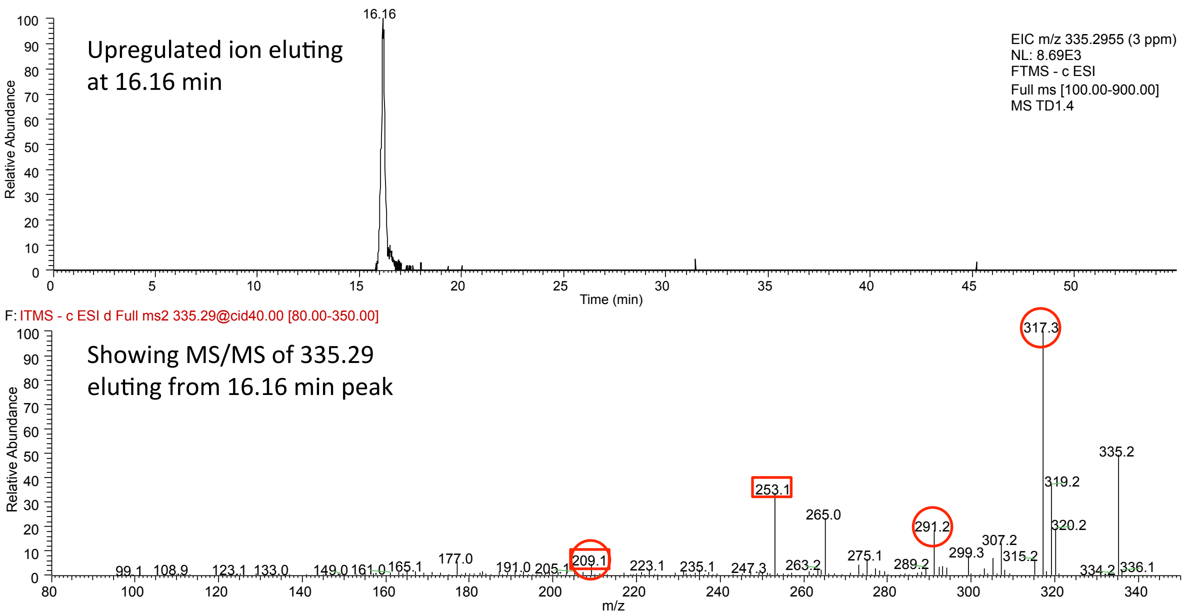

## DiHETrE-like (4.86, 6.08min) m/z 337.2386 $C_{20}H_{33}O_4$
## Deoxy-ProstaglandinA$_1$-like (6.74 min)

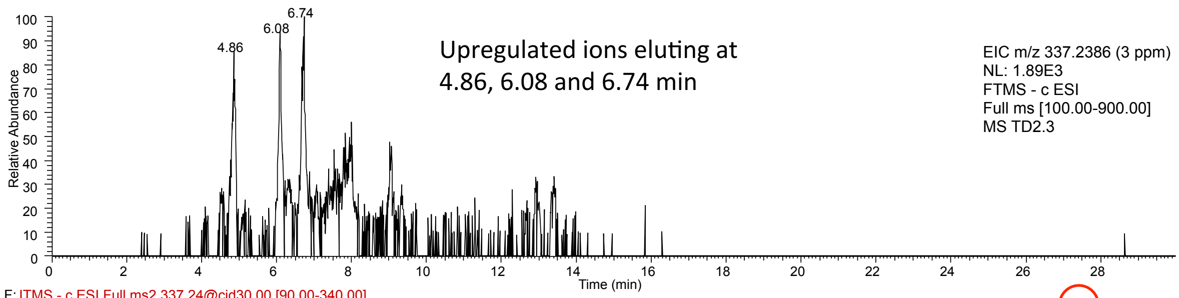


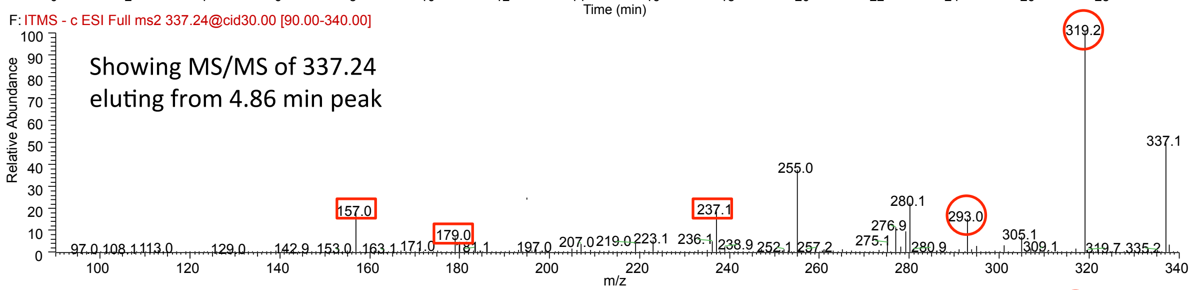


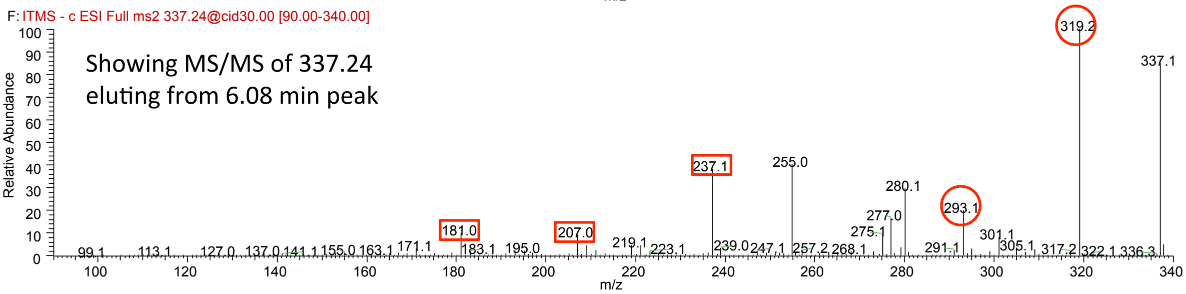


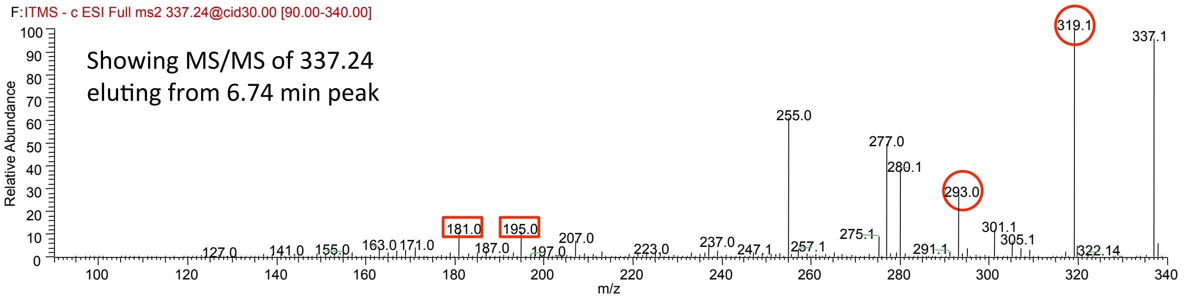


## Unknown m/z 341.2104 $C_{22}H_{29}O_3$

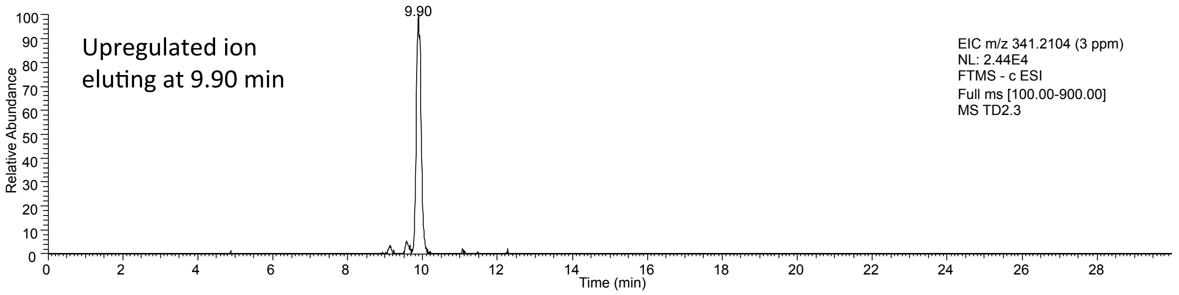


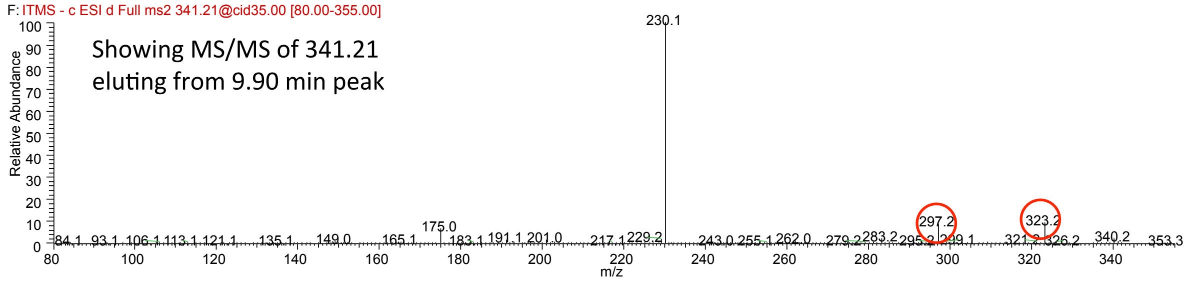

HDoHE mixture (8.98 min) m/z 343.2282 $C_{22}H_{31}O_3$
14-HDoHE (9.64 min)

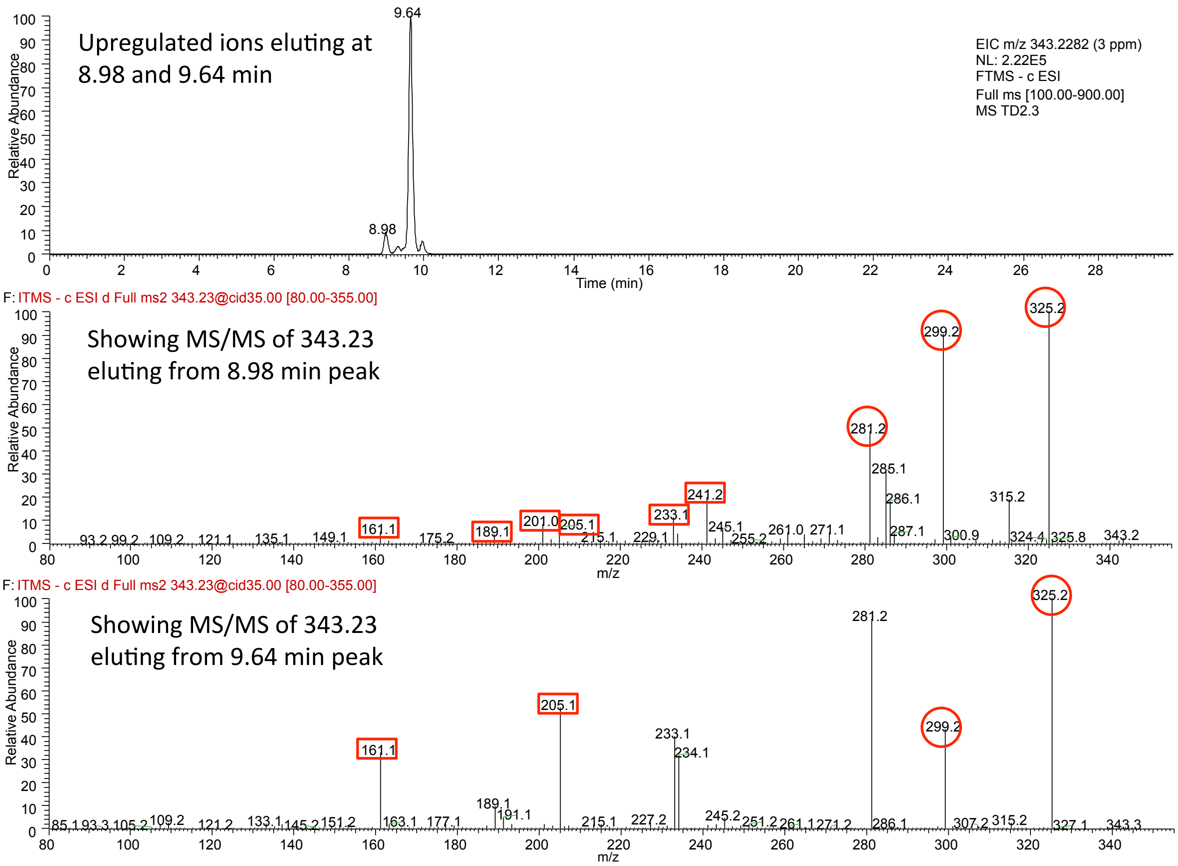

20-HDoPE (n-3), 10.30 min
17-HDoPE (n-3), 10.58 min
14-HDoPE (n-3), 10.78 min
14-HDoPE (n-6), 11.72 min

m/z 345.2438

$C_{22}H_{33}O_3$

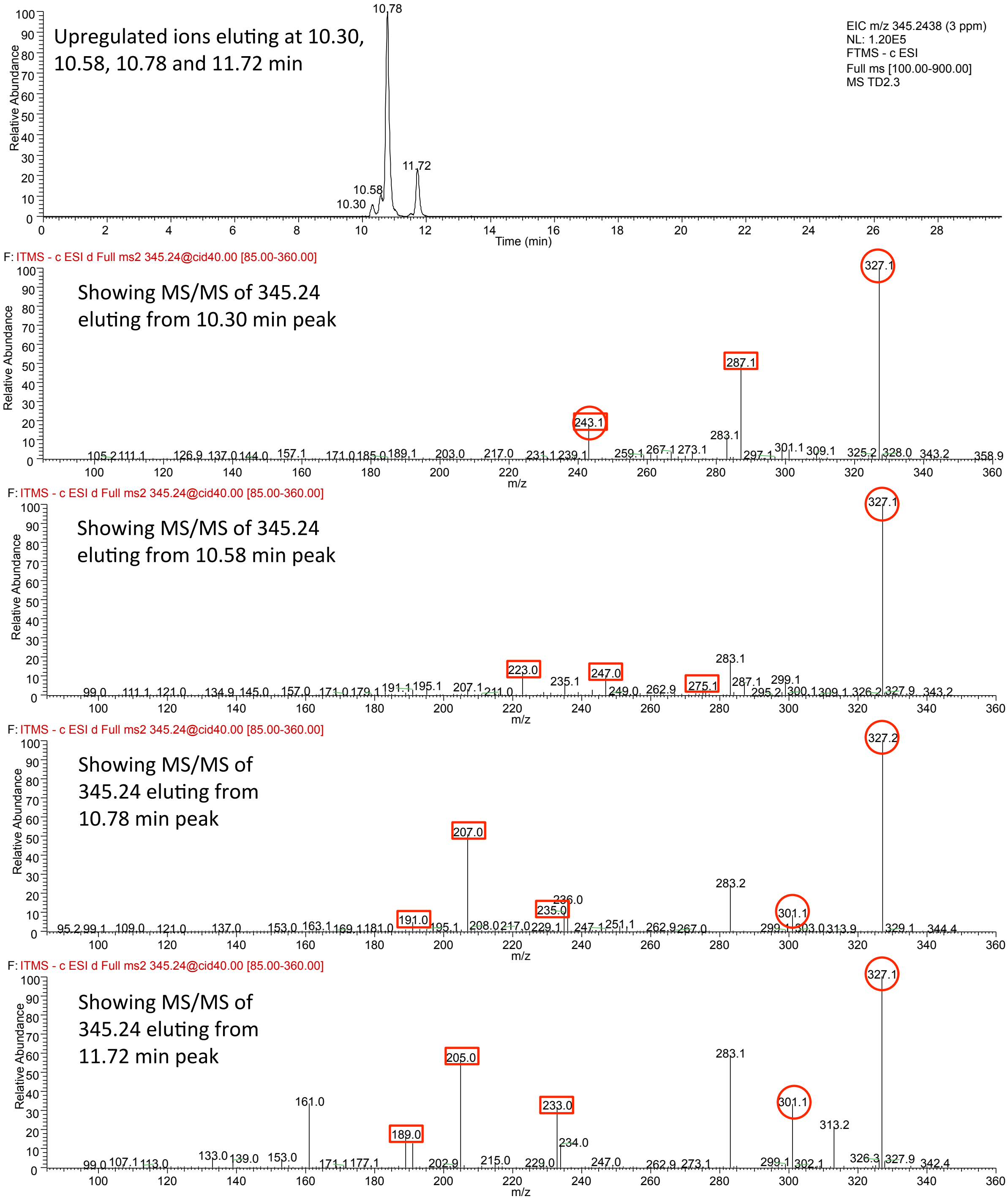

17-HDoTE (n-3), 12.26 min
13-HDoTE (n-3), 12.62 min
14-HDoTE (n-3), 12.84 min

m/z 347.2595

$C_{22}H_{35}O_3$

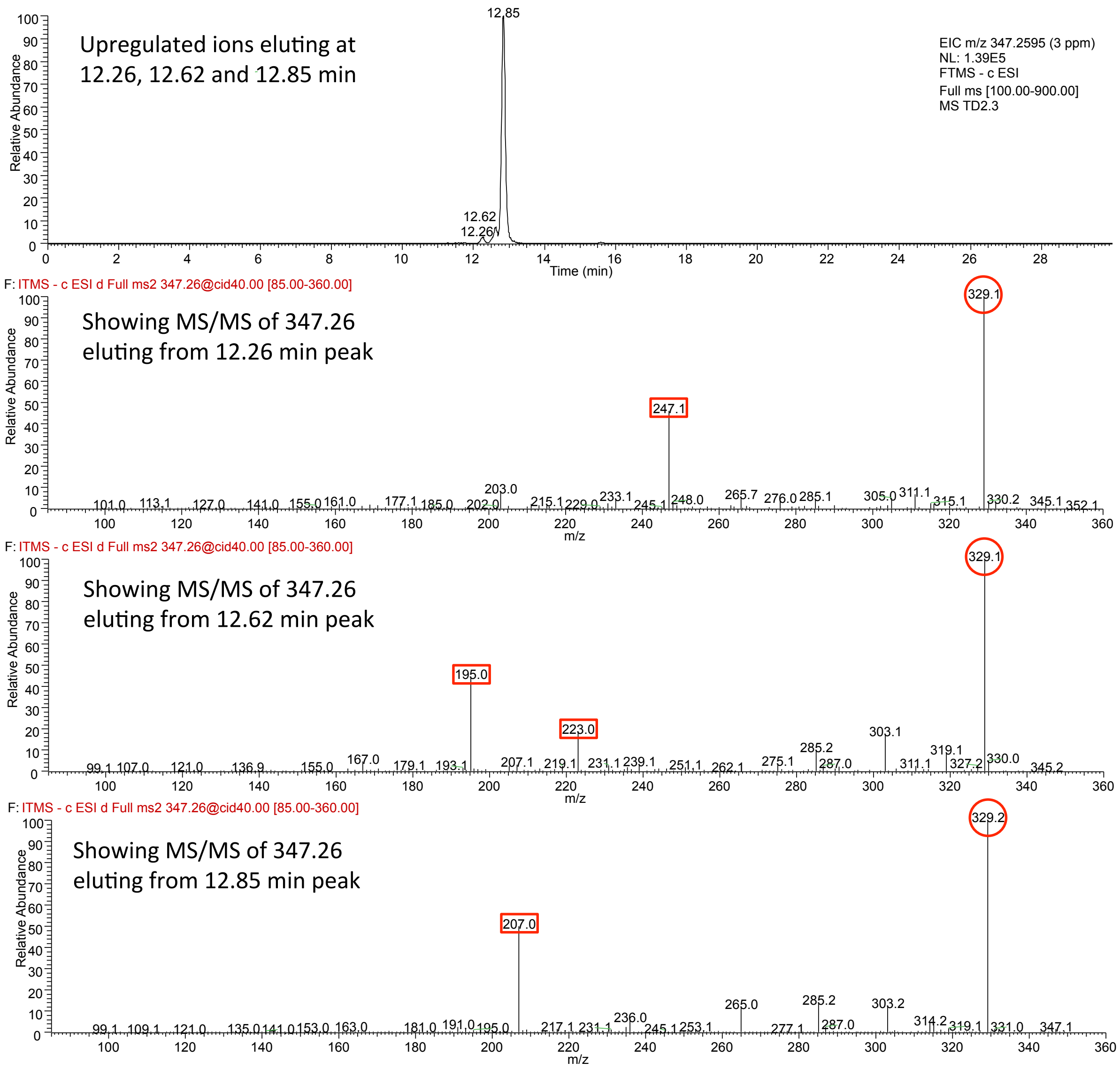

14-DoTrE (n-6), 14.56 min
14-DoTrE (n-9), 14.88 min

m/z 349.2752

$C_{22}H_{37}O_3$

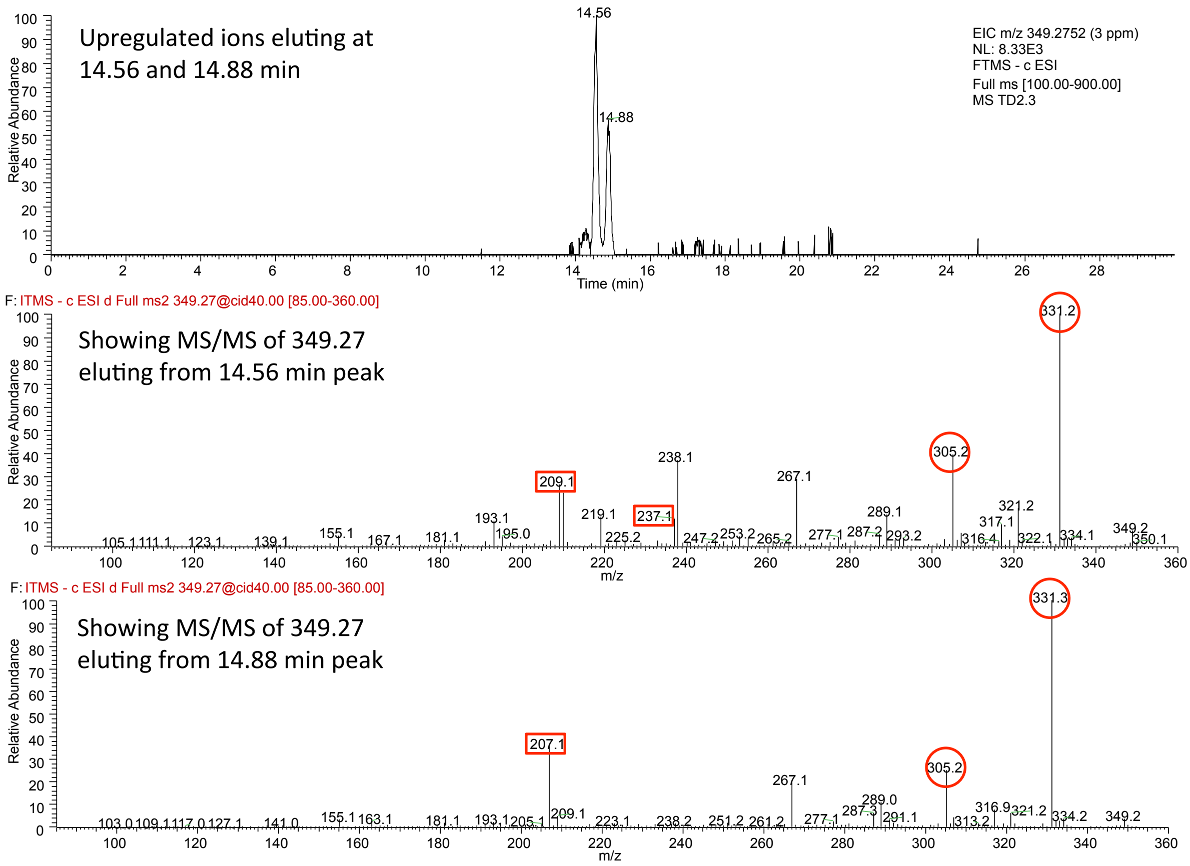

# Prostaglandin $E_2$ and $D_2$ (2.03, 2.28 min) m/z 351.2180 $C_{20}H_{31}O_5$

PGE2/D2 undergo in-source fragmentation , losing 2 $H_20$ to m/z 315.1970. See their reports under that mass. (p12).

# Unknown Prostanoids (2,78, 2.96, 3.16 min)

# $DXA_3$-like (3.64 min), $DXA_3$ (3.85 min).

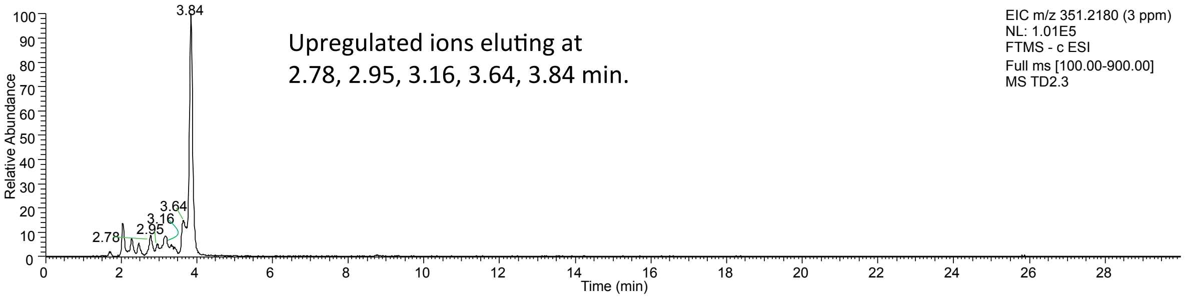


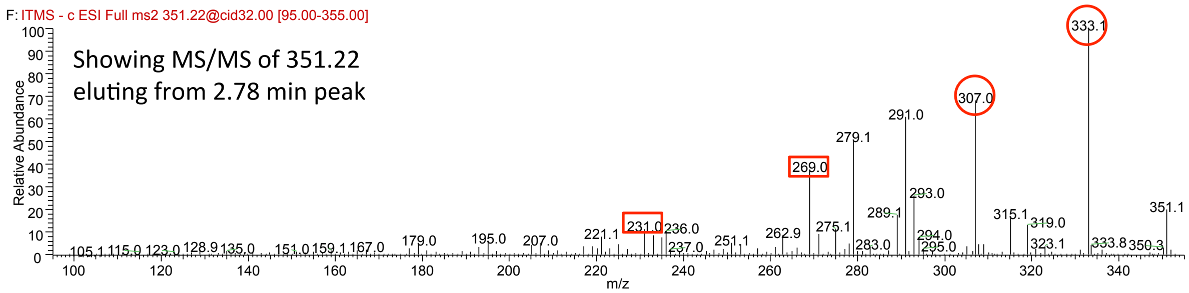


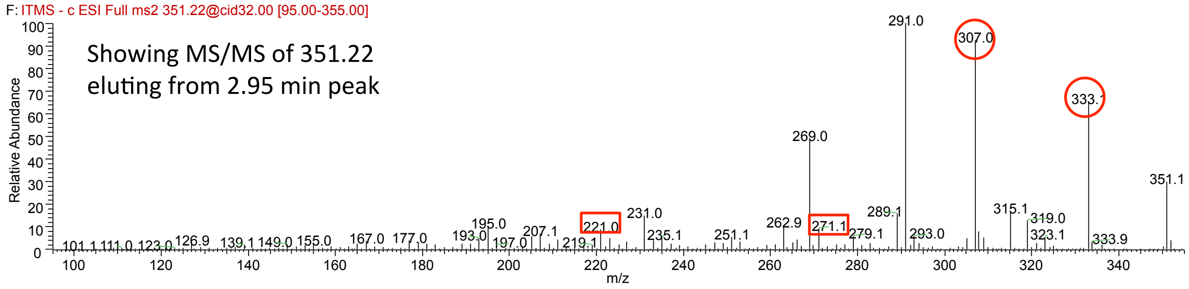


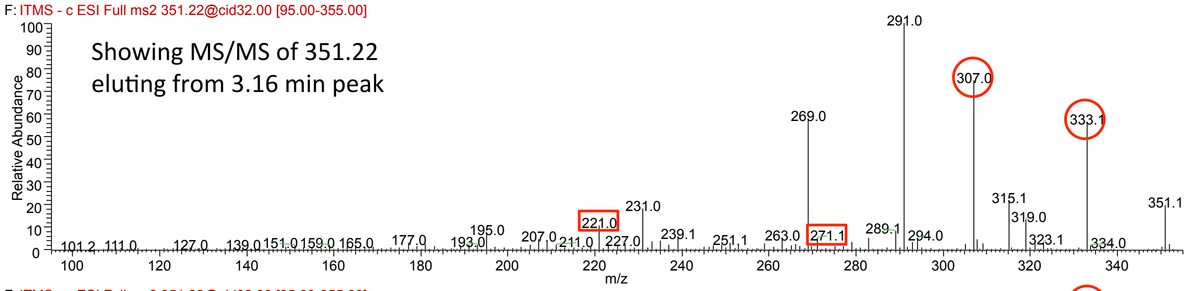


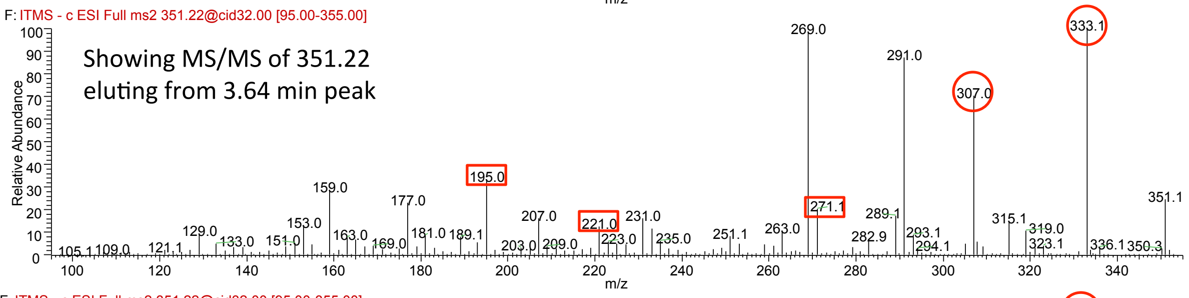


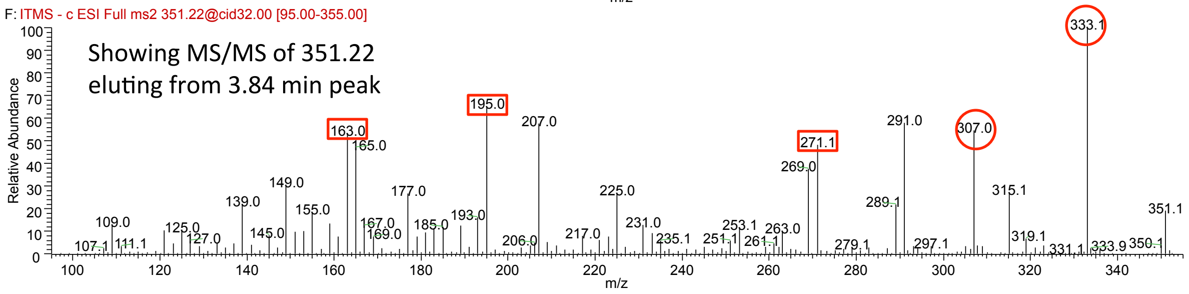

# Prostaglandin $F_2$-like (2.68, 2.84, 3.20 min) m/z 353.2335 $C_{20}H_{33}O_5$
# Unknown (3.42 min)

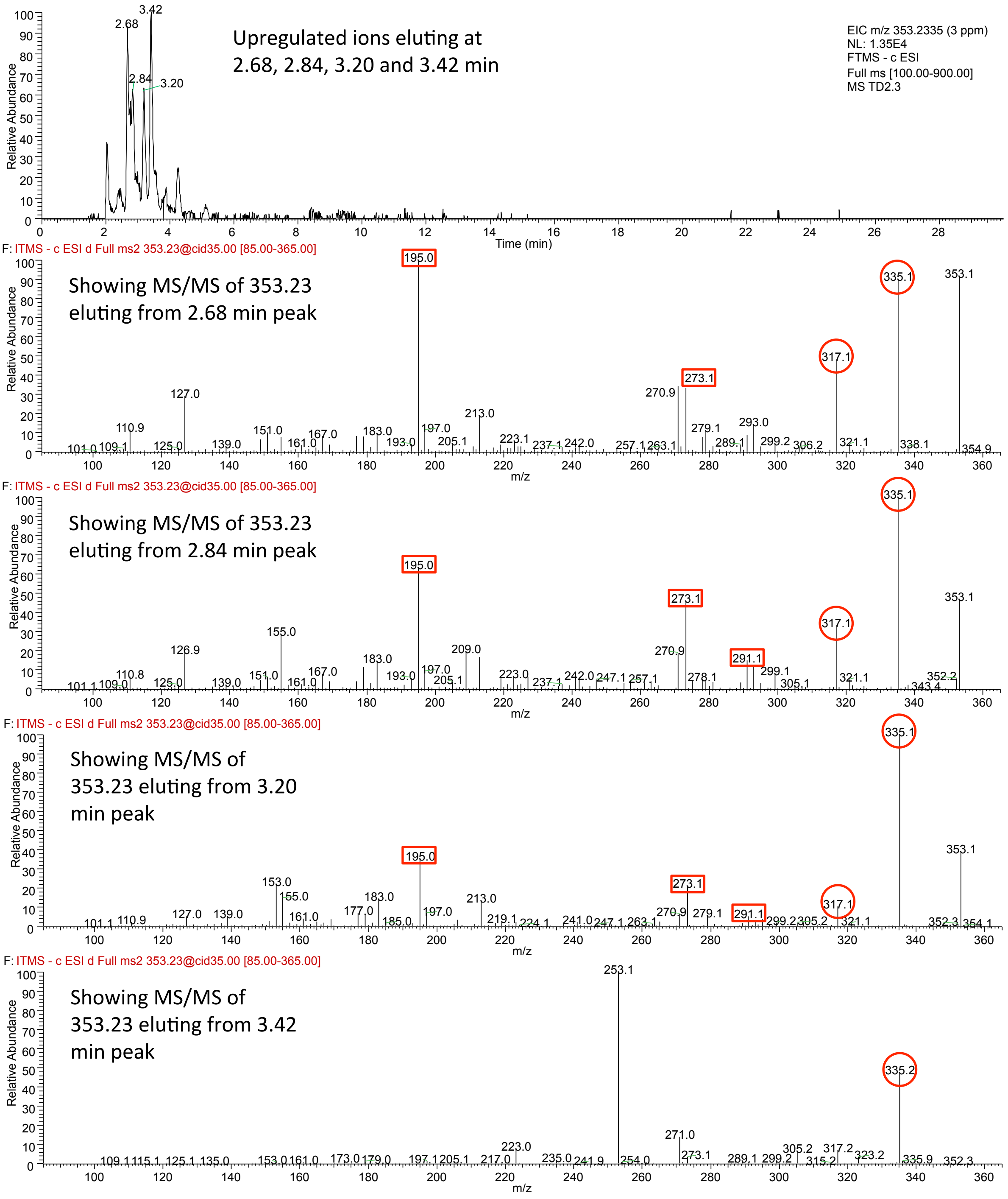

Thromboxane $B_2$ m/z 369.2285 $C_{20}H_{33}O_6$

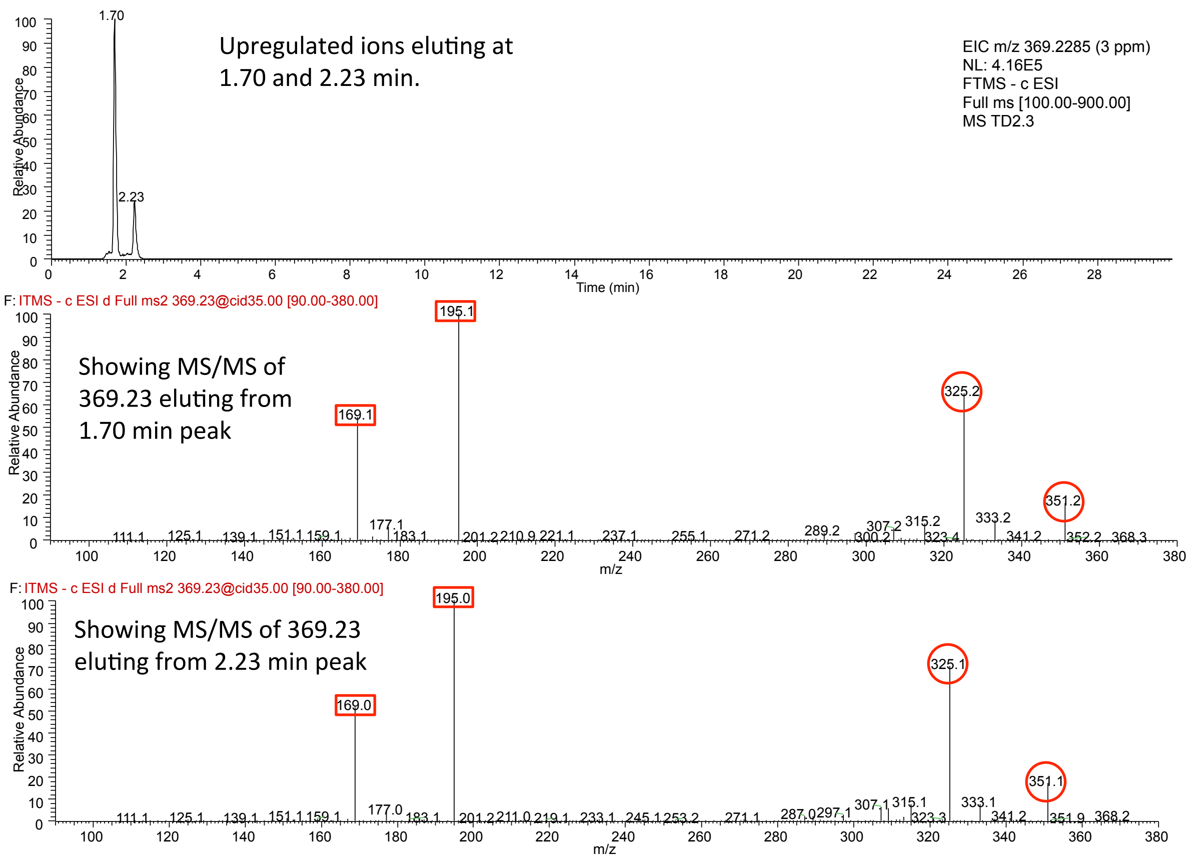


Note- $TXB_2$ undergoes keto-enol tautomerism and elutes as two peaks
