# Supplementary material for: Mapping the Human Platelet Lipidome Reveals Cytosolic Phospholipase A2 as a Regulator of Mitochondrial Bioenergetics during Activation
Source: Cell Metab. 2016 May 10;23(5):930–44. doi: 10.1016/j.cmet.2016.04.001 (PMC4873619; doi:10.1016/j.cmet.2016.04.001)

Docosanoic acids.

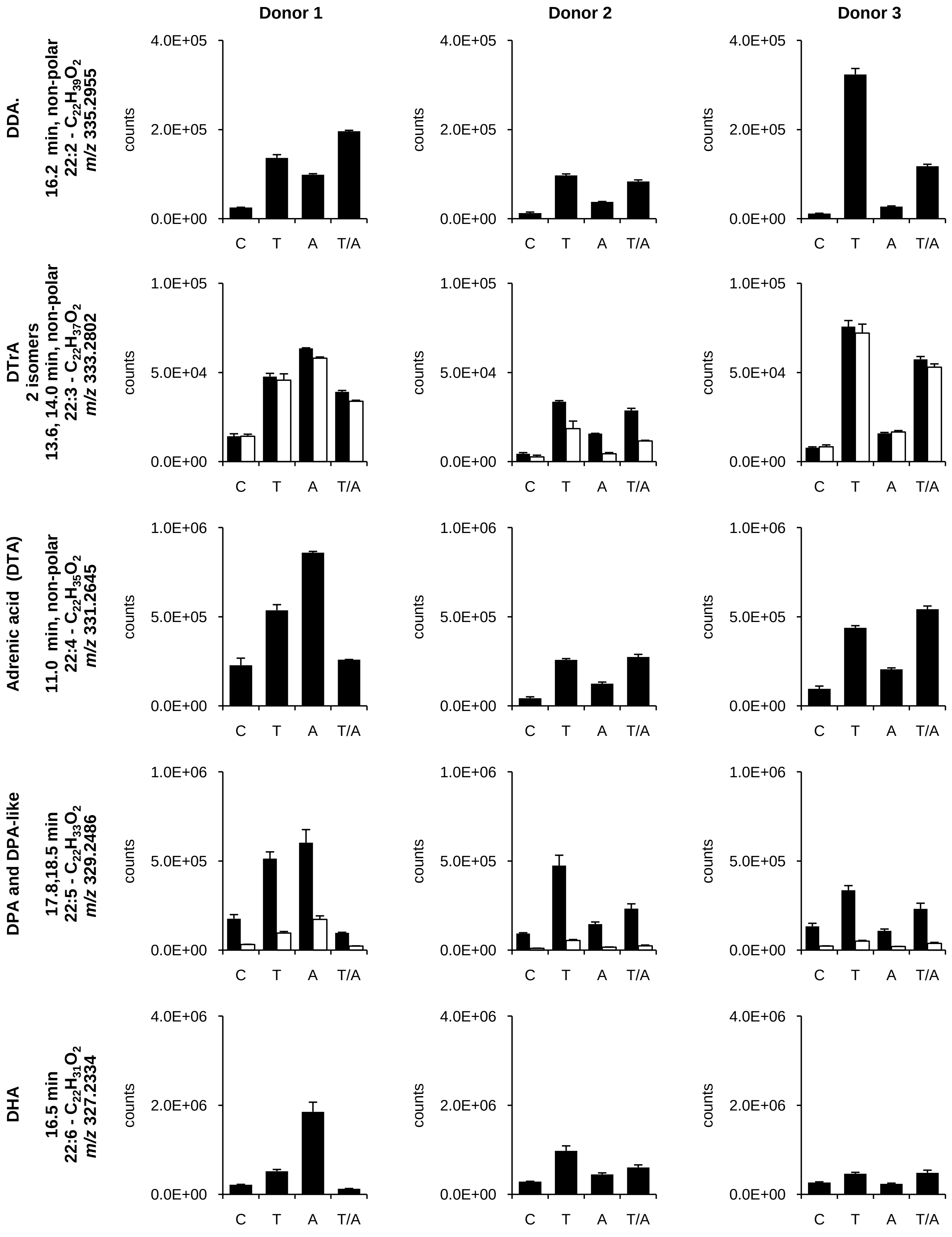

Eicosanoic acids.

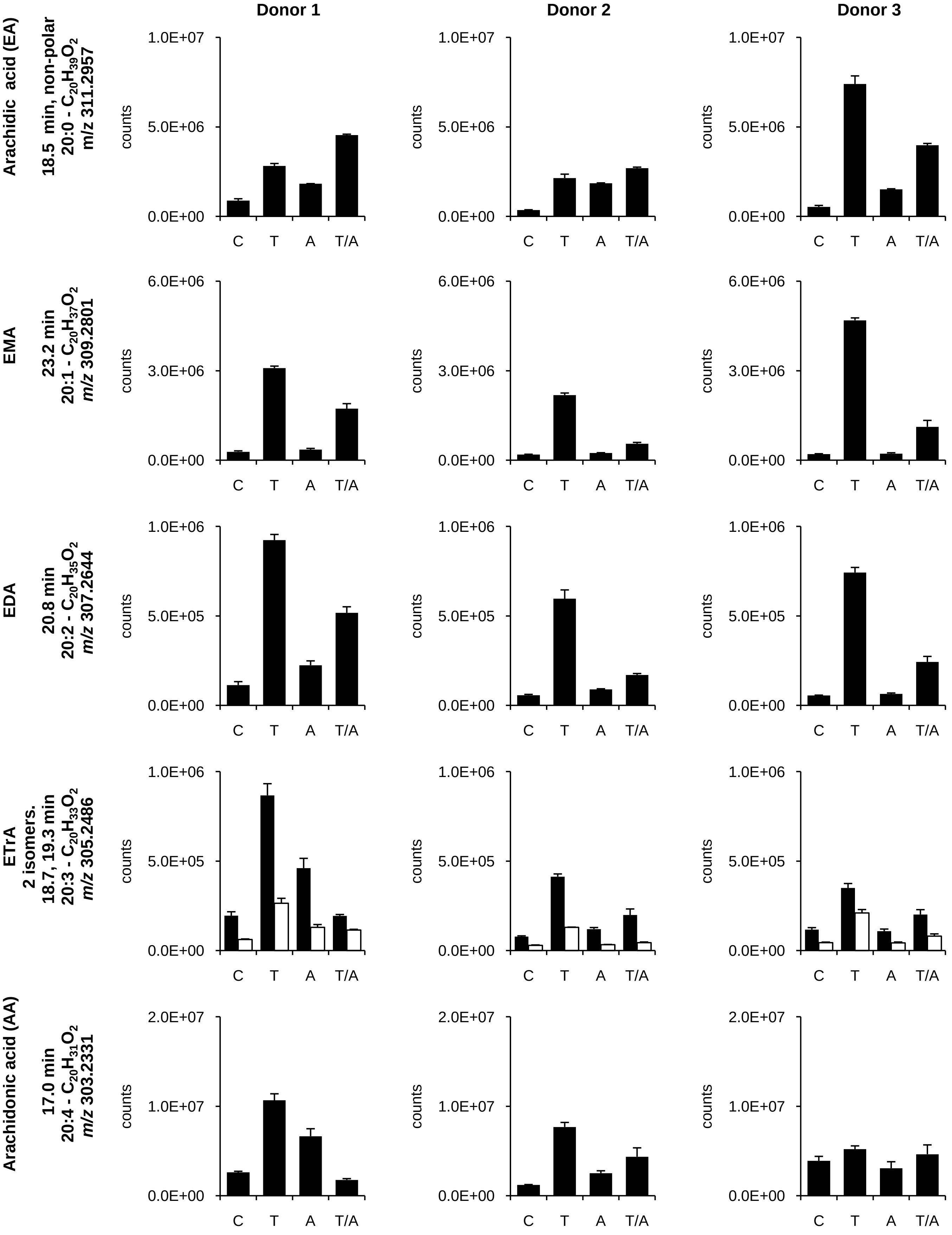

Eicosanoic acids (2).

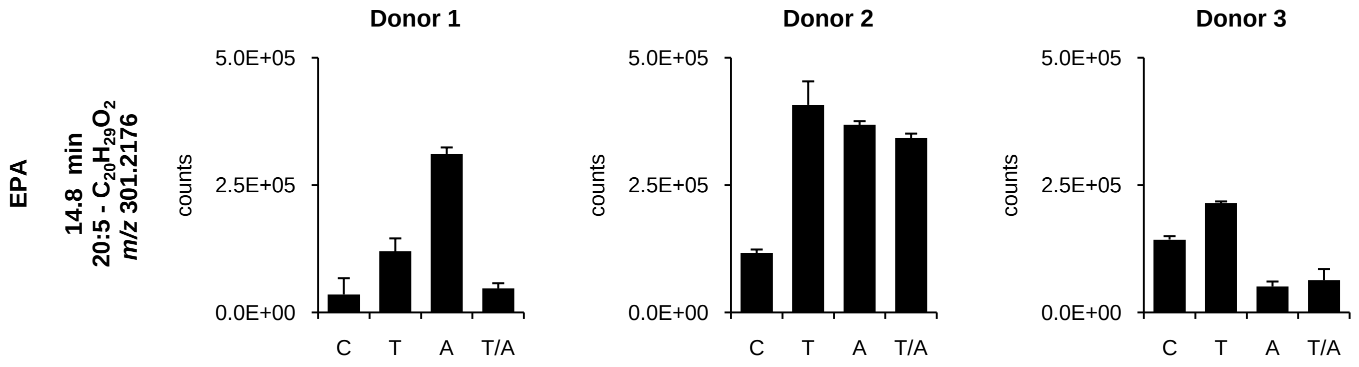

Nonadecanoic acids.

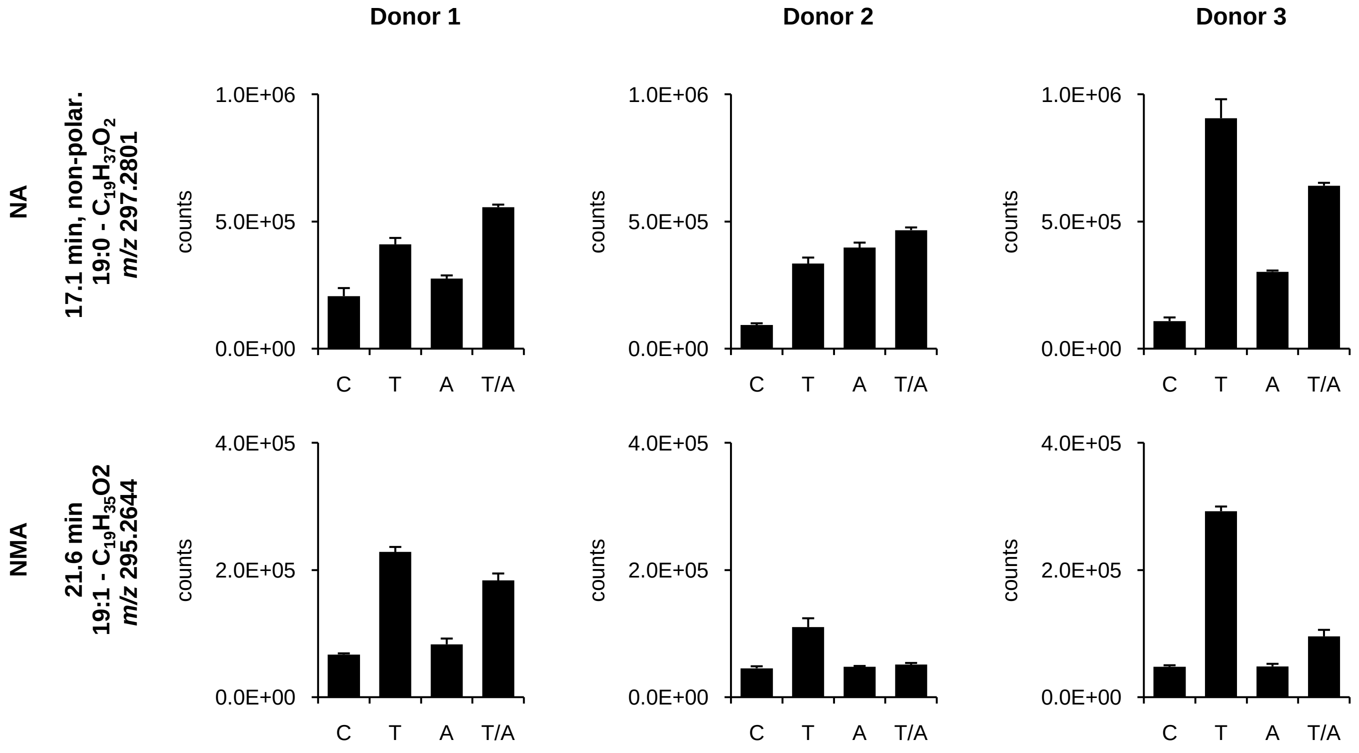

Octadecanoic acids.

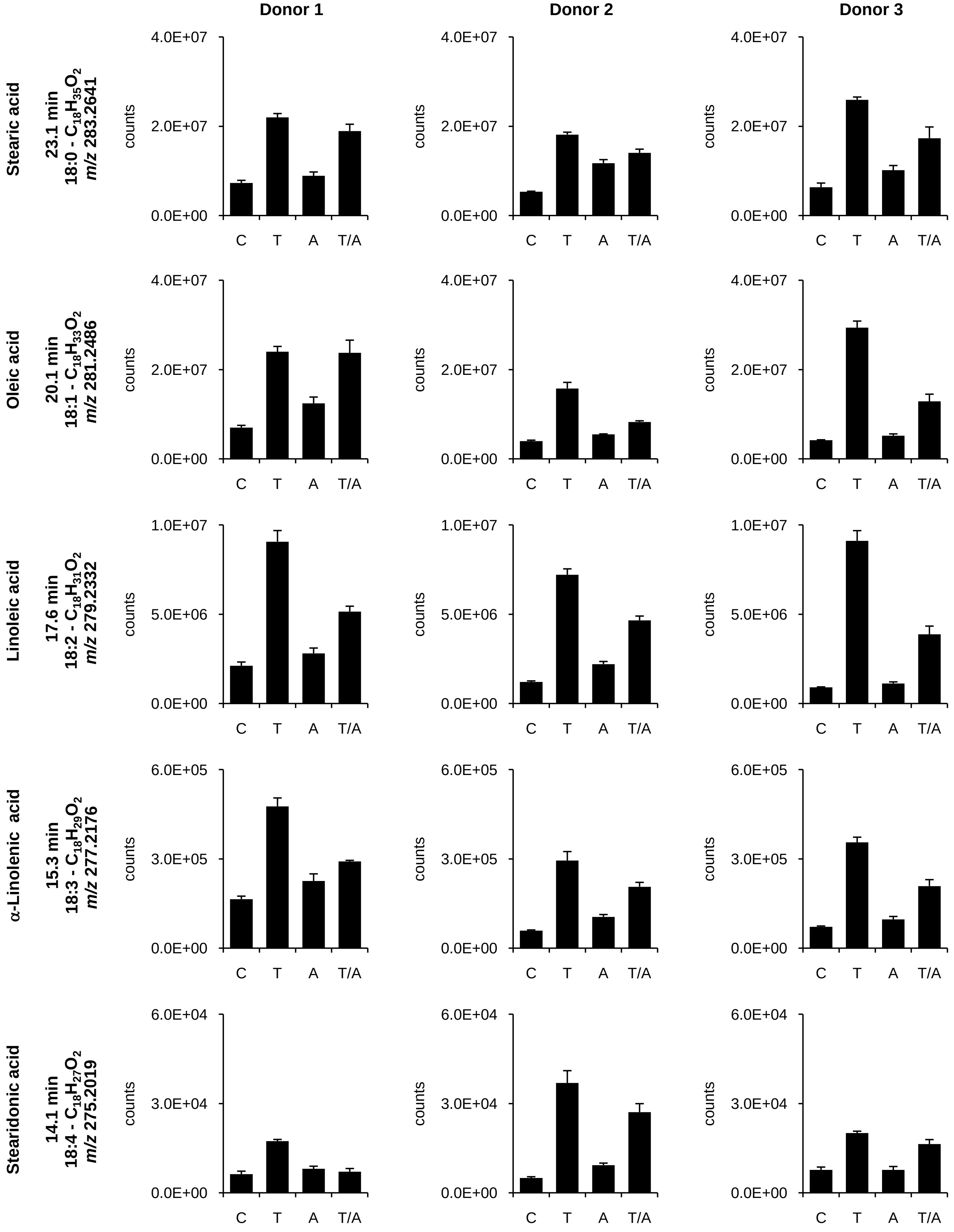

Hexadecanoic acids.

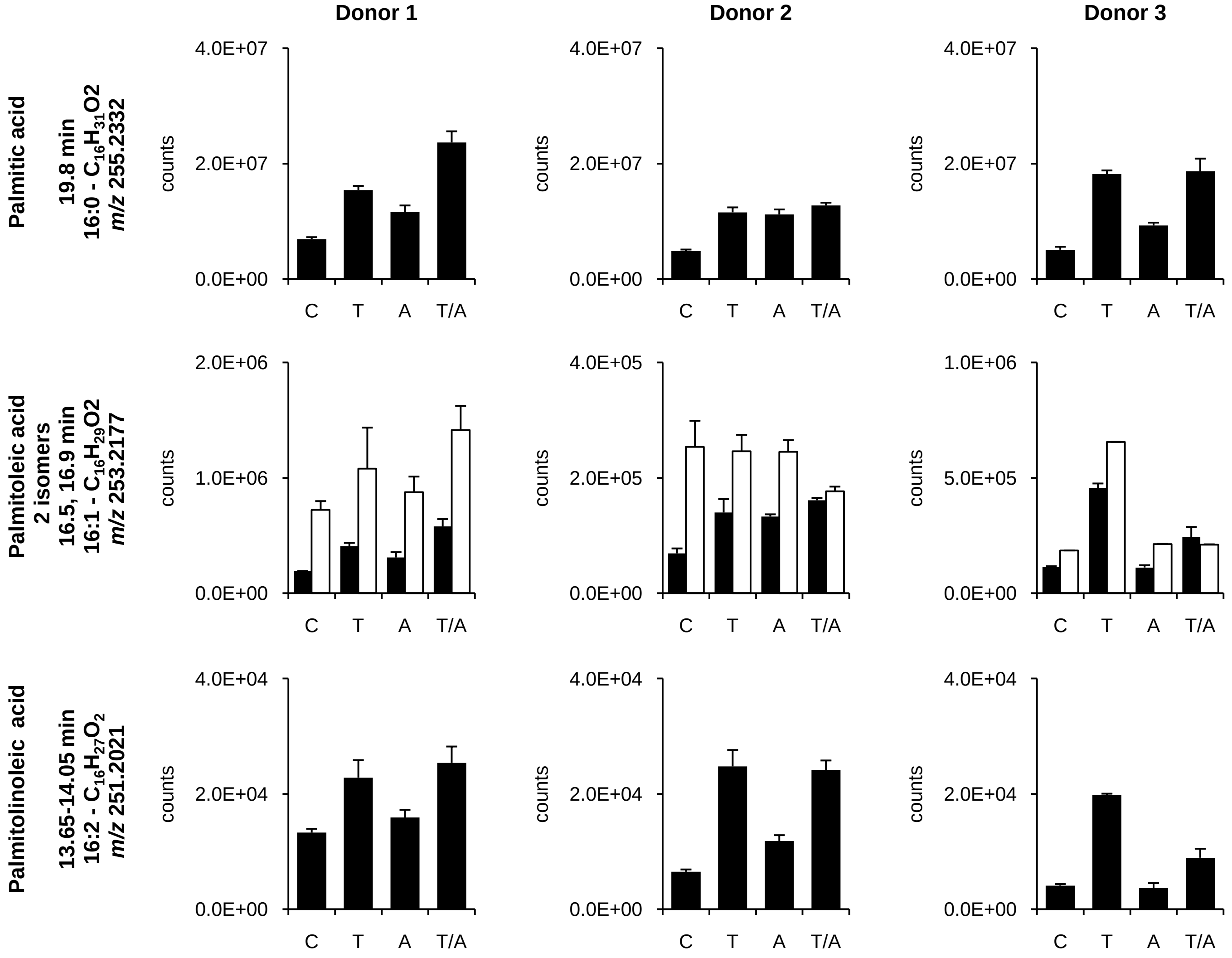

Hydroxydocosanoic acids.

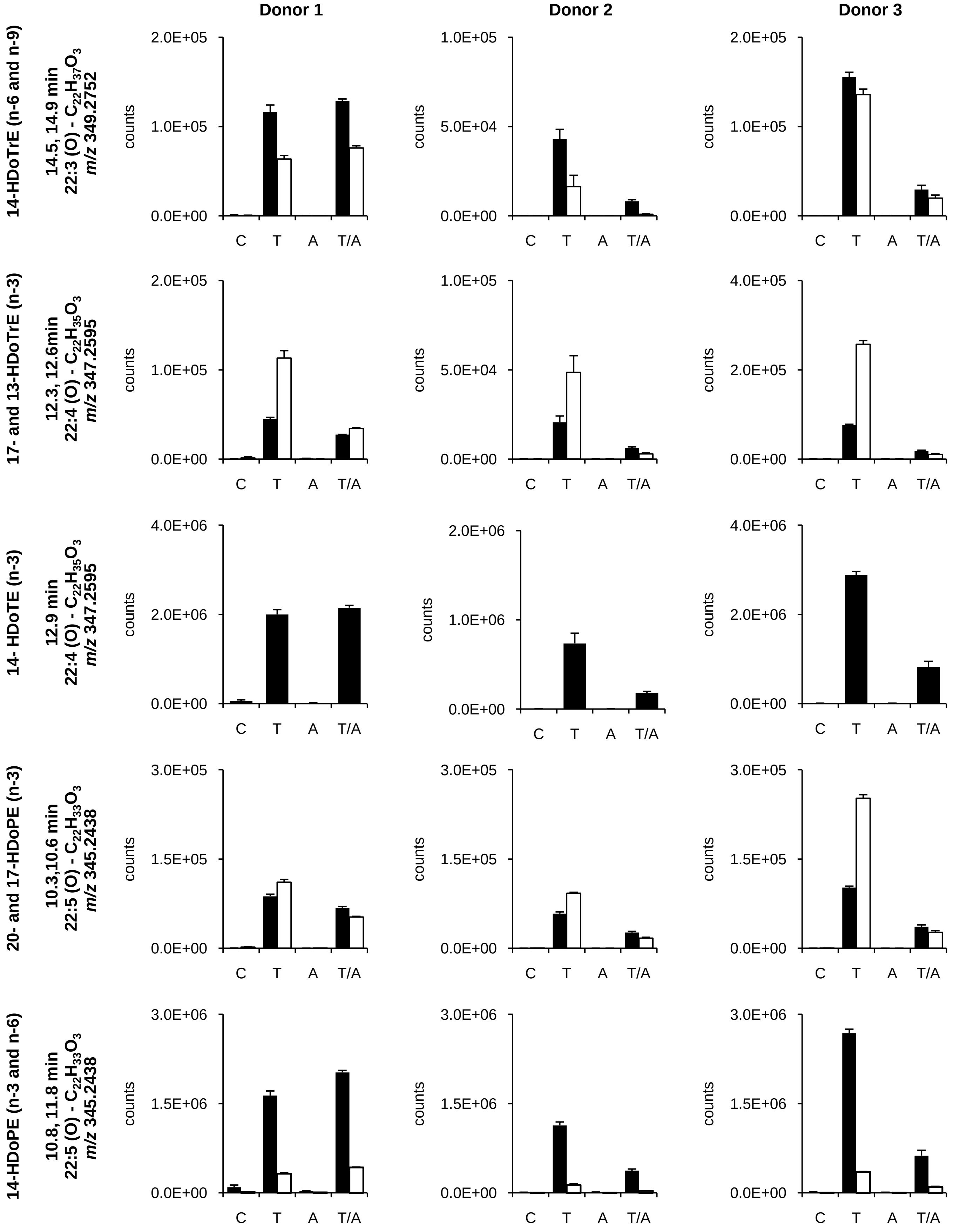

Hydroxydocosanoic acids (2).

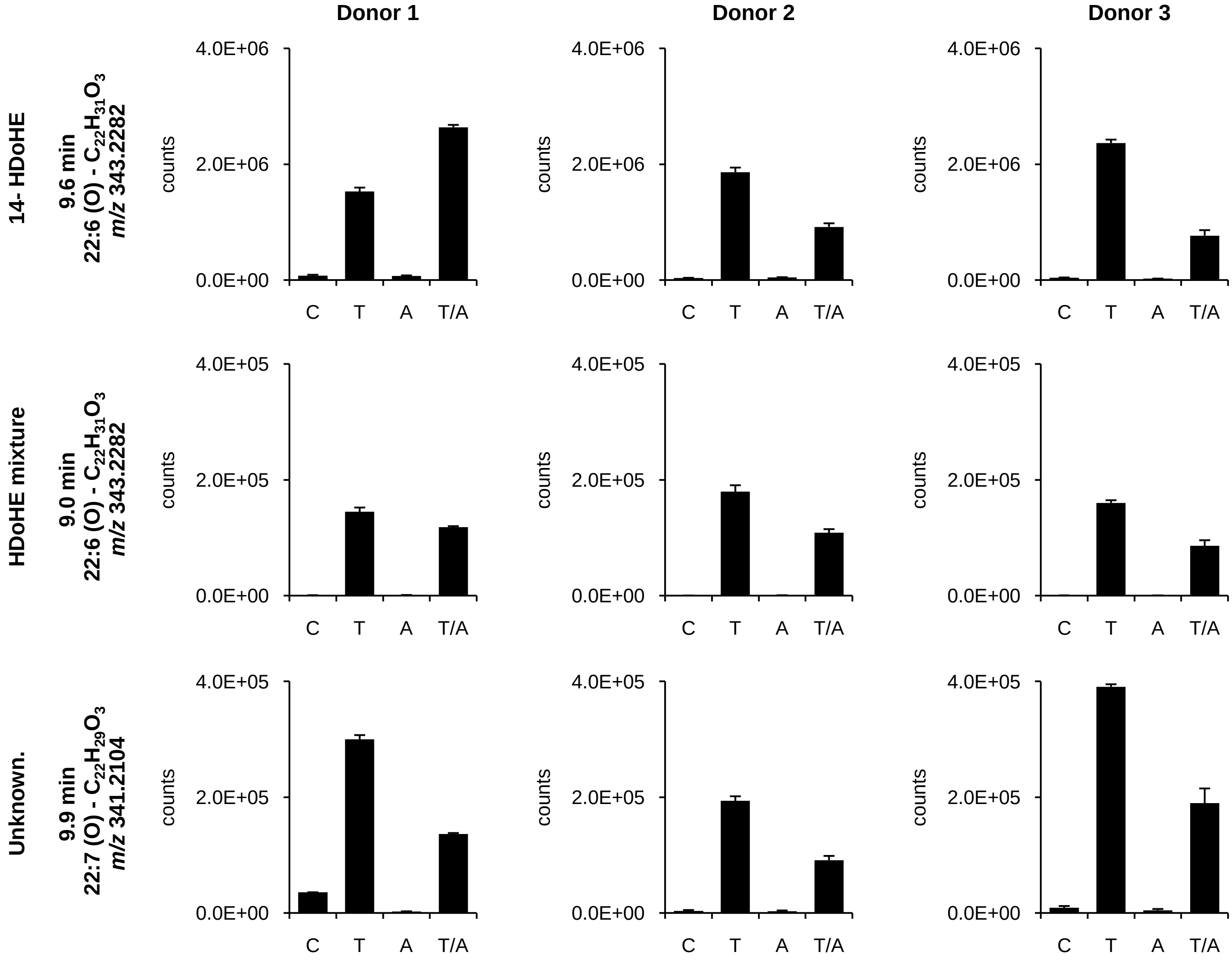

Hydroxyeicosanoic and prostaglandin A2-like acids.

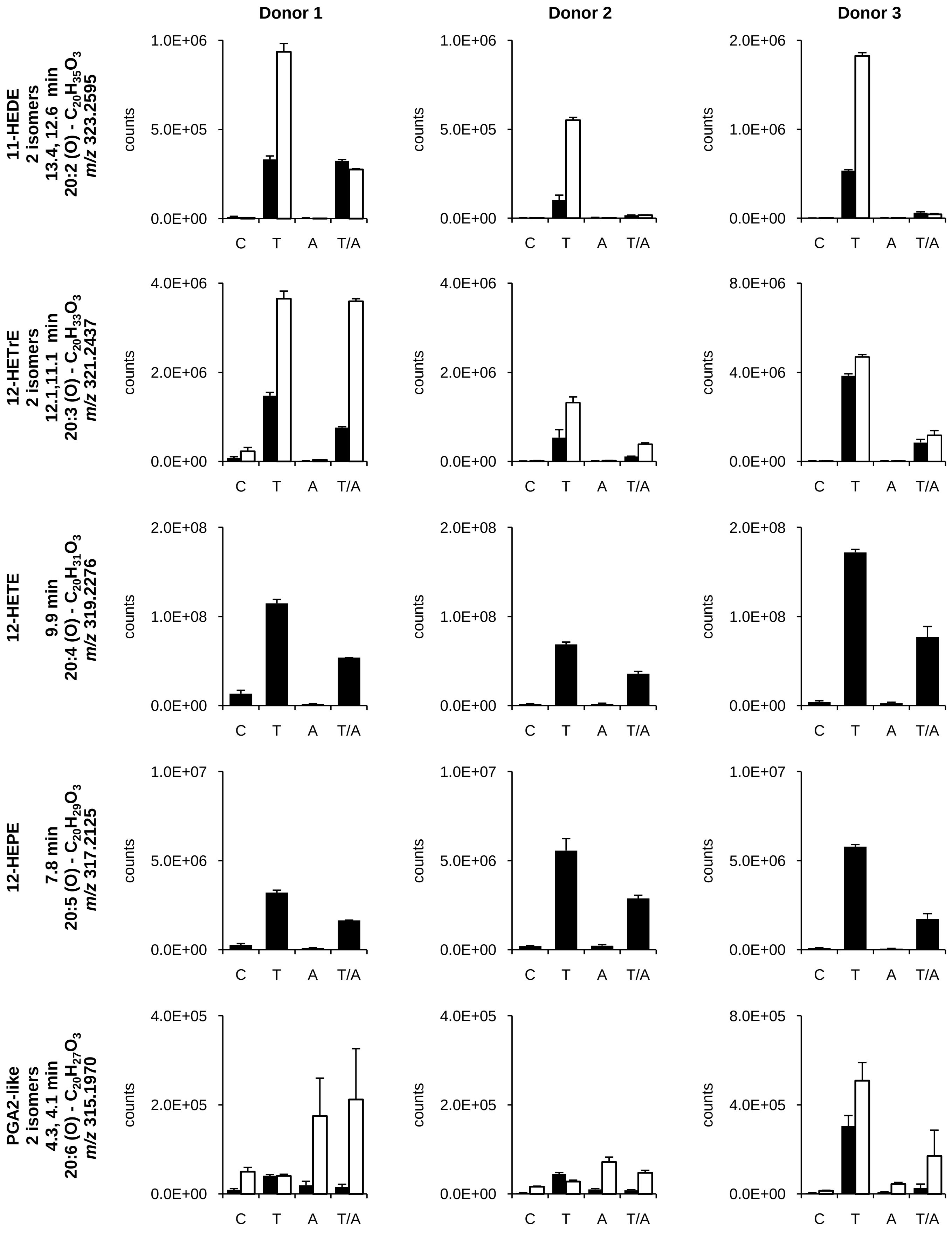

Hydroxynonadecanoic acids.

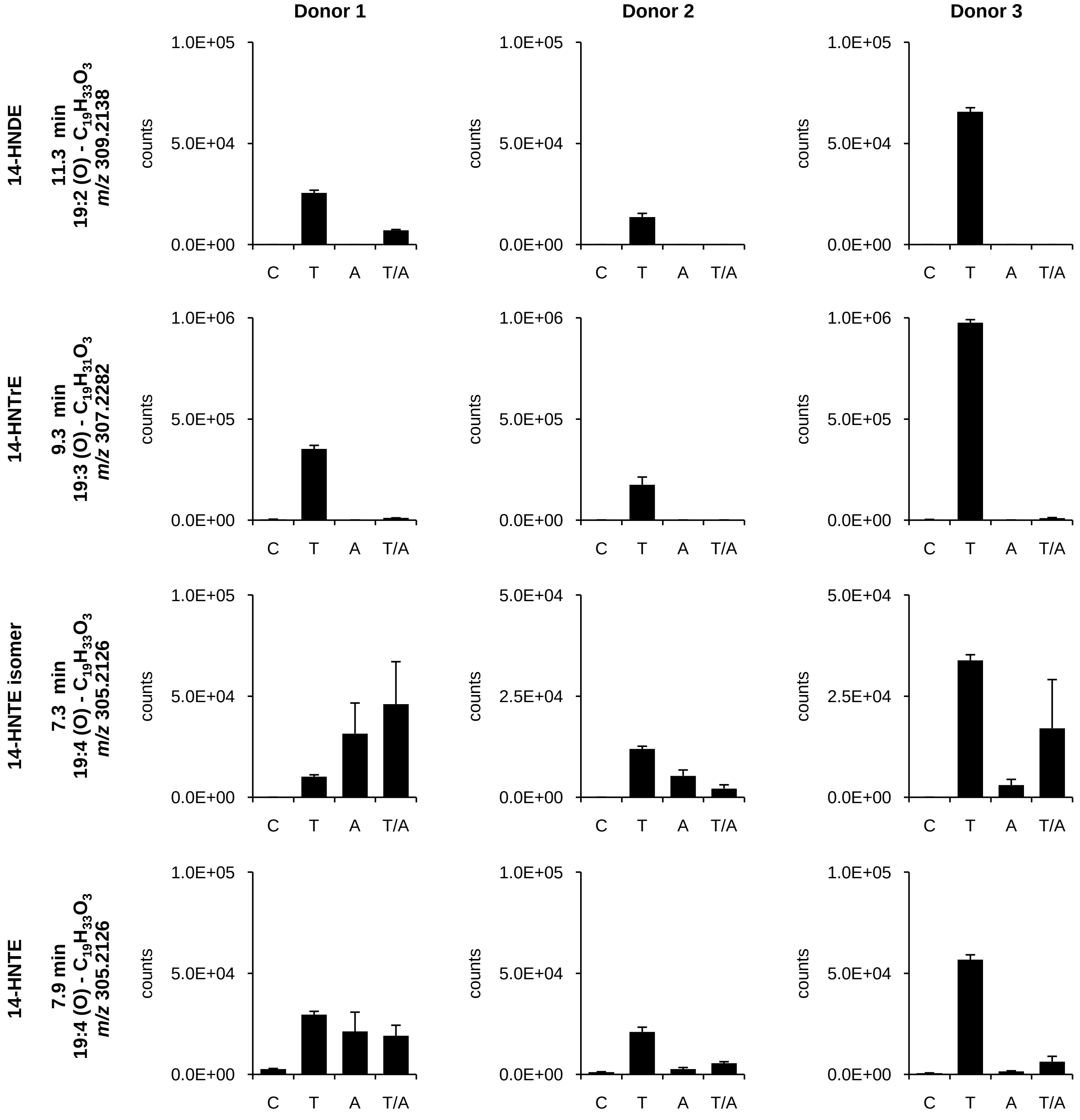

Hydroxyoctadecanoic acids.

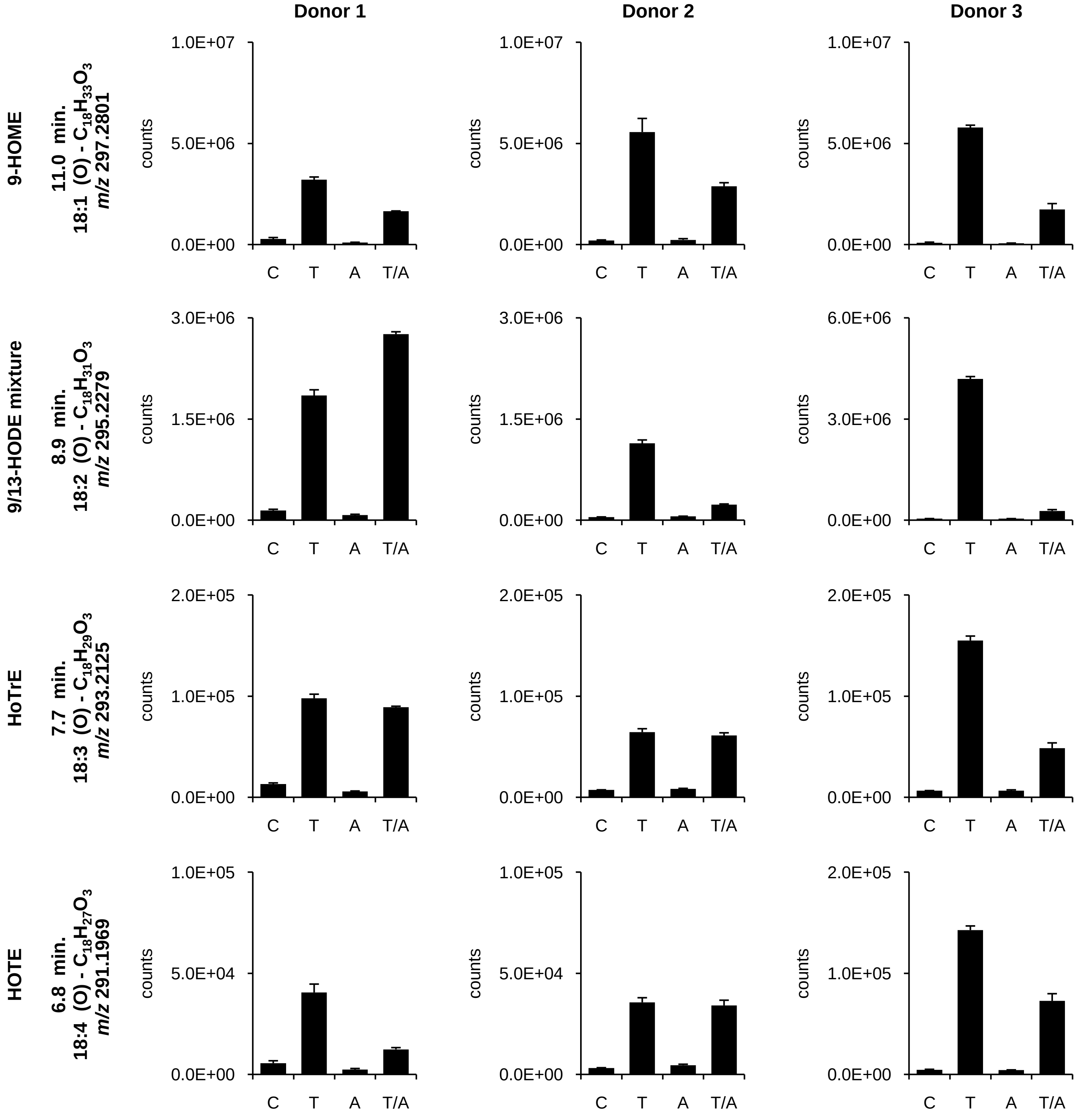

Hydroxyheptadecanoic acids.

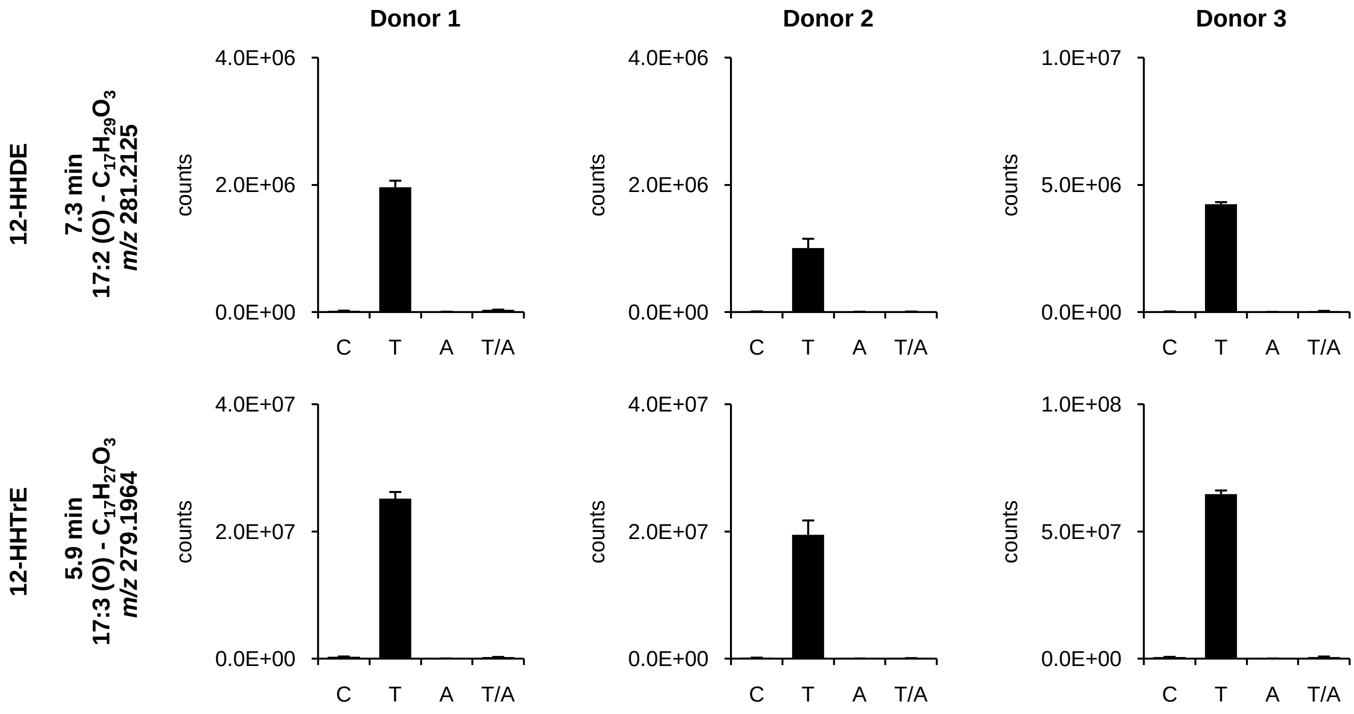

Hydroxytetradecadienoic acid mixture.

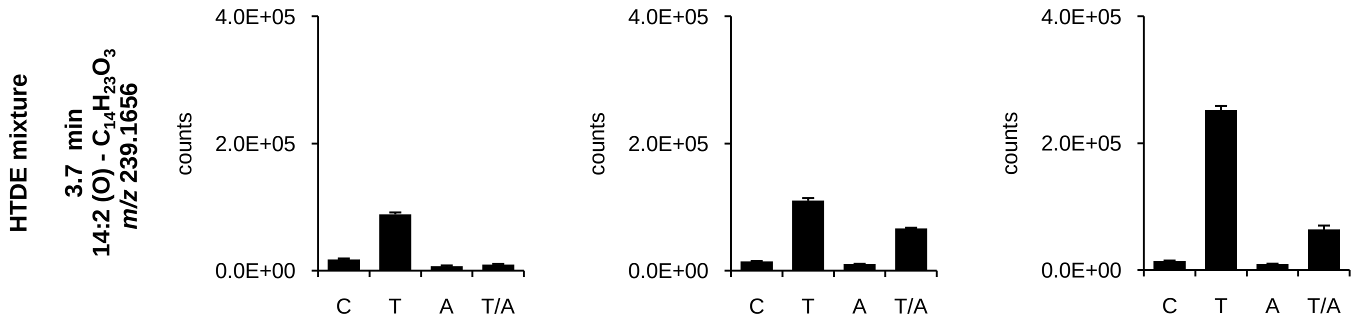

Dihydroxyeicosanoic acids and a Hepoxilin B3-like prostanoid.

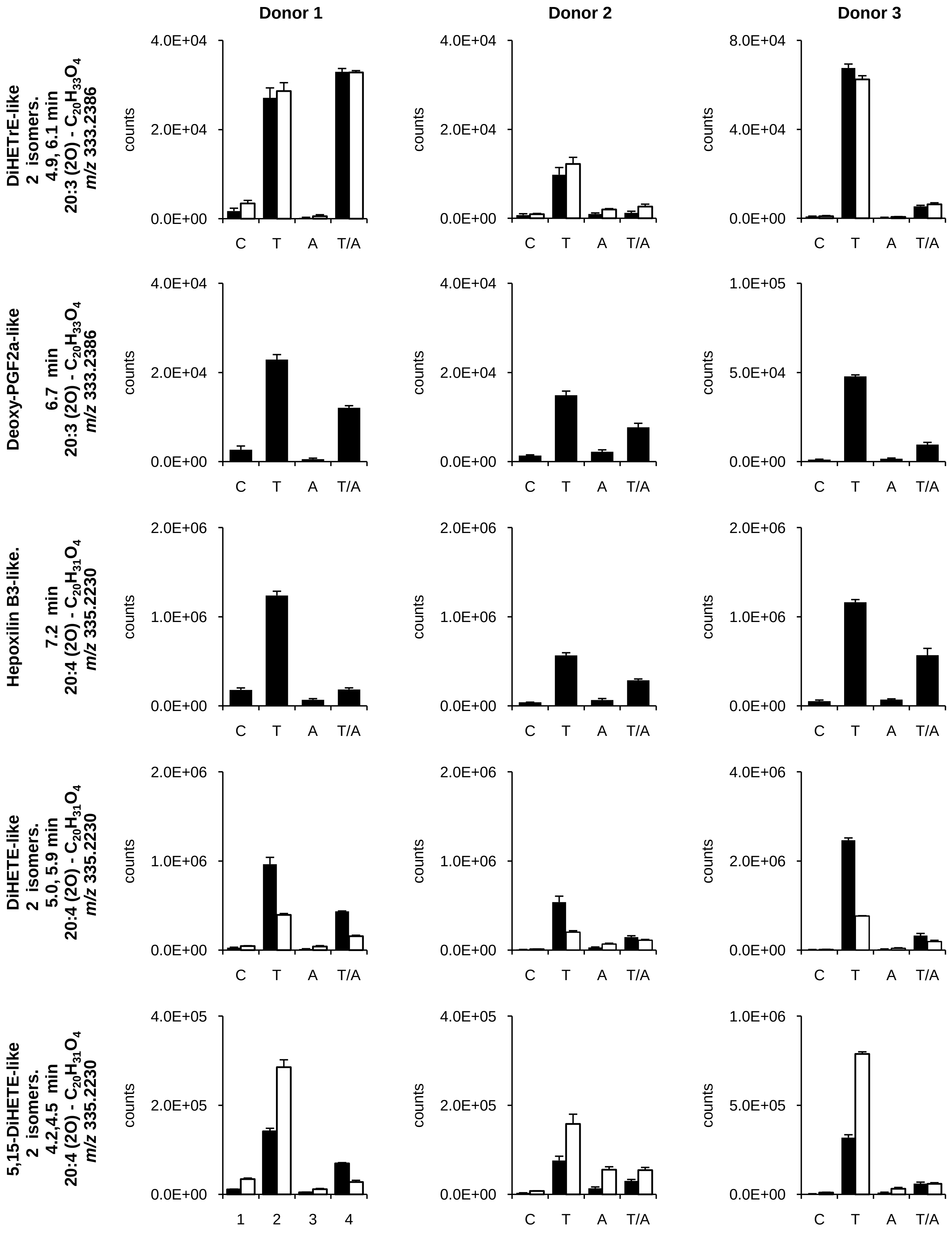

Prostaglandin B2-like and unknown acids.

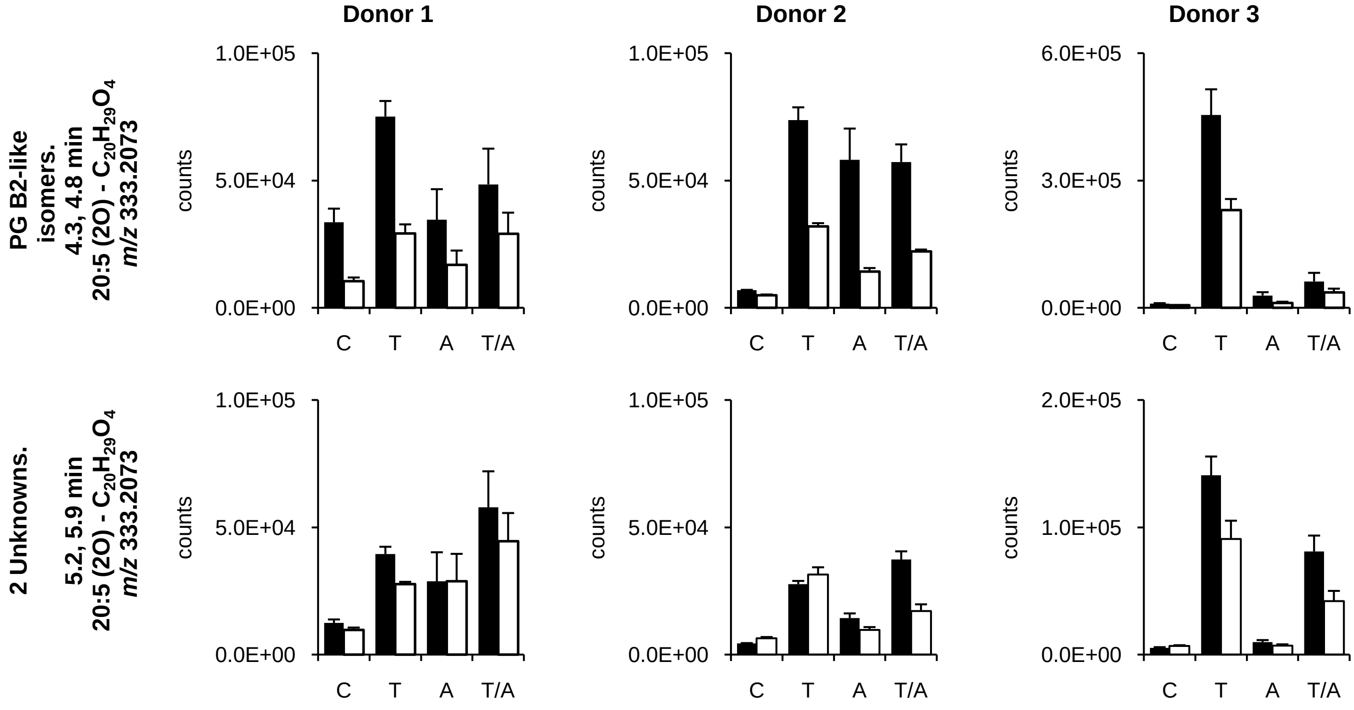

Dihydroxyoctadecatetraenoic acid -like

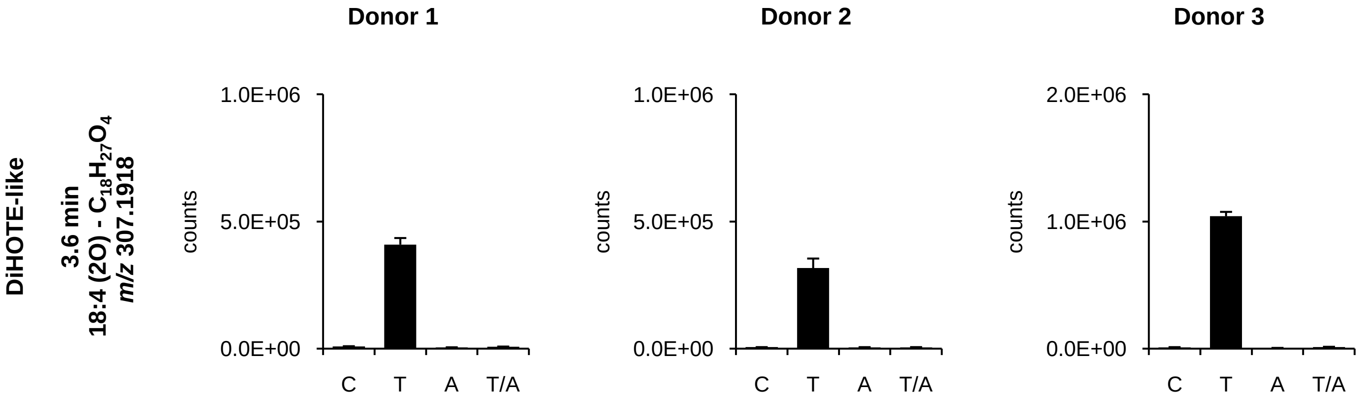

PGF2-like, DXA_3-like, and DXA_3 eicosanoids.

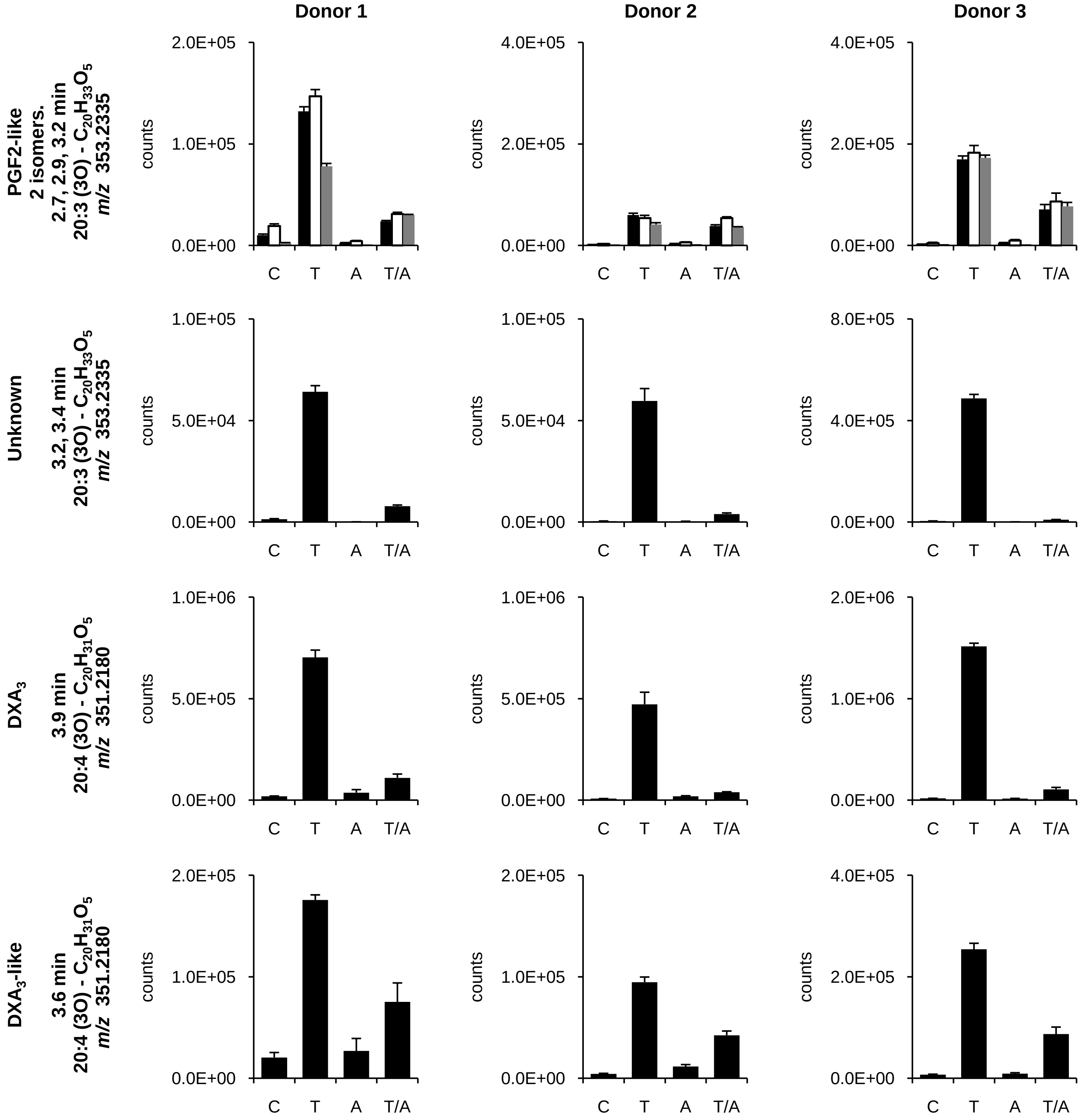

PGD_2, PGE_2, and 3 unknown prostanoids.

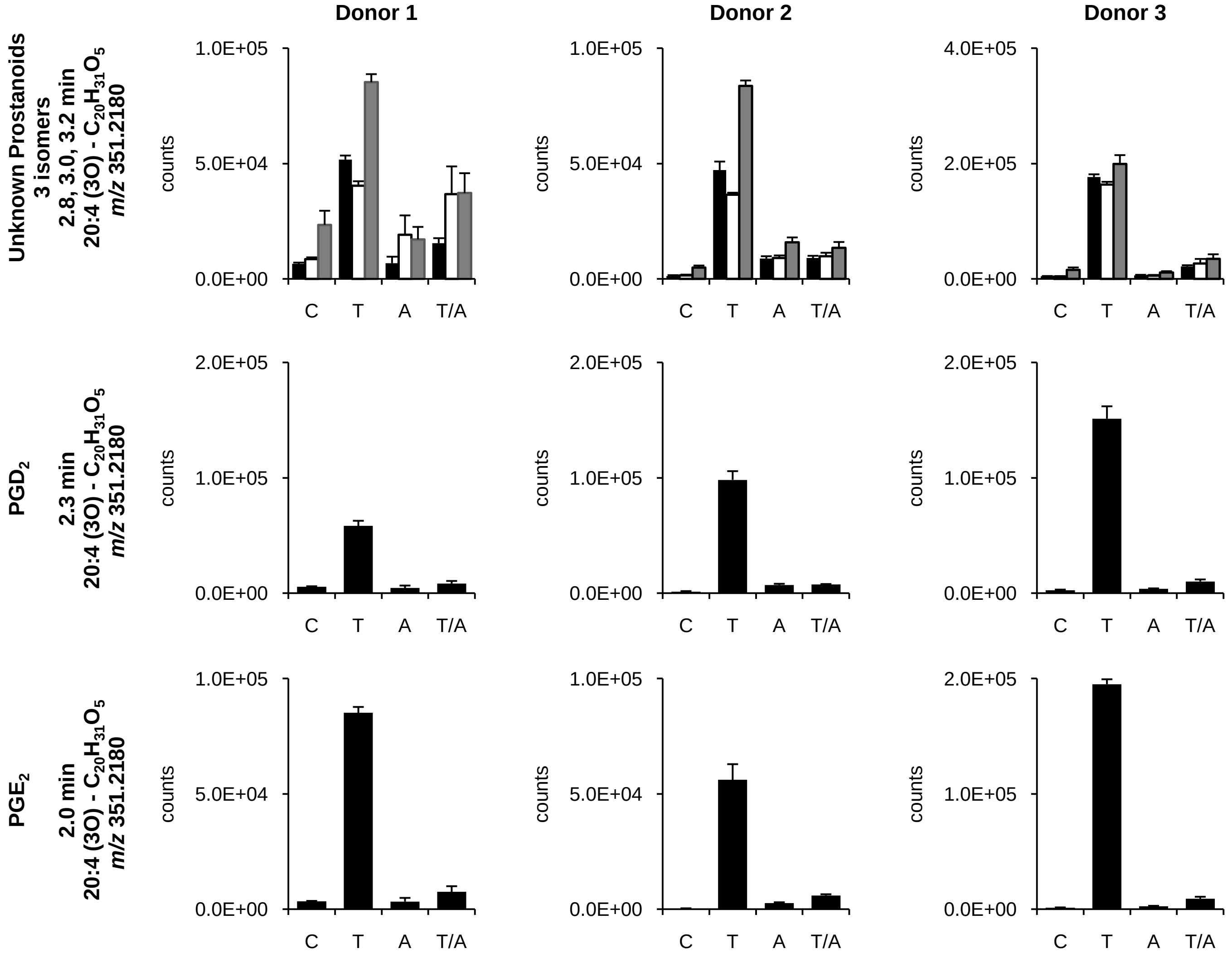

Thromboxane B_2.

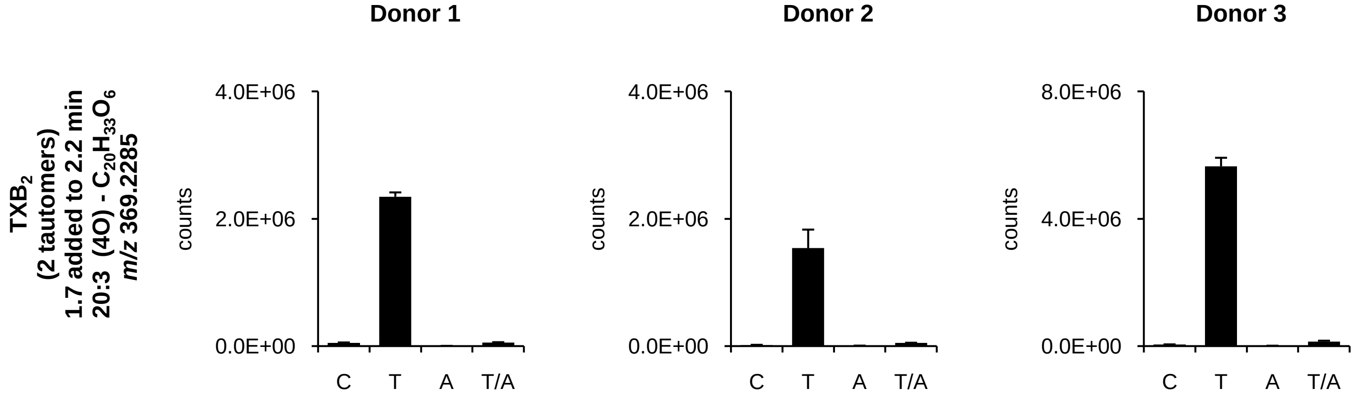

Supplement: Data S6. Response of All FA to Thrombin and Aspirin, Related to Figure 3D [file mmc7.pdf]
