# Supplementary material for: Mapping the Human Platelet Lipidome Reveals Cytosolic Phospholipase A2 as a Regulator of Mitochondrial Bioenergetics during Activation
Source: Cell Metab. 2016 May 10;23(5):930–44. doi: 10.1016/j.cmet.2016.04.001 (PMC4873619; doi:10.1016/j.cmet.2016.04.001)
Supplement: Document S2. Article plus Supplemental Information [file mmc8.pdf]

Resource

Cell Metabolism

Mapping the Human Platelet Lipidome Reveals Cytosolic Phospholipase A_2 as a Regulator of Mitochondrial Bioenergetics during Activation

Graphical Abstract

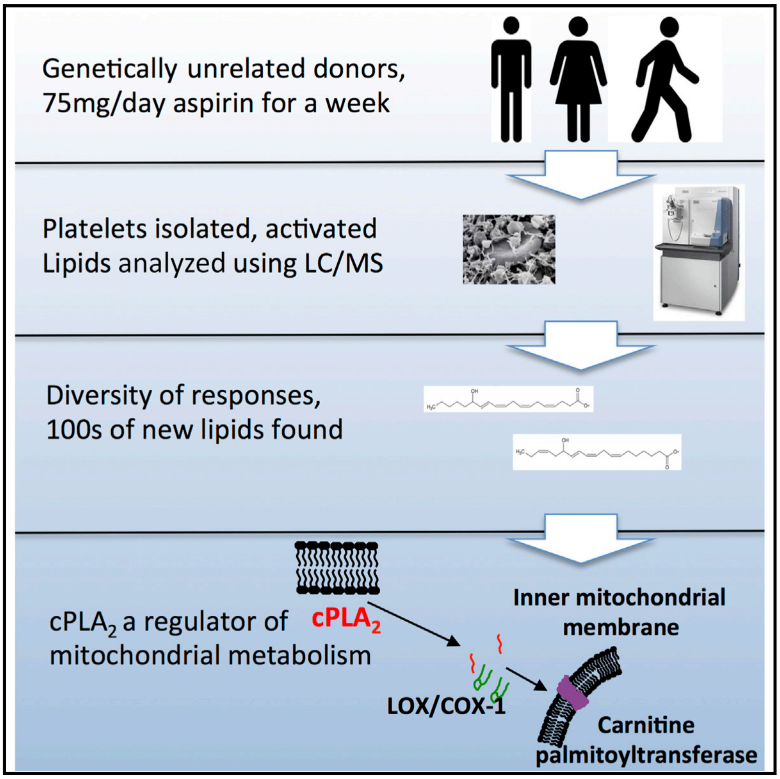

Authors

David A. Slatter, Maceler Aldrovandi, Anne O'Connor, ..., Saranya Ravi, Victor Darley-Usmar, Valerie B. O'Donnell

Correspondence

o-donnellvb@cardiff.ac.uk

In Brief

Slatter et al. characterize the lipidomic network of human platelets. Besides the characterization of nearly 200 oxidized species, the resource shows that remodeling of the membrane via phospholipase activity provides energy substrates for respiration. The findings demonstrate a direct link between innate immunity and mitochondrial bioenergetics in human platelets.

Highlights

- The complete lipidome of resting and agonist-activated human platelets is presented
- Low-dose aspirin supplementation profoundly affects the platelet lipidome
- Significant diversity in the lipidomes of three unrelated individuals is seen
- Cytosolic phospholipase A2 provides lipids for mitochondrial oxygen consumption

Slatter et al., 2016, Cell Metabolism 23, 930–944
May 10, 2016 © 2016 The Authors. Published by Elsevier Inc.
http://dx.doi.org/10.1016/j.cmet.2016.04.001

Cell Metabolism
Resource

Mapping the Human Platelet Lipidome Reveals Cytosolic Phospholipase A_2 as a Regulator of Mitochondrial Bioenergetics during Activation

David A. Slatter,[1] Maceler Aldrovandi,[1] Anne O'Connor,[1] Stuart M. Allen,[2] Christopher J. Brasher,[2] Robert C. Murphy,[3] Sven Mecklemann,[1] Saranya Ravi,[4] Victor Darley-Usmar,[4] and Valerie B. O'Donnell[1,*]
[1]Institute of Infection and Immunity and Systems Immunity Research Institute, School of Medicine, Cardiff University, Cardiff CF14 4XN, UK
[2]School of Computer Science and Informatics, Cardiff University, Cardiff CF14 4XN, UK
[3]Department of Pharmacology, University of Colorado Denver, Aurora, CO 80045, USA
[4]Department of Pathology, University of Alabama at Birmingham, Birmingham, AL 35294, USA
*Correspondence: o-donnellvb@cardiff.ac.uk
http://dx.doi.org/10.1016/j.cmet.2016.04.001

SUMMARY

Human platelets acutely increase mitochondrial energy generation following stimulation. Herein, a lipidomic circuit was uncovered whereby the substrates for this are exclusively provided by $cPLA_2$, including multiple fatty acids and oxidized species that support energy generation via β-oxidation. This indicates that acute lipid membrane remodeling is required to support energetic demands during platelet activation. Phospholipase activity is linked to energy metabolism, revealing $cPLA_2$ as a central regulator of both lipidomics and energy flux. Using a lipidomic approach (LipidArrays), we also estimated the total number of lipids in resting, thrombin-activated, and aspirinized platelets. Significant diversity between genetically unrelated individuals and a wealth of species was revealed. Resting platelets demonstrated ~5,600 unique species, with only ~50% being putatively identified. Thrombin elevated ~900 lipids >2-fold with 86% newly appearing and 45% inhibited by aspirin supplementation, indicating COX-1 is required for major activation-dependent lipidomic fluxes. Many lipids were structurally identified. With ~50% of the lipids being absent from databases, a major opportunity for mining lipids relevant to human health and disease is presented.

INTRODUCTION

Lipids play essential structural roles, act as nutrients, and control a broad range of physiological and pathophysiological events in cells. While several lipid families are well characterized at the molecular level, the total diversity and number of unique lipids in cells and how they change during cellular activation and differ in individuals are unknown. This hampers integration of lipidomics into systems biology, and addressing it will aid (1) fundamental understanding of lipid biology, (2) identification of drug targets for therapy, and (3) discovery of lipid biomarkers from disease cohorts. This is particularly relevant for identification of low-abundance bioactive lipids that are not detected by widely used high-throughput lipidomics.

Lipidomic methods usually target known families of lipids using mass spectrometry and do not "mine" the vast unknowns contained in mass spectrometry datasets. Previous estimates suggested mammalian cells contain 2,000–100,000, with a theoretical number of possible lipids at 100,000–500,000, but experimental attempts to robustly define this have not been undertaken (van Meer, 2005; Yetukuri et al., 2008). We developed a lipidomic workflow that maximizes coverage, ensuring that many low-abundance lipids were detected and verified (LipidArrays), a step essential for discovery of signaling lipids. This required multiple chromatographic separations, high-resolution mass spectrometry, and in-house generation of bioinformatic tools with both automatic and painstaking manual verification of data.

Platelets are central players in hemostasis, have emerging roles in cancer and immune regulation, and robustly activate lipid metabolism during thrombin-dependent aggregation. They are the therapeutic target of low-dose aspirin, via inhibition of cyclooxygenase-1 (COX-1)-dependent formation of thromboxane (TX). Aspirin at platelet-selective doses may prevent cancer metastasis in humans (Langley and Rothwell, 2013; Rothwell, 2013a, 2013b), through mechanisms that could involve lipid signaling. Last, COX-1-derived eicosanoids are mediators of pain, cancer metastasis, fever, vasoconstriction, and platelet activation. Thus, we sought to characterize the platelet COX-sensitive lipidome as a model system of relevance for human health and disease and to use this to uncover lipidomic control networks.

Lipids were mapped under basal and thrombin-activated conditions, with or without in vivo aspirin supplementation (75 mg/day for 7 days). The results indicate that the human platelet lipidome is complex and heterogeneous, with profound changes on activation and in vivo aspirin inhibition. In particular, 192 fatty acids (FAs) and oxidized phospholipids (oxPLs) that support coagulation were structurally identified, including many lipids. FA formation required cytosolic phospholipaseA_2 ($cPLA_2$), which then fed into oxidative phosphorylation via β-oxidation. The activation of $cPLA_2$ by thrombin was also revealed to be an energy-requiring process itself. Last, energy

This is an open access article under the CC BY license (http://creativecommons.org/licenses/by/4.0/).

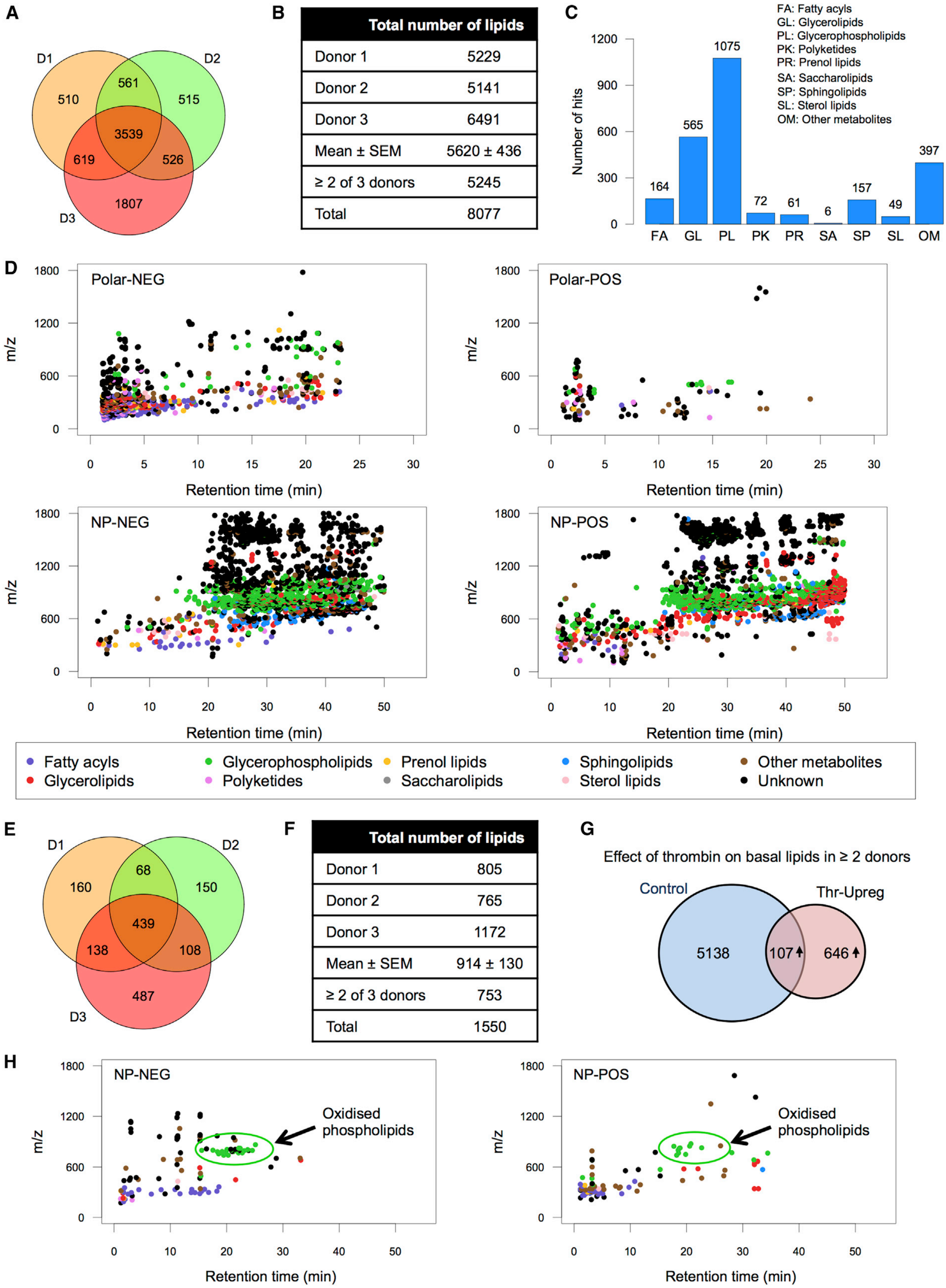

B

Total number of lipids	
Donor 1	5229
Donor 2	5141
Donor 3	6491
Mean ± SEM	5620 ± 436
≥ 2 of 3 donors	5245
Total	8077

F

Total number of lipids	
Donor 1	805
Donor 2	765
Donor 3	1172
Mean ± SEM	914 ± 130
≥ 2 of 3 donors	753
Total	1550

(legend on next page)

from FA oxidation was required for maintaining phospholipid asymmetry. These data link acute lipidomic flux with metabolic processes during innate immunity. Finally, the large numbers of unknowns represent a significant opportunity for lipid discovery.

RESULTS

Developing a Comprehensive Method to Analyze the Complete Platelet Lipidome

To determine the complete lipidome, high-resolution mass spectrometry was used to maximize detection of lipids with close *m/z* values, and extensive chromatography was used to separate isobaric species and enable detection of low-abundance lipids. New software was generated, and datasets were also subject to painstaking manual verification. The approach, including bioinformatic and technical aspects, is described in full in Supplemental Information.

Characterizing the Size and Diversity of the Platelet Lipidome in Three Genetically Unrelated Donors under Basal Conditions

Three genetically unrelated volunteers provided a baseline blood sample, and a second was provided following aspirin ingestion (75 mg/day, 7 days). First, we identified donor-specific total lipids at baseline. Platelets from all donors contained a total of 8,077 lipids, of which 5,245 (65%) were common in two or more donors. A single individual's platelets showed 5,620 ± 436 species (mean ± SEM, n = 3; Figures 1A and 1B). Full details of all ions detected in two or more donors are in Data S1. A degree of variation between individuals is noted, with each donor being ~84% the same as two or more donors, or 63.7% the same as all three (Figures 1A and 1B).

Results were queried against Human Metabolome Database (HMDB), LipidMaps, LipidHome, and METLIN, with tolerance of 5 ppm (Figures S1A and S1B) (Foster et al., 2013; Smith et al., 2005; Sud et al., 2007; Wishart et al., 2013). Many identified with multiple names, in a few cases with HMDB, as many as 200–300 (Figure S1B). Thus, to putatively assign an ion to a lipid family, we used the most common class for each. Most lipid classes were detected in platelets, with glycerolipids (GLs) and glycerophospholipids (PLs) predominating (Figure 1C; Data S1). METLIN does not assign molecules to a class, thus "other metabolites (OM)" refers to METLIN-identified ions of unknown class.

Scatter diagrams show the range of lipids contained in platelets (Figure 1D). Several lipids of unknown structure group together, typically at higher mass ranges and hydrophobicity. These and many other unknowns (total 51%) leave a wealth of lipids to be discovered and characterized. To allow detailed examination of the data, we created interactive scatter diagrams using GoogleVis that allow users to mine the unknowns, with the ability to zoom and identify based on *m/z* and retention time (Data S2). Crosschecking with Data S1 provides putative identifications for *m/z* values of interest. For example, note the large group of glycerophospholipids (PLs) in green in both non-polar negative and non-polar positive, and glycerides (GLs; including triglycerides) in red in the non-polar-positive dataset.

Characterizing the Platelet Lipidome on Thrombin Activation

Next, we examined global lipid changes on thrombin activation, analyzing lipids that upregulate ≥2-fold. Thrombin caused a selected subset of lipids to be generated, 1,550 in all three donors, with 753 lipids common to two or more donors, and mean of 914 per isolate (Figures 1E and 1F; Data S1). Of these, 86% were absent basally and only detected on thrombin activation (Figure 1 G). Within all upregulated lipids, 420 were increased by >10-fold. The most predominant upregulated lipids were putatively identified as FAs (including eicosanoids) and PLs (Figures S1C and S1D). Principal component analysis (PCA) and a heatmap show the diversity in thrombin responses between donors, with samples separated in both PC1 and PC2, before and after activation (Figures S1E and S1F). Furthermore, the heat map (showing lipids upregulated in two or more donors) demonstrates that each donor elevates groups of individual lipids to different extents (Figure S1E). Scatter diagrams show the pattern of thrombin upregulated lipids found in two or more donors, with individual signatures shown for reference (Figure S2). FAs (purple), including eicosanoids, are strongly represented in negative mode. As for basal lipids, there are similarities and differences between donors, in particular a prominent group of PLs (green) in both positive and negative mode at ~18–25 min in the non-polar datasets (*m/z* ~700–900), most of which are oxidized PLs, as shown later (Figure 1H, showing lipids present in two or more donors). The scatter patterns appear unique to each donor, analogous to a fingerprint (Figure S2). How they differ in larger groups of people, and with variables including age, gender, health, disease, and diet, will be the subject of

Figure 1. The Resting Human Platelet Lipidome and How It Changes upon Thrombin Activation

Platelet lipid extracts from three genetically unrelated donors (D1–D3) were analyzed by LC-FTMS on the Orbitrap Elite, at 60,000 resolution, then processed using SIEVE 2.0 followed by in-house-generated software as described in Supplemental Information.

(A) Venn diagram showing the number of ions in each donor lipid isolate. Common and donor specific ions are demonstrated.

(B) Table showing the number of ions in each donor and mean, total and lipids common to two or more donors. Calculated from (A).

(C) Predominant lipid classes in platelets. Ions were grouped according to the most predominant classification family based on classifications in databases.

(D) Scatter diagrams showing elution of lipids from polar or non-polar (NP) columns, in either negative- or positive-ion mode. Lipids are color-coded according to classification from databases. To view in interactive mode, download Data S2, which allows zooming and viewing of individual accurate *m/z* values for all ions. Putative identifications for all are in Data S1.

(E–H) The thrombin-stimulated lipidome. Platelet lipid extracts from three genetically unrelated donors (D1–D3), before and after activation for 30 min using 0.2 U/mL thrombin, were analyzed as above, showing ions that are elevated at least 2-fold. (E) Venn diagram thrombin-upregulated lipids in two or more donors. (F) Total number of lipids in each donor, shown individually (G) The effect of thrombin on basal platelet lipids. Lipids that are newly detected or elevate at least 2-fold in two or more donors are shown. Out of 5,245 lipids, only 107 were upregulated, with an additional 646 appearing only in the thrombin-activated samples. (H) Scatter diagrams showing elution of lipids from polar or NP columns, in either negative- or positive-ion mode. Putative identifications for all are in Data S1.

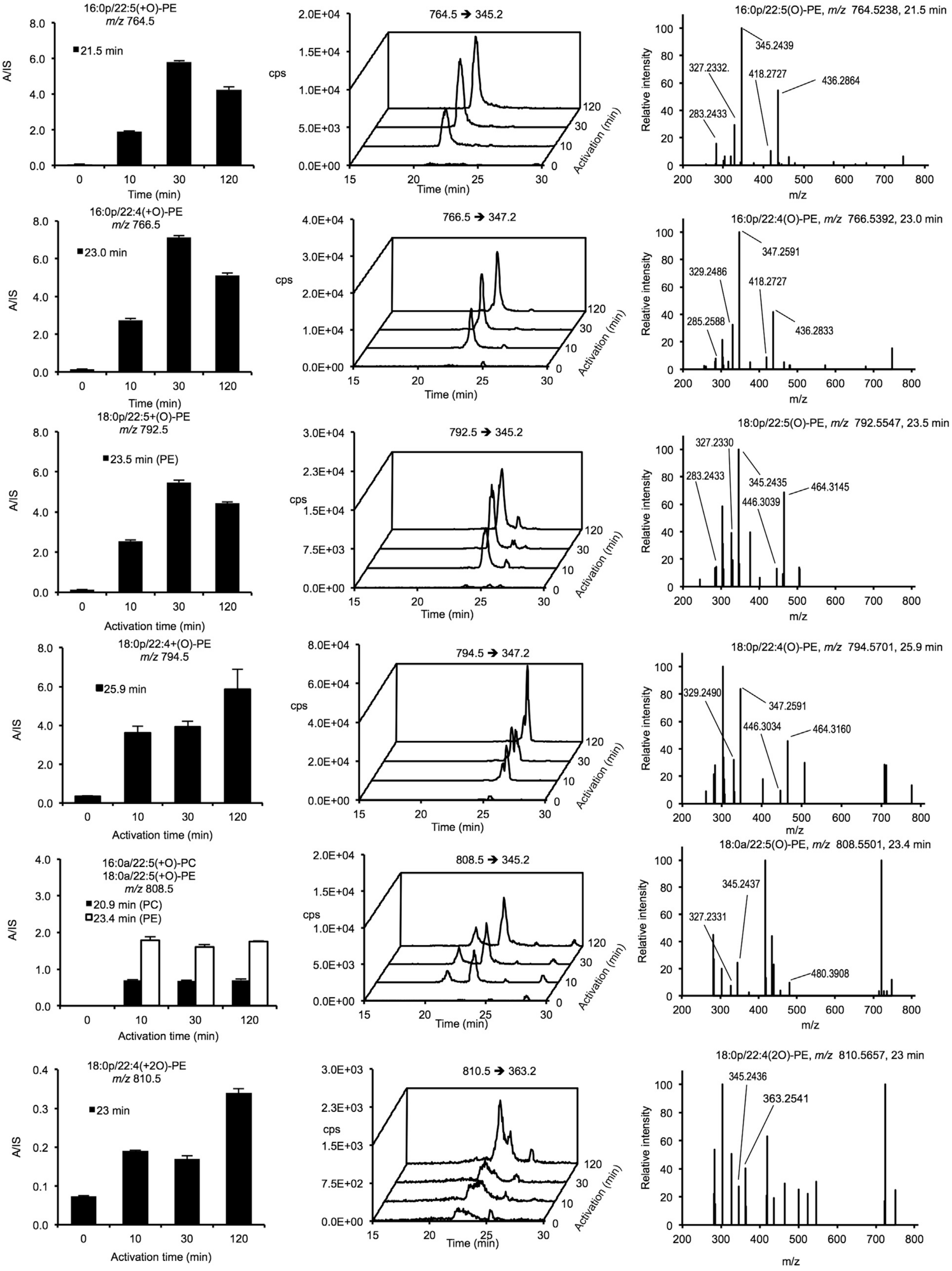

(legend on next page)

future study. Interactive GoogleVis scatter diagrams for these are in Data S3.

Full Structural Identification of a Large Group of OxPLs Generated Acutely by Thrombin-Activated Platelets

Our global analysis putatively identified several lipid families in platelets. Given the large number of ions, full structural validation of all is not feasible. Thus, we concentrated on mapping FAs and PLs that are elevated on thrombin activation. We previously found that platelets acutely generate oxPL via cyclooxygenase (COX) and lipoxygenase (LOX), giving a total of 18 molecular species (Aldrovandi et al., 2013; Morgan et al., 2010; O'Donnell and Murphy, 2012; O'Donnell et al., 2014; Thomas et al., 2010). Herein, a prominent group of ions was noted at 15–25 min on the nonpolar column (green in Figure 1H) and initially assigned as PLs via database searching. Further manual interrogation identified 111 oxPLs, indicating a large number of species (Table S2; Data S4). Tandem mass spectrometry (MS/MS) spectra were acquired and compared against LipidMaps and METLIN spectral databases, in tandem with manual interrogation. Assignments were based on the presence of diacyl fatty acid (*sn1*), oxidized fatty acid (*sn2*), neutral loss of ketene with and without water, and deprotonated precursor mass ion or loss of methyl ($[M\text{-}CH_3]^-$) for phosphatidylcholines (PCs) in negative ion mode, and many isobaric species are noted indicating positional and regioisomers. To further validate, we monitored the ions in a new donor platelet isolate, using molecular reaction monitoring (MRM) mode on a Q-Trap platform, monitoring parent *m/z* to *sn2* carboxylate anion as daughter ion (Table S3). As examples, time courses for generation of seven phosphatidylcholines (PC) and –ethanolamines (PE) containing 22:4 and 22:5 hydroxy lipids are shown, along with chromatograms and MS/MS spectra (Figure 2). All are virtually absent in resting platelets with immediate rises on activation and levels sustained for at least 2 hr. The top 52 upregulated oxPLs are displayed on a radar plot, which shows the relative abundance of specific families (Figure 3A). Twenty-three of the top 26 are monohydroxy lipids from arachidonic acid (AA), docosapentanoic acid (DPA), docosahexanoic acid (DHA), eicosapentanoic acid (EPA), and eicosatetraenoic acid (ETrA). Less abundant are multiple oxidized species and unsaturated fatty acids containing more than one oxygen group.

Identification of Fatty Acids and Oxidized Fatty Acids that Are Generated Acutely by Thrombin-Activated Platelets

A number of lipids putatively identified as FAs in Figure 1H, with additional ions eluting primarily on the polar-negative column, were subject to liquid chromatography (LC) MS/MS for identification. Of these, 81 were validated, by comparison with MS/MS spectra at LipidMaps or HMDB, or through manual curation (Table S4; Data S5). Many have never been detected before in human platelets, and include saturated and unsaturated fatty acids, known COX and LOX products, and mono/poly-oxygenated species of unknown origin. Several had daughter ions consistent with prostaglandins (PGs) or eicosanoids and include lipids with fragmentation similar to PGB_2 and PGF2α.

We highlight four, at *m/z* 309.1538, 307.2282, and 305.2126 (two lipids; Figures 3B and 3C). Their elemental composition indicated four C19-monohydroxy fatty acids, with two, three, or four double bonds, respectively. MS/MS spectra confirmed they were structurally related, showing +2 for several daughter ions, including *m/z* 205, 261, and 287 (Figure 3B; Data S5). Three showed neutral loss of 100 amu, indicating the position of the −OH at C14. The fourth showed loss of 98 due to the presence of a double bond distal to the −OH at C14. This indicates these to be 14-hydroxynonadecatetraenoic, -trienoic and -dienoic acids, respectively, for *m/z* 305, 307, and 309 (14-HNTE, 14-HNTrE, and 14-HNDE, n-6), with an n-3 form also found for 14-HNTE (Figure 3C). These are analogous to 12-hydroxy-5,8,10-heptadecatrienoic acid (12-HHTrE) generated by COX/thromboxane synthase oxidation of AA but instead form via oxidation of 22:5(n6), 22:4(n6), 22:3(n6), and 22:5(n3). We also highlight dioxolane A3 (DXA_3) identified via extensive gas chromatography/mass spectrometry (GC/MS), MS^3, and biological studies as a new lipid derived from COX-1. The complete data on this lipid will be presented elsewhere (unpublished data). MS/MS spectra for all 81 FAs are provided in Data S5.

Aspirin Blocks Selected Thrombin-Dependent Lipid Mobilization in Platelets, but Responses Are Highly Heterogeneous

To block platelet COX-1 in vivo, the same donors were administered 75 mg/day aspirin for 7 days, then platelets were isolated and activated using thrombin. Aspirin blocked TXB_2 and 12-HHT generation, demonstrating donor compliance (Data S6). Forty-five percent of thrombin-upregulated lipids were decreased by at least 50% by aspirin consumption. Aspirin also upregulated a small group of lipids (34 ions, 4.5%), while just over 50% were unchanged (Figure 3D).

Given the therapeutic importance of aspirin, we focused on suppressed ions. Forty-eight percent were absent from databases, while the majority were putatively identified primarily as FAs (Figure 3E). There were both similarities and variations in donor responses to aspirin. To illustrate this, we focused on the large group of fully validated FA elevated in thrombin-activated cells. Accurate mass data extracted from Orbitrap Fourier transform mass spectrometry (FTMS) at 60,000 resolution were manually extracted and integrated. Heatmaps show the diversity of responses with lipids color-coded depending on class (Figure 4). As examples of divergent responses, donor 1 shows elevations by aspirin alone of several unsaturated FAs (AA, DHA, EPA, adrenic, DPA, and DTrA isomers) that is greater than the thrombin response. In contrast, donor 2 shows low level increases in a far smaller number of largely different FAs, including some that are saturated (arachidic, palmitic, NA, palmitoleic, and

Figure 2. Time Course of Selected OxPL Generation by Human Platelets in Response to Thrombin
Washed platelets were activated using 0.2 U/min thrombin for varying times, then lipids were extracted and analyzed using a 4000 Q-Trap as described in Experimental Procedures, and with parent to daughter transitions as listed on the chromatograms. Left: time courses of integrated areas compared to internal standard (n = 3, mean ± SEM). Middle: sample chromatograms showing almost absence of lipids at 0 min followed by upregulation from 10 to 120 min. Right: MS/MS spectra for all lipids shown. More detailed MS/MS data are available in Data S4.

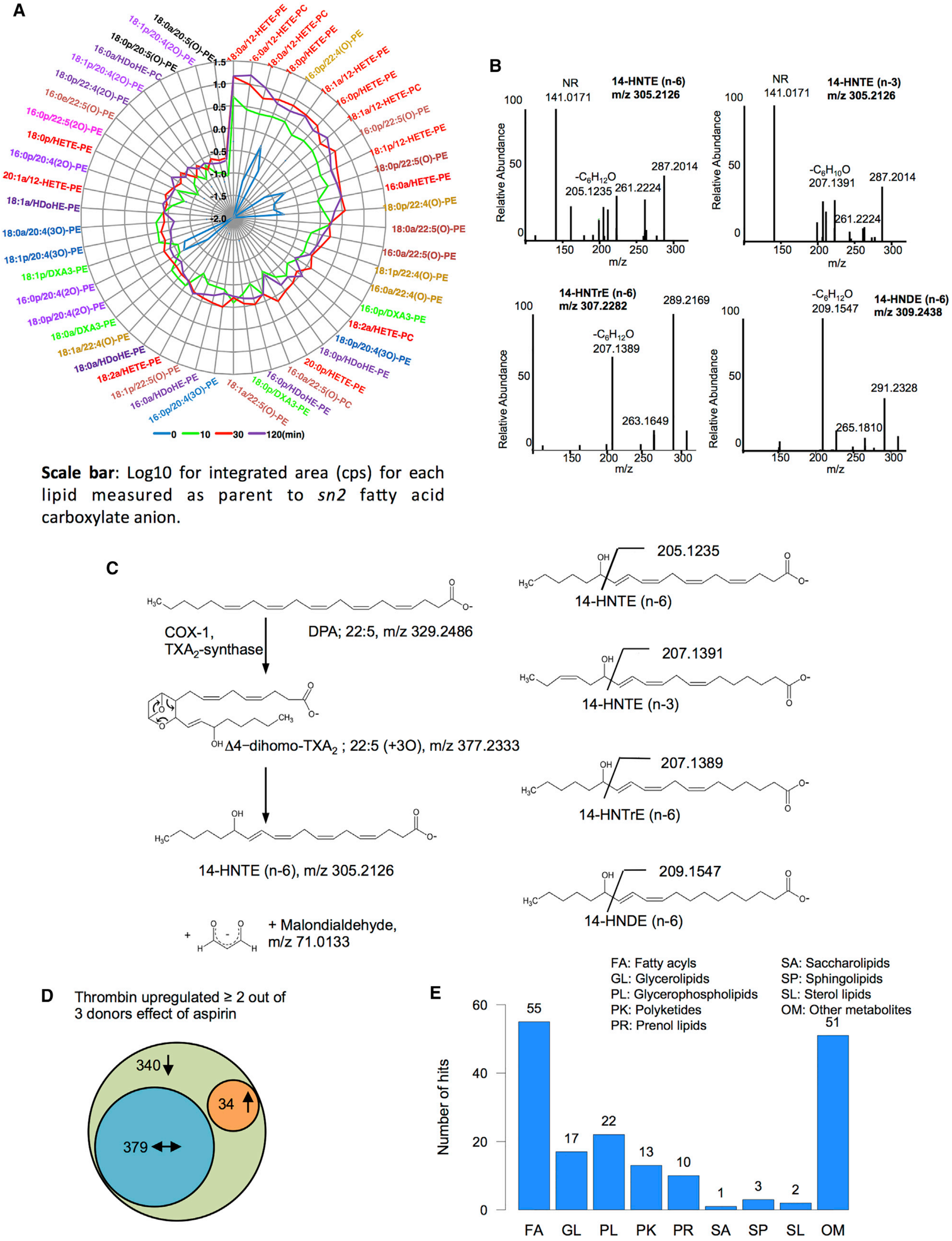

(legend on next page)

EPA). Last, donor 3 shows no elevation of any FAs by aspirin. This stimulation of FA release by aspirin in two donors occurs in the absence of COX activation and aggregation, and the mechanisms involved deserve further study.

In terms of oxidized FA, COX products tend to cluster with lipids that show the greatest inhibition by aspirin, while LOX products tend to show medium to low aspirin suppression (Figure 4). Monohydroxy fatty acids and other oxidized lipids are evenly spread, indicating they may originate from either pathway.

These data are also shown using a network analysis (Cytoscape 3.2.1). Nodes represent individual lipids, where degree (size) is determined by the number of links to others. Edge thickness represents the strength of correlation between individual nodes. Nodes with the highest degree clustered toward the center. These network diagrams illustrate that while there are clear donor variations, FA fluxes are significantly interconnected, with a major part of the pool changing in tandem. Related lipids cluster together, e.g., pink COX products always cluster toward the top left, while navy blue LOX products are further right. Unoxidized fatty acids link together but tended to show divergent responses to oxidized ones (Figure 4). Raw data on integration of all these lipids are presented in Data S6 to enable comparison of relative abundance of individual lipid species.

We also characterized the effect of aspirin on validated oxPLs, screening the 70 that were identified by the initial Sieve analysis, and found that the majority were unchanged or upregulated during aspirin treatment (comparing the thrombin with thrombin + aspirin datasets) (Data S1, tab 3). This agrees with the general view that aspirin selectively regulates platelet aggregation, but not coagulation.

Effect of cPLA$_2$ Blockade on FA and OxPL Generation

Platelets express several isoforms of PLA$_2$; thus, their role in regulating generation of FA and oxPL families was determined using inhibitors; for calcium-sensitive (cPLA$_2$; cPLA$_2$i), calcium-insensitive PLA$_2$ (iPLA$_2$; bromoenol lactone [BEL]), and secretory PLA$_2$ (sPLA$_2$; oleyloxyethylphosphocholine [OOEPC]). Overall, thrombin-stimulated generation of COX, LOX products, and FAs was effectively suppressed by cPLA$_2$ inhibition but insensitive to iPLA$_2$ or sPLA$_2$ blockade (Figures 5A, 5B, S3, and S4). With cPLA$_2$ blockade, free AA was inhibited to baseline levels (Figures 5A and S3B). However, platelets already contain free AA before activation, thus allowing up to 50% generation of COX or LOX products. OxPL generation was only partially sensitive to inhibition of cPLA$_2$ but insensitive to iPLA$_2$ blockade (Figures 5 C and S3D; individual isomers in Figure S5). Thus, sufficient eicosanoids are generated to allow formation oxPLs, even in the absence of AA release. Sensitivity of oxPLs, but not FAs, to sPLA$_2$ inhibition was also seen (see Figure 5C for a summary and Figure S5 for all individual lipids). This suggests sPLA$_2$ maybe the source of lyso-PL required for rapid eicosanoid esterification into PL pools.

Several saturated FAs were elevated in response to thrombin, although there was donor variability (Figure 4). These likely originate from the *sn1* position of PLs and may also be cleaved by the cPLA$_2\alpha$ isoform, which is known to also have PLA$_1$ activity (Loo et al., 1997). Platelets contain a large proportion of plasmalogen PLs and thus would not be considered a major source of hydrolysable saturated FAs. Biological actions and the cellular source of these lipids in the context of vascular health warrants further study.

Blockade of cPLA$_2$ Suppresses Basal- and Thrombin-Linked Mitochondrial Respiration, Linking Lipidomic Flux with Metabolism in Platelets

The diversity of FAs upregulated on platelet activation prompted further investigation. FAs are substrates for mitochondrial β-oxidation, which provides acetyl-coenzyme A for the citric acid cycle. We recently showed that oxygen consumption increases acutely on thrombin activation and is primarily supported by energy from β-oxidation (Ravi et al., 2015). Also, β-oxidation along with glycolysis is required for full aggregation to occur (Ravi et al., 2015). Thus, we queried if mobilization of FAs by cPLA$_2$ upon thrombin activation might feed into acute regulation of mitochondrial respiration, linking lipidomic dynamics with acute changes in energy metabolism.

Accordingly, extracellular flux analysis showed that platelet basal oxygen consumption rate (OCR) decreased 20% with cPLA$_2$i (Figure 5D). Thrombin then stimulated OCR, largely due to increased mitochondrial FA oxidation (Ravi et al., 2015). cPLA$_2$i almost totally blocked thrombin stimulation of OCR compared to control (Figures 5D and 5E). To confirm the oxidative phosphorylation component, at 20–30 min, oligomycin was injected to inhibit complex V, leading to the expected decrease in OCR. Then, FCCP, a proton-ionophore, was injected to stimulate maximal respiration, leading to an increase in maximal OCR, but to a smaller extent in the cPLA$_2$i-treated platelets (Figure 5D). Finally, antimycin A was added. ATP-linked respiration was calculated by subtracting OCR after oligomycin addition from basal. As shown recently, thrombin increased ATP-linked respiration, indicating an increased energetic requirement for aggregation (Figure 5F) (Ravi et al., 2015). Blockade of cPLA$_2$ decreased this, both basally and in the

Figure 3. Radar Plot Showing Relative Levels of OxPLs Generated by Platelets, Identification of Four Oxidized FAs Generated via COX-1/TXS, and Effect of Aspirin on the Platelet Lipidome

(A) Radar plot showing time course of the 52 most upregulated oxPLs. The lipids are color-coded based on sn2 fatty acid, and show that monohydroxylipids of AA, 22:4, and 22:5 predominate (red, brown, and burgundy labels are on right-hand side of plot), while poly-hydroxylated FAs are less abundant.

(B) MS/MS spectra of four lipids identified as 14-HNTE (n6), 14-HNTE (n3), 14-HNTrE (n6), and 14-HNDE (n6). Note a major ion, identified in (C). NR, not related (an ion from an unrelated isobaric lipid).

(C) Mechanism of formation of 14-HNTE (n6) (left) and fragmentation of all four isomers (right).

(D) Aspirin blocks generation of 45% of platelet lipids upregulated by thrombin. Platelet lipid extracts from three genetically unrelated donors with or without 7 days on 75 mg/day aspirin were analyzed after activation for 30 min using 0.2 U/mL thrombin, as in Figure 1. Ions noted in Figure 1G to be upregulated by at least 2-fold in two or more donors are shown.

(E) Predominant lipid classes upregulated by thrombin and aspirin inhibited in platelets. Putative ions were grouped according to the most predominant classification family based on classifications in LipidMaps, LipidHome, or HMDB, as for Figure 1C.

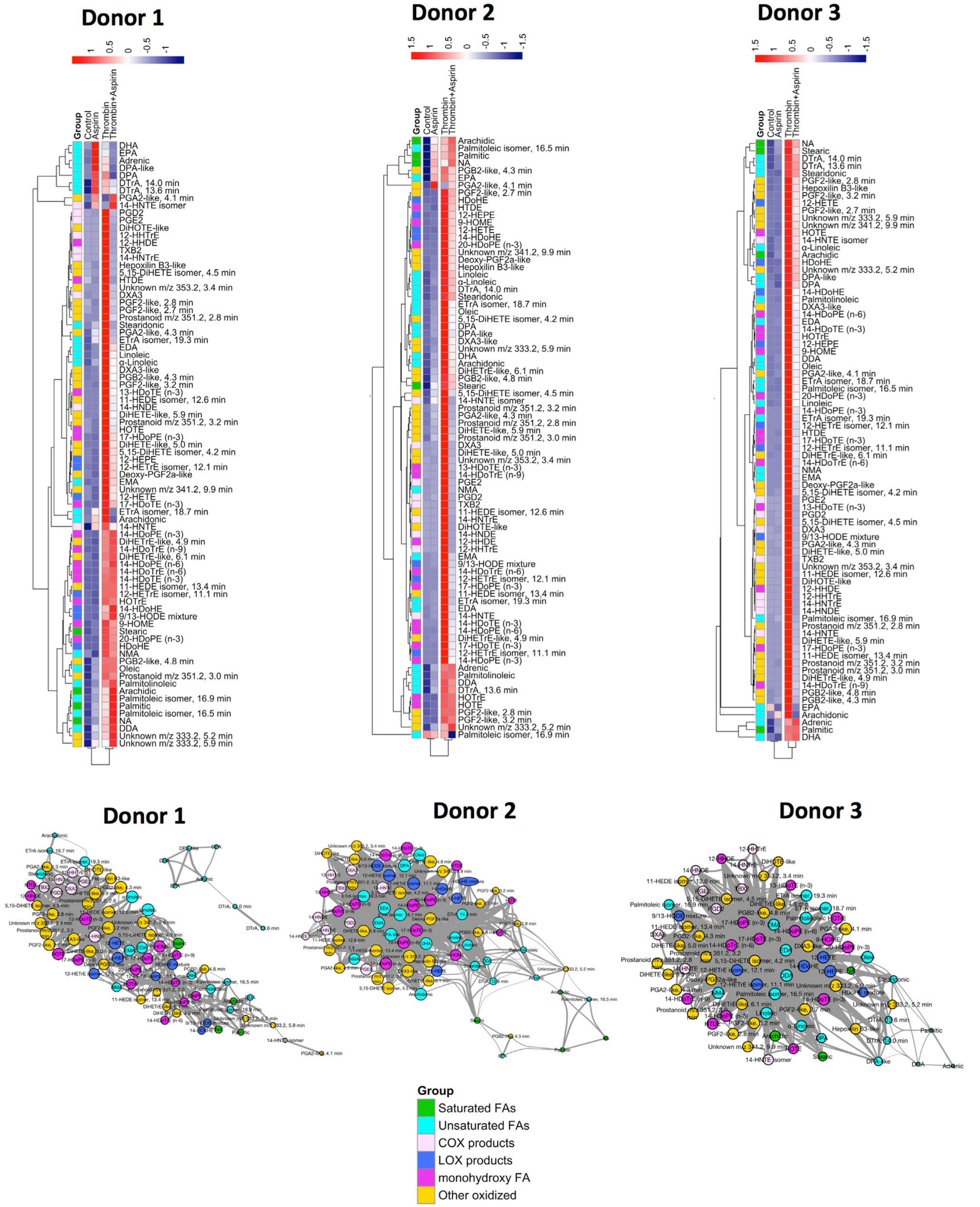

(legend on next page)

presence of thrombin, and in the presence of $cPLA_2i$, thrombin was unable to fully increase ATP-linked OCR (Figure 5F). This indicates that $cPLA_2$ activity is required for ATP-linked oxygen consumption, both basally and on activation. Proton leak, calculated by subtracting OCR post-antimycin A from OCR post-oligomycin, was unchanged in all groups (Figure 5F). Reserve capacity was calculated by subtracting OCR before oligomycin from OCR after FCCP. As shown recently, thrombin decreases reserve capacity, since it is used to provide the mitochondrial function for the increased energy demand during aggregation, and we found this also occurred during $cPLA_2$ inhibition, but to a lesser extent (Figure 5F) (Ravi et al., 2015). $cPLA_2$ inhibition decreased reserve compared to basal (Figure 5F). Taken together, these data show that blockade of $cPLA_2$ decreases mitochondrial electron transfer and lowers the cells' ability to respond to increased energetic demands. It is fully consistent with a lower availability of energy-generating substrates in the absence of sufficient FAs provided by $cPLA_2$.

Glycolysis was measured as the extracellular acidification rate (ECAR). We recently showed that ~70% of the basal and thrombin-stimulated ECAR is inhibited by 2-deoxy-D-glucose (2DG), consistent with a high rate of glycolysis in platelets (Ravi et al., 2015). Both the basal and thrombin-stimulated ECARs were unchanged by $cPLA_2i$ (50 nM), indicating that they were not influenced by lipid metabolism (Figure 5G).

Several Eicosanoids and FAs Act as Substrates for Mitochondrial β-Oxidation during Platelet Activation

We sought to determine flux of eicosanoids and FAs through β-oxidation in platelets using the carnitine palmitoyltransferase inhibitor etomoxir, which prevents transfer of FA across the inner mitochondrial membrane. Data are summarized in heatmaps with bar charts as Supplemental Information (Figures 6A and S6). In the resting platelet, increases in saturated and unsaturated FAs upon etomoxir inhibition show that these are ongoing β-oxidation substrates. However, thrombin-stimulated platelets utilized these and many oxidized eicosanoids at higher rates, as shown by the stronger response to etomoxir. Last, etomoxir elevated eicosanoids less when $cPLA_2$ was inhibited (Figures 6A and S6).

Platelet β-Oxidation and Glycolysis Are Required for FA and Eicosanoid Elevations during Platelet Activation, Representing a Positive Feedback Cycle Where Energy Metabolism Is Required for Acute Lipidomic Flux

Next, we sought to determine eicosanoid and FA flux via β-oxidation when glycolysis was blocked (glucose-free media + 2DG). Here, the expected thrombin-stimulated eicosanoid burst was absent (Figures 6B and S7). Furthermore, etomoxir failed to elevate eicosanoids or FAs, indicating that instead of accelerated consumption, there was a failure to generate FAs and eicosanoids when energy production from glycolysis was inhibited.

Blocking β-Oxidation Leads to Aminophospholipid Externalization on the Platelet Surface

Since β-oxidation was recently shown to be required for full aggregation, we instead determined whether it was required for an additional energy-requiring pathway in platelets: maintenance of phospholipid asymmetry (Ravi et al., 2015). In this, flippases and floppases use ATP to keep PC on the outer leaflet of the plasma membrane and aminophospholipids (aPLs), phosphatidylserine (PS), and PE on the inner leaflet. Asymmetry is required for preventing aPL externalization, an event that occurs during platelet aggregation, apoptosis, and aging (storage lesion), supporting thrombosis and platelet clearance. This is measured using Annexin V, which binds to the aPL headgroups. Over time, etomoxir alone led to a small, but not significant, increase in aPL externalization, where glycolysis was able to compensate for blockade of β-oxidation (Figure 6C). In contrast, when glycolysis was blocked (glucose free medium + 2DG), large elevations in aPL externalization were noted. These were significantly enhanced by etomoxir, suggesting that β-oxidation is upregulated to compensate for the lack of glycolysis. Collectively, these data establish a functional link between β-oxidation/$cPLA_2$ and mitochondrial metabolism that further extends our knowledge of how acute innate immune activation links to essential energy-requiring processes in platelets.

DISCUSSION

Currently, lipidomic approaches that simultaneously profile all lipids in the cellular or tissue lipidome, while ensuring that interfering artifact signals (e.g., isotopes, adducts, contaminating ions, and split-peak ions) have been thoroughly and robustly removed, are not widely available. This has made estimating the total lipidome in any mammalian cell extremely difficult. Herein, we generated and applied new informatics tools to address this gap. We estimated the total number and diversity of lipids in platelets and structurally identified many molecular species. An open resource is presented, allowing further mining of the lipidome for potential transducers of platelet-dependent activities, and it is described in more detail below.

We recently showed that β-oxidation contributes to the extra platelet energy demands that occur on activation (Ravi et al., 2015). Herein, we demonstrate using etomoxir that the substrates required for this are exclusively provided by $cPLA_2$ (Figures 5D–5F). This links platelet lipid mobilization to acute mitochondrial bioenergetics and reveals a function for mitochondrial fatty oxidation in modulating the cellular lipidome (Figure 6D). A dynamic cycle is revealed where, via $cPLA_2$, platelets dramatically

Figure 4. Heatmaps and Network Analysis Showing the Diversity of Thrombin and Aspirin Responses for Free and Oxidized FAs Generated by Three Genetically Unrelated Donors

Heatmaps were generated using full-scan FTMS data for each donor using the pheatmap package in R, and lipids are color-coded according to lipid group. Levels of treatment response are represented by a color gradient ranging from blue (decrease in response) to white (no change) to red (increase in response). Lipids are color-coded by group and clustered by similarity in overall response to the treatments. Network analysis was done using Cytoscape 3.2.1. For each donor, pairwise correlations between lipids were calculated in R. Nodes represent individual lipids, and degree (size) is the number of links. Edge thickness represents strength of correlation between nodes. Full data are available in Data S6 for all individual lipids.

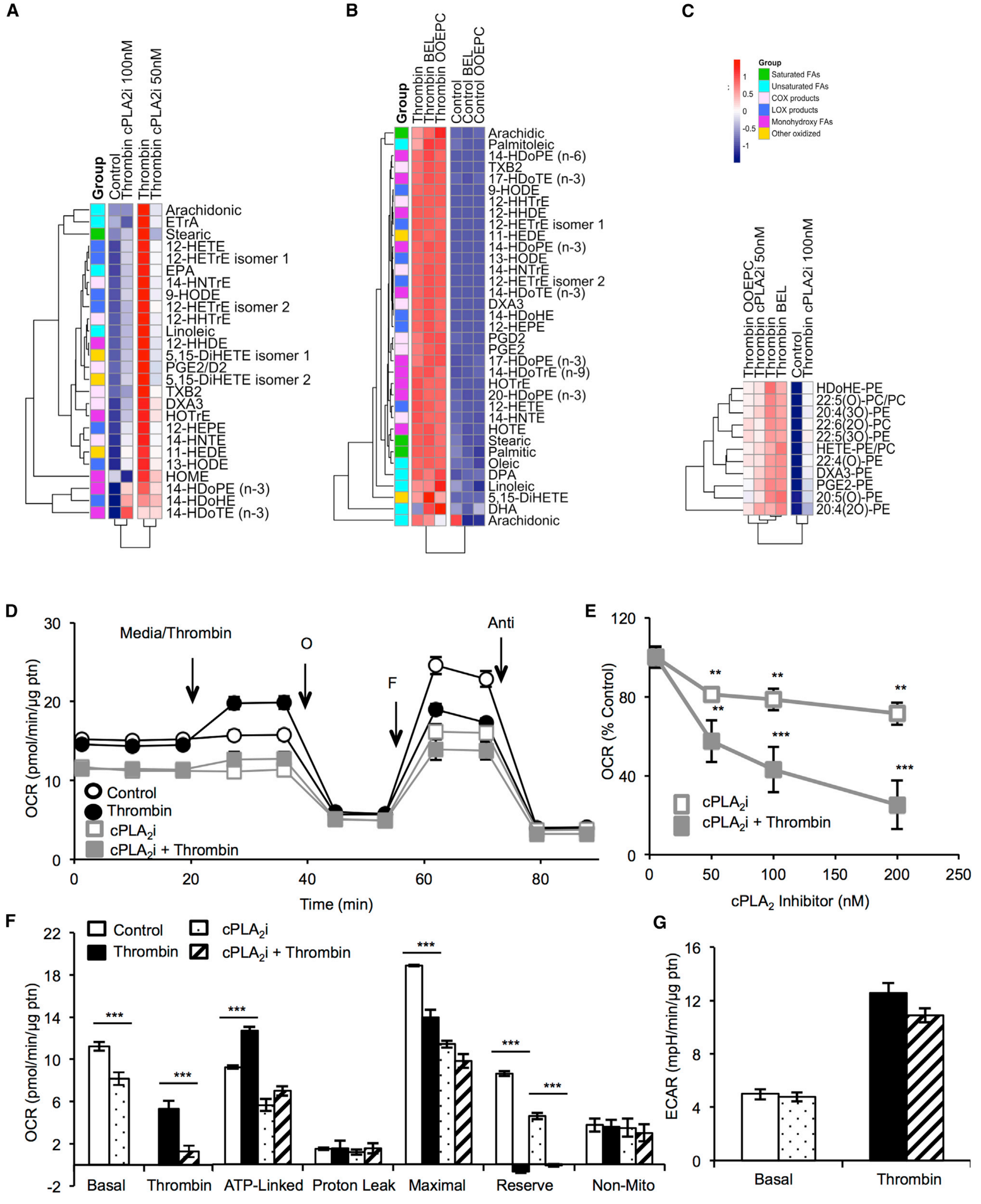

Figure 5. Thrombin-Stimulated Generation of FAs, Eicosanoids, OxPLs, and Mitochondrial Oxygen Consumption Is Sensitive to $cPLA_2$ Inhibition, while $sPLA_2$ Inhibition Suppresses OxPLs Only

Heatmaps were generated using the pheatmap package in R and lipids color-coded according to lipid group. Levels of treatment response are represented by a color gradient ranging from blue to red (increase in response). Lipids are color-coded by group and clustered by similarity in overall response to the treatments

(legend continued on next page)

upregulate both FA release and oxidation as well as their removal via β-oxidation on thrombin activation (Figure 6D). Several eicosanoids and FAs contributed to energy generation (Figures 6A and 6B). We note some apparent donor variability in terms of which FA and eicosanoids were utilized (Figures 6A and 6B). This may relate to differences in substrate supply for carnitine palmitoylacyltransferase between donors. Unexpectedly, when $cPLA_2$ was inactivated, fewer eicosanoids were subsequently removed through β-oxidation (Figure S6, top). Thus, the ability of this pathway to consume eicosanoids is highly sensitive to their generation rates and thus, most likely, their availability as substrates.

When energy generation was blocked (via both glycolysis and β-oxidation inhibition), levels of eicosanoids fell dramatically (Figures 6B and S7). However, this was due to inhibition of their generation and not their increased consumption. This reveals a positive feedback loop where eicosanoid generation itself requires energy, as well as a dynamic mechanism for removal of eicosanoids that may also function to downregulate their signaling actions. This unexpected finding may be due to the requirement for ATP as a kinase substrate, e.g., for *src* tyrosine kinases and mitogen-activated protein kinase that are activated in response to thrombin upstream of $cPLA_2$. Further studies are required in order to delineate this. Conducting a global lipidomic screen would determine whether this observation relates to lipidomic flux in general or is restricted to the effects on $cPLA_2$ seen herein. To our knowledge, energetic communication or coupling between mitochondrial respiration and eicosanoid generation has not been reported for any cell type. Of relevance, we note that mitochondrial respiration and phosphorylation have been observed to be functionally coupled for nuclear transport (Dzeja et al., 2002).

To expand the functional relevance, the role of eicosanoids in supporting energy-dependent maintenance of phospholipid asymmetry in platelets via β-oxidation was characterized. Blocking β-oxidation inhibited asymmetry maintenance, particularly when combined with glycolysis inhibition. This form of anergy could be important if FA supply becomes limiting, e.g., at later time points following activation of cells during inflammation/injury, and may contribute to shutting down cell activities, promoting coagulation and clearance of dying and apoptotic platelets. We note that levels of lipids presented herein represent a steady state, impacted by both generation and removal rates, and thus do not provide information on total flux through the pathway. Thus, the actual amounts mobilized and ultimately used as substrate for energy generation are not yet known. It is possible that a reliance on phospholipids to meet energy requirements could deplete the cell of essential structural building blocks. However, phospholipids are the predominant platelet lipid class, and these cells are well known to undergo significant remodeling of their membranes during activation. Furthermore, once trapped in a clot, they are considered to have mediated their primary function and are ultimately removed by macrophage efferocytosis.

Our characterization is a much closer approximation compared to previous studies of the total platelet lipidome (herein, defining lipids as hydrophobic molecules extracted using standard lipidomic extraction protocols), in terms of basal size and diversity, and represents a best estimate with the following limitations: (1) instrument sensitivity may prevent all lipids being detected; (2) despite major manual efforts, insufficient cleanup of datasets may lead to some overestimation; (3) some lipid classes are difficult to extract and analyze using standard methods and maybe under-represented (e.g., some phosphoinositides, oxysterols, and sphingolipids); and (4) the dataset may include some unidentified species not classed as lipids (e.g., hydrophobic peptides). However, even with these limitations, we believe our strategy offers a major step forward in defining the total lipidome of a mammalian cell and demonstrates several findings about both diversity and dynamic changes in lipids during cell activation and inhibition. Many lipids from the most abundant classes were found, as well as thrombin-upregulated lipids in the low-nanogram range (PGE_2, PGD_2, and esterified eicosanoids), validating both the coverage and sensitivity of the approach. While several automated and manual steps to remove artifact ions were included, further optimization is still required. For example, we are currently implementing a machine-learning approach to parameter optimization that will further enhance data quality, and consolidating LC analysis into a single separation.

Given the size of lipidomics datasets, it is not feasible to structurally validate every ion. Thus, we selected a smaller number of ions for detailed structural characterization. We fully identified and established triple-quadrupole-based assays for 111 oxPLs and 81 FAs generated on thrombin activation, including several that have never been detected before in platelets or other mammalian cells. While a small number of oxPLs have been found previously, we show that membrane PL oxidation is a far more diverse and wide-ranging process in platelets than previously considered (Thomas et al., 2010). The most abundant oxPLs containing HETEs are pro-coagulant both in vitro and in vivo, through altering membrane charge and facilitating coagulation factor binding at the cell surface (Thomas et al., 2010; unpublished data). Thus, global oxidation of the unsaturated platelet membrane PL pool is considerable, may support hemostasis, and is analogous to the lipid whisker model proposed by Hazen and colleagues whereby activated macrophages become coated in oxPLs and visible to lipid scavenger receptors (Greenberg et al., 2008).

(A–C) Effect of PLA_2 inhibitors on generation of FAs and oxPLs by platelets. Washed platelets were incubated with inhibitors or vehicle for 15 min, then activated using 0.2 U/mL thrombin for 30 min and generation of lipids determined using LC/MS/MS as described in Supplemental Information. Inhibitors were as follows: (A) $cPLA_2$, 50–100 nM $cPLA_2$i; (B) $iPLA_2$, 50 nM BEL; and $sPLA_2$, 2 μM OOEPC. (C) Inhibition of oxPLs by PLA_2 inhibitors. oxPLs are grouped depending on the *sn2* fatty acid to aid data visualization. Results are shown as heatmaps, with full data in Figures S3–S5.

(D–G) $cPLA_2$ inhibition suppresses mitochondrial oxygen consumption, but not glycolysis. Platelets were plated on Cell-Tak-coated XF96 plates, and pre-treated with $cPLA_2$i (<200 nM) for 15 min prior to bioenergetic measurements. (D) Basal OCR of platelets was measured prior to injection of thrombin (0.5 U/mL), followed by 1 μg/mL oligomycin (O), 0.6 μM FCCP (F), and 10 μM antimycin A (Anti). (E) $cPLA_2$i (0–200 nM) dose response of thrombin-linked OCR presented as a percentage of control. (F) Indices of mitochondrial function, basal, thrombin responsive, ATP-linked, proton leak, maximal, reserve capacity, and non-mitochondrial OCR were calculated. (G) Basal and thrombin-responsive ECARs were calculated from parallel ECAR measurements. Data expressed as mean ± SEM from one representative donor, n = 3–5 replicates per sample. *$p < 0.05$, **$p < 0.01$, and ***$p < 0.005$ different from control (ANOVA and Bonferroni post hoc test).

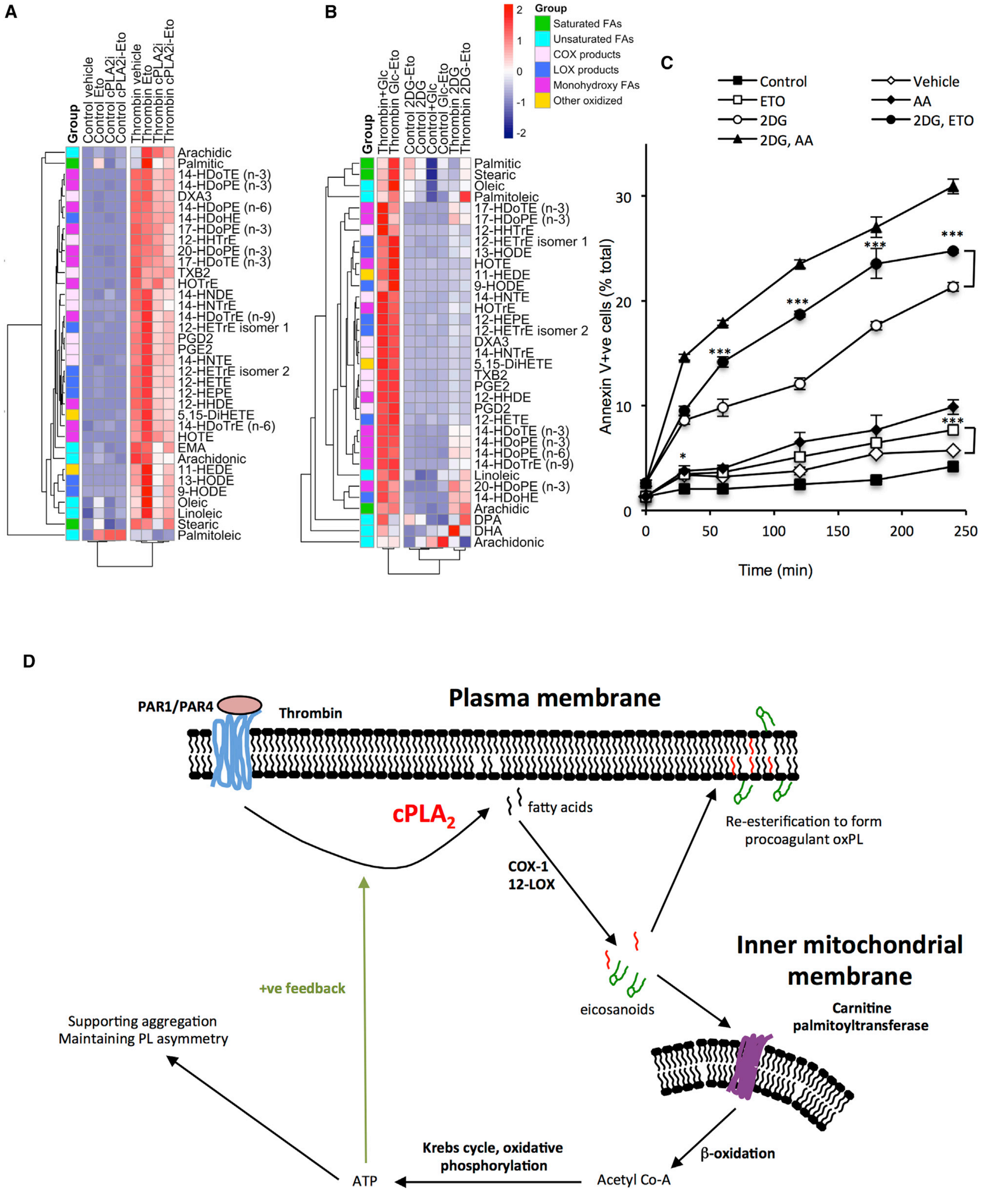

Figure 6. Eicosanoids and Fatty Acids Acutely Feed into β-Oxidation during Platelet Activation and Support Maintenance of Membrane Phospholipid Asymmetry

Heatmaps were generated using the pheatmap package in R and lipids color-coded according to lipid group. Levels of treatment response are represented by a color gradient ranging from blue (decrease in response) to white (no change) to red (increase in response). Lipids are color-coded by group and clustered by similarity in overall response to the treatments.

(legend continued on next page)

Three genetically unrelated donors showed considerable diversity in their basal and thrombin-stimulated lipidomes and responses to aspirin (Figures 1 and 4). Furthermore, one showed far higher activation by thrombin in terms of the number or amount of lipids generated (donor 3), while another showed far less inhibition of the thrombin-stimulated lipid pool by aspirin (donor 1), despite full blockade of the primary COX-1-derived eicosanoids TX and 12-HHTrE. Low-dose aspirin is considered platelet COX-1 selective, through acetylation of the active site. However, many aspirin-inhibitable lipids, including FAs, are generated via phospholipases upstream of COX-1. This underscores the importance of secondary activation as a positive feedback mechanism during platelet-aggregation-associated lipid mobilization. Specifically, COX-1-generated TXA_2 activates the thromboxane receptor (TP), inducing release of secondary mediators such as ADP that then potentiate the thrombin-mediated global lipidomic changes observed. We also noted aspirin upregulation of FAs that was highly donor specific (Figure 4). How lipid variations are controlled at a genetic and environmental level is not understood and will require further study on the lipidomes of large numbers of people and in different cells or tissues. In particular, given the importance of aspirin as both a cardioprotective and possible cancer therapeutic, a full understanding of how it regulates platelet lipids will be the focus of a follow-up study with a larger number of volunteers. The stability of the global lipidome with age, diet, and over time is unknown, and the influence of external factors such as epigenetic control of lipid-metabolizing enzymes could be considerable. Of relevance, it was recently shown that cellular immune systems are highly variable between individuals but remain remarkably constant over time (Carr et al., 2016). Environmental factors were a key influence, and during infection, transient changes were observed that quickly reverted to baseline. Our understanding of intra and inter-individual variability in terms human cell biology of the immune and blood systems is in its infancy but currently a major topic of research interest. Our study represents a first look at a small group but importantly provides a platform for future longitudinal studies of platelet lipidomics.

The platelet lipidome contains large numbers of species that are absent from current databases (Figures S1A and S1C). These included structures characterized herein, such 14-HDTE, HDTrE, and HNDE, and many oxPLs. Most of the unknowns are above 1 kDa, and could include some classes absent from databases, such as glycolipids (Figure 1D). Developing informatics workflows to assist with identification of specific families from these large numbers of unknowns will be a major future endeavor. The presence of 14-hydroxy C19 lipids was intriguing, since platelets contain no detectable C19 unsaturated substrate (Figure 3). They were largely inhibited by aspirin, suggesting they originate via COX-1 (Data S1). Platelets generate significant amounts of the C17 12-hydroxyheptadecatrienoic acid (12-HHTrE) due to degradation of thromboxane A2 (TXA_2), where part of the prostanoid ring is lost (malondialdehyde), generating the C17 monohydroxy lipid. We reasoned that platelets might generate analogous lipids through metabolizing C22 substrates with three, four, or five double bonds. Indeed, previous studies found exogenous C22 substrate can be metabolized by COX (Milks and Sprecher, 1985; Sprecher, 1986; Sprecher and Careaga, 1986); however, their generation from endogenous substrate on agonist activation has never been shown. Analogous lipids from 22:6 were not detected, and ions giving MS/MS fragmentation spectra consistent with resolvins, lipoxins (LXs), or protectins were absent from our dataset. While some lipids with parent *m/z* 351.2180 suggestive of lipoxins were detected (Data S5 [p28] and Data S6 [p 15, top three bars]), their MS/MS spectra were distinct from either LXA_2 or B_2. These were highly sensitive to aspirin inhibition, indicating they likely originated via COX-1. We propose these to be additional prostanoids based on the presence of some common daughter ions, but at this stage, their structures are unknown. Thus, pro-resolving mediators, including aspirin-triggered lipoxins, were not generated by acutely activated platelets.

In summary, we surveyed the global diversity of human lipids in a single cell type, including both knowns and unknowns and to a high degree of sensitivity. We then used the information to discover many lipids and link acute lipidomic changes with metabolism. Specifically, $cPLA_2$ blockade acutely decreased β-oxidation and mitochondrial respiration, indicating a link between inflammatory signaling and regulation of the cellular metabolome. These data represent the first full estimate of total lipid molecular species for any cell type to date.

EXPERIMENTAL PROCEDURES

Chemicals

These are provided in Supplemental Information.

Human Platelet Isolation, Activation, and Inhibition

For lipidomic studies, platelets were isolated from three genetically unrelated healthy volunteers, as described in Supplemental Information. For baseline samples, donors were free from nonsteroidal anti-inflammatory drugs for at least 14 days and the study was approved by the Cardiff University School of Medicine Ethics Committee (SMREC 12/13). For aspirinized samples, the same donors were administered 75 mg/day aspirin for 7 days before donation. For mitochondria experiments, platelets were isolated from concentrates

(A) Several eicosanoids are substrates for platelet β-oxidation on thrombin generation. Washed platelets were incubated with inhibitors or vehicle at room temperature (RT), then activated using 0.2 U/mL thrombin at 37°C for 30 min and generation of lipids determined using LC-MS/MS as described in Supplemental Information (n = 3). Inhibitors were as follows: etomoxir (Eto), 25 μM; $cPLA_{2}i$, 100 nM.

(B) Inhibition of glycolysis leads to a failure to generate free fatty acids and eicosanoids on thrombin activation of platelets. Washed platelets were incubated with inhibitors or vehicle at RT, then activated using 0.2 U/mL thrombin at 37°C for 30 min, with generation of lipids determined using LC-MS/MS as described in Supplemental Information (n = 3). Cells were incubated in the presence or absence of 5 mM glucose (Glc). Inhibitors were as follows: Eto, 25 μM; 2-deoxy-D-glucose (2DG), 120 mM.

(C) Blocking β-oxidation leads to a failure to maintain phospholipid asymmetry in platelets. Washed platelets were incubated with inhibitors or vehicle for up to 240 min at RT, then stained for PS using Annexin V-FITC, for 15 min at RT in the dark, and analyzed by flow cytometry. Inhibitors were as follows: Eto, 25 μM; antimycin A (Anti), 10 μM; 2DG, 120 mM. Data were analyzed using one-way ANOVA with Bonferroni post hoc test (n = 3, mean ± SEM), comparing Eto with vehicle and Eto+2DG with 2DG.

(D) Scheme for proposed role of $cPLA_2$ in acutely feeding substrates into mitochondrial oxidative phosphorylation and generation of oxPLs.

obtained from the blood bank by centrifugation then washed 2× with PBS and counted as described previously (Chacko et al., 2013). Collection and use of these samples was approved by University of Alabama at Birmingham Institutional Review Board.

Lipid Extraction

Lipids were extracted as described in Supplemental Information, resuspended in 200 μL methanol and stored at −80°C until analysis. For time course and MRM experiments, internal standards (at 10 ng each of PGE2-*d4*, 12-HETE-*d8*, DMPE, and DMPC) were added to the extraction solvent of time course activation.

Global Lipidomics and Analysis of Lipids

Untargeted global analysis of lipids was performed, separately in positive- or negative-ion mode, using LC coupled to an Orbitrap Elite mass spectrometer (UPLC-Orbitrap Elite MS; Thermo Fisher Scientific). Lipids extracts were separated on two different reverse-phase (RP) columns and gradient methods, as described in Supplemental Information.

Targeted Analysis of Lipids

Selected lipids upregulated on thrombin activation in human platelets were analyzed for temporal dynamics of their generation using LC-MS/MS (ABI 4000 or 6500 QTRAP) by monitoring precursor-to-product ion transitions in MRM mode, as described in Supplemental Information.

Data Processing

Global scanning data, chromatographic alignment, peak or feature detection, and isotopic filtering used SIEVE 2.0 (Thermo Fisher Scientific), as described in Supplemental Information. SIEVE-aligned data were further processed using in-house-generated software as described in Supplemental Information.

Statistical Analysis and Data Visualization

Treatment effect on donor grouping was projected using PCA from mixOmics package in R. This dataset (features/mass ions common in two or more donors) was scaled to unit variance (UV). Global distribution of features and mass ions among donors was shown using VennDiagram in R (vennDiagram). Radar plots were generated using Excel. Heatmaps were generated for each donor using the pheatmap package in R, and lipids were color-coded according to lipid group. Network analysis was done using Cytoscape 3.2.1. For each donor, pairwise correlations between lipids were calculated in R. Due to the high number of interactions, the network diagram shows only positive correlations with a Pearson product-moment correlation coefficient value (r) > 0.9.

Structural Interpretation and Assignment of Lipids

Lipid species were putatively identified by matching their accurate mass to records in the following databases: (1) the HMDB (Wishart et al., 2013; http://www.hmdb.ca/), (2) Lipidhome (Foster et al., 2013; http://www.ebi.ac.uk/apweiler-srv/lipidhome/), (3) LipidMaps (Lipid Metabolites and Pathways Strategy) (Sud et al., 2007; http://www.lipidmaps.org/), and (4) METLIN (metabolite and tandem MS database) (Smith et al., 2005; http://metlin.scripps.edu/) (as described in Supplemental Information).

Seahorse Extracellular Flux Analysis

Bioenergetic measurements of platelets were performed as previously described (Chacko et al., 2013; Ravi et al., 2015). In brief, platelets suspended in XF DMEM assay buffer (DMEM with 1 mM pyruvate, 5.5 mM D-glucose, and 4 mM L-glutamine [pH 7.4]), were plated on Cell-Tak coated platelets and then pre-incubated with $cPLA_2i$ (0–200 nM) for 15 min prior to the assay. First basal bioenergetics were measured, followed by injection of either thrombin (0.5 U/mL) or media, oligomycin (1 μg/mL), FCCP (0.6 μM), and antimycin A (10 μM).

SUPPLEMENTAL INFORMATION

Supplemental Information includes Supplemental Experimental Procedures, seven figures, five tables, and six data files and can be found with this article online at http://dx.doi.org/10.1016/j.cmet.2016.04.001.

AUTHOR CONTRIBUTIONS

D.A.S., C.J.B., M.A., S.M., S.R., G.A.B., and V.B.O. conducted experiments. D.A.S., V.B.O., V.D.U., and S.A. designed experiments. A.O.C. and C.J.B. wrote software, conducted analysis, and generated figures. R.C.M. and V.D.U. analyzed data. V.B.O., D.A.S., and A.O.C. drafted the manuscript. All authors edited the manuscript.

ACKNOWLEDGMENTS

Funding from Wellcome Trust (094143/Z/10/Z) and European Research Council (LipidArrays) is gratefully acknowledged (V.B.O.). V.B.O. is an ERC Investigator. Funding from NIH (U54HL117798 to R.C.M.) is acknowledged. C.J.B. was supported by the Cardiff University Research Opportunities Programme (CUROP). Support from the UAB School of Medicine for the Blue Sky Award is gratefully acknowledged (V.D.U.).

Received: May 27, 2015
Revised: January 11, 2016
Accepted: March 31, 2016
Published: April 28, 2016

REFERENCES

Aldrovandi, M., Hammond, V.J., Podmore, H., Hornshaw, M., Clark, S.R., Marnett, L.J., Slatter, D.A., Murphy, R.C., Collins, P.W., and O'Donnell, V.B. (2013). Human platelets generate phospholipid-esterified prostaglandins via cyclooxygenase-1 that are inhibited by low dose aspirin supplementation. J. Lipid Res. *54*, 3085–3097.

Carr, E.J., Dooley, J., Garcia-Perez, J.E., Lagou, V., Lee, J.C., Wouters, C., Meyts, I., Goris, A., Boeckxstaens, G., Linterman, M.A., and Liston, A. (2016). The cellular composition of the human immune system is shaped by age and cohabitation. Nat. Immunol. *17*, 461–468.

Chacko, B.K., Kramer, P.A., Ravi, S., Johnson, M.S., Hardy, R.W., Ballinger, S.W., and Darley-Usmar, V.M. (2013). Methods for defining distinct bioenergetic profiles in platelets, lymphocytes, monocytes, and neutrophils, and the oxidative burst from human blood. Lab. Invest. *93*, 690–700.

Dzeja, P.P., Bortolon, R., Perez-Terzic, C., Holmuhamedov, E.L., and Terzic, A. (2002). Energetic communication between mitochondria and nucleus directed by catalyzed phosphotransfer. Proc. Natl. Acad. Sci. USA *99*, 10156–10161.

Foster, J.M., Moreno, P., Fabregat, A., Hermjakob, H., Steinbeck, C., Apweiler, R., Wakelam, M.J., and Vizcaíno, J.A. (2013). LipidHome: a database of theoretical lipids optimized for high throughput mass spectrometry lipidomics. PLoS ONE *8*, e61951.

Greenberg, M.E., Li, X.M., Gugiu, B.G., Gu, X., Qin, J., Salomon, R.G., and Hazen, S.L. (2008). The lipid whisker model of the structure of oxidized cell membranes. J. Biol. Chem. *283*, 2385–2396.

Langley, R.E., and Rothwell, P.M. (2013). Potential biomarker for aspirin use in colorectal cancer therapy. Nat. Rev. Clin. Oncol. *10*, 8–10.

Loo, R.W., Conde-Frieboes, K., Reynolds, L.J., and Dennis, E.A. (1997). Activation, inhibition, and regiospecificity of the lysophospholipase activity of the 85-kDa group IV cytosolic phospholipase A2. J. Biol. Chem. *272*, 19214–19219.

Milks, M.M., and Sprecher, H. (1985). Metabolism of 4,7,10,13,16-docosapentaenoic acid by human platelet cyclooxygenase and lipoxygenase. Biochim. Biophys. Acta *835*, 29–35.

Morgan, L.T., Thomas, C.P., Kühn, H., and O'Donnell, V.B. (2010). Thrombin-activated human platelets acutely generate oxidized docosahexaenoic-acid-containing phospholipids via 12-lipoxygenase. Biochem. J. *431*, 141–148.

O'Donnell, V.B., and Murphy, R.C. (2012). New families of bioactive oxidized phospholipids generated by immune cells: identification and signaling actions. Blood *120*, 1985–1992.

O'Donnell, V.B., Murphy, R.C., and Watson, S.P. (2014). Platelet lipidomics: modern day perspective on lipid discovery and characterization in platelets. Circ. Res. *114*, 1185–1203.

Ravi, S., Chacko, B., Sawada, H., Kramer, P.A., Johnson, M.S., Benavides, G.A., O'Donnell, V., Marques, M.B., and Darley-Usmar, V.M. (2015). Metabolic plasticity in resting and thrombin activated platelets. PLoS ONE *10*, e0123597.

Rothwell, P.M. (2013a). Alternate-day, low-dose aspirin and cancer risk. Ann. Intern. Med. *159*, 148–150.

Rothwell, P.M. (2013b). Aspirin in prevention of sporadic colorectal cancer: current clinical evidence and overall balance of risks and benefits. Recent Results Cancer Res. *191*, 121–142.

Smith, C.A., O'Maille, G., Want, E.J., Qin, C., Trauger, S.A., Brandon, T.R., Custodio, D.E., Abagyan, R., and Siuzdak, G. (2005). METLIN: a metabolite mass spectral database. Ther. Drug Monit. *27*, 747–751.

Sprecher, H. (1986). The metabolism of (n-3) and (n-6) fatty acids and their oxygenation by platelet cyclooxygenase and lipoxygenase. Prog. Lipid Res. *25*, 19–28.

Sprecher, H., and Careaga, M.M. (1986). Metabolism of (n-6) and (n-3) polyunsaturated fatty acids by human platelets. Prostaglandins Leukot. Med. *23*, 129–134.

Sud, M., Fahy, E., Cotter, D., Brown, A., Dennis, E.A., Glass, C.K., Merrill, A.H., Jr., Murphy, R.C., Raetz, C.R., Russell, D.W., and Subramaniam, S. (2007). LMSD: LIPID MAPS structure database. Nucleic Acids Res. *35*, D527–D532.

Thomas, C.P., Morgan, L.T., Maskrey, B.H., Murphy, R.C., Kühn, H., Hazen, S.L., Goodall, A.H., Hamali, H.A., Collins, P.W., and O'Donnell, V.B. (2010). Phospholipid-esterified eicosanoids are generated in agonist-activated human platelets and enhance tissue factor-dependent thrombin generation. J. Biol. Chem. *285*, 6891–6903.

van Meer, G. (2005). Cellular lipidomics. EMBO J. *24*, 3159–3165.

Wishart, D.S., Jewison, T., Guo, A.C., Wilson, M., Knox, C., Liu, Y., Djoumbou, Y., Mandal, R., Aziat, F., Dong, E., et al. (2013). HMDB 3.0–The Human Metabolome Database in 2013. Nucleic Acids Res. *41*, D801–D807.

Yetukuri, L., Ekroos, K., Vidal-Puig, A., and Oresic, M. (2008). Informatics and computational strategies for the study of lipids. Mol. Biosyst. *4*, 121–127.

Cell Metabolism, Volume *23*

Supplemental Information

Mapping the Human Platelet Lipidome Reveals Cytosolic Phospholipase A_2 as a Regulator of Mitochondrial Bioenergetics during Activation

David A. Slatter, Maceler Aldrovandi, Anne O'Connor, Stuart M. Allen, Christopher J. Brasher, Robert C. Murphy, Sven Mecklemann, Saranya Ravi, Victor Darley-Usmar, and Valerie B. O'Donnell

Supplementary Methods, Tables and Figures.

Supplementary Methods

Chemicals Lipid standards, 12(S)-hydroxyeicosatetraenoic acid (12s-HETE), 12-HETE-d_8, prostaglandin E_2-d_4 (PGE_2-d_4), 1-stearoyl-2-arachidonoyl-*sn*-glycero-3-phosphatidylethanolamine (18:0a/20:4-PE), 1,2-Dimyristoyl-*sn*-glycero-3-phosphatidylcholine (DMPC), 1,2-Dimyristoyl-*sn*-glycero-3-phosphatidylethanolamine (DMPE) were purchased from Avanti Polar Lipids or Cayman Chemical. Phospholipase inhibitors were from Calbiochem. HPLC grade solvents and acids were purchased from Fisher Scientific UK Ltd. All other reagents were obtained from Sigma-Aldrich unless otherwise stated.

Lipid extraction Lipids were extracted by adding a solvent mixture (1 M acetic acid/propan-2-ol/hexane; 2:20:30, v/v) to platelets at a ratio of 2.5 ml of solvent mixture/ml platelets in 10 ml extraction vial and vortexed for 30 seconds. 2.5 ml of hexane was added and then vortexed and centrifuged (500 *g* for 5 minutes at 4 °C) to recover lipids in the upper hexane layer. Aqueous samples were re-extracted by addition of 2.5 ml hexane. The combined hexane layers were dried in a RapidVap (Labconco) at room temperature.

Human platelet isolation and activation/inhibition. For baseline samples, donors were free from nonsteroidal anti-inflammatory drugs for at least 14 days and the study was approved by the Cardiff University School of Medicine Ethics Committee (SMREC 12/13). For aspirinized samples, donors were administered 75 mg/day aspirin for 7 days before donation. Briefly, blood was collected into ACD (Acid-Citrate-Dextrose; 85 mM trisodium citrate, 65 mM citric acid and 100 mM glucose) at a blood/ACD ratio of 8.1:1.9 (v/v) and centrifuged at 250 *g* for 10 minutes at room temperature (22 °C). Platelet-rich plasma was collected and centrifuged at 900 *g* for 10 minutes, and the pellet resuspended in Tyrode's buffer (134 mM NaCl, 12 mM $NaHCO_3$, 2.9 mM

KCl, 0.34 mM Na_2HPO_4, 1.0 mM $MgCl_2$, 10 mM HEPES and 5 mM glucose, pH 7.4) containing ACD (9:1, v/v). The platelets were washed by centrifuging at 800 *g* for 10 minutes then re-suspended in Tyrode's buffer at a concentration of 2 × 10^8 cells/ml. Platelets were pre-incubated with 1 mM $CaCl_2$ at 37 °C for 5 minutes and then activated by thrombin (0.2 units/ml) at 37 °C for up to 30 min. Where used, inhibitors were pre-incubated for 10 min before thrombin activation, with appropriate vehicle controls used.

Global lipidomics/analysis of lipids Two methods were used: the first (for lipophilic species, termed "Non-Polar") used a Hypersil GOLD C_{18} RP UPLC column (150 x 2.1 mm I.D., 1.9 µm particle size) with gradient of mobile phase A (acetonitrile:water, 50:50 v/v, 1 mM ammonium acetate, 0.1 % glacial acetic acid) and B (iso-propanol:acetonitrile, 70:30 v/v, 1 mM ammonium acetate, 0.1 % glacial acetic acid) at 0.4 ml/min over 55 min. The elution gradient of B (%) over time was: 35 to 50 % for 10 min, 50 to 66 % for 6 min, 66 to 76 % for 22 min, 76 to 96 % for 10 min, held at 96 % for 4.5 min then equilibrated at 35 % for 2.5 min. The second (for non-lipophilic lipids, termed "Polar") used a Spherisorb ODS2 column (150 x 2.1 mm, 3 µm particle size) with solvent gradient of mobile phase A (water:acetonitrile, 75:25, v/v, 1mM ammonium acetate and 0.1 % glacial acetic acid) and B (methanol:acetonitrile, 60:40, v/v, 1mM ammonium acetate and 0.1 % glacial acetic acid) at 0.4 ml/min over 30 min. The elution gradient of B (%) over time was: 50 to 90 % for 20 min, held at 90 % for 5.1 min and equilibrated at 50 % for 4.9 min. Samples were maintained at 4 °C and the column at 25 °C. The MS conditions were as follows: HESI-II temperature 350 °C, N_2 as drying gas, sheath gas flow 52 arbitrary units, auxiliary gas flow 17 units, capillary temp 320 °C, spray voltage +/- 3.5 kV and S-lens RF level 69.8/65.60 % respectively for positive and negative ion mode. High resolution (60,000 at 400 amu) full-scan MS spectra were acquired over 100 to 900 *m/z* or 900 to 1800 *m/z* (for both lipid separation and ion polarity methods) in centroid mode. Samples were analyzed at random, with every fifth or sixth injection of solvent blank. A one-off column conditioning at the beginning was

done with two runs of solvent blank followed by five runs of unrelated platelet extract and then two runs of solvent blank using same solvent gradient.

Developing a method to analyze the complete platelet lipidome.

To determine the complete lipidome, features from several available lipidomics methods were combined to achieve broad coverage with high sensitivity across the full range of lipids. High resolution scanning was used to maximize detection of lipids with close *m/z* values and extensive chromatography to separate isobaric species and enable detection of low abundance lipids. Two reverse phase chromatographic methods were optimized, one for relatively lipophilic (e.g. phospholipids (PL), triglycerides, neutral lipids, called “Non-Polar”) and the other for non-lipophilic species (e.g. eicosanoids, fatty acids (FA), called “Polar”). Chromatographic separation provides retention time information, important given the numerous isobaric species not distinguished using shotgun approaches. This applies not only to phospholipids/glycerides, but also for less abundant prostaglandins/eicosanoids, which can co-elute during shorter analyses. Lipids were extracted using a standard method that broadly extracts lipids from most classes (Zhang et al., 2002).

MS detection used an Orbitrap Elite in full-scan FTMS mode, at 60,000 resolution to enable sufficient scans (approx. 3 per second) to be acquired for preliminary identification of lipids, without compromising mass accuracy. Each sample was analyzed using 8 separate chromatographic runs: (i) to resolve lipophilic vs non-lipophilic lipids, (ii) in positive or negative mode, and (iii) at two mass ranges (100-900 and 900-1800 *m/z*). This enabled acquisition of enough high resolution scans to allow detection of lipids with similar mass but identical retention time to be distinguished solely based on *m/z*. Due to the comprehensive analyses, significant post acquisition processing of large data files was required (described below). For each donor, we analyzed 3-4 technical replicates (448 runs) in random order, using a blank solvent injection every 5th or 6th sample. Data files were processed using SIEVE 2.0 (ThermoFisher Scientific), for peak alignment, isotope removal, and data extraction, as described below. This allows all

features (ions) to be identified and compared between samples, based on accurate mass and retention time. Next, SIEVE-processed data was refined using an in-house custom-generated workflow that: removed noise peaks, combined features making up the same ion peak into one feature, identified and removed lipid duplicates detected by more than one run (e.g. that generate both positive and negative ions, generate adducts, or are retained by both columns). Adducts and artifact ions removed are listed in Table S1. A large number of peaks were checked manually to verify correct processing. The wokflow was initially implemented in Excel. To speed up processing and improve automation, it is currently being refactored into Python and will be available as a stand alone software package in 2016. The approach detected several well-known, but quantitatively minor, platelet lipids thus validating its sensitivity (shown later). We present GoogleVis interactive scatter diagrams for data mining and detailed visualization as Supplementary Data. These allow in-depth analysis of sub-sections of the lipidome based on *m/z,* lipid class and retention time, and are available with a short manual in Supplementary Data. We also include spreadsheets of *m/z* values and putative identifications.

For studies on aspirin inhibition, we compared the *thrombin* with *thrombin+aspirin* datasets for lipids that were suppressed at least 2-fold in 2 or more donors. All 753 thrombin-upregulated ions were manually verified to ensure data accuracy.

Data processing SIEVE 2.0 (ThermoFisher) parameters for chromatographic alignment and framing (feature identifier) were optimized according to the column type. SIEVE first aligns data from multiple runs, removing isotopes. It finds the highest intensity mass/time peak in all the aligned runs. All the counts within a specified time (“retention time width” or RT width) and mass tolerance (5 ppm) of that mass/time maxima are summed for each run in the analysis, and are extracted that as a “frame”. SIEVE then iteratively extracts additional frames until the highest remaining intensity peak is below a specified count value, or a specified frame maximum is reached. Because of this methodology, SIEVE does not attempt to group frames into peaks, or distinguish frames representing artifacts or solvent as opposed to lipids.

For non-lipophilic lipids separated on the ODS2 RP column (Polar), RT width was 0.4 min over 1.0 to 28.0 min and intensity threshold of 1000 counts. For lipophilic lipids separated on the HypersilGold RP column (Non-Polar), RT width was 0.8 min over 1.0 to 50.0 min and intensity threshold 1000 counts.

SIEVE aligned data was further processed using our in-house generated Excel-based workflow to eliminate solvent-like features, based-on compliance of at least three parameters from retention time-sorted accurate mass groups (mass tolerance 5 ppm), (1) mass ion/feature is continuously present over 1.2 min, (2) variance of mean peak intensity over this time is relatively low (SEM < 20 %), (3) more than 6 features with same accurate mass and (4) standard deviation of the elution times from all elements in mass group is more than 1 min. Next, peak finding was undertaken, based on local feature/frame maxima and concatenation of features separated due to occasional chromatographic shifts. Only single frames with highest peak intensity on either side of local maxima and within half-time of RT framing were added to the frame with local maxima. Next, adduct ions of low abundance within positive and negative mode data sets were removed. Elimination was only undertaken where adduct ions eluted at the same time as the molecular ion. Adduct species were removed from positive and negative ion data sets, along with additional contaminating *m/z* ions forming either a series of resolved or stacked multiple adduct species (see Table S1 for full details). Next, RT-matched redundant or duplicate features detected between positive and negative ion mode from same column type data set with low average peak intensity were eliminated. Early eluting lipid species/mass ions on the Hypersil Gold column were retained on the ODS2 column. These early eluting duplicate mass ions were eliminated from the Hypersil Gold column by matching progressive RT shift. The total number of unique features in resting and thrombin-treated platelets within all donors was estimated as follows. Features/mass ions above a fixed intensity threshold (200 counts for polar and 500 counts for non-polar) with relative standard deviation (RSD) below 35 % from resting and thrombin-treated data sets were retained. Differentially changed features/mass ions upon thrombin-activation from all three donors were identified by applying a 2-fold threshold.

Upregulated features/mass ions common to at least two out of three donors and with highest average peak intensity were used for database searches.

Structural interpretation and assignment of lipids

Lipid species were putatively identified by matching their accurate mass to records in the following databases: (1) the Human metabolome database (HMDB) (Wishart et al., 2013): http://www.hmdb.ca/, (2) Lipidhome (Foster et al., 2013): http://www.ebi.ac.uk/metabolights/lipidhome/, (3) LipidMaps (Lipid metabolites and pathways strategy) (Sud et al., 2007): http://www.lipidmaps.org/, *(4)* METLIN (metabolite and tandem MS database) (Smith et al., 2005): http://metlin.scripps.edu/ as described in Supplementary Data. To automate the identification process a python program was developed to search 3 of these databases (HMDB, Lipidhome and LipidMaps). METLIN was searched manually in batch mode (maximum 500 masses in one batch). A query was generated against each database using mass value, ion mode (plus adduct types) and MW tolerance (±0.005 Da). The only exception was LipidMaps, which does not allow for adduct ion selection. Thus, for LipidMaps, masses were converted to neutral (from positive or negative ions) prior to query. Then, resulting masses were converted back to positive or negative mode after the search. Output files were saved in Microsoft Excel .csv format. Additionally, a .csv file was produced for each database listing the mass values for which no match was found. Each record includes mass error, retention time and polarity. Output files were merged, during which entries were standardized. Entries were removed where the mass error value was ≥ ± 5 ppm. In addition, only entries containing adducts listed in Table S2 were retained, all other entries were removed. Lastly, duplicate records with the same mass and identification, but originating from a different database were removed. For this, the record with the lowest mass error value was retained.

Structural analysis of lipids. Lipids of interest were fragmented for structural analysis in data dependent acquisition (DDA) mode, where sequential MS^2 or MS^3 scans were triggered

following identification of precursor mass in the first full scan event. Target mass ion (precursor or product ion) isolation and fragmentation was done in the high pressure cell of Velos Pro and accurate mass measurements on resulting product ions acquired in the Orbitrap at 30,000 resolution. Parameters for target mass ion isolation and fragmentation were: isolation width 1.4 Da, collision energy 35 to 50 %, activation Q of 0.25 and activation time 10 ms. Helium was used as collision gas.

Targeted analysis of lipids. For oxPL, lipid extracts (20 µL) were separated on a LUNA RP C_{18} column (150×2.1 mm, 3 µm particle size) using a binary solvent gradient of mobile phase A (methanol:acetonitrile:water 60:20:20, v/v/v, 1 mM ammonium acetate) and B (methanol 100 %, 1mM ammonium acetate) at a flow rate 0.2 ml/min over 50 min. The elution gradient of B (%) over time was: 50 to 100 % for 10 min, 100 % for 30 min, reduced to 50 % in 2 min and held at 50% for next 8 min. Specific precursor-to-product ion transitions are listed in Table S2. Optimized ESI-MS/MS conditions were: source temperature 500 °C, GS1 40, GS2 30, curtain gas (CUR) 20, ion spray voltage (IS) -4500 V, first quadrupole (Q1) at low resolution, third quadrupole (Q3) at unit resolution, dwell time 150 msec, declustering potential (DP) -140 V, entrance potential (EP) -10 V, collision energy (CE) -45 V and collision cell exit potential (CXP) at -7 V. The parent to daughter MRM transitions used are listed in Table S3. Dimyristoyl-PE (DMPE) internal standard was added at 10 ng per sample. Lipids were normalized to internal standards to correct for extraction efficiencies. For fatty acids and eicosanoids, lipids were analyzed using a C18 Spherisorb ODS2, 5 µ, 150 x 4.6 mm column (Waters, Hertfordshire, UK). The mobile phase was gradient of 50–90% B over 10 min (A, water:acetonitrile:acetic acid, 75:25:0.1; B, methanol:acetonitrile:acetic acid, 60:40:0.1) with a flow rate of 1 ml/min. MS was performed using a Sciex 4000 Q-Trap, using DP -55 V, CE -26 V. Internal standards were added at 10 ng per sample as follows: PGE_2-*d4*, 12-HETE-*d8* and AA-*d8*. Lipids were normalized to internal standards to correct for extraction efficiencies. For oxidized FA, MRM

transitions were determined using the MS/MS spectra for each lipid, while unoxidized FA were analyzed by LC/MS, using the parent mass and scanning in Q1 mode.

Annexin V binding to platelets. Platelets were incubated with inhibitors or vehicle in the presence or absence of 5 mM glucose for 120 min. Each sample was analysed by flow cytometry for annexin V binding (Annexin V-FITC; BioLegend). 5 µl of annexin V was added to $2x10^6$ platelets in Tyrodes'buffer containing 3 mM calcium. Platelets were then incubated for 15 min at RT in the dark. Platelets incubated with 4 % paraformaldehyde for 30 min were used as positive control.

References

Foster, J.M., Moreno, P., Fabregat, A., Hermjakob, H., Steinbeck, C., Apweiler, R., Wakelam, M.J., and Vizcaino, J.A. (2013). LipidHome: a database of theoretical lipids optimized for high throughput mass spectrometry lipidomics. PloS one *8*, e61951.

Smith, C.A., O'Maille, G., Want, E.J., Qin, C., Trauger, S.A., Brandon, T.R., Custodio, D.E., Abagyan, R., and Siuzdak, G. (2005). METLIN: a metabolite mass spectral database. Therapeutic drug monitoring *27*, 747-751.

Sud, M., Fahy, E., Cotter, D., Brown, A., Dennis, E.A., Glass, C.K., Merrill, A.H., Jr., Murphy, R.C., Raetz, C.R., Russell, D.W., *et al.* (2007). LMSD: LIPID MAPS structure database. Nucleic acids research *35*, D527-532.

Wishart, D.S., Jewison, T., Guo, A.C., Wilson, M., Knox, C., Liu, Y., Djoumbou, Y., Mandal, R., Aziat, F., Dong, E., *et al.* (2013). HMDB 3.0--The Human Metabolome Database in 2013. Nucleic acids research *41*, D801-807.

Zhang, R., Brennan, M.L., Shen, Z., MacPherson, J.C., Schmitt, D., Molenda, C.E., and Hazen, S.L. (2002). Myeloperoxidase functions as a major enzymatic catalyst for initiation of lipid peroxidation at sites of inflammation. The Journal of biological chemistry *277*, 46116-46122.

**Adduct ion series elimination**			
Negative mode		Positive mode	
**Adduct**	**m/z (amu)**	**Adduct**	**m/z (amu)**
M+Na-2H	20.97357	M+NH_4	10.033823
M+Cl-	34.9694	M+Na	22.989218
M+AcO-	59.0133	M+K	38.963158
2M-H	-1.00783	2M+NH_4	18.033823
		2M+Na	22.989218

**Additional repeating units**	
**Name**	**m/z (amu)**
Quadruply charged ion series (from propanal)	14.5104
Triply charged ion series (from propanal)	19.347
Doubly charged ion series (from propanal)	29.021
PEG related components	44.0262
PPG related components, NaCl	58.0419
Dimethylsiloxane $Si(CH_3)_2O$ from rubber	74.0188
Sodium acetate NaAc	82.003

Table S1, related to Experimental Procedures. List of adducts and contaminating ions removed from the data set.

Parent	RT	ID	Parent	RT	ID	Parent	RT	ID
738.5079	18.9	16:0p/15-HETE-PE	778.503	17.55	18:2a/HETE-PE	798.5291	18.03	18:0a/20:4(2O)-PE
738.5079	19.56	16:0p/11-HETE-PE	778.503	17.56	18:1a/20:5(O)-PE	798.5291	18.31	18:0a/20:4(2O)-PE
738.5079	19.78	16:0p/12-HETE-PE	778.503	17.71	16:0a/HDoHE-PE	798.5291	18.36	18:0p/20:4(3O)-PE
738.5079	20.4	16:0p/8-HETE-PE	778.503	17.75	16:0p/22:6(2O)-PE	798.5291	19.25	18:0a/20:4(2O)-PE
738.5079	20.95	16:0p/HETE-PE	778.503	18.1	18:2a/HETE-PE	798.5291	20.27	18:0a/20:4(2O)-PE
754.5027	16.5	16:0p/AA(2O)-PE	778.503	18.7	16:0p/22:6(2O)-PE	798.5291	20.71	18:0a/20:4(2O)-PE
754.5027	17.22	16:0p/AA(2O)-PE	778.503	20.11	18:1a/20:5(O)-PE	798.5291	20.71	18:0a/20:4(2O)-PE
754.5027	17.84	16:0a/HETE-PE	778.503	20.4	16:1a/22:5(O)-PE	804.5548	16.32	16:1p/22:5(O)-PC
754.5027	17.97	16:0p/AA(2O)-PE	780.5186	16.57	18:1p/20:4(2O)-PE	804.5548	16.32	16:0p/22:6(2O)-PC
754.5027	18.61	16:0a/12-HETE-PE	780.5186	16.79	16:0p/22:5(2O)-PE	804.5548	16.79	18:2a/22:5(O)-PE
754.5027	18.88	16:0p/AA(2O)-PE	780.5186	17.25	18:1p/20:4(2O)-PE	804.5548	16.79	18:1p/22:6(2O)-PE
762.508	17.67	18:2p/HETE-PE	780.5186	18.07	18:1p/20:4(2O)-PE	804.5548	17.54	18:1a/HDoHE-PE
762.508	18.19	16:0p/HDoHE-PE	780.5186	18.11	16:0a/22:5(O)-PE	804.5548	17.8	18:1a/HDoHE-PE
762.508	18.71	16:0p/HDoHE-PE	780.5186	18.59	18:1a/12-HETE-PE	804.5548	18.14	18:1a/HDoHE-PE
762.508	18.88	16:0p/HDoHE-PE	780.5186	18.97	18:1p/20:4(2O)-PE	806.5342	16.77	18:2a/HETE-PC
762.508	21.02	16:0p/HDoHE-PE	780.5186	19.74	18:0a/20:5(O)-PE	806.5342	17.12	18:1p/22:5(2O)-PE
762.508	21.23	18:1p/20:5(O)-PE	780.5186	22.72	18:0a/20:5(O)-PE	806.5342	17.8	16:0a/HDoHE-PC
764.5238	18.82	16:0p/22:5(O)-PE	782.5341	19.36	18:0p/20:(2O)-PE	806.5342	17.84	18:2a/22:4(O)-PE
764.5238	19.2	16:0p/22:5(O)-PE	782.5341	19.62	16:0a/22:4(O)-PE	806.5342	18.3	18:1a/22:5(O)-PE
764.5238	19.68	18:1p/12-HETE-PE	782.5341	20.11	16:0a/12-HETE-PC	806.5342	18.61	18:0p/22:6(2O)-PE
764.5238	21.1	18:0p/20:5(O)-PE	782.5341	21.02	18:0a/12-HETE-PE	806.5342	19.36	18:0a/HDoHE-PE
766.5392	19.7	16:0e/22:5(O)-PE	790.5392	19.18	18:1p/22:5(O)-PE	806.5342	19.66	18:0a/HDoHE-PE
766.5392	20.88	16:0p/22:4(O)-PE	790.5392	20.38	18:0p/HDoHE-PE	806.5342	20.04	18:0a/HDoHE-PE
766.5392	21.33	18:0p/15-HETE-PE	790.5392	20.75	18:0p/HDoHE-PE	808.5501	18.45	16:0a/22:5(O)-PC
766.5392	22.38	18:0p/12-HETE-PE	792.5547	20.88	18:1p/22:4(O)-PE	808.5501	18.9	18:1a/12-HETE-PC
770.4978	13.42	16:0p/20:4(3O)-PE	792.5547	21.27	18:0p/22:5(O)-PE	808.5501	19.23	18:0p/22:5(2O)-PE
770.4978	13.81	16:0p/20:4(3O)-PE	792.5547	21.73	18:0p/22:5(O)-PE	808.5501	19.9	18:1a/22:4(O)-PE
770.4978	14.85	16:0p/DXA3-PE	792.5547	22.08	18:0p/22:5(O)-PE	808.5501	20.99	18:0a/22:5(O)-PE
770.4978	15.41	16:0a/20:4(2O)-PE	794.5701	23.87	18:0p/22:4(O)-PE	808.5501	21.28	20:1a/12-HETE-PE
770.4978	16.05	16:0a/20:4(2O)-PE	794.5701	25.8	20:0p/HETE-PE	810.5657	20.95	18:0p/22:4(2O)-PE
770.4978	16.1	16:0p/20:4(3O)-PE	796.5134	13.9	18:1p/20:4(3O)-PE	810.5657	21.12	18:0a/12-HETE-PC
770.4978	17.05	16:0a/20:4(2O)-PE	796.5134	13.97	16:0p/22:5(3O)-PE	814.5242	14.56	18:0a/PGE2/D2-PE
770.4978	17.84	16:0a/20:4(2O)-PE	796.5134	15.29	18:1p/DXA3-PE	814.5242	15.36	18:0p/20:4(4O)-PE
778.503	15.71	16:0p/22:6(2O)-PE	798.5291	16.45	18:0p/20:4(3O)-PE	814.5242	15.58	18:0a/20:4(3O)-PE
778.503	16.34	16:0p/22:6(2O)-PE	798.5291	17.42	18:0p/DXA3-PE	814.5242	15.58	18:0a/DXA3-PE
778.503	16.8	18:2a/HETE-PE	798.5291	17.75	18:0a/20:4(2O)-PE	814.5242	16.64	18:0a/20:4(3O)-PE
778.503	17.04	16:0a/HDoHE-PE	798.5291	17.75	16:0a/20:4(2O)-PC	814.5242	17.58	18:0p/20:4(4O)-PE

Table S2, related to Experimental Procedures. Oxidized phospholipids generated acutely by thrombin activated platelets. All lipids were structurally verified via MS/MS, with spectra included in Data S5. Retention time is based on elution on Orbitrap Elite.

Parent	Daughter	Name	Parent	Daughter	Name	Parent	Daughter	Name
738.6	219.1	16:0p/15-HETE-PE	778.6	317.2	18:1a/20:5(O)-PE	804.7	345.2	18:2a/22:5(O)-PE
738.6	167.1	16:0p/11-HETE-PE	778.6	345.2	16:1a/22:5(O)-PE	804.7	359.2	18:1p/22:6(2O)-PE
738.6	179.1	16:0p/12-HETE-PE	780.6	335.2	18:1p/20:4(2O)-PE	804.7	343.2	18:1a/HDoHE-PE
738.6	155.1	16:0p/8-HETE-PE	780.6	361.2	16:0p/22:5(2O)-PE	804.7	345.2	16:1p/22:5(O)-PC
738.6	319.2	16:0p/HETE-PE	780.6	345.2	16:0a/22:5(O)-PE	804.7	359.2	16:0p/22:6(2O)-PC
754.6	335.2	16:0p/20:4(2O)-PE	780.6	179.1	18:1a/12-HETE-PE	806.7	319.2	18:2a/HETE-PC
754.6	319.2	16:0a/HETE-PE	780.6	317.2	18:0a/20:5(O)-PE	806.7	361.2	18:1p/22:5(2O)-PE
754.6	179.1	16:0a/12-HETE-PE	782.6	335.2	18:0p/20:4(2O)-PE	806.7	343.2	16:0a/HDoHE-PC
762.6	319.2	18:2p/HETE-PE	782.6	347.2	16:0a/22:4(O)-PE	806.7	347.2	18:2a/22:4(O)-PE
762.6	343.2	16:0p/HDoHE-PE	782.6	179.1	16:0a/12-HETE-PC	806.7	345.2	18:1a/22:5(O)-PE
762.6	317.2	18:1p/20:5(O)-PE	782.6	179.1	18:0a/12-HETE-PE	806.7	359.2	18:0p/22:6(2O)-PE
764.6	345.2	16:0p/22:5(O)-PE	790.6	345.2	18:1p/22:5(O)-PE	806.7	343.2	18:0a/HDoHE-PE
764.6	179.1	18:1p/12-HETE-PE	790.6	343.2	18:0p/HDoHE-PE	808.7	345.2	16:0a/22:5(O)-PC
764.6	317.2	18:0p/20:5(O)-PE	792.6	347.2	18:1p/22:4(O)-PE	808.7	179.2	18:1a/12-HETE-PC
766.6	345.2	16:0e/22:5(O)-PE	792.6	345.2	18:0p/22:5(O)-PE	808.7	361.2	18:0p/22:5(2O)-PE
766.6	347.2	16:0p/22:4(O)-PE	794.6	347.2	18:0p/22:4(O)-PE	808.7	347.2	18:1a/22:4(O)-PE
766.6	219.1	18:0p/15-HETE-PE	794.6	319.2	20:0p/HETE-PE	808.7	345.2	18:0a/22:5(O)-PE
766.6	179.1	18:0p/12-HETE-PE	796.6	351.2	18:1p/20:4(3O)-PE	808.7	179.1	20:1a/12-HETE-PE
770.6	351.2	16:0p/20:4(3O)-PE	796.6	377.2	16:0p/22:5(3O)-PE	810.7	359.2	18:0p/22:4(2O)-PE
770.6	351.2	16:0p/DXA3-PE	796.6	351.2	18:1p/DXA3-PE	810.7	179.1	18:0a/12-HETE-PC
770.6	335.2	16:0a/20:4(2O)-PE	798.6	16.45	18:0p/20:4(3O)-PE	814.7	271.1	18:0a/PGE2/D2-PE
778.6	359.2	16:0p/22:6(2O)-PE	798.6	351.2	18:0p/DXA3-PE	814.7	367.2	18:0p/20:4(4O)-PE
778.6	319.2	18:2a/HETE-PE	798.6	335.2	18:0a/20:4(2O)-PE	814.7	351.2	18:0a/20:4(3O)-PE
778.6	343.2	16:0a/HDoHE-PE	798.6	335.2	16:0a/20:4(2O)-PC	814.7	351.2	18:0a/DXA3-PE

Table S3, related to Experimental Procedures. Parent to daughter transitions used for measuring oxPL generation by platelets. OxPL generation was measured using a 6500 Q-Trap as outlined in Methods. Note that several ions are detected more than once due to the presence of isobaric lipids, as seen in Table S2.

m/z	Full Name	Abbreviation/casual name	m/z	Full Name	Abbreviation/casual name
239.1656	Hydroxytetradecadienoic acid.	HTDE mixture	331.2645	Docosatetraenoic acid.	Adrenic
251.2021	Hexadecadienoic acid.	Palmitolinoleic	333.2073	Prostaglandin B2-like, 4.3min.	PGB2-like, 4.3 min
253.2177	Hexadecenoic acid isomer, 16.5min.	Palmitoleic isomer, 16.5 min	333.2073	Prostaglandin B2-like, 4.8min.	PGB2-like, 4.8 min
253.2177	Hexadecenoic acid isomer, 16.9min.	Palmitoleic isomer, 16.9 min	333.2073	Unknown m/z 333.2, 5.2min	Unknown m/z 333.2, 5.2 min
255.2332	Hexadecanoic acid.	Palmitic	333.2073	Unknown m/z 333.2, 5.9 min	Unknown m/z 333.2, 5.9 min
275.2019	Octadecatetraenoic acid.	Stearidonic	333.2802	Docosatrienoic acid isomer, 13.6min.	DTrA, 13.6 min
277.2176	Octadecatrienoic acid.	α-Linolenic	333.2802	Docosatrienoic acid isomer, 14.0min.	DTrA, 14.0 min
279.1964	12-Hydroxyheptadecatrienoic acid	12-HHTrE	335.2230	5,15-Dihydroxyeicosatetraenoic acid isomer,4.2min.	5,15-DiHETE isomer, 4.2 min
279.2332	Octadecadienoic acid.	Linoleic	335.2230	5,15-Dihydroxyeicosatetraenoic acid isomer, 4.5min.	5,15-DiHETE isomer, 4.5 min
281.2125	12-Hydroxyheptadecadienoic acid	12-HHDE	335.2230	Dihydroxyeicosatetraenoic acid-like, 5.0min.	DiHETE -like, 5.0 min
281.2486	Octadecenoic acid.	Oleic	335.2230	Dihydroxyeicosatetraenoic acid-like, 5.9min.	DiHETE -like, 5.9 min
283.2641	Octadecanoic acid.	Stearic	335.2230	Hepoxilin B3-like.	Hepoxilin B3 -like
291.1969	Hydroxyoctadecatetraenoic acid.	HOTE	335.2955	Docosadienoic acid.	DDA
293.2125	Hydroxyoctadecatrienoic acid.	HOTrE	337.2386	Dihydroxyeicosatrienoic acid-like, 4.9min.	DiHETrE -like, 4.9 min
295.2279	9/13-Hydroxyoctadecadienoic acid mixture.	9/13-HODE mixture	337.2386	Dihydroxyeicosatrienoic acid-like, 6.1min.	DiHETrE -like, 6.1 min
297.2435	9-Hydroxyoctadecenoic acid.	9-HOME	337.2386	Deoxy-ProstaglandinF2a-like.	Deoxy-PGF2a-like.
295.2644	Nonadecenoic acid .	NMA	341.2104	Unknown m/z 341.2, 9.9 min	Unknown m/z 341.2, 9.9 min
297.2801	Nonadecanoic acid.	NA	343.2282	hydroxy-docosahexaenoic acid mixture	HDoHE mixture
301.2176	Eicosapentaenoic acid.	EPA	343.2282	14-Hydroxydocosahexaenoic acid.	14-HDoHE
303.2331	Eicosatetraenoic acid.	Arachidonic	345.2438	20-Hydroxydocosapentaenoic (n-3) acid.	20-HDoPE (n-3)
305.2126	14-Hydroxynonadecatetraenoic acid isomer.	14-HNTE isomer	345.2438	17-Hydroxydocosapentaenoic (n-3) acid.	17-HDoPE (n-3)
305.2126	14-Hydroxynonadecatetraenoic acid.	14-HNTE	345.2438	14-Hydroxydocosapentaenoic (n-3) acid.	14-HDoPE (n-3)
305.2486	Eicosatrienoic acid isome, 18.7min.	ETrA isomer, 18.7 min	345.2438	14-Hydroxydocosapentaenoic (n-6) acid.	14-HDoPE (n-6)
305.2486	Eicosatrienoic acid isomer, 19.3min.	ETrA isomer, 19.3 min	347.2595	17-Hydroxydocosatetraenoic (n-3) acid.	17-HDoTE (n-3)
307.1918	Dihydroyxyoctadecatetraenoic acid -like	DiHOTE -like	347.2595	13-Hydroxydocosatetraenoic (n-3) acid.	13-HDoTE (n-3)
307.2282	14-Hydroxynonadecatrienoic acid.	14-HNTrE	347.2595	14-Hydroxydocosatetraenoic (n-3) acid.	14-HDoTE (n-3)
307.2644	Eicosadienoic acid.	EDA	349.2752	14-Hydroxydocosatrienoic (n-6) acid.	14-HDoTrE (n-6)
309.2438	14-Hydroxynonadecadienoic acid.	14-HNDE	349.2752	14-Hydroxydocosatrienoic (n-9) acid.	14-HDoTrE (n-9)
309.2801	Eicosenoic acid.	EMA	351.2180	Prostaglandin E2.	PGE2
311.2957	Eicosanoic acid.	Arachidic	351.2180	Prostaglandin D2.	PGD2
315.1970	Prostaglandin A2-like.	PGA2-like, 4.1 min	351.2180	Prostanoid m/z 351.2, 2.8 min	Prostanoid m/z 351.2, 2.8 min
315.1970	Prostaglandin A2-like.	PGA2-like, 4.3 min	351.2180	Prostanoid m/z 351.2, 3.0 min	Prostanoid m/z 351.2, 3.0 min
317.2125	12-Hydroxyeicosapentaenoic acid.	12-HEPE	351.2180	Prostanoid m/z 351.2, 3.2 min	Prostanoid m/z 351.2, 3.2 min
319.2276	12-Hydroxyeicosatetraenoic acid.	12-HETE	351.2180	DXA3-like.	DXA3-like
321.2437	12-Hydroxyeicosatrienoic acid isomer, 11.1min.	12-HETrE isomer, 11.1 min	351.2180	8-hydroxyl-9,11-dioxolane A3.	DXA3
321.2437	12-Hydroxyeicosatrienoic acid isomer,12.1min.	12-HETrE isomer, 12.1 min	353.2335	Prostaglandin F2-like, 2.7min.	PGF2 -like, 2.7 min
323.2595	11-Hydroxyeicosadienoic acid isomer, 12.6min.	11-HEDE isomer, 12.6 min	353.2335	Prostaglandin F2-like, 2.9min.	PGF2 -like, 2.9 min
323.2595	11-Hydroxyeicosadienoic acid isomer, 13.4min.	11-HEDE isomer, 13.4 min	353.2335	Prostaglandin F2-like, 3.2min.	PGF2 -like, 3.2 min
327.2334	Docosahexaenoic acid.	DHA	353.2335	Unknown m/z 353.2, 3.42 min	Unknown m/z 353.2, 3.42 min
329.2486	Docosapentaenoic acid isomer.	DPA	369.2285	Thromboxane B2.	TXB2
329.2486	Docosapentaenoic acid-like.	DPA -like			

Table S4, related to Figure 1H. Structures of fatty acids and eicosanoids generated by thrombin-activated platelets. All these lipids were manually verified via MS/MS on the Orbitrap Elite platform. Spectra are provided in Data S6.

Ion	Mode
[M-H]-	Negative
[M+H]+	Positive
[M+Na]+	Positive
[M+NH_4]+	Positive
[M+CH_3COO]-	Negative

Table S5, related to Experimental Procedures. Adduct ions retained after database searching.

Figure S1

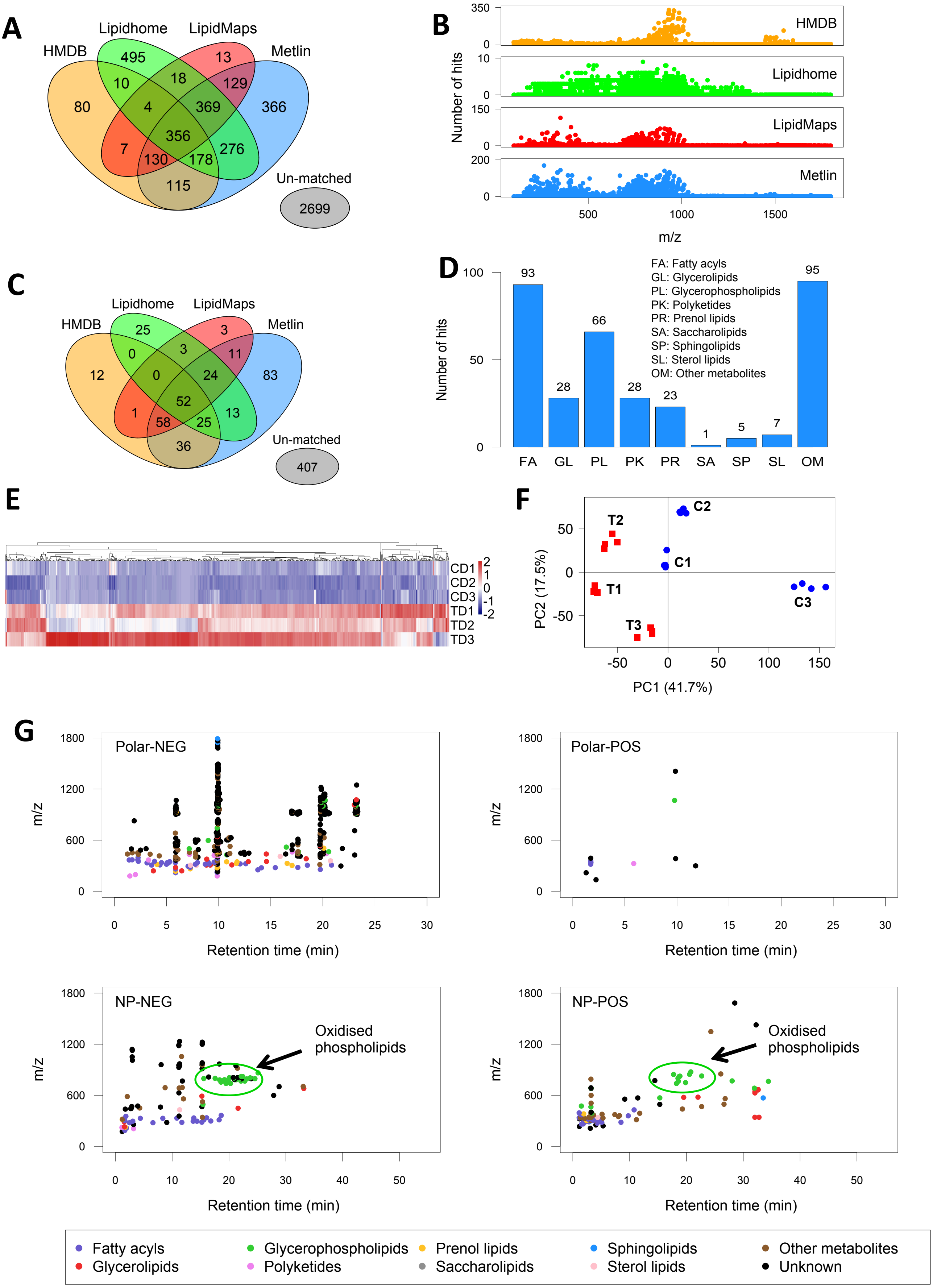
A
HMDB
Lipidhome
LipidMaps
Metlin
495
13
10
18
129
80
4
369
366
356
7
276
130
178
115
Un-matched
2699
B
HMDB
Lipidhome
LipidMaps
Metlin
Number of hits
m/z
350
0
10
0
150
0
200
0
500
1000
1500
C
HMDB
Lipidhome
LipidMaps
Metlin
25
3
0
3
11
12
0
24
83
52
1
13
58
25
36
Un-matched
407
D
Number of hits
FA: Fatty acyls
GL: Glycerolipids
PL: Glycerophospholipids
PK: Polyketides
PR: Prenol lipids
SA: Saccharolipids
SP: Sphingolipids
SL: Sterol lipids
OM: Other metabolites
93
28
66
28
23
1
5
7
95
0
50
100
FA
GL
PL
PK
PR
SA
SP
SL
OM
E
CD1
CD2
CD3
TD1
TD2
TD3
2
1
0
-1
-2
F
PC2 (17.5%)
PC1 (41.7%)
T2
C2
C1
T1
C3
T3
50
0
-50
-50
0
50
100
150
G
Polar-NEG
Polar-POS
NP-NEG
NP-POS
m/z
Retention time (min)
1800
1200
600
0
0
5
10
15
20
25
30
40
50
Oxidised phospholipids
Fatty acyls
Glycerolipids
Glycerophospholipids
Polyketides
Prenol lipids
Saccharolipids
Sphingolipids
Sterol lipids
Other metabolites
Unknown

Figure S1, related to Figure 1. Putative identification of lipids present basally and on thrombin activation of platelets. *Panel A. Results of database searches on basal ions.* Ions common to ≥ 2 donors are shown that were assigned at least one putative match, and those with no match. *Panel B. Number of hits in databases per m/z value*. *Panel C. Results of database searchers on thrombin-upregulated ions*. Ions common to ≥ 2 donors are shown that were assigned at least one putative match, and those with no match. *Panel D. Predominant lipid classes upregulated by thrombin in platelets.* Ions were grouped according to the most predominant classification family based on classifications in databases *Panel E. Heat map showing the diversity of thrombin responses for individual donors*. Lipids that upregulated on thrombin activation from control (C1-3) and thrombin-activated (T1-3) platelet extracts were plotted using a heat map. Note that Donor 3 upregulates many lipids differentially and more strongly than either Donors 1 or 2. *Panel F. Principal Component Analysis showing the effect of thrombin on platelet lipids.* Donors' basal (C1-3) and thrombin-elevated (T1-3) lipids were analyzed, with four technical replicates per donor. Donors are separated in both PC1 and PC2, but thrombin causes similar changes in PC1 for all donors. *Panel G. Scatter diagrams showing elution of lipids from Polar or NP columns, in either negative or positive ion mode*. Lipids are color coded according to classification from databases. Putative identifications for all are in Supplementary Data File 1.

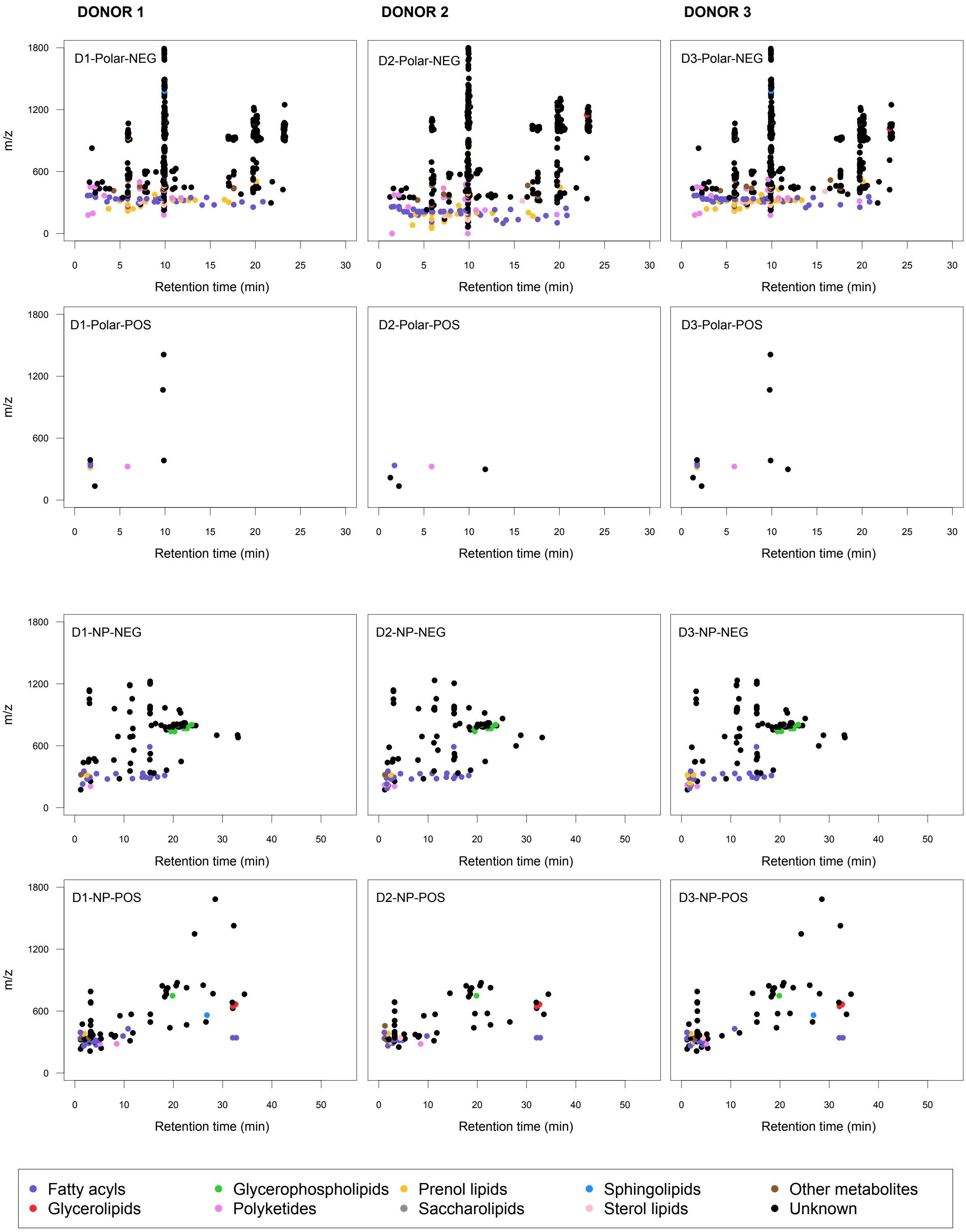

Figure S2, related to Figure 1. Donor specific thrombin-upregulated lipids shown as scatter diagrams. Scatter diagrams were generated as described in Figure 2 H, following LC/FTMS analysis of control and thrombin-activated platelet lipid extracts as described in Methods.

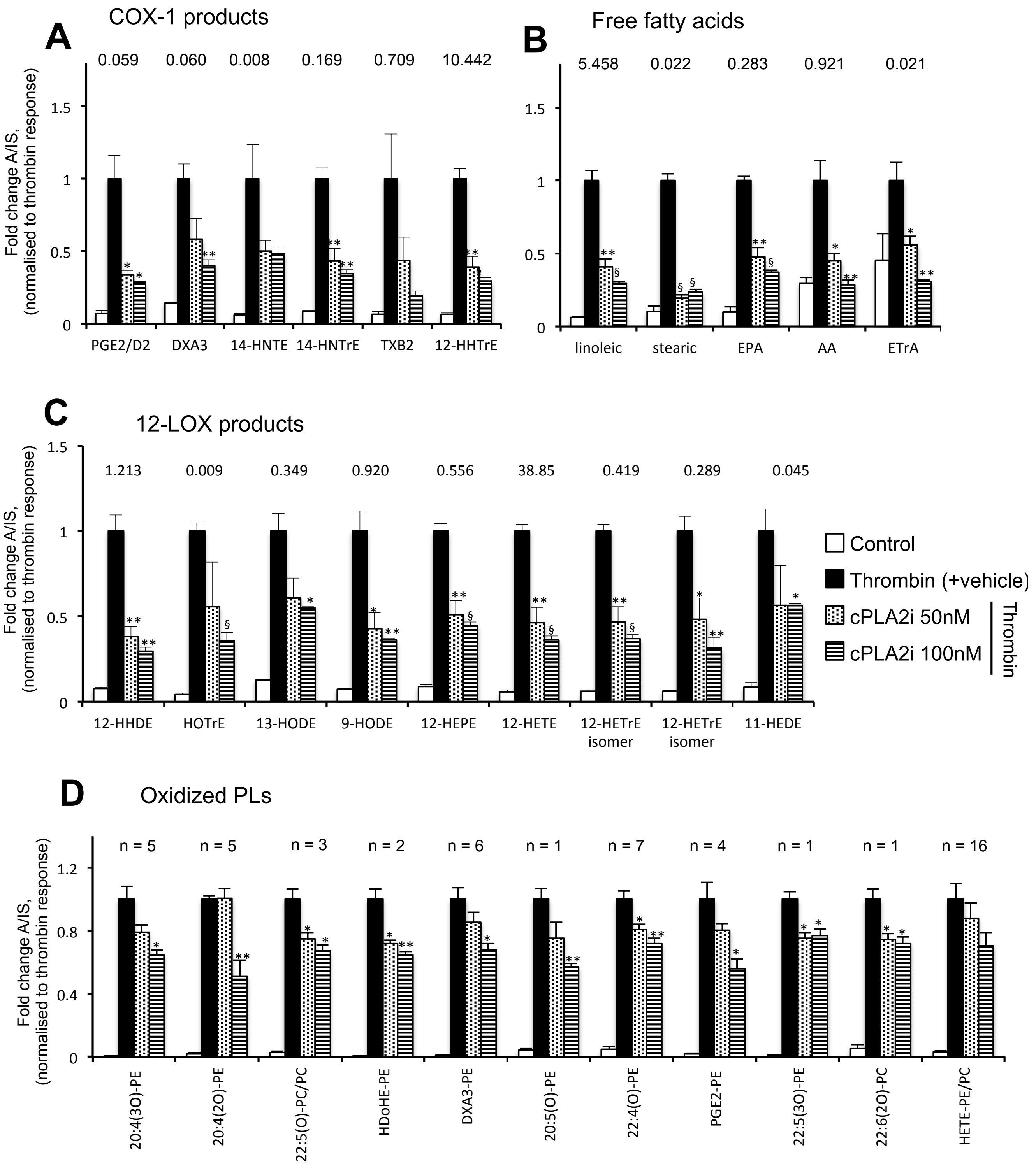

Figure S3, related to Figure 5. Inhibition of cPLA$_2$ suppresses generation of FAs and oxPLs. Washed platelets were incubated with 50-100 nM cPLA$_2$i or vehicle for 15 min, then activated using 0.2 U/ml thrombin for 30 min and generation of lipids determined using LC/MS/MS as described in Supplementary Methods. For Panel D, oxPL are grouped depending on the *sn2* fatty acid to aid data visualization. Individual lipids for this data are shown in Supplementary 5. Figure A-C: Numbers above each refer to the analyte:internal standard integrated area for the thrombin activated sample.

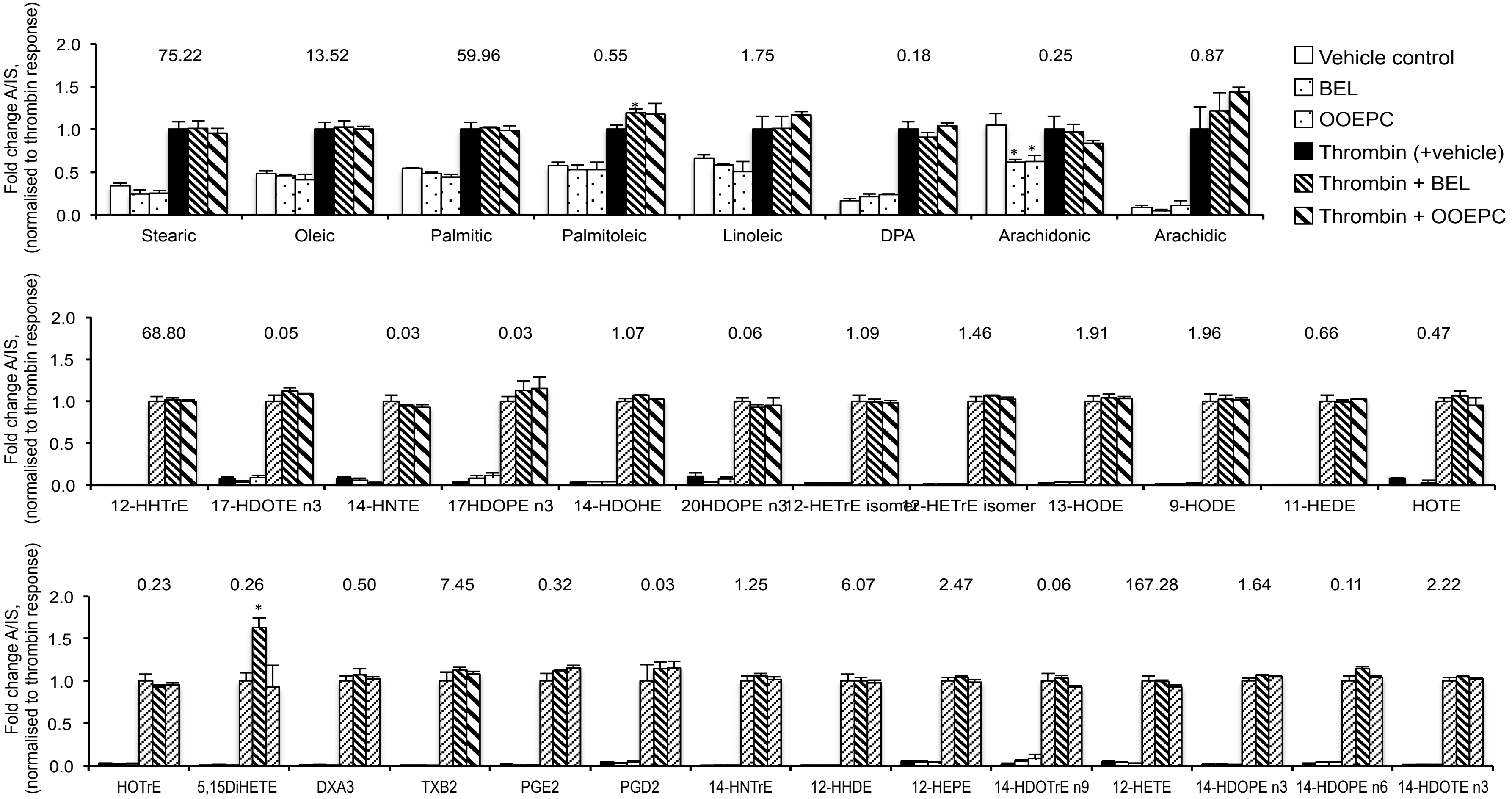

Figure S4, related to Figure 5. Effect of PLA_2 inhibitors on generation of FAs by platelets. Washed platelets were incubated with inhibitors or vehicle for 15 min, then activated using 0.2 U/ml thrombin for 30 min and generation of lipids determined using LC/MS/MS as described in Supplementary Methods. Inhibitors were as follows: $iPLA_2$: 50 nM BEL and $sPLA_2$: 2 μM OOEPC. Numbers above each refer to the analyte:internal standard integrated area for the thrombin activated sample.

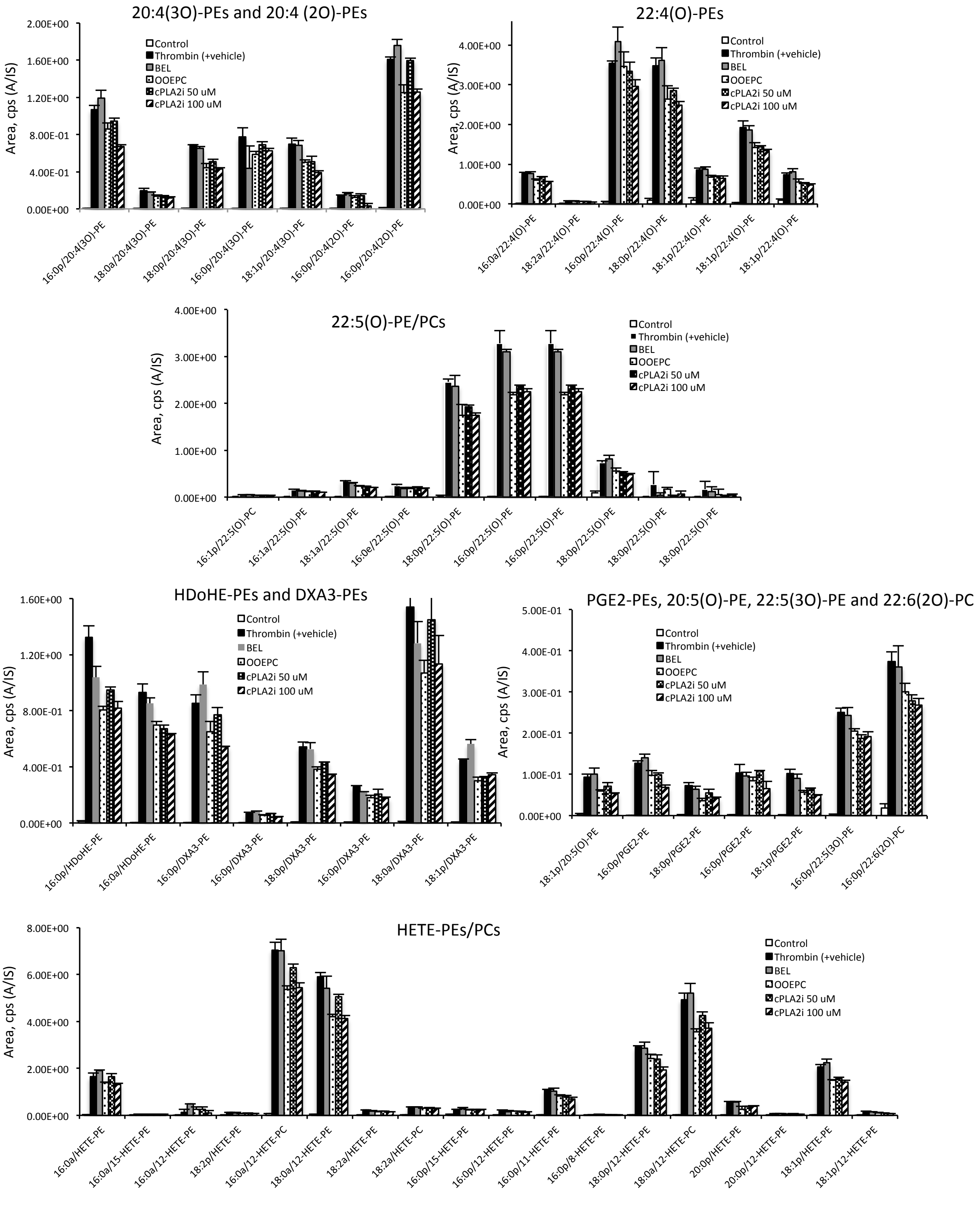

Figure S5, related to Figure 5. Bar charts showing effects of PLA_2 inhibitors on levels of individual oxPL. Lipids were analyzed by LC/MS/MS, on a 6500 Q-Trap, as described in Methods. Some of this data is combined based on *sn2* functional group and summarized in Supplementary Figure 3 D.

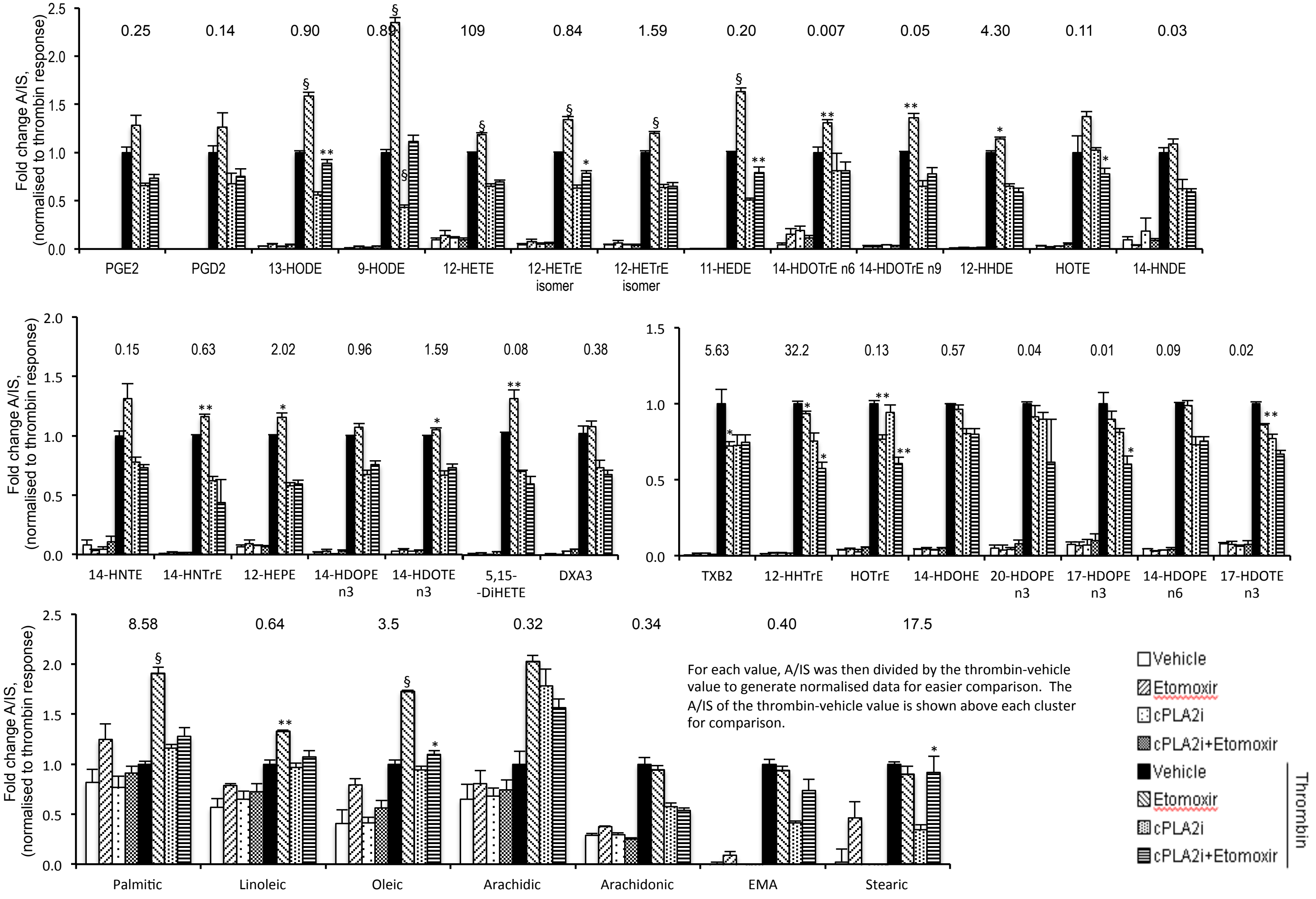

Figure S6, related to Figure 6. Several eicosanoids are substrates for platelet β-oxidation on thrombin generation. Washed platelets were incubated with inhibitors or vehicle, then activated using 0.2 U/ml thrombin at 37 ^{0}C for 30 min and generation of lipids determined using LC/MS/MS as described in Supplementary Methods (n = 3). Inhibitors were as follows: Etomoxir (Eto): 25 µM, cPLA$_2$i: 100 nM. n = 3, mean ± SEM, 1-way ANOVA with Bonferroni post hoc test. Numbers above each refer to the analyte:internal standard integrated area for the thrombin activated sample.

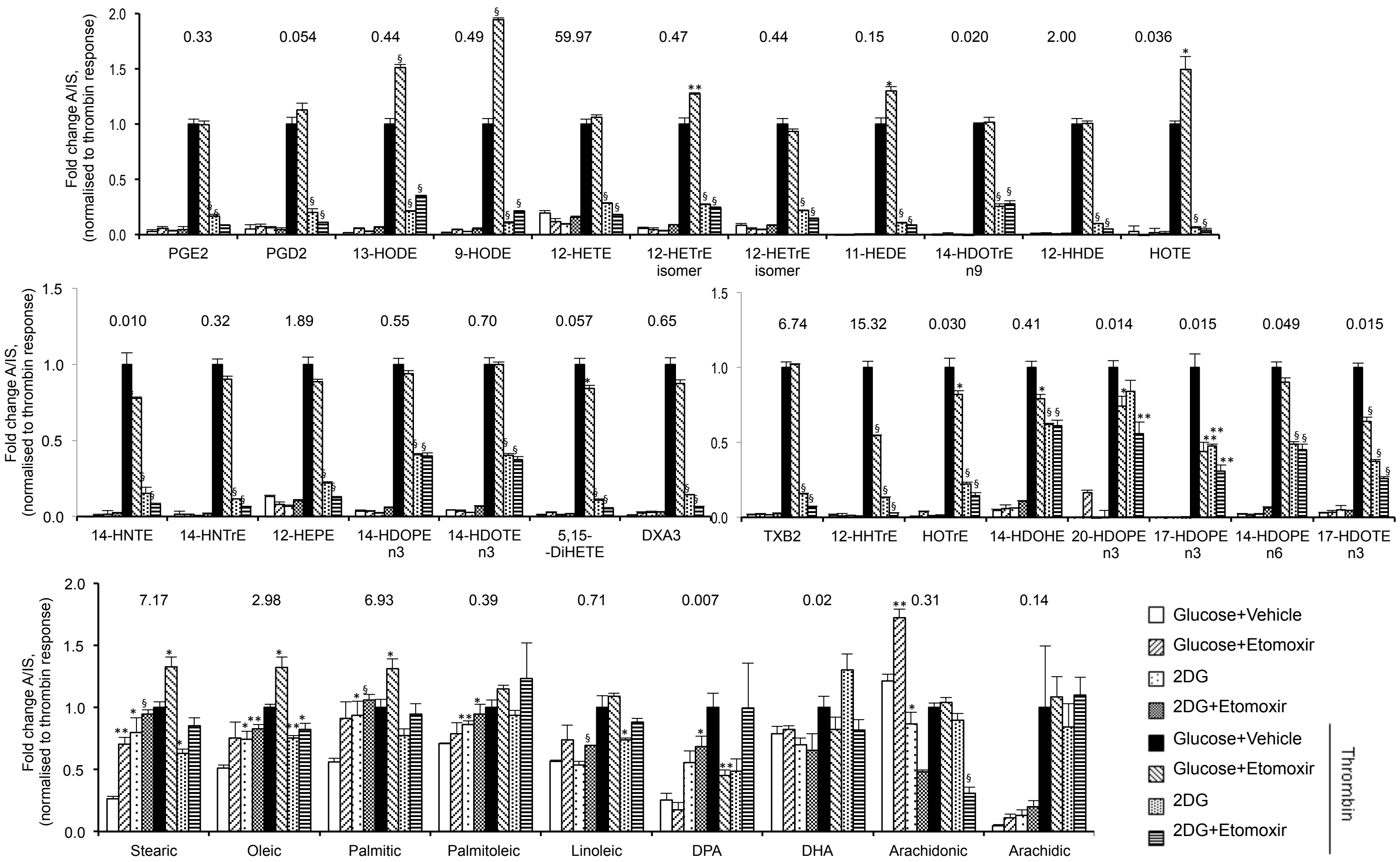

Figure S7, related to Figure 6. Inhibition of glycolysis leads to a failure to generate free fatty acids and eicosanoids on thrombin activation of platelets. Washed platelets were incubated with inhibitors or vehicle for 60 min at RT, then activated using 0.2 U/ml thrombin at 37 ^{0}C for 30 min and generation of lipids determined using LC/MS/MS as described in Supplementary Methods (n = 3). Cells were either incubated in the presence (Glc) or absence of 5 mM glucose (Glc). Inhibitors were as follows: Eto: 25 µM, 2-deoxy-D-glucose (2DG): 120 mM. n = 3, mean ± SEM, 1-way ANOVA with Bonferroni post hoc test. Numbers above each refer to the analyte:internal standard integrated area for the thrombin activated sample.

Viewing GoogleVis files in a browser

To view GoogleVis interactive html files in a browser the settings for Adobe (Macromedia) Flash need to be updated.

Open the URL:
http://www.macromedia.com/support/documentation/en/flashplayer/help/settings_manager04.html

The Global Security Settings panel will be displayed as below.
Click on the third tab (Global Security Settings).

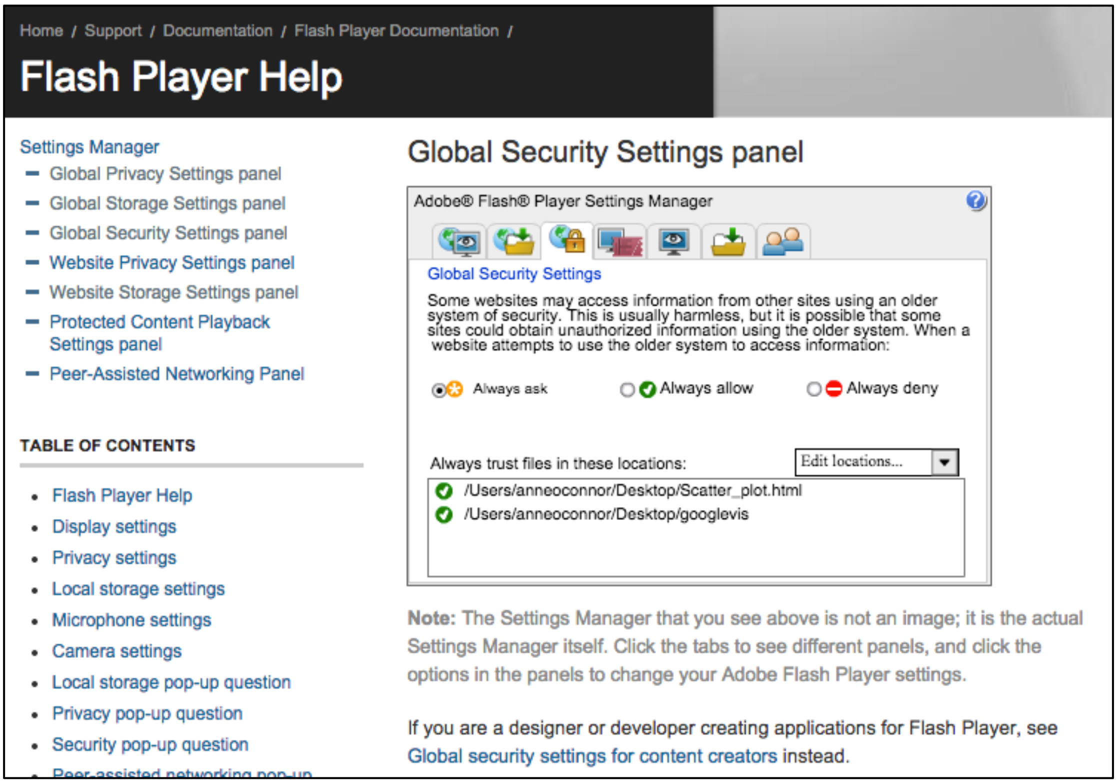

In the 'Edit Locations' dropdown box, select 'Add Location'.
Select 'Browse for files…' (or 'Browse for folder…').

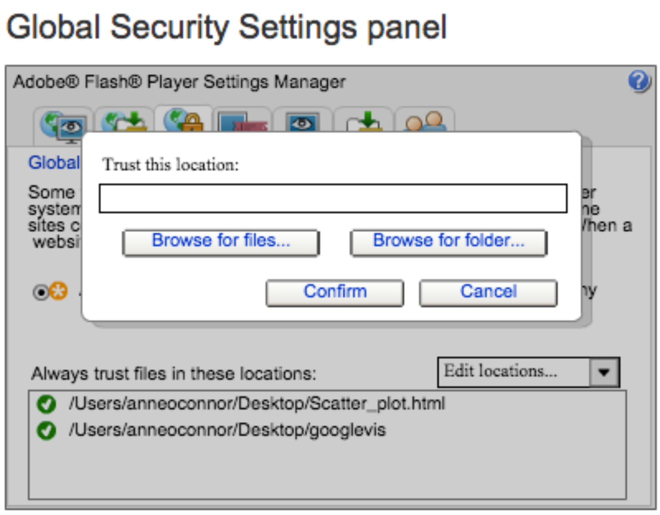

Navigate to the location of the GoogleVis html file(s), select file(s) and click Open.
The file(s) that you selected should now be listed in the box 'Always trust files in these locations:"

You can now open the html file(s) by clicking on them. Each point on the scatter diagram represents one lipid species. You can hover over a point to get the lipid category for this species, the retention time and the *m/z* value.

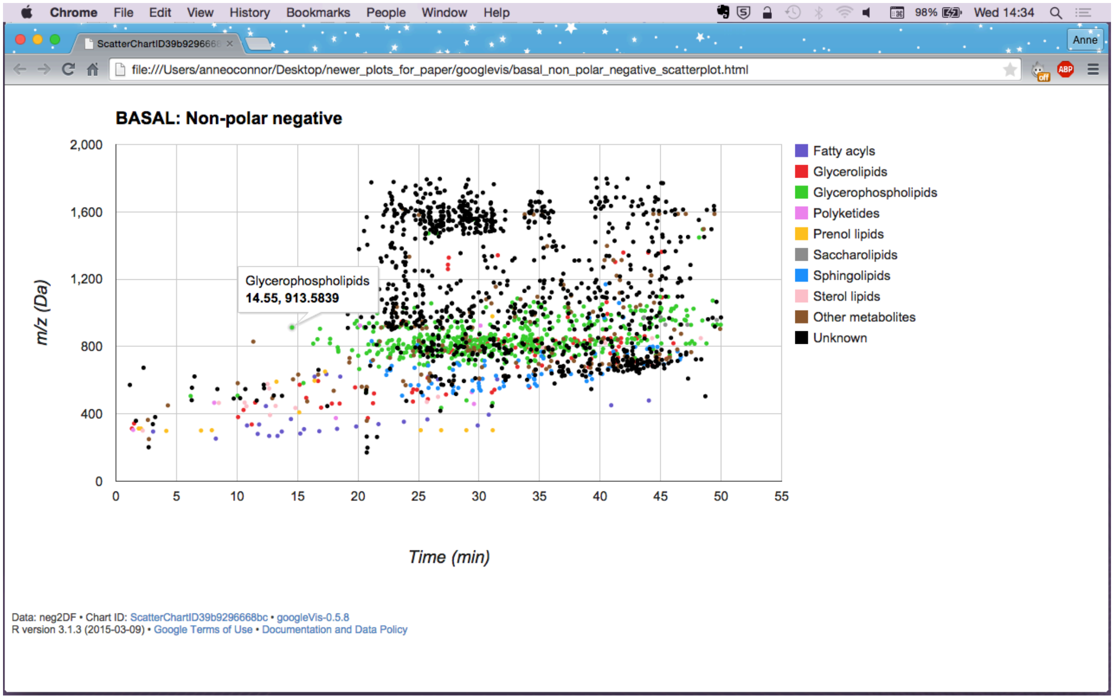

You can zoom in on sections of a plot to see points clearer. Just drag the cursor over an area to zoom into.

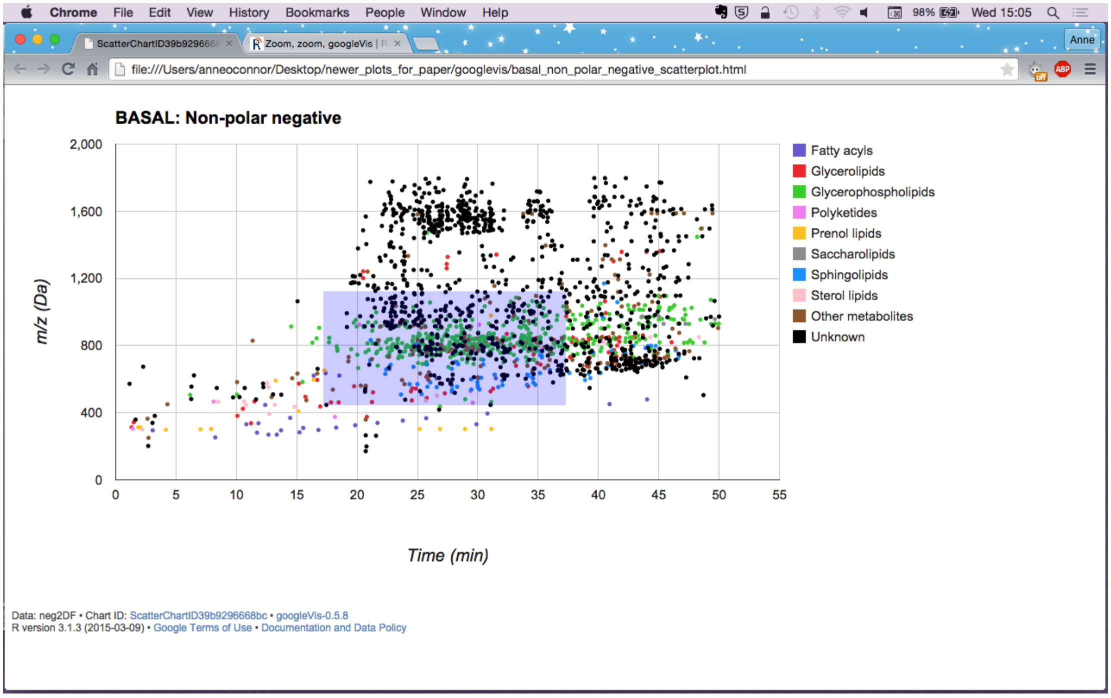

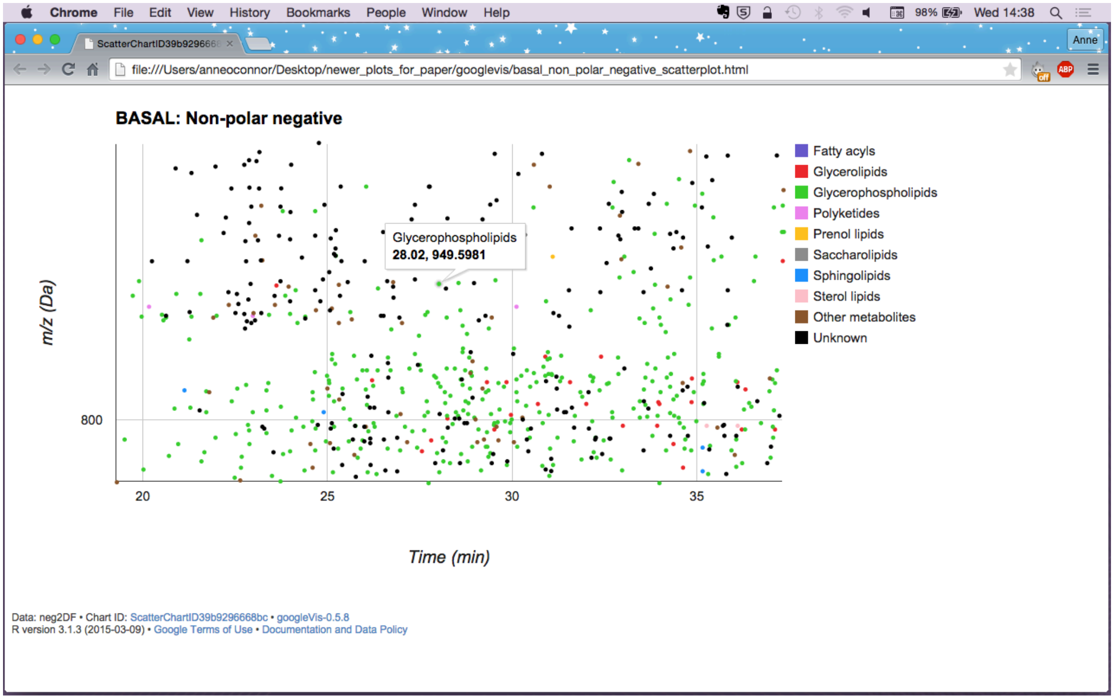

You can also hover over a category in the legend to highlight all points in that category on the scatter diagram.

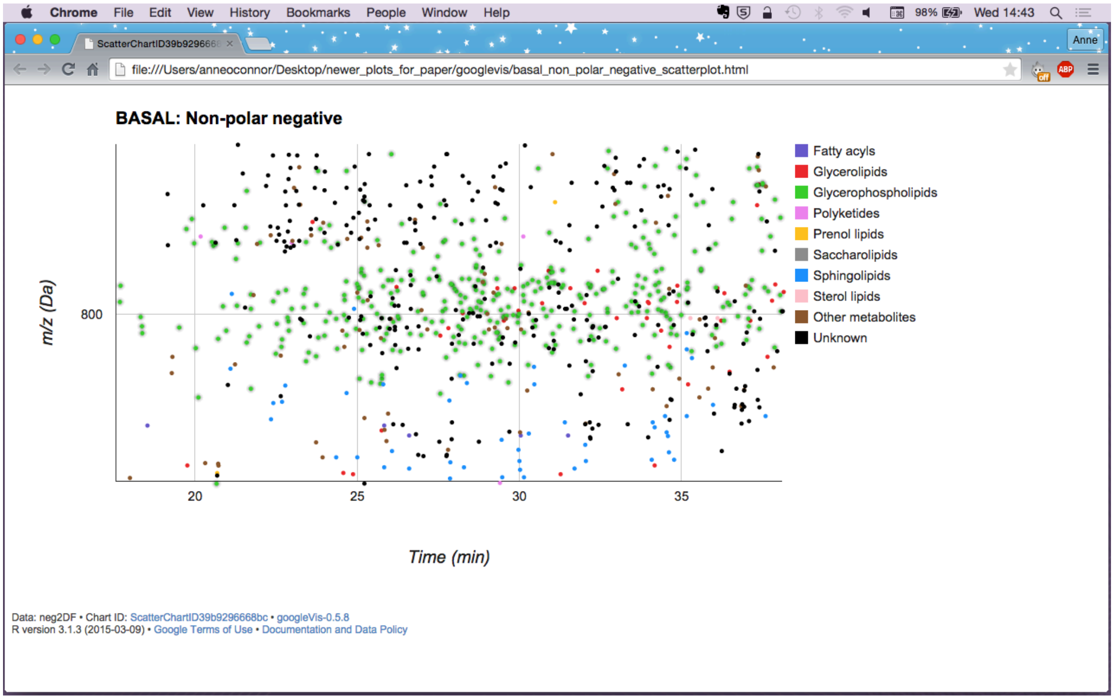

To zoom back out you can just refresh the page or right click.

NOTE: This has been tested on Google Chrome and Safari only.
